# Mendelian Randomization Analysis of Mitochondria‐Related Genes and Screening of Prognostic Genes in Colorectal Cancer

**DOI:** 10.1002/cam4.71012

**Published:** 2025-07-07

**Authors:** Limin Zhu, Xiaowei Huang, Fan Zhang, Jinzu Yang, Zhenye Xu

**Affiliations:** ^1^ Department of Oncology LongHua Hospital, Shanghai University of Traditional Chinese Medicine Shanghai China; ^2^ Department of Nephrology Longhua Hospital Shanghai University of Traditional Chinese Medicine Shanghai China

**Keywords:** colorectal cancer, Mendelian randomization, mitochondria‐related genes, potential targets, prognosis

## Abstract

**Background:**

Mitochondria have been linked with inflammatory colorectal cancer (CRC) development; however, the association between mitochondria‐related genes (MRGs) and CRC remains unknown.

**Aims:**

To explore the causal relationship between MRGs and CRC, screen prognostic genes, conduct drug prediction analyses, and investigate the correlations between prognostic genes and immune cells.

**Materials and Methods:**

We obtained 1136 MRGs from the MitoCarta3.0 database and analyzed the causal relationship between MRGs expression, methylation, and protein abundance and CRC by Mendelian randomization and sensitivity testing. Prognostic genes were screened via protein–protein interaction networks, enrichment, multi‐omics, and survival analyses. Selected key genes were subjected to drug prediction analyses. The prognostic genes and immune cell correlations were explored using Spearman‘s correlation.

**Results:**

The results indicated that 44 MRGs showed causal relationships with CRC. Six genes (sterol carrier protein2 [SCP2], ATP binding cassette subfamily D member 3 [ABCD3], cytochrome coxidase assembly factor heme A: farnesyltransferase [COX10], mitochondrial contact site and cristae organizing system subunit 10 [MiCOS 10], glutaryl‐Coenzyme A dehydrogenase [GCDH], and mitochondrial translational release factor 1‐like [MTRF1L] were causally associated with CRC and showed better prognostic significance when their expression levels were high, and there were 106 drugs targeting them. SCP2, ABCD3, MICOS10, GCDH, and MTRF1L were associated with most immune cells, while COX10 was not associated with any of the 96 immune cells.

**Discussion:**

The identification of causal MRGs and their prognostic significance provides new insights into mitochondria‘s role in CRC. Drug prediction and immune correlations may guide therapy, but validation in larger cohorts and models is needed.

**Conclusion:**

This study reveals causal associations between specific MRGs and CRC, identifies prognostic genes with therapeutic potential, and clarifies immune cell relationships, advancing CRC pathogenesis understanding and treatment development.

## 1 Introduction

Colorectal cancer (CRC), which includes rectal and colon cancers, ranks third and second globally in terms of incidence and mortality, accounting for 9.8%and 9.2% [1], respectively. The incidence and mortality rates of CRC are increasing rapidly. Approximately 3.2 million new cases of CRC are projected to occur globally and the death toll is expected to exceed 1–6 million by 2040 [2, 3, 4]. Currently, CRC treatment primarily relies on surgery, radiotherapy, chemotherapy, immunotherapy, and targeted drug therapy [5, 6]. Although the overall efficacy of CRC has improved, the efficacy of advanced CRC remains unsatisfactory, with a recurrence rate as high as 54.5% and poor patient prognosis [7, 8, 9].

Mitochondria are major producers of energy in mammalian cells and are involved in cell differentiation, signal transduction, cell cycle regulation, apoptosis, and tumorigenesis. They provide energy through the tricarboxylic acid (TCA) cycle and oxidative phosphorylation (OXPHOS) of adenosine triphosphate (ATP) [10]. Mitochondria are crucial for tumorigenesis because they support the growth and survival of tumor cells in hostile environments such as nutrient depletion and hypoxia, thus highlighting their importance as mediators of tumorigenesis [11]. Mild mitochondrial dysfunction can promote cancer cell growth and invasion, whereas severe dysfunction can cause cell death, thereby inhibiting tumor occurrence [12]. The mitochondrial genome includes over 1000 additional genes. Genetic predisposition to these genes may lead to mitochondrial dysfunction, thereby affecting the occurrence and development of tumors [13].

Several studies have linked mitochondria‐related genes (MRGs) to cancer development. Elevated D‐2‐hydroxyglutarate levels are biomarkers of many cancers [14]. Isocitrate dehydrogenase (NADP (+)) 1 is the most frequently mutated gene in cancer [15]. Previous studies have found that mitochondrial autophagy genes may predict the prognosis of patients with uveal melanoma [16]. Alpha‐ketoglutarate, which participates in the TCA cycle, is a potent anti‐tumor metabolite that can directly regulate the signaling pathway of CRC and be used for potential differentiation therapy in patients with CRC [17]. Fang et al. analyzed bioinformatics data to find links between MRGs and breast cancer prognosis and metastasis, discovering a significant impact [18]. As a gatekeeper of mitochondria, the voltage‐dependent anion channel 1 (*VDAC1*) is associated with the development of breast cancer. Overexpressed VDAC1 in breast cancer could be served as a novel biomarker for diagnosis and VDAC1 was an independent factor for adverse prognosis prediction [19]. The abnormal expression of mitochondrial ATP‐binding cassette subfamily D member 3 (*ABCD3*) is a standalone prognostic factor for CRC [20]. Although mitochondria are closely related to cancer, current research on the causal relationship between MRGs and CRC remains insufficient. Studying the causal relationship between MRGs and CRC and screening prognosis‐related genes may contribute to the development of targeted therapies for CRC.

Mendelian randomization (MR) analysis applies genetic variation as an instrumental variable to strengthen the determination of causal relationships between exposure and outcomes. With the continuous increase in large‐scale genome‐wide association studies (GWAS) and molecular quantitative trait locus (QTL) data, causal relationships between the expression, methylation, and protein abundance of MRGs and CRC can be explored. This study investigated the potential associations of MRGs expression, methylation, and protein abundance with CRC risk using MR analysis, and screened genes associated with CRC prognosis.

## 2 Results

### 2.1 MR Analysis of Causal Relationship Between MRGs Cis‐eQTLs and CRC

**FIGURE 1:**
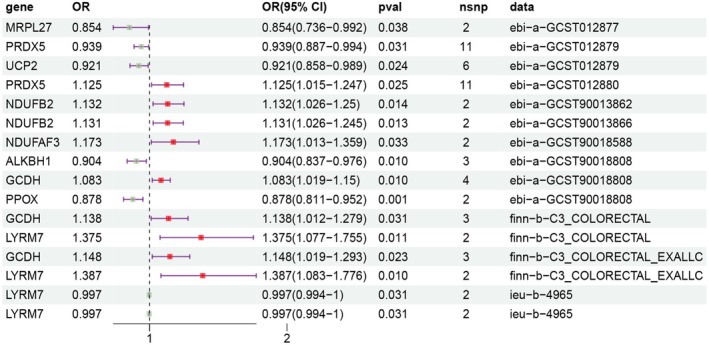
MR results for the causal relationship between the MRGs eQTL and CRC risk. The OR value greater than 1 suggests that the exposure factor may elevate the risk of the outcome, while an OR value less than 1 implies that it may decrease the risk. When the OR value equals 1, it indicates no impact of the exposure factor on the outcome. The 95% CI indicates that we have a 95% confidence that the true value of the OR value falls within this interval. When the width of CI is narrow, it indicates that the estimation of OR value is more accurate. If the *p* value is lower than 0.05, it indicates that this causal association has statistical significance. *ALKBH1*, alkylation repair homolog 1; CI, confidence interval; *GCDH*, glutaryl‐CoA dehydrogenase; *LYRM7*, LYR motif containing 7; *MRPL27*, mitochondrial ribosomal protein L27; *NDUFAF3*, NADH ubiquinone oxidoreductase complex assembly factor 3; *NDUFB2*, NADH ubiquinone oxidoreductase subunit B2; OR, odds ratio; *PPOX*, protoporphyrinogen oxidase; *PRDX5*, peroxiredoxin 5; *UCP2*, uncoupling protein 2.

TwoSampleMR analysis revealed 239 genes with a causal association with CRC (Table S1). Using SMR analysis, 73 genes with causal relationships with CRC were identified (*P*_SMR_ < 0.05) and co‐localized with CRC associations with high evidence support (*P*_HEIDI_ > 0.05) (Table S2). Nine genes (*NDUFB2*, *ALKBH1*, *GCDH*, *MRPL27*, *PRDX5*, *PPOX*, *UCP2*, *NDUFAF3*, and *LYRM7*) simultaneously fulfilled the analytical requirements of TwoSampleMR and SMR (Figure 1). The sensitivity analysis results for heterogeneity and horizontal pleiotropy showed that the MR analysis results were robust (Table S3).

### 2.2 MR Analysis of Causal Relationship Between MRGs Cis‐mQTLs and CRC

**FIGURE 2:**
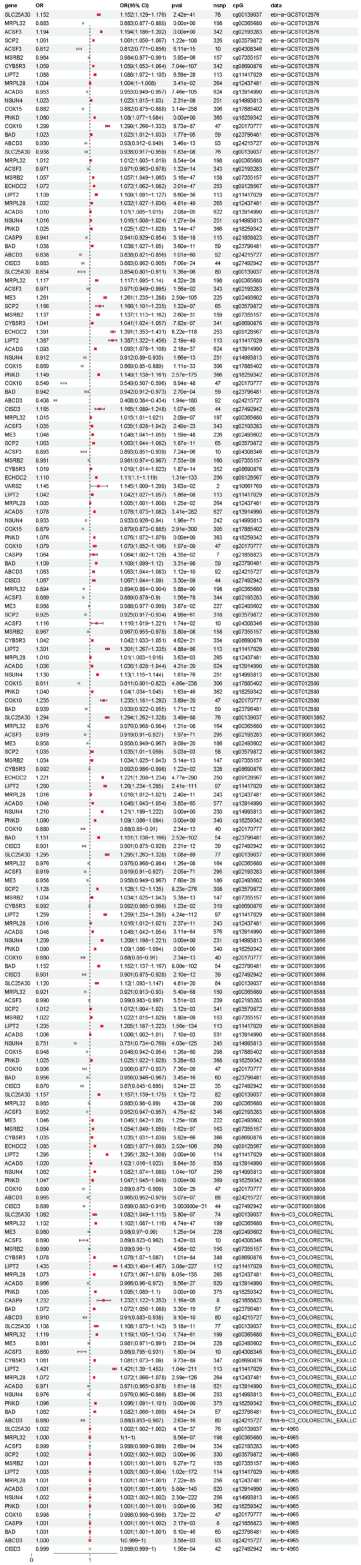
MR results for the causal relationship between the MRGs mQTL and CRC risk. The OR value greater than 1 suggests that the exposure factor may elevate the risk of the outcome, while an OR value less than 1 implies that it may decrease the risk. When the OR value equals 1, it indicates no impact of the exposure factor on the outcome. The 95% CI indicates that we have a 95% confidence that the true value of the OR value falls within this interval. When the width of CI is narrow, it indicates that the estimation of OR value is more accurate. If the *p* value is lower than 0.05, it indicates that this causal association has statistical significance. *ABCD3*, ATP binding cassette subfamily D member 3; *ACADS*, acyl‐CoA dehydrogenase short chain; *ACSF3*, acyl‐CoA synthetase family member 3; *BAD*, BCL2 associated agonist of cell death; *CASP9*, Caspase 9; CI, confidence interval; *CISD3*, CDGSH iron sulfur domain 3; *COX10*, cytochrome c oxidase assembly factor heme A, Farnesyltransferase COX10; *COX15*, cytochrome c oxidase assembly homolog COX15; *CYB5R3*, cytochrome b5 reductase 3; ECHDC2, enoyl‐CoA hydratase domain containing 2; *LIPT2*, lipoyl (octanoyl) transferase 2; *ME3*, Malic enzyme 3; *MRPL28*, mitochondrial ribosomal protein L28; *MRPL32*, mitochondrial ribosomal protein L32; *MSRB2*, methionine sulfoxide reductase B2; *NSUN4*, NOP2/Sun RNA methyltransferase 4; OR, odds ratio; *PNKD*, PNKD metallo‐beta‐lactamase domain containing; *SCP2*, sterol carrier protein 2; *SLC25A30*, solute carrier family 25 member 30; *VARS2*, valyl‐tRNA synthetase 2.

Using TwoSampleMR analysis, 356 DNA methylation sites (annotated to 254 genes) were found to have causal associations with CRC (Table S4). Using SMR analysis, 114 DNA methylation sites (annotated to 80 genes) with causal relationships with CRC were obtained (*P*_SMR_ < 0.05) and co‐localized with CRC associations with high support of evidence (*P*_HEIDI_ > 0.05) (Table S5). Twenty genes (*SLC25A30*, *MRPL32*, *ACSF3*, *ME3*, *SCP2*, *MSRB2*, *CYB5R3*, *ECHDC2*, *VARS2*, *LIPT2*, *MRPL28*, *ACADS*, *NSUN4*, *COX15*, *PNKD*, *COX10*, *CASP9*, *BAD*, *ABCD3*, and *CISD3*) simultaneously fulfilled the analysis requirements of TwoSampleMR and SMR (Figure 2). The sensitivity analysis results for heterogeneity and horizontal pleiotropy showed that the MR analysis results were robust (Table S6).

### 2.3 MR Analysis of Causal Relationship Between MRGs Cis‐pQTLs and CRC

**FIGURE 3:**
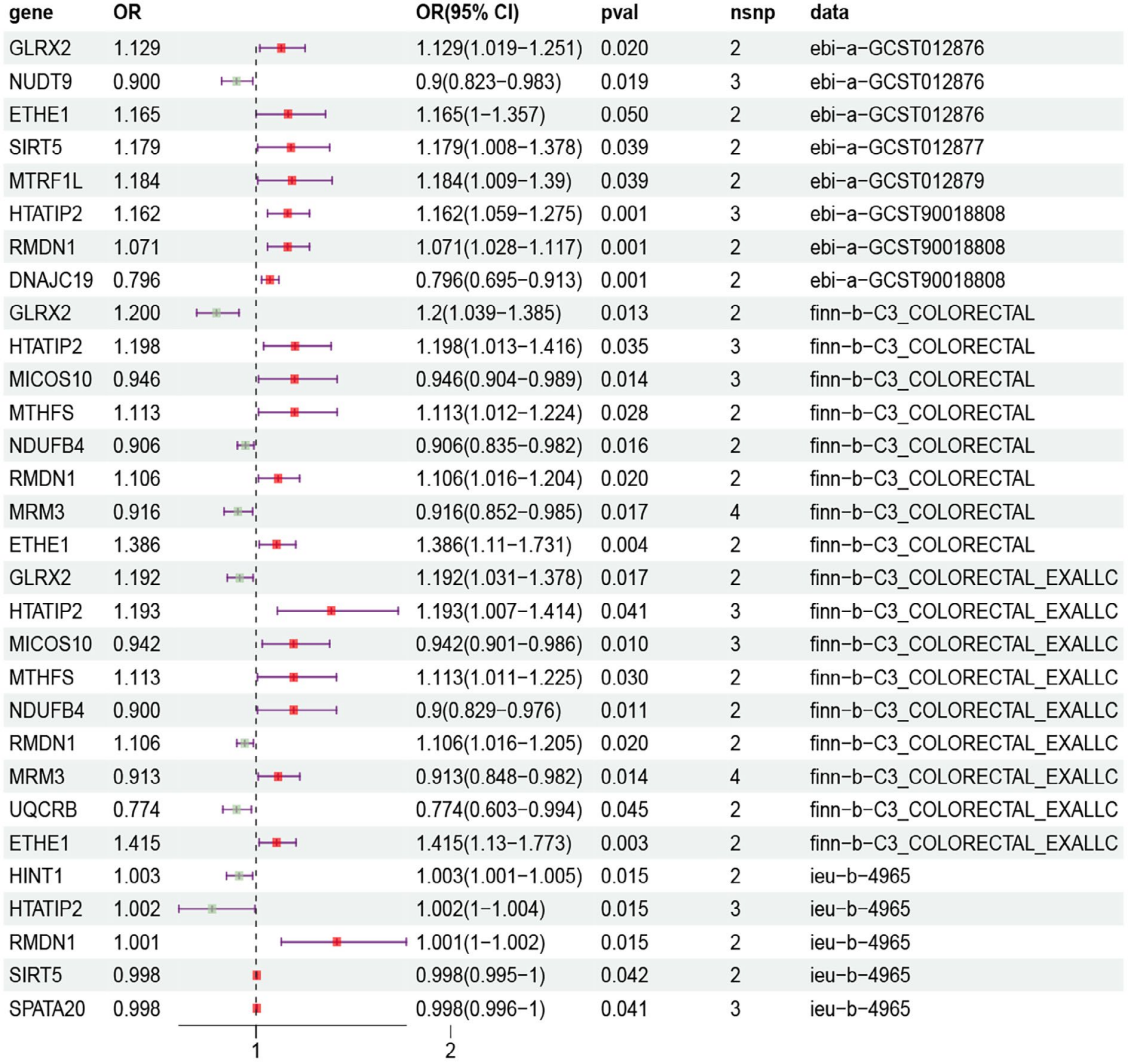
MR results for the causal relationship between the MRGs pQTL and CRC risk. The OR value greater than 1 suggests that the exposure factor may elevate the risk of the outcome, while an OR value less than 1 implies that it may decrease the risk. When the OR value equals 1, it indicates no impact of the exposure factor on the outcome. The 95% CI indicates that we have a 95% confidence that the true value of the OR value falls within this interval. When the width of CI is narrow, it indicates that the estimation of OR value is more accurate. If the *p* value is lower than 0.05, it indicates that this causal association has statistical significance. CI, confidence interval; *DNAJC19*, DnaJ heat shock protein family (Hsp40) member C19; *ETHE1*, ETHE1 persulfide dioxygenase; *GLRX2*, glutaredoxin 2; *HINT1*, histidine triad nucleotide binding protein 1; *HTATIP2*, HIV‐1 Tat interactive protein 2; *MICOS10*, mitochondrial contact site and cristae organizing system subunit 10; *MRM3*, mitochondrial rRNA methyltransferase 3; *MTHFS*, methenyltetrahydrofolate synthetase; *MTRF1L*, mitochondrial translation release factor 1 like; *NDUFB4*, NADH ubiquinone oxidoreductase subunit B4; *NUDT9*, nudix hydrolase 9; OR, odds ratio; *RMDN1*, regulator of microtubule dynamics 1; *SIRT5*, sirtuin 5; *SPATA20*, spermatogenesis associated 20; *UQCRB*, Ubiquinol‐cytochrome c reductase binding protein.

TwoSampleMR analysis revealed that 15 genes (*DNAJC19*, *ETHE1*, *HTATIP2*, *HINT1*, *GLRX2*, *MTRF1L*, *MICOS10*, *MTHFS*, *MRM3*, *NUDT9*, *NDUFB4*, *RMDN1*, *SIRT5*, and *SPATA20*, *UQCRB*) were causally associated with CRC (Figure 3). The sensitivity analysis results for heterogeneity and horizontal pleiotropy showed that the MR analysis results were robust (Table S7).

### 2.4 Protein–Protein Interaction (PPI) Network and Enrichment Analysis

**FIGURE 4:**
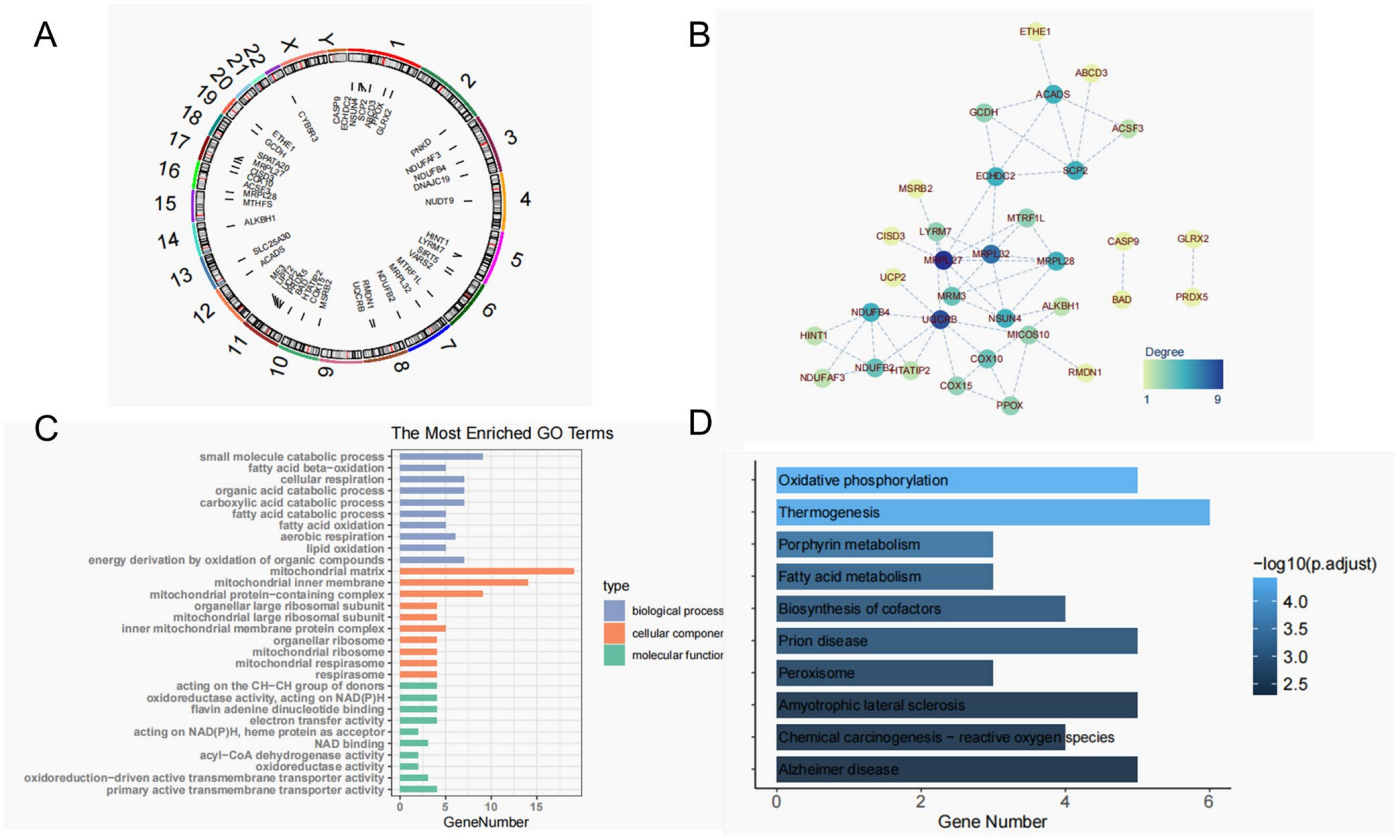
Results for PPI network and enrichment analysis of integrative genes. (A) Chromosomal localization of integrated genes. The position and length of each bar represent the position and length of the gene on the genome, respectively. (B) Integration of gene‐protein interaction networks. The depth of color indicates the degree size, and the darker the color, the greater the degree. (C) GO enrichment analysis of integrated genes. The horizontal axis represents the number of enriched genes, and the vertical axis represents the enriched entries. (D) KEGG enrichment analysis of the integrated genes. The horizontal axis represents the number of enriched genes, and the vertical axis represents the enriched entries.

After concatenating the eQTL, mQTL, and pQTL results, 44 MRGs (denoted as integrative genes) were causally associated with CRC. The chromosomal localization of the integrated genes is illustrated using a circular plot (Figure 4A). A PPI network was constructed based on the integrated genes, and 52 interactions corresponding to 33 genes were predicted (Figure 4B).

GO functional enrichment and KEGG signaling pathway analyses were performed on the 44 integrated genes. We selected the top 10 GO enrichment results and the top 10 KEGG pathway results based on *p*‐value ranking for display. GO analysis showed that 44 integrative genes were mainly located in the mitochondrial matrix and mitochondrial inner membrane, participating in small molecule catabolic processes and fatty acid beta‐oxidation processes, and acting on the CH‐CH group of donors (Figure 4C). The KEGG enrichment analysis results showed that the genes related to mitochondria in CRC were mainly enriched in the thermogenesis, OXPHOS, prion disease, and amyotrophic lateral sclerosis pathways (Figure 4D).

### 2.5 Mutation Identification and CNV of Integrative Genes

**FIGURE 5:**
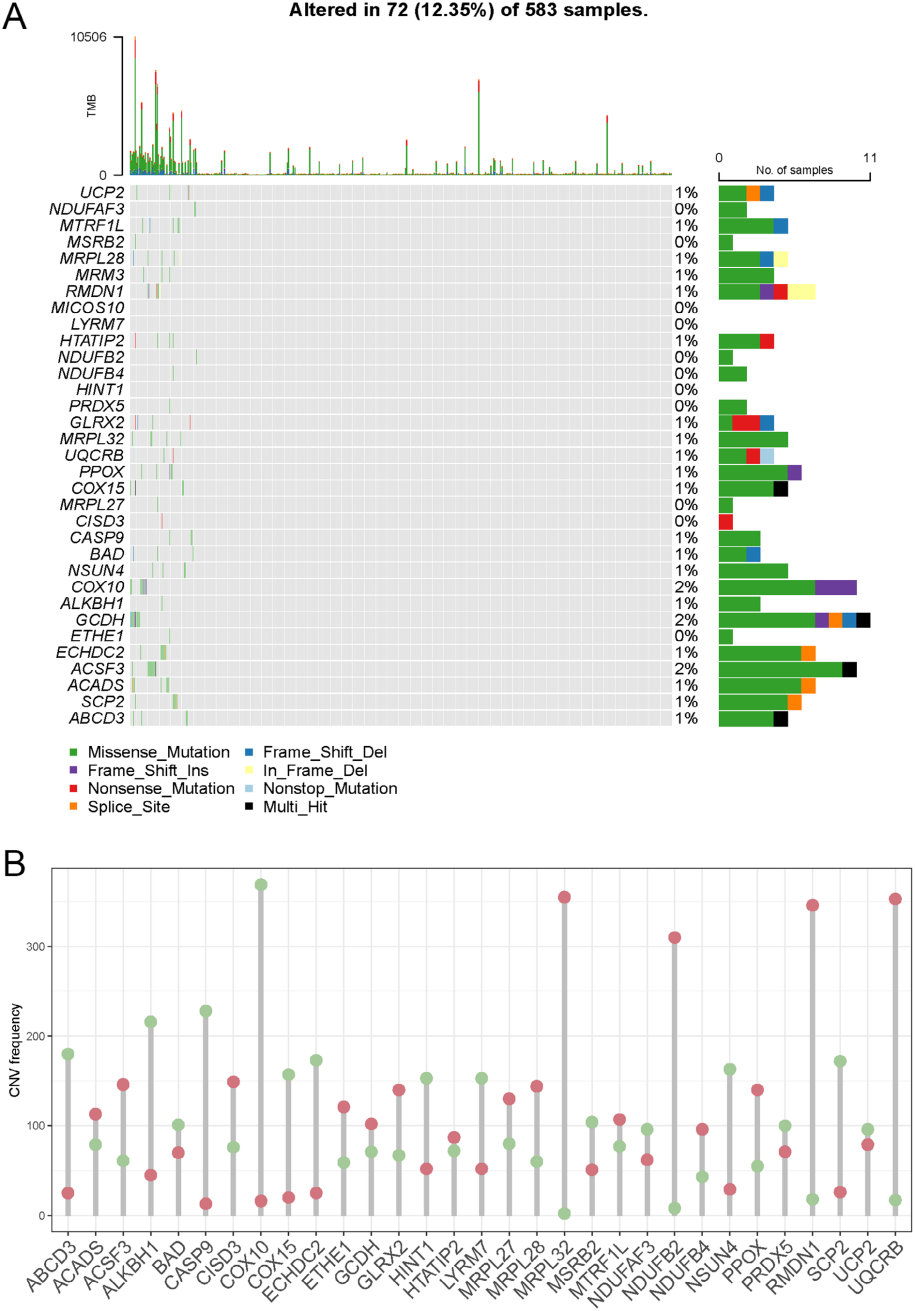
Results for mutation identification and CNV of integrative genes. (A) Estimation of mutations in integrating genes. (B) Evaluation of CNV frequency in integrating genes. The red dots indicate a higher frequency of CNV for the gene, while the green dots indicate a lower frequency.

Genetic level‐based analyses suggested that most of the somatic variants in the 33 genes obtained from the PPI network were missense mutations. Seventy‐two of the 583 CRC samples (12.35%) exhibited variations in mutation frequency within the MRGs by mutation analysis. The mutation frequencies of *COX10*, *GCDH*, and *ACSF3* were 2%, 2%, and 2%, respectively (Figure 5A). CNV analysis revealed amplification of CNVs in 16 genes and deletion of CNVs in 15 genes (Figure 5B). These findings showed the differences between MRGs mutations and CNVs, highlighting their potential effects on CRC carcinogenesis.

### 2.6 Expression Profiling and Survival Analysis of Integrative Genes

**FIGURE 6:**
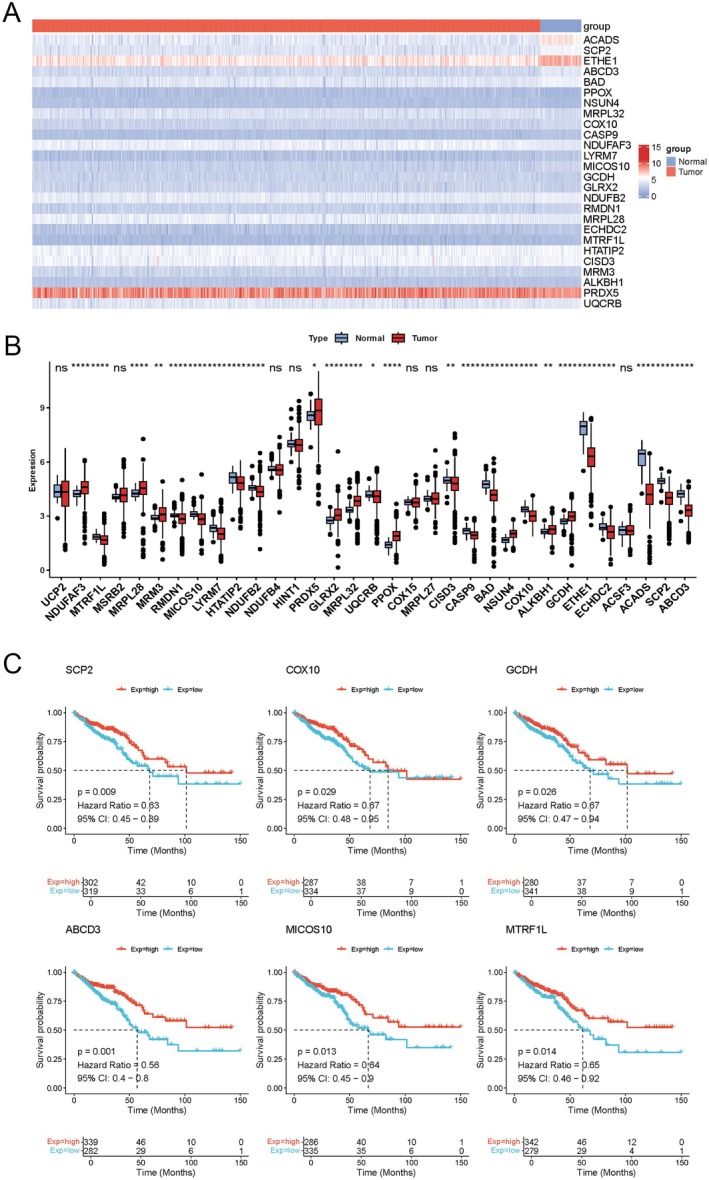
Results for expression profiling and survival analysis of integrative genes. (A) Heatmap of differentially integrated gene expression; (B) Differential analysis of integrated genes in normal and CRC tissues; (C) KM survival curves for integrated genes. *p < 0.05; **p < 0.01; ***p < 0.001; ****p < 0.0001.

We analyzed 44 MRGs that showed a causal relationship with CRC and studied their potential roles in CRC progression. Thirty‐three integrated gene expression data points from the above interactions were extracted, and the genes were compared between the disease and control groups using the Wilcoxon signed‐rank test. Twenty‐six MRGs were differentially expressed between tumors and controls (Figure 6A,B). Specifically, 15 genes were downregulated in CRC, while 11 genes were overexpressed.

Survival analyses of the differentially expressed integrative genes were performed, and KM survival curves were plotted. As a result, *SCP2* (HR = 0.63; 95% CI: 0.45–0.89; *p* = 0.009); *ABCD3* (HR = 0.85, 95% CI: 0.4–0.8; *p* = 0.001); *COX10* (HR = 0.67: 95% CI: 0.48–0.95; *p* = 0.029); *MICOS10* (HR = 0.64; 95% CI: 0.45–0.9: *p* = 0.013): *GCDH* (HR = 0.67: 95% CI: 0.47–0.94: *p* = 0.026); and *MTRF1L* (HR = 0.635; 95% CI: 0.46–0.92; *p* = 0.014) had prognostic significance (Figure 6C).

### 2.7 Immunological Analyses of Integrated Genes

**FIGURE 7:**
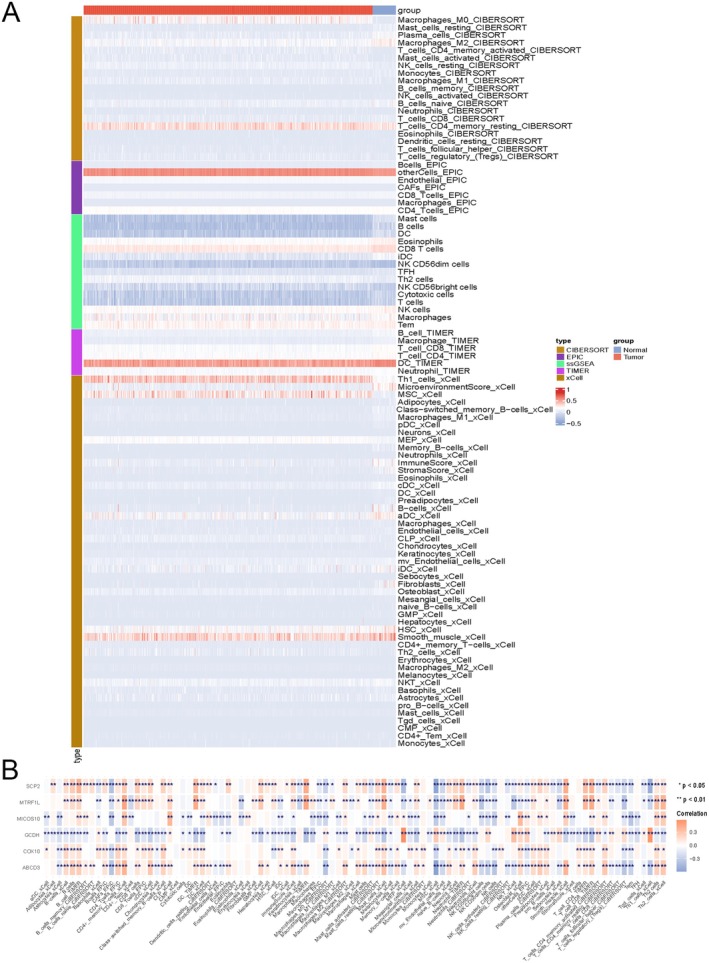
Results for immunological analyses of integrated genes. (A) Heat map of differential immune cells. (B) Heatmap of the correlation between differentially integrated MRGs and differentially expressed immune cells. *p < 0.05; **p < 0.01; ***p < 0.001; ****p < 0.0001.

Five different methods were used to calculate the proportion of immune cells and merge significantly different immune cells. We compared immune cell differences between the disease and control groups. The results showed that 96 immune cell types were significantly different between the disease and control groups (*p* < 0.05) (Figure 7A).

We conducted a correlation analysis between six prognostic MRGs and the differential immune cells mentioned above, as shown in Figure 7B. *ABCD3*, *MICOS10*, and *MTRF1L* significantly and positively correlated with CD4+ memory T cells. *SCP2*, *ABCD3*, and *MICOS10* were significantly negatively correlated with cancer‐associated fibroblasts (CAFs). *SCP2*, *ABCD3*, *MICOS10*, and *MTRF1L* were significantly negatively correlated with mesenchymal stem cells (MSC). *SCP2* positively correlated with macrophages, whereas *ABCD3* and *COX10* negatively correlated. In addition, *COX10* did not correlate with 96 types of immune cells.

### 2.8 Small Molecule Drug Forecast Analysis

**FIGURE 8:**
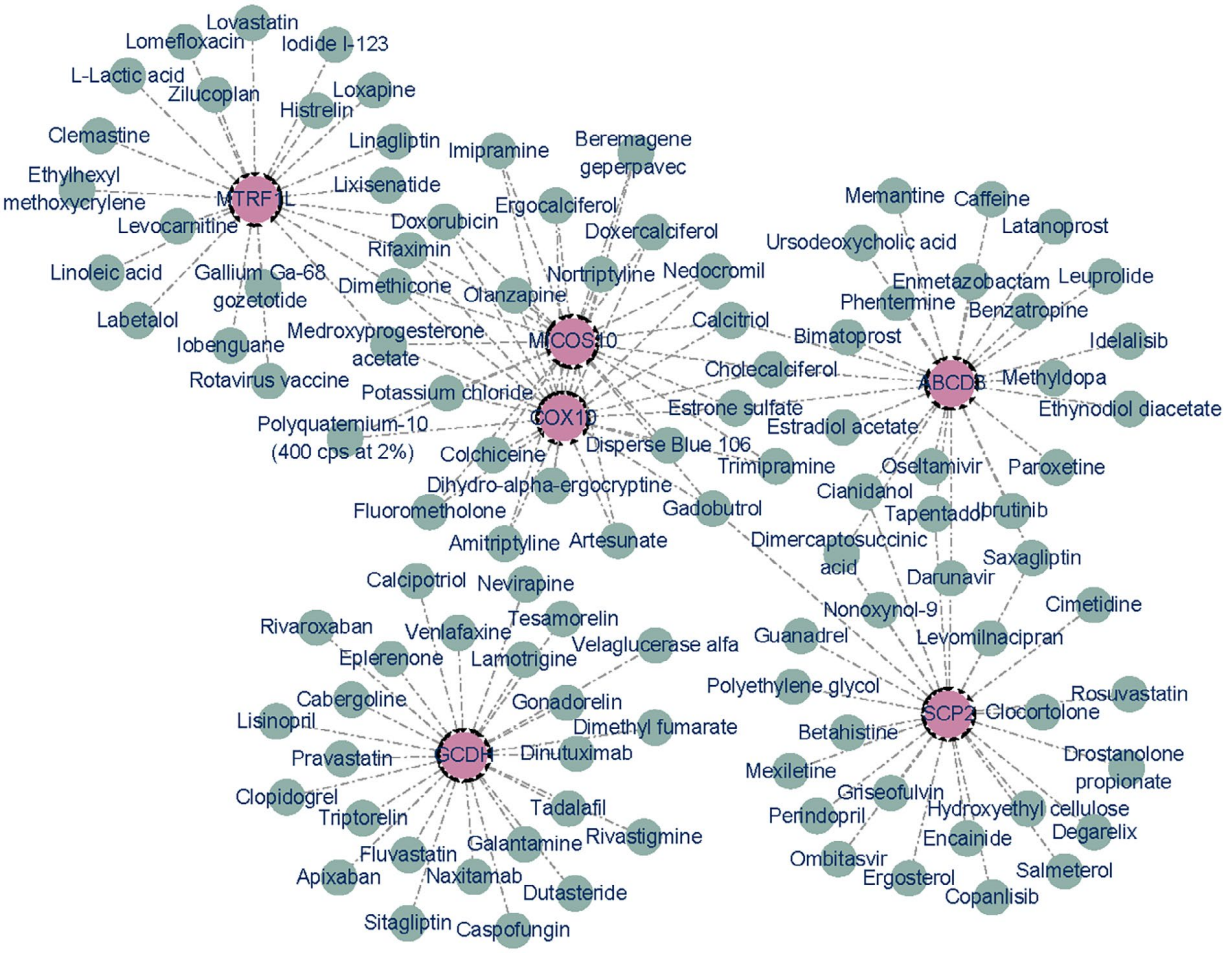
Results for small molecule drug prediction analyses of six prognostically significant genes.

We conducted drug prediction analysis on six key genes, which showed that there were 106 drugs targeting these six genes (Figure 8). Doxorubicin, Rifaximin, Dimethicone, and Medroxyprogesterone acetate simultaneously target *MTRF1L*, *COX10*, and *MICOS10*. Calcitriol, cholecystiferol, and estrone sulfate simultaneously target *ABCD3*, *COX10*, and *MICOS10*. Gadobutrol targets *ABCD3*, *COX10*, and *SCP2*.

## 3 Discussion

CRC is the third most prevalent cancer globally and the second most common cause of cancer‐related fatalities [2]. Due to the possibility of early metastasis in CRC, the survival rate of patients with CRC is usually less than 5 years [21]. Identifying genes that have a causal relationship with good prognosis in CRC is crucial for understanding and treating CRC. A causal association between 44 MRGs and CRC was identified using MR and SMR analyses in the study. MRGs that had a causal relationship with CRC were mainly located on the mitochondrial matrix and inner membrane and were involved in small molecule catabolism and fatty acid beta oxidation processes. They were mainly enriched in thermogenesis, OXPHOS, prion disease, and amyotrophic lateral sclerosis pathways by GO functional enrichment and KEGG signaling pathway analysis. Through PPI network, integrated gene multi omics, and survival analyses, we selected six genes (*SCP2*, *ABCD3*, *COX10*, *MICOS10*, *GCDH*, *MTRF1L*) with prognostic significance from the 44 integrated genes. Meanwhile, differences in the degree of immune cell infiltration were highly correlated with tumor progression and prognosis, and predicted drug‐target interactions for these six genes. These results enhance our understanding of the pathology of CRC and may uncover potential pharmacological targets.

The MRGs in CRC were mainly enriched in the thermogenesis and OXPHOS pathways. The OXPHOS system is the major supplier of ATP in malignant cells, and inhibiting OXPHOS levels can suppress CRC proliferation and metastasis [22, 23]. In addition, cancer subtypes upregulated by OXPHOS are treatable through targeted therapies with OXPHOS inhibitors [24, 25]. Previous studies have found that prohibitin 2 promoted CRC cell proliferation and tumorigenesis through OXPHOS [26]. Inhibition of OXPHOS enhances the sensitivity of patients with CRC to naphthylamide derivatives and mitoxantrone drugs [27]. Thus, OXPHOS is an emerging target in CRC therapy.

*SCP2* is a nonspecific lipid transport protein that binds to fatty acids, phospholipids, and other lipids [28]. It was significantly expressed in the mitochondria, lysosomes, and cytoplasmic lysates, which presumably reflect the extensive transport activity of this protein. Previous studies have explored the association between *SCP2* and cancers. It has been reported that *SCP2* expression was upregulated in gliomas, with its expression levels found to correlate with the grades of gliomas [29]. Bao et al. found that SCP2 was a prognostic gene for clear cell renal cell carcinoma [30]. Moreover, hepatocellular carcinoma cells overexpressing *SCP2* are more susceptible to cholesterol hydroperoxides (7α‐OOH) damage [31]. *SCP2* regulates angiogenesis and tumor migration, and the expression of *SCP2* in pancreatic carcinoma cells effectively inhibits angiogenesis and cell cycle progression [32]. In this study, we first discovered a causal relationship between the gene expression of *SCP2* and CRC, with better prognostic significance when its level is high. After PPI network analysis, *SCP2* demonstrated similar interactions with numerous miRNAs, indicating that the expression of crucial genes involved in mitochondrial metabolism in CRC is complexly regulated.

*ABCD3* is localized to the peroxisomal monolayer, and its expression increases with peroxisomal proliferation [33]. *ABCD3* is involved in fatty acid transport in the peroxisomes and may play a major role in tumorigenesis [34]. The roles of high and low levels of expression of *ABCD3* vary among different tumor cells. Downregulation of *ABCD3* is reportedly associated with better chemotherapy sensitivity and progression time in patients [35]. Li et al. found that ABCD3 serves as a prognostic biomarker for gliomas and exhibited a connection with immune cell infiltration [36]. Zhang et al. found that the mRNA expression of ABCD3 could be viewed as a potential diagnostic and prognostic biomarker in patients with CRC. In CRC patients, low ABCD3 mRNA expression was associated with poorer prognosis than high ABCD3 expression [20]. In this study, the gene expression of *ABCD3* had a causal relationship with CRC, and better prognostic significance existed when its levels were high, which was consistent with previous research results.

However, reports regarding the function of *COX10* in cancer are limited. *COX10* is an assembly factor for cytochrome c oxidase, and findings suggest that hypoxia has the potential to downregulate *COX10* expression, thereby exacerbating mitochondrial damage [37]. It is a terminal component of the mitochondrial respiratory chain and is involved in a variety of tumors [38]. Elevated levels of *COX10* are negatively correlated with the prognosis of patients with gliomas and meningiomas and may lead to aberrant phosphorylation [39, 40]. In lung cancer and melanoma, while a deficiency in *COX10* decreased tumor neovascularization and decelerated tumor growth, it also led to an expanded region of ischemic necrosis and facilitated tumor metastasis [41]. By sponging miR‐361‐5p, *COX10* acts as a competing endogenous RNA to positively regulate the expression of actin gamma 1, a pathogenic gene in CRC [42]. We demonstrated that *COX10* was causally associated with CRC and had better prognostic significance when its levels were high. A bioinformatics analysis of the Hub gene for irinotecan resistance in CRC showed that the expression of *COX10* was associated with irinotecan resistance [43]. The prognostic significance of *COX10* in CRC may be associated with irinotecan resistance, and further experiments are needed to verify this.

*GCDH* is a mitochondrial enzyme that participates in the breakdown of tryptophan, lysine, and hydroxylysine [44]; its depletion promotes the growth and metastasis of hepatocellular carcinoma, while its overexpression reverses these processes [45]. Verma et al. found that a reduction in *GCDH* expression was associated with an increase in the survival of patients with melanoma, identifying an addiction of melanoma cells to *GCDH*, which restricts apoptotic signaling through the control of NRF2 glut arylation [46]. In this study, *GCDH* was found to be causally associated with CRC, and better prognostic significance was observed when its levels were high.

The mitochondrial contact site and cristae organization system (MICOS) is a conserved multiunit complex that is essential for maintaining the characteristic mitochondrial structure [47]. *MICOS10*, as one of the core subunits of the MICOS complex, associates with other peripheral proteins and complexes to form the mature MICOS, directly shaping membrane characteristics and playing a crucial role in regulating mitochondrial cristae structure and function [47, 48]. Currently, studies have shown that changes in MICOS subunits are associated with various cancers, but so far there are few reports specifically linking MICOS10 to cancer. Elevated or altered *MICOS60* levels have been associated with various cancers including liver, prostate, colon, pancreatic, and breast, as well as with gastric tumor progression and poor survival outcomes [49, 50, 51, 52]. *MICOS19* upregulation is associated with breast and bladder cancers [53, 54]. In this study, *MICOS10* was found to be causally associated with CRC as a mitochondrial gene and had a better prognostic significance when it was highly expressed. MICOS also appears to play roles in the effectiveness of cancer treatment, resistance, and relapse. Overexpression of *MICOS25* enhances cellular resistance to etoposide and doxorubicin, whereas *MICOS25* deficiency increases sensitivity to these anti‐cancer drugs [55]. Although the exact mechanisms by which MICOS‐associated alterations impact cancer initiation, progression, and resistance are not fully understood, MICOS does play roles in cancer‐related cellular pathways. Further research can be conducted using physiological‐related in vivo models to determine whether changes in *MICOS10* are a key driving factor for the development and progression of CRC.

*MTRF1L* is a classical mitochondrial release factor that played a role in regulating mitochondrial fission and cell progression [56, 57, 58]. *MTRF1L* has been reported to be associated with cancer. Wang et al. observed that *MTFR1* could classify tumors based on extracapsular spread status, with significantly worse overall survival in cancer patients with negative lymph nodes who had extracapsular spread‐positive tumors [58]. Li et al. consistently reported that *MTFR1*, which was overexpressed in lung adenocarcinoma and associated with unfavorable prognosis in cancer patients, could enhance the proliferation, invasion, migration, and glycolysis of lung adenocarcinoma cells [59]. In this study, we found for the first time that *MTFR1* was causally associated with CRC as a mitochondrial gene and had better prognostic significance when highly expressed. In the later stage, clinical samples or animal model experiments can be utilized to verify the expression level of the *MTRF1L* in tumor versus normal tissue. Additionally, cell experiments will be conducted to observe the impact of *MTRF1L* knockout or overexpression on cancer cell proliferation, migration, and invasion abilities, further elucidating its role in cancer. These findings will serve as a foundation for the application of the *MTRF1L* in cancer diagnosis and treatment.

Cancer and the tumor microenvironment are inseparable. *SCP2*, *ABCD3*, *MICOS10*, *GCDH*, and *MTRF1L* were associated with most immune cells, while *COX10* was not associated with any of the 96 immune cells. This discovery suggested that these genes may play important roles in the immune microenvironment of CRC, potentially influencing the occurrence, development, and treatment response of cancer by regulating the activity or quantity of immune cells. In particular, we noticed that *SCP2*, *ABCD3*, and *MICOS10* were significantly negatively correlated with CAFs. CAFs are an important component of the tumor microenvironment, closely related to tumor angiogenesis, invasion, metastasis, and drug resistance [60]. The negative correlation between these three genes and CAFs may indicated that their high expression can inhibit the activity or quantity of CAFs, thus having potential prognostic significance. The study of *SCP2*, *ABCD3*, *MICOS10*, *GCDH*, and *MTRF1L* genes' immune infiltration and cancer correlation in CRC offers a fresh perspective. Future research should delve into their functions and mechanisms in CRC to uncover new immunotherapy targets and strategies.

This study also predicted drug–target interactions for these six genes and identified 106 drugs targeting these genes, effectively narrowing down the search range for candidate drug molecules corresponding to drugs and making a certain contribution to the treatment of CRC. Doxorubicin, Rifaximin, Dimethicone, and Medroxyprogesterone acetate concurrently target *MTRF1L*, *COX10*, and *MICOS10*, suggesting their potential efficacy in addressing pathways or conditions associated with these genes. Similarly, Calcitriol, cholecystiferol, and estrone sulfate share *ABCD3*, *COX10*, and *MICOS10* as targets, indicating a possible therapeutic synergy for specific diseases. Gadobutrol uniquely targets *ABCD3*, *COX10*, and *SCP2*, highlighting its specificity in modulating these gene‐related pathways. This analysis underscores the complexity of drug‐gene interactions and presents opportunities for targeted therapeutic strategies by leveraging drugs with shared gene targets. Further research is warranted to explore the clinical implications of these findings.

An advantage of our study was that we explored the causal relationship between MRGs expression, methylation, and protein abundance levels and CRC through MR, colocalization, and sensitivity analyses. Moreover, evidence from multiple omics levels was integrated, reinforcing the causal relationship between MRGs and CRC risk, and six prognosis‐related genes were identified. In addition, the statistical power of our study was increased by the GWASs with larger sample sizes. Finally, we solely incorporated samples of European descent, thereby diminishing the bias stemming from varied genetic backgrounds.

Furthermore, this study has some non‐negligible limitations. First, our results were analyzed based on public datasets; therefore, there may have been some unavoidable selection bias. Second, this study exclusively examined populations from Europe; therefore, extrapolation of our findings to populations from other regions should be performed with caution. In addition, no sex‐related differences were observed in the present study. In terms of our results, there is a need to consider whether there are differences when the data are applied only to the male or only to the female population. Finally, among the six genes we identified, *MICOS10* and *MTRF1L* were less likely to be associated with cancer; more experiments are needed to verify their relevance. In future, we will continue to explore the potential of these six genes as prognostic targets for CRC. Specifically, we plan to conduct gene knockout or overexpression experiments to investigate their impact on CRC cell proliferation, migration, and apoptosis. Additionally, we will utilize clinical samples to validate the correlation between gene expression and CRC prognosis, as well as to assess the sensitivity and specificity of these genes as prognostic biomarkers. In this study, based on 1136 MRGs, by MR and SMR analyses, colocalization analyses, susceptibility validation, PPI networks, integrative genes multi‐omics analysis, and survival analyses, six genes (*SCP2*, *ABCD3*, *COX10*, *MICOS10*, *GCDH*, and *MTRF1L*) were found to be causally associated with CRC, and their high expression levels had a better prognostic significance. These results indicated that these six genes may be potential targets for treating CRC, laying a theoretical foundation for future research on MRGs in CRC. This research may offer new insights for forecasting the clinical outcomes for CRC patients with CRC.

## 4 Materials and Methods

### 4.1 Study Design

**FIGURE 9:**
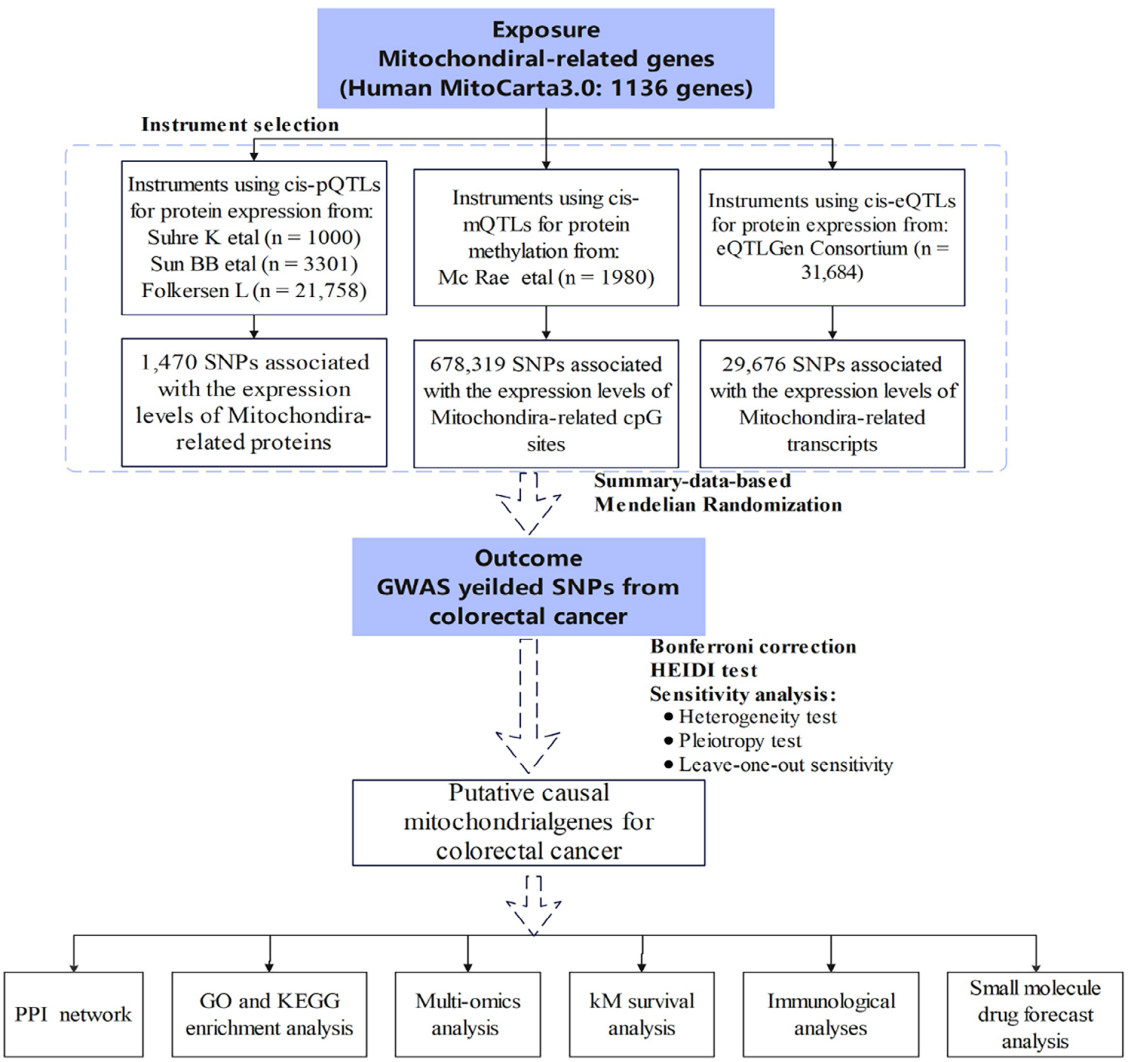
Flowchart of the analyses performed.

Figure 9 shows the study's overall design, detailing the procedure for selection of genetic variants and analytical methods.

### 4.2 Data Acquisition and Screening

Transcriptome data: We obtained transcriptome data of CRC from the publicly available The Cancer Genome Atlas (TCGA) [61], including colon adenocarcinoma (TCGA‐COAD) and rectal adenocarcinoma (TCGA‐READ) data, with 51 control and 638 tumor samples. Samples without complete survival time and survival status were excluded, and 51 control samples and 621 tumor samples were ultimately included for subsequent analysis.

MRGs: We obtained 1136 known MRGs from the MitoCarta3.0 database [13].

Expression quantitative trait loci (eQTL): Genetic variation within a 1000 kb range flanking the coding sequence (in cis) closely linked to gene expression was derived from eQTL aggregation statistics obtained from the eQTLGen consortium [62]. Single nucleotide polymorphisms (SNPs) linked to the expression of transcripts related to MRGs were selected from the cis‐eQTLs.

Methylation quantitative trait loci (mQTL): Cis‐mQTL for genetic variations closely related to the selected genes were derived using summary data from the Brisbane Systematic genetics study (*n* = 614) and the Lothian Birth Cohorts of 1921 and 1936 (*n* = 1366) [62, 63, 64].

Protein quantitative trait loci (pQTL): Cis‐pQTL for genetic variations linked to the expression of mitochondria‐related proteins were selected from five proteomic datasets, and SNPs strongly linked to mitochondrial protein expression were extracted.

CRC‐related SNPs were downloaded from the IEU OPEN GWAS. The 12 European population ID information included in this study is listed in Table 1.

**TABLE 1:** The 12 European population ID information corresponding to SNPs related to CRC.

GWAS ID	Year	Trait	Consortium	Sample size	Number of SNPs
ebi‐a‐GCST012876	2018	Colorectal cancer	NA	26,554	38,370,461
ebi‐a‐GCST012877	2018	Colorectal cancer	NA	23,691	12,313,483
ebi‐a‐GCST012878	2018	Colorectal cancer	NA	2234	11,517,359
ebi‐a‐GCST012879	2018	Colorectal cancer	NA	32,072	38,356,021
ebi‐a‐GCST012880	2018	Colorectal cancer	NA	8554	12,424,584
ebi‐a‐GCST90013862	2021	Colorectal cancer (Firth correction)	NA	407,746	11,039,202
ebi‐a‐GCST90013866	2021	Colorectal cancer (SPA correction)	NA	407,746	11,037,981
ebi‐a‐GCST90018588	2021	Colorectal cancer	NA	167,691	12,453,143
ebi‐a‐GCST90018808	2021	Colorectal cancer	NA	470,002	24,182,361
finn‐b‐C3_COLORECTAL	2021	Colorectal cancer	NA	—	16,380,466
finn‐b‐C3_COLORECTAL_EXALLC	2021	Colorectal cancer (all cancers excluded)	NA	—	16,380,321
ieu‐b‐4965	2021	Colorectal cancer	UK Biobank	377,673	11,738,639

#### 4.2.1 Instrumental Variable Selection

Quality control conditions for instrumental variables were: (1) All SNPs included in the initial analysis had at least suggestive *p* < 5 × 10 ^−8^; (2) SNPs with *R* ^2^ > 0.9 and *R* ^2^ < 0.05 around the top SNPs were removed, and only SNPs with an *R* ^2^ ≤ 0.9 in the remaining pairwise were retained; (3) The 1000 Gene Project European population for LD was removed from GWAS data (https://ctg.cncr.nl/software/magma); (4) Instrumental variables with *F* > 10 were calculated and retained; (5) Instrumental variables for exposure and outcome factors were unified using harmonize_data.

#### 4.2.2 MR Analysis and Summary‐Data‐Based MR (SMR) Analysis

We performed MR and SMR analysis with cis‐eQTL/cis‐mQTL/cis‐pQTL of MRGs as exposure factors and CRD as outcome factors. The SMR approach was devised to investigate pleiotropic links between genetic characteristics, including gene expression, DNA methylation, protein abundance, and significant complex traits such as disease phenotype. SMR is mainly based on Linux SMR (version 1.3.1) [65] The R package TwoSampleMR (version 0.5.10) was used for the MR analysis [66]. We used five methods for MR analysis, consisting of MR‐Egger, weighted median, inverse variance weighted (IVW), simple, and weighted models. Odds ratios (ORs) with 95% confidence intervals (CIs) were used to estimate the effect size. Our MR findings relied primarily on the results obtained using the IVW algorithm, with additional support provided by other methodologies [67].

#### 4.2.3 Sensitivity Analyses

Heterogeneity was assessed by Cochran's Q test (MR heterogeneity) [68]. For exposure factors with heterogeneity (*p* < 0.05), we used a model with random effects to test causality. Fixed effects were used to test the causality of exposure factors without heterogeneity (*p* > 0.05). The MR‐Egger regression intercept and its 95% CI were used to investigate the random estimation bias caused by directional pleiotropy.

#### 4.2.4 Protein–Protein Interaction (PPI) Network and Enrichment Analysis

MRGs with causal relationships in the MR were integrated, followed by chromosome mapping. We used the STRING database [69] to predict protein interactions of integrated MRGs under default threshold conditions (interaction score of 0.4) and constructed a network using Cytoscape (version 3.8.2) [70].

We used the R language package clusterProfiler (version 4.10.0) to execute Gene Ontology (GO) (including biological process [BP], cellular component [CC], and molecular function [MF]) and Kyoto Encyclopedia of Genes and Genomes (KEGG) pathway enrichment analysis with a general *p* < 0.05 for threshold screening [71].

#### 4.2.5 Multi Omics Analysis of Integrated Genes

Mutation frequencies of integrated genes were counted using Maltols (version 2.18.0) [72], and waterfall *p*lots were drawn for presentation. The copy number variation (CNV) data of TCGA‐COAD/TCGA‐READ were obtained from UCSC Xena, and ggplot2 (version 3.5.0) [73] was used to demonstrate frequencies of copy number amplification, normal diploid, and deletion with lollipop plots.

We extracted gene expression data and compared CRC vs. normal groups using the rank sum test. Differences (*p* < 0.05) were visualized with ggpubr boxplots (version 0.6.0) [74] and ComplexHeatmap heatmaps (version 2.18.0) [75].

#### 4.2.6 Kaplan–Meier (KM) Survival Analysis of Integrated Genes

The expression data of the integrated genes were extracted from the transcriptome data and merged with survival data. We used Survminer (version 0.4.9) [76] to determine the optimal threshold, dividing samples into high and low expression groups, and subsequently conducted survival analyses with survival (version 3.5–7) [77] to plot KM survival curves.

#### 4.2.7 Immunological Analyses of Integrated Genes

This study evaluated CRC and control immune microenvironments using IOBR (version 0.99.8) [78] with CIBERSORT, ssGSEA, xCell, TIMER, and EPIC. The Wilcoxon test compared groups, and ComplexHeatmap (version 2.18.0) [79] displayed results as a heatmap.

#### 4.2.8 Small Molecule Drug Forecast Analysis

Information on key gene‐drug relationships was obtained from the DrugBank database (https://go.drugbank.com/) and the networks were mapped for visualization.

## Author Contributions

**Limin Zhu:** conceptualization, methodology, software, data curation, formal analysis, writing – original draft, investigation, validation. **Xiaowei Huang:** data curation, software, writing – original draft. **Fan Zhang:** data curation, writing – original draft, visualization. **Jinzu Yang:** writing – review and editing, funding acquisition, supervision, project administration. **Zhenye Xu:** supervision, writing – review and editing, validation, resources, project administration.

## Conflicts of Interest

The authors declare no conflicts of interest.

## Supporting information


**Table S1.** MR analysis used TwoSampleMR package on the causal effect of mitochondria‐related genes eQTL on CRC outcomes.
**Table S2.** SMR and colocalization results of the association between mitochondria‐related genes eQTL and CRC outcomes.
**Table S3.** Sensitivity analysis used TwoSampleMR package on the association between mitochondria‐related genes eQTL and CRC outcomes.
**Table S4.** MR analysis used TwoSampleMR package on the causal effect of mitochondria‐related genes mQTL on CRC outcomes.
**Table S5.** SMR and colocalization results of the association between mitochondria‐related genes mQTL and CRC outcomes.
**Table S6.** Sensitivity analysis used TwoSampleMR package on the association between mitochondria‐related genes mQTL and CRC outcomes.
**Table S7.** Sensitivity analysis used TwoSampleMR package on the association between mitochondria‐related genes pQTL and CRC outcomes.

## Data Availability

All the data and material supporting the conclusions of this study are available from the corresponding author upon request.
