# Supplementary material for: Mendelian Randomization Analysis of Mitochondria‐Related Genes and Screening of Prognostic Genes in Colorectal Cancer
Source: Cancer Med. 2025 Jul 7;14(13):e71012. doi: 10.1002/cam4.71012 (PMC12230350; doi:10.1002/cam4.71012)
Supplement: Supplementary file 1 — Table S1. MR analysis used TwoSampleMR package on the causal effect of mitochondria‐related genes eQTL on CRC outcomes. Table S2. SMR and colocalization results of the association between mitochondria‐related genes eQTL and CRC outcomes. Table S3. Sensitivity analysis used TwoSampleMR package on the association between mitochondria‐related genes eQTL and CRC outcomes. Table S4. MR analysis used TwoSampleMR package on the causal effect of mitochondria‐related genes mQTL on CRC outcomes. Table S5. SMR and colocalization results of the association between mitochondria‐related genes mQTL and CRC outcomes. Table S6. Sensitivity analysis used TwoSampleMR package on the association between mitochondria‐related genes mQTL and CRC outcomes. Table S7. Sensitivity analysis used TwoSampleMR package on the association between mitochondria‐related genes pQTL and CRC outcomes. [file CAM4-14-e71012-s001.docx]

**Supplementary material**

**Contents**

**Supplementary tables**

**Supplementary Table 1.** MR analysis used TwoSampleMR package on the causal effect of mitochondria-related genes eQTL on CRC outcomes.

**Supplementary Table 2.** SMR and colocalization results of the association between mitochondria-related genes eQTL and CRC outcomes.

**Supplementary Table 3.** Sensitivity analysis used TwoSampleMR package on the association between mitochondria-related genes eQTL and CRC outcomes.

**Supplementary Table 4.** MR analysis used TwoSampleMR package on the causal effect of mitochondria-related genes mQTL on CRC outcomes.

**Supplementary Table 5.** SMR and colocalization results of the association between mitochondria-related genes mQTL and CRC outcomes.

**Supplementary Table 6.** Sensitivity analysis used TwoSampleMR package on the association between mitochondria-related genes mQTL and CRC outcomes.

**Supplementary Table 7.** Sensitivity analysis used TwoSampleMR package on the association between mitochondria-related genes pQTL and CRC outcomes.

**Supplementary Table 1.** MR analysis used TwoSampleMR package on the causal effect of mitochondria-related genes eQTL on CRC outcomes.

| **gene** | **id.exposure** | **outcome** | **method** | **nsnp** | **pval** | **OR (95% CI)** |
| --- | --- | --- | --- | --- | --- | --- |
| NDUFB2 | eqtl-a-ENSG00000090266 | Colorectal cancer || id:ieu-b-4965 | Inverse variance weighted (fixed effects) | 2 | 0.111840422 | 1.001(1-1.002) |
| ALKBH1 | eqtl-a-ENSG00000100601 | Colorectal cancer || id:ieu-b-4965 | Inverse variance weighted (fixed effects) | 3 | 0.06549258 | 0.998(0.997-1) |
| ALKBH1 | eqtl-a-ENSG00000100601 | Colorectal cancer || id:ieu-b-4965 | Weighted mode | 3 | 0.187854442 | 0.998(0.996-1) |
| ALKBH1 | eqtl-a-ENSG00000100601 | Colorectal cancer || id:ieu-b-4965 | MR Egger | 3 | 0.299302397 | 0.997(0.995-1) |
| ALKBH1 | eqtl-a-ENSG00000100601 | Colorectal cancer || id:ieu-b-4965 | Weighted median | 3 | 0.046995308 | 0.998(0.996-1) |
| ALKBH1 | eqtl-a-ENSG00000100601 | Colorectal cancer || id:ieu-b-4965 | Simple mode | 3 | 0.419337277 | 0.998(0.995-1.002) |
| GCDH | eqtl-a-ENSG00000105607 | Colorectal cancer || id:ieu-b-4965 | Simple mode | 4 | 0.949024938 | 1(0.998-1.002) |
| GCDH | eqtl-a-ENSG00000105607 | Colorectal cancer || id:ieu-b-4965 | Inverse variance weighted (fixed effects) | 4 | 0.20207643 | 1.001(1-1.002) |
| GCDH | eqtl-a-ENSG00000105607 | Colorectal cancer || id:ieu-b-4965 | MR Egger | 4 | 0.765882498 | 1(0.998-1.003) |
| GCDH | eqtl-a-ENSG00000105607 | Colorectal cancer || id:ieu-b-4965 | Weighted median | 4 | 0.212305247 | 1.001(1-1.002) |
| GCDH | eqtl-a-ENSG00000105607 | Colorectal cancer || id:ieu-b-4965 | Weighted mode | 4 | 0.305757342 | 1.001(1-1.002) |
| MRPL27 | eqtl-a-ENSG00000108826 | Colorectal cancer || id:ieu-b-4965 | Inverse variance weighted (fixed effects) | 2 | 0.085710906 | 0.998(0.996-1) |
| PRDX5 | eqtl-a-ENSG00000126432 | Colorectal cancer || id:ieu-b-4965 | Weighted median | 9 | 0.602725019 | 1.001(0.998-1.003) |
| PRDX5 | eqtl-a-ENSG00000126432 | Colorectal cancer || id:ieu-b-4965 | Simple mode | 9 | 0.288411743 | 0.997(0.992-1.002) |
| PRDX5 | eqtl-a-ENSG00000126432 | Colorectal cancer || id:ieu-b-4965 | Inverse variance weighted (multiplicative random effects) | 9 | 0.571925527 | 1.001(0.998-1.003) |
| PRDX5 | eqtl-a-ENSG00000126432 | Colorectal cancer || id:ieu-b-4965 | MR Egger | 9 | 0.500369287 | 1.002(0.997-1.007) |
| PRDX5 | eqtl-a-ENSG00000126432 | Colorectal cancer || id:ieu-b-4965 | Weighted mode | 9 | 0.023110426 | 1.003(1.001-1.006) |
| UCP2 | eqtl-a-ENSG00000175567 | Colorectal cancer || id:ieu-b-4965 | Weighted mode | 7 | 0.179775804 | 0.999(0.998-1) |
| UCP2 | eqtl-a-ENSG00000175567 | Colorectal cancer || id:ieu-b-4965 | MR Egger | 7 | 0.191361095 | 0.997(0.993-1.001) |
| UCP2 | eqtl-a-ENSG00000175567 | Colorectal cancer || id:ieu-b-4965 | Inverse variance weighted (fixed effects) | 7 | 0.379355662 | 0.999(0.998-1.001) |
| UCP2 | eqtl-a-ENSG00000175567 | Colorectal cancer || id:ieu-b-4965 | Simple mode | 7 | 0.436387755 | 0.999(0.996-1.002) |
| UCP2 | eqtl-a-ENSG00000175567 | Colorectal cancer || id:ieu-b-4965 | Weighted median | 7 | 0.144921519 | 0.999(0.998-1) |
| NDUFAF3 | eqtl-a-ENSG00000178057 | Colorectal cancer || id:ieu-b-4965 | Inverse variance weighted (multiplicative random effects) | 2 | 0.847439598 | 0.999(0.994-1.005) |
| LYRM7 | eqtl-a-ENSG00000186687 | Colorectal cancer || id:ieu-b-4965 | Inverse variance weighted (fixed effects) | 2 | 0.03124457 | 0.997(0.994-1) |
| NDUFB2 | eqtl-a-ENSG00000090266 | Colorectal cancer (all cancers excluded) || id:finn-b-C3_COLORECTAL_EXALLC | Inverse variance weighted (multiplicative random effects) | 2 | 0.861219012 | 0.98(0.778-1.233) |
| ALKBH1 | eqtl-a-ENSG00000100601 | Colorectal cancer (all cancers excluded) || id:finn-b-C3_COLORECTAL_EXALLC | Weighted median | 3 | 0.099563793 | 0.872(0.742-1.026) |
| ALKBH1 | eqtl-a-ENSG00000100601 | Colorectal cancer (all cancers excluded) || id:finn-b-C3_COLORECTAL_EXALLC | Inverse variance weighted (fixed effects) | 3 | 0.071957429 | 0.863(0.734-1.013) |
| ALKBH1 | eqtl-a-ENSG00000100601 | Colorectal cancer (all cancers excluded) || id:finn-b-C3_COLORECTAL_EXALLC | Weighted mode | 3 | 0.275632407 | 0.885(0.754-1.04) |
| ALKBH1 | eqtl-a-ENSG00000100601 | Colorectal cancer (all cancers excluded) || id:finn-b-C3_COLORECTAL_EXALLC | MR Egger | 3 | 0.739361707 | 0.944(0.727-1.226) |
| ALKBH1 | eqtl-a-ENSG00000100601 | Colorectal cancer (all cancers excluded) || id:finn-b-C3_COLORECTAL_EXALLC | Simple mode | 3 | 0.284388366 | 0.789(0.573-1.087) |
| GCDH | eqtl-a-ENSG00000105607 | Colorectal cancer (all cancers excluded) || id:finn-b-C3_COLORECTAL_EXALLC | Inverse variance weighted (fixed effects) | 3 | 0.02270073 | 1.148(1.019-1.293) |
| GCDH | eqtl-a-ENSG00000105607 | Colorectal cancer (all cancers excluded) || id:finn-b-C3_COLORECTAL_EXALLC | Weighted median | 3 | 0.011688117 | 1.165(1.035-1.312) |
| GCDH | eqtl-a-ENSG00000105607 | Colorectal cancer (all cancers excluded) || id:finn-b-C3_COLORECTAL_EXALLC | MR Egger | 3 | 0.597236322 | 1.126(0.82-1.547) |
| GCDH | eqtl-a-ENSG00000105607 | Colorectal cancer (all cancers excluded) || id:finn-b-C3_COLORECTAL_EXALLC | Simple mode | 3 | 0.209544257 | 1.2(0.987-1.459) |
| GCDH | eqtl-a-ENSG00000105607 | Colorectal cancer (all cancers excluded) || id:finn-b-C3_COLORECTAL_EXALLC | Weighted mode | 3 | 0.13646875 | 1.167(1.03-1.322) |
| MRPL27 | eqtl-a-ENSG00000108826 | Colorectal cancer (all cancers excluded) || id:finn-b-C3_COLORECTAL_EXALLC | Inverse variance weighted (fixed effects) | 2 | 0.709870186 | 0.961(0.777-1.187) |
| PRDX5 | eqtl-a-ENSG00000126432 | Colorectal cancer (all cancers excluded) || id:finn-b-C3_COLORECTAL_EXALLC | Simple mode | 11 | 0.898590982 | 0.986(0.796-1.221) |
| PRDX5 | eqtl-a-ENSG00000126432 | Colorectal cancer (all cancers excluded) || id:finn-b-C3_COLORECTAL_EXALLC | Weighted median | 11 | 0.776897558 | 0.985(0.888-1.093) |
| PRDX5 | eqtl-a-ENSG00000126432 | Colorectal cancer (all cancers excluded) || id:finn-b-C3_COLORECTAL_EXALLC | Inverse variance weighted (fixed effects) | 11 | 0.561259909 | 0.974(0.89-1.065) |
| PRDX5 | eqtl-a-ENSG00000126432 | Colorectal cancer (all cancers excluded) || id:finn-b-C3_COLORECTAL_EXALLC | Weighted mode | 11 | 0.853524999 | 0.99(0.888-1.103) |
| PRDX5 | eqtl-a-ENSG00000126432 | Colorectal cancer (all cancers excluded) || id:finn-b-C3_COLORECTAL_EXALLC | MR Egger | 11 | 0.916542898 | 0.991(0.845-1.163) |
| PPOX | eqtl-a-ENSG00000143224 | Colorectal cancer (all cancers excluded) || id:finn-b-C3_COLORECTAL_EXALLC | Inverse variance weighted (fixed effects) | 2 | 0.512739007 | 1.066(0.88-1.291) |
| UCP2 | eqtl-a-ENSG00000175567 | Colorectal cancer (all cancers excluded) || id:finn-b-C3_COLORECTAL_EXALLC | Weighted median | 6 | 0.61438646 | 0.973(0.875-1.082) |
| UCP2 | eqtl-a-ENSG00000175567 | Colorectal cancer (all cancers excluded) || id:finn-b-C3_COLORECTAL_EXALLC | MR Egger | 6 | 0.690742174 | 0.93(0.669-1.294) |
| UCP2 | eqtl-a-ENSG00000175567 | Colorectal cancer (all cancers excluded) || id:finn-b-C3_COLORECTAL_EXALLC | Inverse variance weighted (fixed effects) | 6 | 0.698072265 | 0.98(0.886-1.084) |
| UCP2 | eqtl-a-ENSG00000175567 | Colorectal cancer (all cancers excluded) || id:finn-b-C3_COLORECTAL_EXALLC | Weighted mode | 6 | 0.573191152 | 0.969(0.874-1.074) |
| UCP2 | eqtl-a-ENSG00000175567 | Colorectal cancer (all cancers excluded) || id:finn-b-C3_COLORECTAL_EXALLC | Simple mode | 6 | 0.79670892 | 1.034(0.814-1.312) |
| NDUFAF3 | eqtl-a-ENSG00000178057 | Colorectal cancer (all cancers excluded) || id:finn-b-C3_COLORECTAL_EXALLC | Inverse variance weighted (multiplicative random effects) | 2 | 0.916118061 | 0.969(0.545-1.726) |
| LYRM7 | eqtl-a-ENSG00000186687 | Colorectal cancer (all cancers excluded) || id:finn-b-C3_COLORECTAL_EXALLC | Inverse variance weighted (fixed effects) | 2 | 0.009593756 | 1.387(1.083-1.776) |
| NDUFB2 | eqtl-a-ENSG00000090266 | Colorectal cancer || id:finn-b-C3_COLORECTAL | Inverse variance weighted (multiplicative random effects) | 2 | 0.826485509 | 0.974(0.772-1.229) |
| ALKBH1 | eqtl-a-ENSG00000100601 | Colorectal cancer || id:finn-b-C3_COLORECTAL | MR Egger | 3 | 0.740726555 | 0.945(0.729-1.224) |
| ALKBH1 | eqtl-a-ENSG00000100601 | Colorectal cancer || id:finn-b-C3_COLORECTAL | Weighted mode | 3 | 0.305979937 | 0.889(0.751-1.053) |
| ALKBH1 | eqtl-a-ENSG00000100601 | Colorectal cancer || id:finn-b-C3_COLORECTAL | Weighted median | 3 | 0.140285464 | 0.885(0.752-1.041) |
| ALKBH1 | eqtl-a-ENSG00000100601 | Colorectal cancer || id:finn-b-C3_COLORECTAL | Inverse variance weighted (fixed effects) | 3 | 0.068636315 | 0.862(0.735-1.011) |
| ALKBH1 | eqtl-a-ENSG00000100601 | Colorectal cancer || id:finn-b-C3_COLORECTAL | Simple mode | 3 | 0.510424089 | 0.871(0.619-1.225) |
| GCDH | eqtl-a-ENSG00000105607 | Colorectal cancer || id:finn-b-C3_COLORECTAL | MR Egger | 3 | 0.510980767 | 1.13(0.881-1.45) |
| GCDH | eqtl-a-ENSG00000105607 | Colorectal cancer || id:finn-b-C3_COLORECTAL | Weighted median | 3 | 0.019283626 | 1.153(1.023-1.298) |
| GCDH | eqtl-a-ENSG00000105607 | Colorectal cancer || id:finn-b-C3_COLORECTAL | Inverse variance weighted (fixed effects) | 3 | 0.031143267 | 1.138(1.012-1.279) |
| GCDH | eqtl-a-ENSG00000105607 | Colorectal cancer || id:finn-b-C3_COLORECTAL | Weighted mode | 3 | 0.143645096 | 1.154(1.024-1.302) |
| GCDH | eqtl-a-ENSG00000105607 | Colorectal cancer || id:finn-b-C3_COLORECTAL | Simple mode | 3 | 0.273289448 | 1.17(0.953-1.437) |
| MRPL27 | eqtl-a-ENSG00000108826 | Colorectal cancer || id:finn-b-C3_COLORECTAL | Inverse variance weighted (fixed effects) | 2 | 0.7525778 | 0.967(0.784-1.192) |
| PRDX5 | eqtl-a-ENSG00000126432 | Colorectal cancer || id:finn-b-C3_COLORECTAL | MR Egger | 11 | 0.939715374 | 0.994(0.846-1.168) |
| PRDX5 | eqtl-a-ENSG00000126432 | Colorectal cancer || id:finn-b-C3_COLORECTAL | Weighted median | 11 | 0.814311444 | 0.987(0.889-1.097) |
| PRDX5 | eqtl-a-ENSG00000126432 | Colorectal cancer || id:finn-b-C3_COLORECTAL | Inverse variance weighted (fixed effects) | 11 | 0.655051849 | 0.98(0.897-1.071) |
| PRDX5 | eqtl-a-ENSG00000126432 | Colorectal cancer || id:finn-b-C3_COLORECTAL | Simple mode | 11 | 0.700328058 | 0.958(0.777-1.182) |
| PRDX5 | eqtl-a-ENSG00000126432 | Colorectal cancer || id:finn-b-C3_COLORECTAL | Weighted mode | 11 | 0.9255199 | 0.995(0.891-1.11) |
| PPOX | eqtl-a-ENSG00000143224 | Colorectal cancer || id:finn-b-C3_COLORECTAL | Inverse variance weighted (fixed effects) | 2 | 0.433727205 | 1.079(0.893-1.303) |
| UCP2 | eqtl-a-ENSG00000175567 | Colorectal cancer || id:finn-b-C3_COLORECTAL | MR Egger | 6 | 0.575158587 | 0.903(0.651-1.253) |
| UCP2 | eqtl-a-ENSG00000175567 | Colorectal cancer || id:finn-b-C3_COLORECTAL | Weighted median | 6 | 0.377742632 | 0.954(0.858-1.06) |
| UCP2 | eqtl-a-ENSG00000175567 | Colorectal cancer || id:finn-b-C3_COLORECTAL | Weighted mode | 6 | 0.435255733 | 0.954(0.855-1.064) |
| UCP2 | eqtl-a-ENSG00000175567 | Colorectal cancer || id:finn-b-C3_COLORECTAL | Simple mode | 6 | 0.97644573 | 0.997(0.802-1.239) |
| UCP2 | eqtl-a-ENSG00000175567 | Colorectal cancer || id:finn-b-C3_COLORECTAL | Inverse variance weighted (fixed effects) | 6 | 0.54511927 | 0.97(0.878-1.071) |
| NDUFAF3 | eqtl-a-ENSG00000178057 | Colorectal cancer || id:finn-b-C3_COLORECTAL | Inverse variance weighted (multiplicative random effects) | 2 | 0.816475641 | 0.941(0.563-1.573) |
| LYRM7 | eqtl-a-ENSG00000186687 | Colorectal cancer || id:finn-b-C3_COLORECTAL | Inverse variance weighted (fixed effects) | 2 | 0.010720956 | 1.375(1.077-1.755) |
| NDUFB2 | eqtl-a-ENSG00000090266 | Colorectal cancer || id:ebi-a-GCST90018808 | Inverse variance weighted (fixed effects) | 2 | 0.79698625 | 1.008(0.949-1.071) |
| ALKBH1 | eqtl-a-ENSG00000100601 | Colorectal cancer || id:ebi-a-GCST90018808 | MR Egger | 3 | 0.511666918 | 0.941(0.831-1.065) |
| ALKBH1 | eqtl-a-ENSG00000100601 | Colorectal cancer || id:ebi-a-GCST90018808 | Weighted mode | 3 | 0.165020174 | 0.913(0.841-0.992) |
| ALKBH1 | eqtl-a-ENSG00000100601 | Colorectal cancer || id:ebi-a-GCST90018808 | Weighted median | 3 | 0.02224392 | 0.911(0.841-0.987) |
| ALKBH1 | eqtl-a-ENSG00000100601 | Colorectal cancer || id:ebi-a-GCST90018808 | Inverse variance weighted (fixed effects) | 3 | 0.010176457 | 0.904(0.837-0.976) |
| ALKBH1 | eqtl-a-ENSG00000100601 | Colorectal cancer || id:ebi-a-GCST90018808 | Simple mode | 3 | 0.336814051 | 0.894(0.75-1.065) |
| GCDH | eqtl-a-ENSG00000105607 | Colorectal cancer || id:ebi-a-GCST90018808 | Weighted mode | 4 | 0.103383571 | 1.084(1.012-1.16) |
| GCDH | eqtl-a-ENSG00000105607 | Colorectal cancer || id:ebi-a-GCST90018808 | Inverse variance weighted (fixed effects) | 4 | 0.009790713 | 1.083(1.019-1.15) |
| GCDH | eqtl-a-ENSG00000105607 | Colorectal cancer || id:ebi-a-GCST90018808 | Weighted median | 4 | 0.013556487 | 1.085(1.017-1.157) |
| GCDH | eqtl-a-ENSG00000105607 | Colorectal cancer || id:ebi-a-GCST90018808 | MR Egger | 4 | 0.705144289 | 1.028(0.909-1.162) |
| GCDH | eqtl-a-ENSG00000105607 | Colorectal cancer || id:ebi-a-GCST90018808 | Simple mode | 4 | 0.505699763 | 0.947(0.821-1.092) |
| MRPL27 | eqtl-a-ENSG00000108826 | Colorectal cancer || id:ebi-a-GCST90018808 | Inverse variance weighted (fixed effects) | 2 | 0.119938322 | 0.915(0.817-1.023) |
| PRDX5 | eqtl-a-ENSG00000126432 | Colorectal cancer || id:ebi-a-GCST90018808 | MR Egger | 11 | 0.894151701 | 1.005(0.934-1.082) |
| PRDX5 | eqtl-a-ENSG00000126432 | Colorectal cancer || id:ebi-a-GCST90018808 | Weighted median | 11 | 0.687681138 | 0.99(0.945-1.038) |
| PRDX5 | eqtl-a-ENSG00000126432 | Colorectal cancer || id:ebi-a-GCST90018808 | Inverse variance weighted (fixed effects) | 11 | 0.964886558 | 0.999(0.959-1.041) |
| PRDX5 | eqtl-a-ENSG00000126432 | Colorectal cancer || id:ebi-a-GCST90018808 | Weighted mode | 11 | 0.88098537 | 0.996(0.95-1.045) |
| PRDX5 | eqtl-a-ENSG00000126432 | Colorectal cancer || id:ebi-a-GCST90018808 | Simple mode | 11 | 0.815228031 | 1.017(0.885-1.17) |
| PPOX | eqtl-a-ENSG00000143224 | Colorectal cancer || id:ebi-a-GCST90018808 | Inverse variance weighted (fixed effects) | 2 | 0.001499863 | 0.878(0.811-0.952) |
| UCP2 | eqtl-a-ENSG00000175567 | Colorectal cancer || id:ebi-a-GCST90018808 | Inverse variance weighted (fixed effects) | 7 | 0.349207754 | 0.976(0.928-1.027) |
| UCP2 | eqtl-a-ENSG00000175567 | Colorectal cancer || id:ebi-a-GCST90018808 | MR Egger | 7 | 0.98666112 | 0.997(0.729-1.365) |
| UCP2 | eqtl-a-ENSG00000175567 | Colorectal cancer || id:ebi-a-GCST90018808 | Weighted mode | 7 | 0.195355537 | 0.962(0.914-1.013) |
| UCP2 | eqtl-a-ENSG00000175567 | Colorectal cancer || id:ebi-a-GCST90018808 | Weighted median | 7 | 0.189371549 | 0.965(0.915-1.018) |
| UCP2 | eqtl-a-ENSG00000175567 | Colorectal cancer || id:ebi-a-GCST90018808 | Simple mode | 7 | 0.384880093 | 0.895(0.709-1.129) |
| NDUFAF3 | eqtl-a-ENSG00000178057 | Colorectal cancer || id:ebi-a-GCST90018808 | Weighted mode | 3 | 0.305108375 | 1.086(0.965-1.222) |
| NDUFAF3 | eqtl-a-ENSG00000178057 | Colorectal cancer || id:ebi-a-GCST90018808 | MR Egger | 3 | 0.822708663 | 0.956(0.702-1.301) |
| NDUFAF3 | eqtl-a-ENSG00000178057 | Colorectal cancer || id:ebi-a-GCST90018808 | Inverse variance weighted (fixed effects) | 3 | 0.051403277 | 1.111(0.999-1.236) |
| NDUFAF3 | eqtl-a-ENSG00000178057 | Colorectal cancer || id:ebi-a-GCST90018808 | Simple mode | 3 | 0.767640649 | 1.034(0.853-1.253) |
| NDUFAF3 | eqtl-a-ENSG00000178057 | Colorectal cancer || id:ebi-a-GCST90018808 | Weighted median | 3 | 0.08929093 | 1.098(0.986-1.223) |
| LYRM7 | eqtl-a-ENSG00000186687 | Colorectal cancer || id:ebi-a-GCST90018808 | Inverse variance weighted (fixed effects) | 2 | 0.179505288 | 0.913(0.799-1.043) |
| ALKBH1 | eqtl-a-ENSG00000100601 | Colorectal cancer || id:ebi-a-GCST90018588 | Weighted mode | 3 | 0.579257623 | 0.962(0.855-1.081) |
| ALKBH1 | eqtl-a-ENSG00000100601 | Colorectal cancer || id:ebi-a-GCST90018588 | Weighted median | 3 | 0.214860849 | 0.931(0.832-1.042) |
| ALKBH1 | eqtl-a-ENSG00000100601 | Colorectal cancer || id:ebi-a-GCST90018588 | Simple mode | 3 | 0.237605497 | 0.632(0.368-1.084) |
| ALKBH1 | eqtl-a-ENSG00000100601 | Colorectal cancer || id:ebi-a-GCST90018588 | MR Egger | 3 | 0.570803617 | 1.075(0.9-1.283) |
| ALKBH1 | eqtl-a-ENSG00000100601 | Colorectal cancer || id:ebi-a-GCST90018588 | Inverse variance weighted (fixed effects) | 3 | 0.183269202 | 0.928(0.831-1.036) |
| GCDH | eqtl-a-ENSG00000105607 | Colorectal cancer || id:ebi-a-GCST90018588 | Weighted mode | 3 | 0.480057038 | 1.05(0.94-1.173) |
| GCDH | eqtl-a-ENSG00000105607 | Colorectal cancer || id:ebi-a-GCST90018588 | Inverse variance weighted (fixed effects) | 3 | 0.347499008 | 1.05(0.948-1.164) |
| GCDH | eqtl-a-ENSG00000105607 | Colorectal cancer || id:ebi-a-GCST90018588 | Weighted median | 3 | 0.30910928 | 1.056(0.951-1.171) |
| GCDH | eqtl-a-ENSG00000105607 | Colorectal cancer || id:ebi-a-GCST90018588 | Simple mode | 3 | 0.352812869 | 1.11(0.936-1.317) |
| GCDH | eqtl-a-ENSG00000105607 | Colorectal cancer || id:ebi-a-GCST90018588 | MR Egger | 3 | 0.704325406 | 0.933(0.71-1.225) |
| MRPL27 | eqtl-a-ENSG00000108826 | Colorectal cancer || id:ebi-a-GCST90018588 | Inverse variance weighted (fixed effects) | 2 | 0.4515975 | 0.928(0.764-1.127) |
| PRDX5 | eqtl-a-ENSG00000126432 | Colorectal cancer || id:ebi-a-GCST90018588 | Weighted median | 7 | 0.98308886 | 1.001(0.941-1.064) |
| PRDX5 | eqtl-a-ENSG00000126432 | Colorectal cancer || id:ebi-a-GCST90018588 | Weighted mode | 7 | 0.996129264 | 1(0.941-1.063) |
| PRDX5 | eqtl-a-ENSG00000126432 | Colorectal cancer || id:ebi-a-GCST90018588 | Inverse variance weighted (fixed effects) | 7 | 0.813501339 | 0.993(0.94-1.05) |
| PRDX5 | eqtl-a-ENSG00000126432 | Colorectal cancer || id:ebi-a-GCST90018588 | MR Egger | 7 | 0.989766538 | 0.999(0.912-1.095) |
| PRDX5 | eqtl-a-ENSG00000126432 | Colorectal cancer || id:ebi-a-GCST90018588 | Simple mode | 7 | 0.833147319 | 0.985(0.865-1.123) |
| NDUFAF3 | eqtl-a-ENSG00000178057 | Colorectal cancer || id:ebi-a-GCST90018588 | Inverse variance weighted (fixed effects) | 2 | 0.033528791 | 1.173(1.013-1.359) |
| LYRM7 | eqtl-a-ENSG00000186687 | Colorectal cancer || id:ebi-a-GCST90018588 | Inverse variance weighted (fixed effects) | 2 | 0.756058434 | 0.966(0.777-1.201) |
| NDUFB2 | eqtl-a-ENSG00000090266 | Colorectal cancer (SPA correction) || id:ebi-a-GCST90013866 | Inverse variance weighted (fixed effects) | 2 | 0.012914304 | 1.131(1.026-1.245) |
| ALKBH1 | eqtl-a-ENSG00000100601 | Colorectal cancer (SPA correction) || id:ebi-a-GCST90013866 | Inverse variance weighted (multiplicative random effects) | 2 | 0.912037225 | 0.983(0.73-1.325) |
| GCDH | eqtl-a-ENSG00000105607 | Colorectal cancer (SPA correction) || id:ebi-a-GCST90013866 | Inverse variance weighted (fixed effects) | 3 | 0.501235125 | 0.926(0.742-1.157) |
| GCDH | eqtl-a-ENSG00000105607 | Colorectal cancer (SPA correction) || id:ebi-a-GCST90013866 | Simple mode | 3 | 0.868572664 | 1.034(0.73-1.463) |
| GCDH | eqtl-a-ENSG00000105607 | Colorectal cancer (SPA correction) || id:ebi-a-GCST90013866 | Weighted mode | 3 | 0.864125028 | 1.034(0.739-1.446) |
| GCDH | eqtl-a-ENSG00000105607 | Colorectal cancer (SPA correction) || id:ebi-a-GCST90013866 | MR Egger | 3 | 0.577433834 | 0.75(0.365-1.541) |
| GCDH | eqtl-a-ENSG00000105607 | Colorectal cancer (SPA correction) || id:ebi-a-GCST90013866 | Weighted median | 3 | 0.92125926 | 1.014(0.771-1.333) |
| MRPL27 | eqtl-a-ENSG00000108826 | Colorectal cancer (SPA correction) || id:ebi-a-GCST90013866 | Inverse variance weighted (fixed effects) | 2 | 0.068768823 | 0.854(0.72-1.012) |
| PRDX5 | eqtl-a-ENSG00000126432 | Colorectal cancer (SPA correction) || id:ebi-a-GCST90013866 | Weighted mode | 11 | 0.364677656 | 0.952(0.86-1.054) |
| PRDX5 | eqtl-a-ENSG00000126432 | Colorectal cancer (SPA correction) || id:ebi-a-GCST90013866 | Inverse variance weighted (multiplicative random effects) | 11 | 0.895320524 | 1.007(0.904-1.122) |
| PRDX5 | eqtl-a-ENSG00000126432 | Colorectal cancer (SPA correction) || id:ebi-a-GCST90013866 | MR Egger | 11 | 0.383389636 | 0.923(0.779-1.095) |
| PRDX5 | eqtl-a-ENSG00000126432 | Colorectal cancer (SPA correction) || id:ebi-a-GCST90013866 | Weighted median | 11 | 0.214104939 | 0.941(0.855-1.036) |
| PRDX5 | eqtl-a-ENSG00000126432 | Colorectal cancer (SPA correction) || id:ebi-a-GCST90013866 | Simple mode | 11 | 0.909630125 | 1.017(0.767-1.349) |
| UCP2 | eqtl-a-ENSG00000175567 | Colorectal cancer (SPA correction) || id:ebi-a-GCST90013866 | MR Egger | 6 | 0.8716428 | 1.026(0.763-1.381) |
| UCP2 | eqtl-a-ENSG00000175567 | Colorectal cancer (SPA correction) || id:ebi-a-GCST90013866 | Weighted mode | 6 | 0.867038683 | 0.991(0.897-1.095) |
| UCP2 | eqtl-a-ENSG00000175567 | Colorectal cancer (SPA correction) || id:ebi-a-GCST90013866 | Weighted median | 6 | 0.859832013 | 0.991(0.899-1.093) |
| UCP2 | eqtl-a-ENSG00000175567 | Colorectal cancer (SPA correction) || id:ebi-a-GCST90013866 | Inverse variance weighted (fixed effects) | 6 | 0.778161555 | 0.987(0.902-1.081) |
| UCP2 | eqtl-a-ENSG00000175567 | Colorectal cancer (SPA correction) || id:ebi-a-GCST90013866 | Simple mode | 6 | 0.83455019 | 0.981(0.825-1.166) |
| NDUFAF3 | eqtl-a-ENSG00000178057 | Colorectal cancer (SPA correction) || id:ebi-a-GCST90013866 | Inverse variance weighted (fixed effects) | 2 | 0.889289758 | 0.986(0.806-1.206) |
| LYRM7 | eqtl-a-ENSG00000186687 | Colorectal cancer (SPA correction) || id:ebi-a-GCST90013866 | Inverse variance weighted (fixed effects) | 2 | 0.112955109 | 0.843(0.683-1.041) |
| NDUFB2 | eqtl-a-ENSG00000090266 | Colorectal cancer (Firth correction) || id:ebi-a-GCST90013862 | Inverse variance weighted (fixed effects) | 2 | 0.013854705 | 1.132(1.026-1.25) |
| ALKBH1 | eqtl-a-ENSG00000100601 | Colorectal cancer (Firth correction) || id:ebi-a-GCST90013862 | Inverse variance weighted (multiplicative random effects) | 2 | 0.913080536 | 0.984(0.73-1.325) |
| GCDH | eqtl-a-ENSG00000105607 | Colorectal cancer (Firth correction) || id:ebi-a-GCST90013862 | Weighted mode | 3 | 0.85570733 | 1.034(0.754-1.418) |
| GCDH | eqtl-a-ENSG00000105607 | Colorectal cancer (Firth correction) || id:ebi-a-GCST90013862 | MR Egger | 3 | 0.577433834 | 0.75(0.365-1.541) |
| GCDH | eqtl-a-ENSG00000105607 | Colorectal cancer (Firth correction) || id:ebi-a-GCST90013862 | Simple mode | 3 | 0.875317411 | 1.034(0.717-1.491) |
| GCDH | eqtl-a-ENSG00000105607 | Colorectal cancer (Firth correction) || id:ebi-a-GCST90013862 | Inverse variance weighted (fixed effects) | 3 | 0.501235125 | 0.926(0.742-1.157) |
| GCDH | eqtl-a-ENSG00000105607 | Colorectal cancer (Firth correction) || id:ebi-a-GCST90013862 | Weighted median | 3 | 0.923062704 | 1.014(0.766-1.342) |
| MRPL27 | eqtl-a-ENSG00000108826 | Colorectal cancer (Firth correction) || id:ebi-a-GCST90013862 | Inverse variance weighted (fixed effects) | 2 | 0.068768823 | 0.854(0.72-1.012) |
| PRDX5 | eqtl-a-ENSG00000126432 | Colorectal cancer (Firth correction) || id:ebi-a-GCST90013862 | MR Egger | 11 | 0.386640857 | 0.925(0.781-1.095) |
| PRDX5 | eqtl-a-ENSG00000126432 | Colorectal cancer (Firth correction) || id:ebi-a-GCST90013862 | Weighted median | 11 | 0.213003181 | 0.941(0.855-1.036) |
| PRDX5 | eqtl-a-ENSG00000126432 | Colorectal cancer (Firth correction) || id:ebi-a-GCST90013862 | Inverse variance weighted (multiplicative random effects) | 11 | 0.874253484 | 1.009(0.906-1.123) |
| PRDX5 | eqtl-a-ENSG00000126432 | Colorectal cancer (Firth correction) || id:ebi-a-GCST90013862 | Weighted mode | 11 | 0.362601862 | 0.95(0.856-1.055) |
| PRDX5 | eqtl-a-ENSG00000126432 | Colorectal cancer (Firth correction) || id:ebi-a-GCST90013862 | Simple mode | 11 | 0.908236222 | 1.016(0.786-1.312) |
| UCP2 | eqtl-a-ENSG00000175567 | Colorectal cancer (Firth correction) || id:ebi-a-GCST90013862 | Simple mode | 6 | 0.83941122 | 0.981(0.821-1.172) |
| UCP2 | eqtl-a-ENSG00000175567 | Colorectal cancer (Firth correction) || id:ebi-a-GCST90013862 | MR Egger | 6 | 0.8716428 | 1.026(0.763-1.381) |
| UCP2 | eqtl-a-ENSG00000175567 | Colorectal cancer (Firth correction) || id:ebi-a-GCST90013862 | Weighted median | 6 | 0.859100028 | 0.991(0.9-1.092) |
| UCP2 | eqtl-a-ENSG00000175567 | Colorectal cancer (Firth correction) || id:ebi-a-GCST90013862 | Inverse variance weighted (fixed effects) | 6 | 0.778161555 | 0.987(0.902-1.081) |
| UCP2 | eqtl-a-ENSG00000175567 | Colorectal cancer (Firth correction) || id:ebi-a-GCST90013862 | Weighted mode | 6 | 0.874630175 | 0.991(0.892-1.102) |
| NDUFAF3 | eqtl-a-ENSG00000178057 | Colorectal cancer (Firth correction) || id:ebi-a-GCST90013862 | Inverse variance weighted (fixed effects) | 2 | 0.889289758 | 0.986(0.806-1.206) |
| LYRM7 | eqtl-a-ENSG00000186687 | Colorectal cancer (Firth correction) || id:ebi-a-GCST90013862 | Inverse variance weighted (fixed effects) | 2 | 0.112955109 | 0.843(0.683-1.041) |
| NDUFB2 | eqtl-a-ENSG00000090266 | Colorectal cancer || id:ebi-a-GCST012880 | Inverse variance weighted (multiplicative random effects) | 2 | 0.272680119 | 1.155(0.893-1.496) |
| ALKBH1 | eqtl-a-ENSG00000100601 | Colorectal cancer || id:ebi-a-GCST012880 | Weighted mode | 3 | 0.331471996 | 1.146(0.929-1.414) |
| ALKBH1 | eqtl-a-ENSG00000100601 | Colorectal cancer || id:ebi-a-GCST012880 | MR Egger | 3 | 0.544462523 | 1.145(0.844-1.554) |
| ALKBH1 | eqtl-a-ENSG00000100601 | Colorectal cancer || id:ebi-a-GCST012880 | Weighted median | 3 | 0.15973327 | 1.148(0.947-1.392) |
| ALKBH1 | eqtl-a-ENSG00000100601 | Colorectal cancer || id:ebi-a-GCST012880 | Inverse variance weighted (fixed effects) | 3 | 0.147782309 | 1.149(0.952-1.387) |
| ALKBH1 | eqtl-a-ENSG00000100601 | Colorectal cancer || id:ebi-a-GCST012880 | Simple mode | 3 | 0.522711416 | 1.131(0.826-1.548) |
| GCDH | eqtl-a-ENSG00000105607 | Colorectal cancer || id:ebi-a-GCST012880 | Simple mode | 3 | 0.618874133 | 0.93(0.729-1.187) |
| GCDH | eqtl-a-ENSG00000105607 | Colorectal cancer || id:ebi-a-GCST012880 | Weighted mode | 3 | 0.567013288 | 0.955(0.835-1.091) |
| GCDH | eqtl-a-ENSG00000105607 | Colorectal cancer || id:ebi-a-GCST012880 | MR Egger | 3 | 0.570355667 | 0.673(0.255-1.775) |
| GCDH | eqtl-a-ENSG00000105607 | Colorectal cancer || id:ebi-a-GCST012880 | Inverse variance weighted (fixed effects) | 3 | 0.66312105 | 0.97(0.848-1.111) |
| GCDH | eqtl-a-ENSG00000105607 | Colorectal cancer || id:ebi-a-GCST012880 | Weighted median | 3 | 0.528271278 | 0.956(0.833-1.098) |
| MRPL27 | eqtl-a-ENSG00000108826 | Colorectal cancer || id:ebi-a-GCST012880 | Inverse variance weighted (fixed effects) | 2 | 0.948200378 | 0.992(0.781-1.26) |
| PRDX5 | eqtl-a-ENSG00000126432 | Colorectal cancer || id:ebi-a-GCST012880 | MR Egger | 11 | 0.38592428 | 1.097(0.899-1.34) |
| PRDX5 | eqtl-a-ENSG00000126432 | Colorectal cancer || id:ebi-a-GCST012880 | Weighted median | 11 | 0.027430802 | 1.146(1.015-1.295) |
| PRDX5 | eqtl-a-ENSG00000126432 | Colorectal cancer || id:ebi-a-GCST012880 | Inverse variance weighted (fixed effects) | 11 | 0.025499673 | 1.125(1.015-1.247) |
| PRDX5 | eqtl-a-ENSG00000126432 | Colorectal cancer || id:ebi-a-GCST012880 | Weighted mode | 11 | 0.067608781 | 1.138(1.006-1.287) |
| PRDX5 | eqtl-a-ENSG00000126432 | Colorectal cancer || id:ebi-a-GCST012880 | Simple mode | 11 | 0.53219455 | 0.897(0.645-1.247) |
| PPOX | eqtl-a-ENSG00000143224 | Colorectal cancer || id:ebi-a-GCST012880 | Inverse variance weighted (fixed effects) | 2 | 0.583744471 | 0.941(0.759-1.168) |
| UCP2 | eqtl-a-ENSG00000175567 | Colorectal cancer || id:ebi-a-GCST012880 | MR Egger | 6 | 0.652893111 | 1.145(0.662-1.983) |
| UCP2 | eqtl-a-ENSG00000175567 | Colorectal cancer || id:ebi-a-GCST012880 | Inverse variance weighted (fixed effects) | 6 | 0.998406396 | 1(0.875-1.143) |
| UCP2 | eqtl-a-ENSG00000175567 | Colorectal cancer || id:ebi-a-GCST012880 | Weighted mode | 6 | 0.666361753 | 1.034(0.897-1.192) |
| UCP2 | eqtl-a-ENSG00000175567 | Colorectal cancer || id:ebi-a-GCST012880 | Weighted median | 6 | 0.718261918 | 1.027(0.89-1.184) |
| UCP2 | eqtl-a-ENSG00000175567 | Colorectal cancer || id:ebi-a-GCST012880 | Simple mode | 6 | 0.830001952 | 0.967(0.724-1.292) |
| NDUFAF3 | eqtl-a-ENSG00000178057 | Colorectal cancer || id:ebi-a-GCST012880 | Inverse variance weighted (multiplicative random effects) | 2 | 0.692438221 | 1.176(0.526-2.628) |
| LYRM7 | eqtl-a-ENSG00000186687 | Colorectal cancer || id:ebi-a-GCST012880 | Inverse variance weighted (fixed effects) | 2 | 0.181612428 | 1.211(0.914-1.604) |
| NDUFB2 | eqtl-a-ENSG00000090266 | Colorectal cancer || id:ebi-a-GCST012879 | Inverse variance weighted (fixed effects) | 2 | 0.145630681 | 1.054(0.982-1.131) |
| ALKBH1 | eqtl-a-ENSG00000100601 | Colorectal cancer || id:ebi-a-GCST012879 | MR Egger | 3 | 0.587522241 | 1.067(0.902-1.262) |
| ALKBH1 | eqtl-a-ENSG00000100601 | Colorectal cancer || id:ebi-a-GCST012879 | Inverse variance weighted (fixed effects) | 3 | 0.460151698 | 0.962(0.867-1.067) |
| ALKBH1 | eqtl-a-ENSG00000100601 | Colorectal cancer || id:ebi-a-GCST012879 | Weighted median | 3 | 0.521043183 | 0.966(0.868-1.074) |
| ALKBH1 | eqtl-a-ENSG00000100601 | Colorectal cancer || id:ebi-a-GCST012879 | Simple mode | 3 | 0.294056893 | 0.762(0.523-1.112) |
| ALKBH1 | eqtl-a-ENSG00000100601 | Colorectal cancer || id:ebi-a-GCST012879 | Weighted mode | 3 | 0.908341893 | 0.993(0.889-1.109) |
| GCDH | eqtl-a-ENSG00000105607 | Colorectal cancer || id:ebi-a-GCST012879 | Weighted median | 3 | 0.778301521 | 1.01(0.94-1.086) |
| GCDH | eqtl-a-ENSG00000105607 | Colorectal cancer || id:ebi-a-GCST012879 | Weighted mode | 3 | 0.776861225 | 1.013(0.935-1.098) |
| GCDH | eqtl-a-ENSG00000105607 | Colorectal cancer || id:ebi-a-GCST012879 | Simple mode | 3 | 0.989062179 | 1.001(0.891-1.125) |
| GCDH | eqtl-a-ENSG00000105607 | Colorectal cancer || id:ebi-a-GCST012879 | MR Egger | 3 | 0.69751677 | 1.128(0.712-1.787) |
| GCDH | eqtl-a-ENSG00000105607 | Colorectal cancer || id:ebi-a-GCST012879 | Inverse variance weighted (fixed effects) | 3 | 0.74003645 | 1.012(0.943-1.086) |
| MRPL27 | eqtl-a-ENSG00000108826 | Colorectal cancer || id:ebi-a-GCST012879 | Inverse variance weighted (fixed effects) | 2 | 0.697768789 | 0.975(0.856-1.11) |
| PRDX5 | eqtl-a-ENSG00000126432 | Colorectal cancer || id:ebi-a-GCST012879 | MR Egger | 11 | 0.171854116 | 0.933(0.851-1.023) |
| PRDX5 | eqtl-a-ENSG00000126432 | Colorectal cancer || id:ebi-a-GCST012879 | Inverse variance weighted (fixed effects) | 11 | 0.031438582 | 0.939(0.887-0.994) |
| PRDX5 | eqtl-a-ENSG00000126432 | Colorectal cancer || id:ebi-a-GCST012879 | Simple mode | 11 | 0.987263875 | 1.001(0.867-1.156) |
| PRDX5 | eqtl-a-ENSG00000126432 | Colorectal cancer || id:ebi-a-GCST012879 | Weighted median | 11 | 0.087690591 | 0.944(0.884-1.009) |
| PRDX5 | eqtl-a-ENSG00000126432 | Colorectal cancer || id:ebi-a-GCST012879 | Weighted mode | 11 | 0.163436561 | 0.949(0.887-1.016) |
| PPOX | eqtl-a-ENSG00000143224 | Colorectal cancer || id:ebi-a-GCST012879 | Inverse variance weighted (fixed effects) | 2 | 0.110136055 | 0.908(0.806-1.022) |
| UCP2 | eqtl-a-ENSG00000175567 | Colorectal cancer || id:ebi-a-GCST012879 | MR Egger | 6 | 0.273589986 | 0.866(0.693-1.082) |
| UCP2 | eqtl-a-ENSG00000175567 | Colorectal cancer || id:ebi-a-GCST012879 | Weighted median | 6 | 0.044131236 | 0.924(0.855-0.998) |
| UCP2 | eqtl-a-ENSG00000175567 | Colorectal cancer || id:ebi-a-GCST012879 | Inverse variance weighted (fixed effects) | 6 | 0.024380042 | 0.921(0.858-0.989) |
| UCP2 | eqtl-a-ENSG00000175567 | Colorectal cancer || id:ebi-a-GCST012879 | Weighted mode | 6 | 0.097873105 | 0.918(0.846-0.997) |
| UCP2 | eqtl-a-ENSG00000175567 | Colorectal cancer || id:ebi-a-GCST012879 | Simple mode | 6 | 0.145197089 | 0.882(0.765-1.017) |
| NDUFAF3 | eqtl-a-ENSG00000178057 | Colorectal cancer || id:ebi-a-GCST012879 | Inverse variance weighted (fixed effects) | 2 | 0.514826079 | 1.053(0.901-1.231) |
| LYRM7 | eqtl-a-ENSG00000186687 | Colorectal cancer || id:ebi-a-GCST012879 | Inverse variance weighted (fixed effects) | 2 | 0.359649611 | 0.932(0.801-1.084) |
| NDUFB2 | eqtl-a-ENSG00000090266 | Colorectal cancer || id:ebi-a-GCST012878 | Inverse variance weighted (fixed effects) | 2 | 0.380000609 | 1.11(0.879-1.401) |
| ALKBH1 | eqtl-a-ENSG00000100601 | Colorectal cancer || id:ebi-a-GCST012878 | Weighted median | 3 | 0.807024038 | 1.045(0.736-1.483) |
| ALKBH1 | eqtl-a-ENSG00000100601 | Colorectal cancer || id:ebi-a-GCST012878 | Weighted mode | 3 | 0.919259479 | 0.978(0.672-1.424) |
| ALKBH1 | eqtl-a-ENSG00000100601 | Colorectal cancer || id:ebi-a-GCST012878 | Simple mode | 3 | 0.243896207 | 2.006(0.87-4.624) |
| ALKBH1 | eqtl-a-ENSG00000100601 | Colorectal cancer || id:ebi-a-GCST012878 | MR Egger | 3 | 0.572827825 | 0.8(0.461-1.389) |
| ALKBH1 | eqtl-a-ENSG00000100601 | Colorectal cancer || id:ebi-a-GCST012878 | Inverse variance weighted (fixed effects) | 3 | 0.774718428 | 1.051(0.749-1.474) |
| GCDH | eqtl-a-ENSG00000105607 | Colorectal cancer || id:ebi-a-GCST012878 | Simple mode | 3 | 0.500816492 | 1.184(0.789-1.777) |
| GCDH | eqtl-a-ENSG00000105607 | Colorectal cancer || id:ebi-a-GCST012878 | MR Egger | 3 | 0.783358722 | 0.721(0.118-4.397) |
| GCDH | eqtl-a-ENSG00000105607 | Colorectal cancer || id:ebi-a-GCST012878 | Weighted median | 3 | 0.302955954 | 1.137(0.89-1.453) |
| GCDH | eqtl-a-ENSG00000105607 | Colorectal cancer || id:ebi-a-GCST012878 | Inverse variance weighted (fixed effects) | 3 | 0.268289778 | 1.149(0.899-1.468) |
| GCDH | eqtl-a-ENSG00000105607 | Colorectal cancer || id:ebi-a-GCST012878 | Weighted mode | 3 | 0.460899618 | 1.13(0.867-1.473) |
| MRPL27 | eqtl-a-ENSG00000108826 | Colorectal cancer || id:ebi-a-GCST012878 | Inverse variance weighted (fixed effects) | 2 | 0.82546441 | 0.953(0.621-1.463) |
| PRDX5 | eqtl-a-ENSG00000126432 | Colorectal cancer || id:ebi-a-GCST012878 | Inverse variance weighted (fixed effects) | 11 | 0.524628767 | 1.063(0.881-1.282) |
| PRDX5 | eqtl-a-ENSG00000126432 | Colorectal cancer || id:ebi-a-GCST012878 | Simple mode | 11 | 0.917293321 | 1.025(0.649-1.62) |
| PRDX5 | eqtl-a-ENSG00000126432 | Colorectal cancer || id:ebi-a-GCST012878 | Weighted mode | 11 | 0.358248031 | 1.111(0.897-1.377) |
| PRDX5 | eqtl-a-ENSG00000126432 | Colorectal cancer || id:ebi-a-GCST012878 | MR Egger | 11 | 0.523342511 | 1.13(0.788-1.622) |
| PRDX5 | eqtl-a-ENSG00000126432 | Colorectal cancer || id:ebi-a-GCST012878 | Weighted median | 11 | 0.364396799 | 1.112(0.884-1.398) |
| PPOX | eqtl-a-ENSG00000143224 | Colorectal cancer || id:ebi-a-GCST012878 | Inverse variance weighted (fixed effects) | 2 | 0.58247554 | 1.118(0.751-1.664) |
| UCP2 | eqtl-a-ENSG00000175567 | Colorectal cancer || id:ebi-a-GCST012878 | MR Egger | 6 | 0.311120491 | 0.444(0.112-1.755) |
| UCP2 | eqtl-a-ENSG00000175567 | Colorectal cancer || id:ebi-a-GCST012878 | Simple mode | 6 | 0.704355317 | 0.888(0.499-1.583) |
| UCP2 | eqtl-a-ENSG00000175567 | Colorectal cancer || id:ebi-a-GCST012878 | Weighted mode | 6 | 0.055283562 | 0.719(0.555-0.932) |
| UCP2 | eqtl-a-ENSG00000175567 | Colorectal cancer || id:ebi-a-GCST012878 | Weighted median | 6 | 0.037199811 | 0.755(0.579-0.983) |
| UCP2 | eqtl-a-ENSG00000175567 | Colorectal cancer || id:ebi-a-GCST012878 | Inverse variance weighted (multiplicative random effects) | 6 | 0.325825336 | 0.802(0.517-1.245) |
| NDUFAF3 | eqtl-a-ENSG00000178057 | Colorectal cancer || id:ebi-a-GCST012878 | Inverse variance weighted (fixed effects) | 2 | 0.865571302 | 0.956(0.569-1.608) |
| LYRM7 | eqtl-a-ENSG00000186687 | Colorectal cancer || id:ebi-a-GCST012878 | Inverse variance weighted (fixed effects) | 2 | 0.488587934 | 0.832(0.494-1.401) |
| NDUFB2 | eqtl-a-ENSG00000090266 | Colorectal cancer || id:ebi-a-GCST012877 | Inverse variance weighted (fixed effects) | 2 | 0.209322627 | 1.054(0.971-1.144) |
| ALKBH1 | eqtl-a-ENSG00000100601 | Colorectal cancer || id:ebi-a-GCST012877 | Weighted mode | 3 | 0.367165477 | 1.079(0.948-1.228) |
| ALKBH1 | eqtl-a-ENSG00000100601 | Colorectal cancer || id:ebi-a-GCST012877 | Weighted median | 3 | 0.245942519 | 1.074(0.952-1.21) |
| ALKBH1 | eqtl-a-ENSG00000100601 | Colorectal cancer || id:ebi-a-GCST012877 | Simple mode | 3 | 0.439799855 | 1.104(0.902-1.352) |
| ALKBH1 | eqtl-a-ENSG00000100601 | Colorectal cancer || id:ebi-a-GCST012877 | Inverse variance weighted (fixed effects) | 3 | 0.25861143 | 1.07(0.952-1.202) |
| ALKBH1 | eqtl-a-ENSG00000100601 | Colorectal cancer || id:ebi-a-GCST012877 | MR Egger | 3 | 0.535728548 | 1.09(0.902-1.318) |
| GCDH | eqtl-a-ENSG00000105607 | Colorectal cancer || id:ebi-a-GCST012877 | Simple mode | 3 | 0.765311326 | 0.972(0.828-1.142) |
| GCDH | eqtl-a-ENSG00000105607 | Colorectal cancer || id:ebi-a-GCST012877 | Weighted median | 3 | 0.080255468 | 1.077(0.991-1.17) |
| GCDH | eqtl-a-ENSG00000105607 | Colorectal cancer || id:ebi-a-GCST012877 | Weighted mode | 3 | 0.22287486 | 1.085(0.99-1.189) |
| GCDH | eqtl-a-ENSG00000105607 | Colorectal cancer || id:ebi-a-GCST012877 | Inverse variance weighted (fixed effects) | 3 | 0.089063108 | 1.075(0.989-1.168) |
| GCDH | eqtl-a-ENSG00000105607 | Colorectal cancer || id:ebi-a-GCST012877 | MR Egger | 3 | 0.474584457 | 1.406(0.759-2.605) |
| MRPL27 | eqtl-a-ENSG00000108826 | Colorectal cancer || id:ebi-a-GCST012877 | Inverse variance weighted (fixed effects) | 2 | 0.038460768 | 0.854(0.736-0.992) |
| PRDX5 | eqtl-a-ENSG00000126432 | Colorectal cancer || id:ebi-a-GCST012877 | Weighted mode | 11 | 0.628400742 | 0.98(0.905-1.061) |
| PRDX5 | eqtl-a-ENSG00000126432 | Colorectal cancer || id:ebi-a-GCST012877 | Weighted median | 11 | 0.468663886 | 0.973(0.902-1.049) |
| PRDX5 | eqtl-a-ENSG00000126432 | Colorectal cancer || id:ebi-a-GCST012877 | Inverse variance weighted (fixed effects) | 11 | 0.49684554 | 0.977(0.915-1.044) |
| PRDX5 | eqtl-a-ENSG00000126432 | Colorectal cancer || id:ebi-a-GCST012877 | Simple mode | 11 | 0.535581252 | 1.051(0.903-1.222) |
| PRDX5 | eqtl-a-ENSG00000126432 | Colorectal cancer || id:ebi-a-GCST012877 | MR Egger | 11 | 0.93436241 | 0.995(0.876-1.129) |
| PPOX | eqtl-a-ENSG00000143224 | Colorectal cancer || id:ebi-a-GCST012877 | Inverse variance weighted (fixed effects) | 2 | 0.19182136 | 1.094(0.956-1.252) |
| UCP2 | eqtl-a-ENSG00000175567 | Colorectal cancer || id:ebi-a-GCST012877 | Weighted mode | 6 | 0.456251167 | 1.039(0.947-1.14) |
| UCP2 | eqtl-a-ENSG00000175567 | Colorectal cancer || id:ebi-a-GCST012877 | MR Egger | 6 | 0.987688339 | 0.997(0.739-1.346) |
| UCP2 | eqtl-a-ENSG00000175567 | Colorectal cancer || id:ebi-a-GCST012877 | Weighted median | 6 | 0.560015518 | 1.026(0.941-1.118) |
| UCP2 | eqtl-a-ENSG00000175567 | Colorectal cancer || id:ebi-a-GCST012877 | Inverse variance weighted (fixed effects) | 6 | 0.531715286 | 1.026(0.946-1.113) |
| UCP2 | eqtl-a-ENSG00000175567 | Colorectal cancer || id:ebi-a-GCST012877 | Simple mode | 6 | 0.329789006 | 0.876(0.689-1.114) |
| NDUFAF3 | eqtl-a-ENSG00000178057 | Colorectal cancer || id:ebi-a-GCST012877 | Inverse variance weighted (fixed effects) | 2 | 0.767848149 | 0.973(0.814-1.164) |
| LYRM7 | eqtl-a-ENSG00000186687 | Colorectal cancer || id:ebi-a-GCST012877 | Inverse variance weighted (fixed effects) | 2 | 0.516507347 | 1.06(0.888-1.265) |
| NDUFB2 | eqtl-a-ENSG00000090266 | Colorectal cancer || id:ebi-a-GCST012876 | Inverse variance weighted (multiplicative random effects) | 2 | 0.567900023 | 0.947(0.787-1.141) |
| ALKBH1 | eqtl-a-ENSG00000100601 | Colorectal cancer || id:ebi-a-GCST012876 | Weighted mode | 3 | 0.319925213 | 0.924(0.821-1.04) |
| ALKBH1 | eqtl-a-ENSG00000100601 | Colorectal cancer || id:ebi-a-GCST012876 | Simple mode | 3 | 0.981589363 | 1.003(0.809-1.242) |
| ALKBH1 | eqtl-a-ENSG00000100601 | Colorectal cancer || id:ebi-a-GCST012876 | MR Egger | 3 | 0.869775118 | 0.959(0.645-1.425) |
| ALKBH1 | eqtl-a-ENSG00000100601 | Colorectal cancer || id:ebi-a-GCST012876 | Weighted median | 3 | 0.183580108 | 0.927(0.828-1.037) |
| ALKBH1 | eqtl-a-ENSG00000100601 | Colorectal cancer || id:ebi-a-GCST012876 | Inverse variance weighted (fixed effects) | 3 | 0.113624833 | 0.916(0.821-1.021) |
| GCDH | eqtl-a-ENSG00000105607 | Colorectal cancer || id:ebi-a-GCST012876 | Weighted mode | 3 | 0.36028389 | 0.95(0.871-1.035) |
| GCDH | eqtl-a-ENSG00000105607 | Colorectal cancer || id:ebi-a-GCST012876 | MR Egger | 3 | 0.983573175 | 1.007(0.576-1.763) |
| GCDH | eqtl-a-ENSG00000105607 | Colorectal cancer || id:ebi-a-GCST012876 | Weighted median | 3 | 0.175583413 | 0.947(0.876-1.025) |
| GCDH | eqtl-a-ENSG00000105607 | Colorectal cancer || id:ebi-a-GCST012876 | Simple mode | 3 | 0.356940573 | 0.924(0.811-1.053) |
| GCDH | eqtl-a-ENSG00000105607 | Colorectal cancer || id:ebi-a-GCST012876 | Inverse variance weighted (fixed effects) | 3 | 0.167013834 | 0.947(0.876-1.023) |
| MRPL27 | eqtl-a-ENSG00000108826 | Colorectal cancer || id:ebi-a-GCST012876 | Inverse variance weighted (fixed effects) | 2 | 0.352027906 | 1.068(0.93-1.228) |
| PRDX5 | eqtl-a-ENSG00000126432 | Colorectal cancer || id:ebi-a-GCST012876 | MR Egger | 11 | 0.256804786 | 1.068(0.96-1.187) |
| PRDX5 | eqtl-a-ENSG00000126432 | Colorectal cancer || id:ebi-a-GCST012876 | Weighted median | 11 | 0.606047791 | 1.02(0.946-1.1) |
| PRDX5 | eqtl-a-ENSG00000126432 | Colorectal cancer || id:ebi-a-GCST012876 | Weighted mode | 11 | 0.536313379 | 1.025(0.951-1.105) |
| PRDX5 | eqtl-a-ENSG00000126432 | Colorectal cancer || id:ebi-a-GCST012876 | Simple mode | 11 | 0.903655425 | 1.012(0.841-1.217) |
| PRDX5 | eqtl-a-ENSG00000126432 | Colorectal cancer || id:ebi-a-GCST012876 | Inverse variance weighted (fixed effects) | 11 | 0.911461984 | 0.997(0.937-1.06) |
| PPOX | eqtl-a-ENSG00000143224 | Colorectal cancer || id:ebi-a-GCST012876 | Inverse variance weighted (fixed effects) | 2 | 0.113039971 | 1.107(0.976-1.256) |
| UCP2 | eqtl-a-ENSG00000175567 | Colorectal cancer || id:ebi-a-GCST012876 | MR Egger | 6 | 0.649716078 | 1.073(0.81-1.422) |
| UCP2 | eqtl-a-ENSG00000175567 | Colorectal cancer || id:ebi-a-GCST012876 | Weighted median | 6 | 0.383122907 | 0.966(0.893-1.045) |
| UCP2 | eqtl-a-ENSG00000175567 | Colorectal cancer || id:ebi-a-GCST012876 | Weighted mode | 6 | 0.522710976 | 0.972(0.895-1.055) |
| UCP2 | eqtl-a-ENSG00000175567 | Colorectal cancer || id:ebi-a-GCST012876 | Inverse variance weighted (fixed effects) | 6 | 0.177562886 | 0.948(0.878-1.024) |
| UCP2 | eqtl-a-ENSG00000175567 | Colorectal cancer || id:ebi-a-GCST012876 | Simple mode | 6 | 0.888348093 | 0.984(0.796-1.217) |
| NDUFAF3 | eqtl-a-ENSG00000178057 | Colorectal cancer || id:ebi-a-GCST012876 | Inverse variance weighted (fixed effects) | 2 | 0.317990098 | 1.091(0.92-1.294) |
| LYRM7 | eqtl-a-ENSG00000186687 | Colorectal cancer || id:ebi-a-GCST012876 | Inverse variance weighted (fixed effects) | 2 | 0.221790055 | 0.903(0.767-1.064) |

**Supplementary Table 2.** SMR and colocalization results of the association between mitochondria-related genes eQTL and CRC outcomes.

| **Gene** | **Probe ID** | **Probe Chr** | **Probe bp** | **TopSNP** | **TopSNP chr** | **TopSNP bp** | **A1** | **A2** | **Freq** | **GWAS association** | | | **eQTL association** | | | **SMR association** | | | **HEIDI Test** | | **Date** |
| --- | --- | --- | --- | --- | --- | --- | --- | --- | --- | --- | --- | --- | --- | --- | --- | --- | --- | --- | --- | --- | --- |
|  |  |  |  |  |  |  |  |  |  | **b** | **se** | ***p*** | **b** | **se** | ***p*** | **b** | **se** | ***p*** | ***p*** | **No. of**  **SNPs** |  |
| BAD | ENSG00000002330 | 11 | 64044739 | rs947939 | 11 | 63885287 | T | C | 0.139165 | 0.1417 | 0.0411 | 0.000571702 | 0.0907893 | 0.0111022 | 2.90E-16 | 1.56076 | 0.491285 | 0.001488646 | 0.5378173 | 3 | finn-b-C3-COLO_EX |
| BAD | ENSG00000002330 | 11 | 64044739 | rs947939 | 11 | 63885287 | T | C | 0.139165 | 0.1356 | 0.0407 | 0.000858005 | 0.0907893 | 0.0111022 | 2.90E-16 | 1.49357 | 0.484069 | 0.002032464 | 0.5181393 | 3 | data |
| NDUFB2 | ENSG00000090266 | 7 | 140406583 | rs2968555 | 7 | 140379261 | G | A | 0.158052 | -0.0745257 | 0.0330119 | 0.022837 | -0.444305 | 0.0107654 | 0 | 0.167735 | 0.0744111 | 0.02418543 | 0.786313 | 3 | ebi-a-GCST90013862 |
| NDUFB2 | ENSG00000090266 | 7 | 140406583 | rs2968555 | 7 | 140379261 | G | A | 0.158052 | -0.0736865 | 0.0322201 | 0.0221973 | -0.444305 | 0.0107654 | 0 | 0.165847 | 0.0726292 | 0.0224027 | 0.7932114 | 3 | ebi-a-GCST90013866 |
| ALKBH1 | ENSG00000100601 | 14 | 78156555 | rs56412551 | 14 | 78145196 | G | A | 0.270378 | -0.000639159 | 0.000317057 | 0.0439997 | 0.236826 | 0.00891741 | 2.09E-155 | -0.00269886 | 0.00134263 | 0.04441751 | 0.3167271 | 3 | ieu-b-4965 |
| ALKBH1 | ENSG00000100601 | 14 | 78156555 | rs56412551 | 14 | 78145196 | G | A | 0.270378 | -0.031 | 0.0144 | 0.0316599 | 0.236826 | 0.00891741 | 2.09E-155 | -0.130898 | 0.0610036 | 0.03189375 | 0.7065004 | 3 | ebi-a-GCST90018808 |
| ALKBH1 | ENSG00000100601 | 14 | 78156555 | rs56412551 | 14 | 78145196 | G | A | 0.270378 | -0.000639159 | 0.000317057 | 0.0439997 | 0.236826 | 0.00891741 | 2.09E-155 | -0.00269886 | 0.00134263 | 0.04441751 | 0.3167271 | 3 | ieu-b-4965 |
| GCDH | ENSG00000105607 | 19 | 13013430 | rs1799918 | 19 | 13002400 | C | G | 0.327038 | 0.0681 | 0.0291 | 0.0193798 | 0.312662 | 0.00827454 | 0 | 0.217807 | 0.0932501 | 0.01950527 | 0.2337711 | 3 | finn-b-C3-COLO_EX |
| GCDH | ENSG00000105607 | 19 | 13013430 | rs1799918 | 19 | 13002400 | C | G | 0.327038 | 0.0635 | 0.0288 | 0.0272301 | 0.312662 | 0.00827454 | 0 | 0.203095 | 0.0922689 | 0.02772774 | 0.2995288 | 3 | finn-b-C3-COLO |
| GCDH | ENSG00000105607 | 19 | 13013430 | rs1799918 | 19 | 13002400 | C | G | 0.327038 | 0.0357 | 0.015 | 0.0174301 | 0.312662 | 0.00827454 | 0 | 0.114181 | 0.0480702 | 0.01753503 | 0.2267494 | 3 | ebi-a-GCST90018808 |
| MRPL27 | ENSG00000108826 | 17 | 48447896 | rs11868055 | 17 | 48449270 | G | A | 0.446322 | 0.039434 | 0.019327 | 0.04132 | -0.165867 | 0.00804548 | 1.96E-94 | -0.237744 | 0.11709 | 0.042312 | 0.7580367 | 4 | ebi-a-GCST012877 |
| ACAD10 | ENSG00000111271 | 12 | 112159380 | rs10774634 | 12 | 112099245 | G | A | 0.16998 | 0.082675 | 0.025214 | 0.00104201 | 0.104784 | 0.0104402 | 1.05E-23 | 0.789001 | 0.253143 | 0.001828148 | 0.5871333 | 3 | ebi-a-GCST012877 |
| TSFM | ENSG00000123297 | 12 | 58189113 | rs1599932 | 12 | 58177943 | A | G | 0.318091 | 0.0503372 | 0.0192148 | 0.00880035 | -0.284544 | 0.0087018 | 1.59E-234 | -0.176905 | 0.0677448 | 0.009018742 | 0.5423486 | 3 | ebi-a-GCST012876 |
| PRDX5 | ENSG00000126432 | 11 | 64087421 | rs595880 | 11 | 64006466 | T | G | 0.335984 | -0.0380615 | 0.0182118 | 0.0366235 | 0.439357 | 0.0087235 | 0 | -0.08663 | 0.0414867 | 0.03678528 | 0.8349984 | 4 | ebi-a-GCST012879 |
| PPOX | ENSG00000143224 | 1 | 161142001 | rs12031437 | 1 | 161126975 | G | C | 0.399602 | 0.0498032 | 0.0226662 | 0.0283576 | -0.185975 | 0.00821103 | 1.41E-113 | -0.267795 | 0.12245 | 0.02874411 | 0.3418563 | 4 | ebi-a-GCST90013862 |
| PPOX | ENSG00000143224 | 1 | 161142001 | rs12031437 | 1 | 161126975 | G | C | 0.399602 | 0.0364 | 0.0123 | 0.00316599 | -0.185975 | 0.00821103 | 1.41E-113 | -0.195725 | 0.0667001 | 0.003341848 | 0.6426935 | 4 | ebi-a-GCST90018808 |
| PPOX | ENSG00000143224 | 1 | 161142001 | rs12031437 | 1 | 161126975 | G | C | 0.399602 | 0.000796801 | 0.000288225 | 0.00569994 | -0.185975 | 0.00821103 | 1.41E-113 | -0.00428445 | 0.00156131 | 0.006066873 | 0.2769649 | 4 | ieu-b-4965 |
| PPOX | ENSG00000143224 | 1 | 161142001 | rs12031437 | 1 | 161126975 | G | C | 0.399602 | 0.0500425 | 0.0228269 | 0.0283609 | -0.185975 | 0.00821103 | 1.41E-113 | -0.269082 | 0.123315 | 0.02910492 | 0.3383904 | 4 | ebi-a-GCST90013866 |
| PPOX | ENSG00000143224 | 1 | 161142001 | rs12031437 | 1 | 161126975 | G | C | 0.399602 | 0.000796801 | 0.000288225 | 0.00569994 | -0.185975 | 0.00821103 | 1.41E-113 | -0.00428445 | 0.00156131 | 0.006066873 | 0.2769649 | 4 | ieu-b-4965 |
| NIT1 | ENSG00000158793 | 1 | 161091555 | rs75372745 | 1 | 161082186 | C | G | 0.0735586 | 0.00140459 | 0.000516426 | 0.00649995 | -0.157683 | 0.0147843 | 1.47E-26 | -0.00890767 | 0.0033799 | 0.008401706 | 0.1284406 | 4 | ieu-b-4965 |
| NIT1 | ENSG00000158793 | 1 | 161091555 | rs75372745 | 1 | 161082186 | C | G | 0.0735586 | 0.00140459 | 0.000516426 | 0.00649995 | -0.157683 | 0.0147843 | 1.47E-26 | -0.00890767 | 0.0033799 | 0.008401706 | 0.1284406 | 4 | ieu-b-4965 |
| NDUFS2 | ENSG00000158864 | 1 | 161175539 | rs4379692 | 1 | 161186313 | T | C | 0.319085 | 0.0401 | 0.0126 | 0.00151001 | -0.225171 | 0.00863498 | 6.72E-150 | -0.178087 | 0.0563727 | 0.001582577 | 0.8424793 | 5 | ebi-a-GCST90018808 |
| NDUFS2 | ENSG00000158864 | 1 | 161175539 | rs4379692 | 1 | 161186313 | T | C | 0.319085 | 0.00104085 | 0.000301256 | 0.000549997 | -0.225171 | 0.00863498 | 6.72E-150 | -0.00462249 | 0.00134959 | 0.000614569 | 0.1716254 | 5 | ieu-b-4965 |
| NDUFS2 | ENSG00000158864 | 1 | 161175539 | rs4379692 | 1 | 161186313 | T | C | 0.319085 | 0.00104085 | 0.000301256 | 0.000549997 | -0.225171 | 0.00863498 | 6.72E-150 | -0.00462249 | 0.00134959 | 0.000614569 | 0.1716254 | 5 | ieu-b-4965 |
| NDUFS2 | ENSG00000158864 | 1 | 161175539 | rs4379692 | 1 | 161186313 | T | C | 0.319085 | 0.0749544 | 0.0238416 | 0.00166744 | -0.225171 | 0.00863498 | 6.72E-150 | -0.332878 | 0.106649 | 0.001800868 | 0.9209829 | 5 | ebi-a-GCST90013866 |
| NDUFS2 | ENSG00000158864 | 1 | 161175539 | rs4379692 | 1 | 161186313 | T | C | 0.319085 | 0.0742191 | 0.0234885 | 0.00166537 | -0.225171 | 0.00863498 | 6.72E-150 | -0.329612 | 0.105077 | 0.001707675 | 0.9191693 | 5 | ebi-a-GCST90013862 |
| RAB24 | ENSG00000169228 | 5 | 176729472 | rs4976642 | 5 | 176673068 | T | A | 0.163022 | 0.029 | 0.0135 | 0.0318302 | -0.168959 | 0.00995108 | 1.17E-64 | -0.171639 | 0.0805378 | 0.03307602 | 0.2474712 | 3 | ebi-a-GCST90018808 |
| RAB24 | ENSG00000169228 | 5 | 176729472 | rs4976642 | 5 | 176673068 | T | A | 0.163022 | 0.057767 | 0.0239441 | 0.0158402 | -0.168959 | 0.00995108 | 1.17E-64 | -0.341899 | 0.143139 | 0.01691338 | 0.9240309 | 3 | ebi-a-GCST012876 |
| HINT1 | ENSG00000169567 | 5 | 130501074 | rs3864285 | 5 | 130497902 | G | A | 0.0357853 | 0.00208956 | 0.00082322 | 0.0109999 | -0.694672 | 0.0333016 | 1.24E-96 | -0.00300798 | 0.00119379 | 0.01174578 | 0.2700426 | 3 | ieu-b-4965 |
| HINT1 | ENSG00000169567 | 5 | 130501074 | rs3864285 | 5 | 130497902 | G | A | 0.0357853 | 0.00208956 | 0.00082322 | 0.0109999 | -0.694672 | 0.0333016 | 1.24E-96 | -0.00300798 | 0.00119379 | 0.01174578 | 0.2700426 | 3 | ieu-b-4965 |
| UCP3 | ENSG00000175564 | 11 | 73715903 | rs3781907 | 11 | 73716469 | G | A | 0.26839 | -0.000808191 | 0.00031007 | 0.00909997 | -0.165831 | 0.00873376 | 2.17E-80 | 0.00487357 | 0.00188733 | 0.009815492 | 0.8475453 | 3 | ieu-b-4965 |
| UCP3 | ENSG00000175564 | 11 | 73715903 | rs3781907 | 11 | 73716469 | G | A | 0.26839 | -0.000808191 | 0.00031007 | 0.00909997 | -0.165831 | 0.00873376 | 2.17E-80 | 0.00487357 | 0.00188733 | 0.009815492 | 0.8475453 | 3 | ieu-b-4965 |
| UCP3 | ENSG00000175564 | 11 | 73715903 | rs3781907 | 11 | 73716469 | G | A | 0.26839 | -0.0519019 | 0.024823 | 0.0359435 | -0.165831 | 0.00873376 | 2.17E-80 | 0.31298 | 0.150593 | 0.03768024 | 0.7323229 | 3 | ebi-a-GCST90013862 |
| UCP3 | ENSG00000175564 | 11 | 73715903 | rs3781907 | 11 | 73716469 | G | A | 0.26839 | -0.0517952 | 0.0245937 | 0.0352014 | -0.165831 | 0.00873376 | 2.17E-80 | 0.312337 | 0.149215 | 0.03633142 | 0.733792 | 3 | ebi-a-GCST90013866 |
| UCP2 | ENSG00000175567 | 11 | 73690032 | rs1976139 | 11 | 73670977 | G | T | 0.107356 | -0.237455 | 0.0924733 | 0.0103001 | 0.470708 | 0.0124314 | 0 | -0.504464 | 0.196907 | 0.01040899 | 0.5982691 | 3 | ebi-a-GCST012878 |
| NDUFAF3 | ENSG00000178057 | 3 | 49059410 | rs990211 | 3 | 48721040 | A | G | 0.201789 | -0.0564 | 0.0243 | 0.0200498 | -0.130147 | 0.0113656 | 2.32E-30 | 0.433356 | 0.190509 | 0.02292202 | 0.1016093 | 3 | ebi-a-GCST90018808 |
| LYRM7 | ENSG00000186687 | 5 | 130523811 | rs6595994 | 5 | 130403098 | T | C | 0.0924453 | 0.00119745 | 0.000520293 | 0.021 | -0.229004 | 0.0142717 | 6.10E-58 | -0.00522894 | 0.00229523 | 0.02271602 | 0.1524128 | 3 | ieu-b-4965 |
| LYRM7 | ENSG00000186687 | 5 | 130523811 | rs6595994 | 5 | 130403098 | T | C | 0.0924453 | -0.1296 | 0.0498 | 0.00923804 | -0.229004 | 0.0142717 | 6.10E-58 | 0.565928 | 0.220305 | 0.01020384 | 0.2486917 | 3 | finn-b-C3-COLO_EX |
| LYRM7 | ENSG00000186687 | 5 | 130523811 | rs6595994 | 5 | 130403098 | T | C | 0.0924453 | 0.00119745 | 0.000520293 | 0.021 | -0.229004 | 0.0142717 | 6.10E-58 | -0.00522894 | 0.00229523 | 0.02271602 | 0.1524128 | 3 | ieu-b-4965 |
| LYRM7 | ENSG00000186687 | 5 | 130523811 | rs6595994 | 5 | 130403098 | T | C | 0.0924453 | -0.1238 | 0.0492 | 0.01193 | -0.229004 | 0.0142717 | 6.10E-58 | 0.540601 | 0.217469 | 0.01292313 | 0.1713633 | 3 | finn-b-C3-COLO |
| MARS2 | ENSG00000247626 | 2 | 198571600 | rs2341510 | 2 | 198558679 | T | C | 0.513917 | 0.0481136 | 0.0222035 | 0.0302392 | 0.202769 | 0.00787503 | 3.36E-146 | 0.237282 | 0.109888 | 0.03082656 | 0.1852866 | 3 | ebi-a-GCST90013866 |
| MARS2 | ENSG00000247626 | 2 | 198571600 | rs2341510 | 2 | 198558679 | T | C | 0.513917 | 0.0479881 | 0.0221384 | 0.0303312 | 0.202769 | 0.00787503 | 3.36E-146 | 0.236663 | 0.109566 | 0.03077266 | 0.1858833 | 3 | ebi-a-GCST90013862 |

**Supplementary Table 3.** Sensitivity analysis used TwoSampleMR package on the association between mitochondria-related genes eQTL and CRC outcomes.

| **Gene name** | **Outcome** | **Exposure** | **Method** | **Heterogeneity test** | | **Pleiotropy test** | |
| --- | --- | --- | --- | --- | --- | --- | --- |
|  |  |  |  | **Cochran's Q** | ***P*** | **Intercept** | ***P*** |
| LAP3 | Colorectal cancer || id:ebi-a-GCST012876 | ENSG00000002549 || id:eqtl-a-ENSG00000002549 | Inverse variance weighted | 3.812432673 | 0.702041852 | NA | NA |
| LAP3 | Colorectal cancer || id:ebi-a-GCST012876 | ENSG00000002549 || id:eqtl-a-ENSG00000002549 | MR Egger | 2.719841445 | 0.743082144 | -0.013798881 | 0.343775094 |
| CROT | Colorectal cancer || id:ebi-a-GCST012879 | ENSG00000005469 || id:eqtl-a-ENSG00000005469 | Inverse variance weighted | 2.346766454 | 0.125543179 | NA | NA |
| CROT | Colorectal cancer || id:ebi-a-GCST012880 | ENSG00000005469 || id:eqtl-a-ENSG00000005469 | Inverse variance weighted | 3.108872378 | 0.077866853 | NA | NA |
| AGK | Colorectal cancer || id:ebi-a-GCST012878 | ENSG00000006530 || id:eqtl-a-ENSG00000006530 | Inverse variance weighted | 1.855135214 | 0.395514588 | NA | NA |
| AGK | Colorectal cancer || id:ebi-a-GCST012878 | ENSG00000006530 || id:eqtl-a-ENSG00000006530 | MR Egger | 1.831518978 | 0.175948237 | -0.013749206 | 0.928018076 |
| COX10 | Colorectal cancer || id:ebi-a-GCST012876 | ENSG00000006695 || id:eqtl-a-ENSG00000006695 | Inverse variance weighted | 0.015243331 | 0.901739772 | NA | NA |
| COX10 | Colorectal cancer || id:ebi-a-GCST012878 | ENSG00000006695 || id:eqtl-a-ENSG00000006695 | Inverse variance weighted | 0.10569122 | 0.745103981 | NA | NA |
| LARS2 | Colorectal cancer || id:ebi-a-GCST90018808 | ENSG00000011376 || id:eqtl-a-ENSG00000011376 | Inverse variance weighted | 0.108002333 | 0.742430015 | NA | NA |
| BID | Colorectal cancer || id:ebi-a-GCST90018808 | ENSG00000015475 || id:eqtl-a-ENSG00000015475 | Inverse variance weighted | 3.279174834 | 0.070164198 | NA | NA |
| CYP24A1 | Colorectal cancer || id:ebi-a-GCST012877 | ENSG00000019186 || id:eqtl-a-ENSG00000019186 | Inverse variance weighted | 2.076009362 | 0.149630924 | NA | NA |
| NDUFS1 | Colorectal cancer || id:ebi-a-GCST012878 | ENSG00000023228 || id:eqtl-a-ENSG00000023228 | Inverse variance weighted | 0.2865949 | 0.592410907 | NA | NA |
| NDUFS1 | Colorectal cancer || id:ebi-a-GCST012876 | ENSG00000023228 || id:eqtl-a-ENSG00000023228 | Inverse variance weighted | 0.058491567 | 0.808896163 | NA | NA |
| NDUFS1 | Colorectal cancer || id:ebi-a-GCST90018588 | ENSG00000023228 || id:eqtl-a-ENSG00000023228 | Inverse variance weighted | 0.457060376 | 0.499001024 | NA | NA |
| NDUFS1 | Colorectal cancer || id:ebi-a-GCST90018808 | ENSG00000023228 || id:eqtl-a-ENSG00000023228 | Inverse variance weighted | 1.619759369 | 0.203125343 | NA | NA |
| TOMM34 | Colorectal cancer || id:ebi-a-GCST012876 | ENSG00000025772 || id:eqtl-a-ENSG00000025772 | Inverse variance weighted | 0.531740141 | 0.97033842 | NA | NA |
| TOMM34 | Colorectal cancer || id:ebi-a-GCST012876 | ENSG00000025772 || id:eqtl-a-ENSG00000025772 | MR Egger | 0.530642796 | 0.912109554 | 0.000965681 | 0.975654691 |
| AGPAT4 | Colorectal cancer || id:ebi-a-GCST012880 | ENSG00000026652 || id:eqtl-a-ENSG00000026652 | Inverse variance weighted | 7.291432135 | 0.121265788 | NA | NA |
| AGPAT4 | Colorectal cancer || id:ebi-a-GCST012880 | ENSG00000026652 || id:eqtl-a-ENSG00000026652 | MR Egger | 7.191795849 | 0.066029443 | -0.015888301 | 0.851502138 |
| MPC1 | Colorectal cancer || id:ebi-a-GCST012876 | ENSG00000060762 || id:eqtl-a-ENSG00000060762 | Inverse variance weighted | 0.371459077 | 0.542210019 | NA | NA |
| MPC1 | Colorectal cancer || id:finn-b-C3_COLORECTAL | ENSG00000060762 || id:eqtl-a-ENSG00000060762 | Inverse variance weighted | 3.376358506 | 0.066138033 | NA | NA |
| MPC1 | Colorectal cancer (all cancers excluded) || id:finn-b-C3_COLORECTAL_EXALLC | ENSG00000060762 || id:eqtl-a-ENSG00000060762 | Inverse variance weighted | 3.057271158 | 0.08037655 | NA | NA |
| ACAA1 | Colorectal cancer || id:ebi-a-GCST90018588 | ENSG00000060971 || id:eqtl-a-ENSG00000060971 | Inverse variance weighted | 2.00738585 | 0.156534845 | NA | NA |
| CASP8 | Colorectal cancer || id:ebi-a-GCST012876 | ENSG00000064012 || id:eqtl-a-ENSG00000064012 | MR Egger | 4.97961667 | 0.082925859 | -0.017395891 | 0.756115734 |
| CASP8 | Colorectal cancer || id:ebi-a-GCST90018588 | ENSG00000064012 || id:eqtl-a-ENSG00000064012 | Inverse variance weighted | 0.157992485 | 0.924043397 | NA | NA |
| CASP8 | Colorectal cancer || id:ebi-a-GCST012876 | ENSG00000064012 || id:eqtl-a-ENSG00000064012 | Inverse variance weighted | 5.294533032 | 0.151457528 | NA | NA |
| CASP8 | Colorectal cancer || id:ebi-a-GCST90018588 | ENSG00000064012 || id:eqtl-a-ENSG00000064012 | MR Egger | 0.146431626 | 0.701969018 | 0.003224274 | 0.931811696 |
| KARS1 | Colorectal cancer (all cancers excluded) || id:finn-b-C3_COLORECTAL_EXALLC | ENSG00000065427 || id:eqtl-a-ENSG00000065427 | Inverse variance weighted | 0.162558678 | 0.686811647 | NA | NA |
| KARS1 | Colorectal cancer || id:finn-b-C3_COLORECTAL | ENSG00000065427 || id:eqtl-a-ENSG00000065427 | Inverse variance weighted | 0.119981123 | 0.729054964 | NA | NA |
| KARS1 | Colorectal cancer || id:ebi-a-GCST012877 | ENSG00000065427 || id:eqtl-a-ENSG00000065427 | Inverse variance weighted | 2.182520928 | 0.139585659 | NA | NA |
| HDHD5 | Colorectal cancer || id:finn-b-C3_COLORECTAL | ENSG00000069998 || id:eqtl-a-ENSG00000069998 | Inverse variance weighted | 3.888164605 | 0.273796314 | NA | NA |
| HDHD5 | Colorectal cancer (all cancers excluded) || id:finn-b-C3_COLORECTAL_EXALLC | ENSG00000069998 || id:eqtl-a-ENSG00000069998 | Inverse variance weighted | 3.939789057 | 0.268039655 | NA | NA |
| HDHD5 | Colorectal cancer (all cancers excluded) || id:finn-b-C3_COLORECTAL_EXALLC | ENSG00000069998 || id:eqtl-a-ENSG00000069998 | MR Egger | 2.798066642 | 0.246835459 | 0.030421789 | 0.461675955 |
| HDHD5 | Colorectal cancer || id:finn-b-C3_COLORECTAL | ENSG00000069998 || id:eqtl-a-ENSG00000069998 | MR Egger | 2.933674298 | 0.230653856 | 0.027477864 | 0.504534476 |
| POLB | Colorectal cancer || id:ebi-a-GCST90018808 | ENSG00000070501 || id:eqtl-a-ENSG00000070501 | Inverse variance weighted | 4.60397255 | 0.330396416 | NA | NA |
| POLB | Colorectal cancer || id:ebi-a-GCST90018808 | ENSG00000070501 || id:eqtl-a-ENSG00000070501 | MR Egger | 3.636034474 | 0.303542736 | 0.010782294 | 0.437351044 |
| ACADVL | Colorectal cancer (SPA correction) || id:ebi-a-GCST90013866 | ENSG00000072778 || id:eqtl-a-ENSG00000072778 | MR Egger | 0.295115744 | 0.586960738 | 0.016587868 | 0.779971115 |
| ACADVL | Colorectal cancer || id:ieu-b-4965 | ENSG00000072778 || id:eqtl-a-ENSG00000072778 | MR Egger | 0.070110296 | 0.791176257 | 0.000420848 | 0.600946075 |
| ACADVL | Colorectal cancer || id:ieu-b-4965 | ENSG00000072778 || id:eqtl-a-ENSG00000072778 | Inverse variance weighted | 0.594683747 | 0.742790029 | NA | NA |
| ACADVL | Colorectal cancer (Firth correction) || id:ebi-a-GCST90013862 | ENSG00000072778 || id:eqtl-a-ENSG00000072778 | MR Egger | 0.29464533 | 0.587258957 | 0.01683904 | 0.776725075 |
| ACADVL | Colorectal cancer || id:ieu-b-4965 | ENSG00000072778 || id:eqtl-a-ENSG00000072778 | Inverse variance weighted | 0.594683747 | 0.742790029 | NA | NA |
| ACADVL | Colorectal cancer (Firth correction) || id:ebi-a-GCST90013862 | ENSG00000072778 || id:eqtl-a-ENSG00000072778 | Inverse variance weighted | 0.428487165 | 0.807151753 | NA | NA |
| ACADVL | Colorectal cancer (SPA correction) || id:ebi-a-GCST90013866 | ENSG00000072778 || id:eqtl-a-ENSG00000072778 | Inverse variance weighted | 0.424768603 | 0.808653871 | NA | NA |
| SLC25A3 | Colorectal cancer || id:ebi-a-GCST012878 | ENSG00000075415 || id:eqtl-a-ENSG00000075415 | MR Egger | 0.063499628 | 0.80104777 | -0.064731939 | 0.438219452 |
| SLC25A3 | Colorectal cancer || id:ebi-a-GCST012878 | ENSG00000075415 || id:eqtl-a-ENSG00000075415 | Inverse variance weighted | 1.541424506 | 0.462683403 | NA | NA |
| ACACB | Colorectal cancer || id:ebi-a-GCST012876 | ENSG00000076555 || id:eqtl-a-ENSG00000076555 | MR Egger | 0.66374801 | 0.717577728 | 0.017977311 | 0.426557114 |
| ACACB | Colorectal cancer || id:ebi-a-GCST012876 | ENSG00000076555 || id:eqtl-a-ENSG00000076555 | Inverse variance weighted | 1.64364892 | 0.649533711 | NA | NA |
| ACACB | Colorectal cancer || id:ebi-a-GCST012877 | ENSG00000076555 || id:eqtl-a-ENSG00000076555 | Inverse variance weighted | 2.627604253 | 0.452670704 | NA | NA |
| ACACB | Colorectal cancer || id:ebi-a-GCST012877 | ENSG00000076555 || id:eqtl-a-ENSG00000076555 | MR Egger | 2.255775302 | 0.323716337 | 0.012028495 | 0.623823533 |
| NOA1 | Colorectal cancer || id:ieu-b-4965 | ENSG00000084092 || id:eqtl-a-ENSG00000084092 | Inverse variance weighted | 4.48711851 | 0.106080265 | NA | NA |
| NOA1 | Colorectal cancer || id:ebi-a-GCST012878 | ENSG00000084092 || id:eqtl-a-ENSG00000084092 | MR Egger | 2.526717731 | 0.111932832 | -0.034526272 | 0.831421869 |
| NOA1 | Colorectal cancer || id:ebi-a-GCST012878 | ENSG00000084092 || id:eqtl-a-ENSG00000084092 | Inverse variance weighted | 2.712515137 | 0.257623112 | NA | NA |
| NOA1 | Colorectal cancer || id:ieu-b-4965 | ENSG00000084092 || id:eqtl-a-ENSG00000084092 | MR Egger | 3.621815642 | 0.057026606 | 0.000365414 | 0.710568347 |
| NOA1 | Colorectal cancer || id:ieu-b-4965 | ENSG00000084092 || id:eqtl-a-ENSG00000084092 | Inverse variance weighted | 4.48711851 | 0.106080265 | NA | NA |
| MRPL28 | Colorectal cancer || id:ieu-b-4965 | ENSG00000086504 || id:eqtl-a-ENSG00000086504 | Inverse variance weighted | 0.547824696 | 0.459207887 | NA | NA |
| MRPL28 | Colorectal cancer || id:ieu-b-4965 | ENSG00000086504 || id:eqtl-a-ENSG00000086504 | Inverse variance weighted | 0.547824696 | 0.459207887 | NA | NA |
| MRPL28 | Colorectal cancer || id:ebi-a-GCST90018808 | ENSG00000086504 || id:eqtl-a-ENSG00000086504 | Inverse variance weighted | 1.977406773 | 0.159663865 | NA | NA |
| PGS1 | Colorectal cancer (all cancers excluded) || id:finn-b-C3_COLORECTAL_EXALLC | ENSG00000087157 || id:eqtl-a-ENSG00000087157 | MR Egger | 3.208622323 | 0.073251137 | 0.016080443 | 0.862139227 |
| PGS1 | Colorectal cancer (all cancers excluded) || id:finn-b-C3_COLORECTAL_EXALLC | ENSG00000087157 || id:eqtl-a-ENSG00000087157 | Inverse variance weighted | 3.363920962 | 0.186008951 | NA | NA |
| PGS1 | Colorectal cancer || id:finn-b-C3_COLORECTAL | ENSG00000087157 || id:eqtl-a-ENSG00000087157 | Inverse variance weighted | 2.932675426 | 0.230769082 | NA | NA |
| PGS1 | Colorectal cancer || id:finn-b-C3_COLORECTAL | ENSG00000087157 || id:eqtl-a-ENSG00000087157 | MR Egger | 2.868429876 | 0.090332959 | 0.010222774 | 0.905426773 |
| NDUFB2 | Colorectal cancer (SPA correction) || id:ebi-a-GCST90013866 | ENSG00000090266 || id:eqtl-a-ENSG00000090266 | Inverse variance weighted | 0.791196746 | 0.373738501 | NA | NA |
| NDUFB2 | Colorectal cancer (Firth correction) || id:ebi-a-GCST90013862 | ENSG00000090266 || id:eqtl-a-ENSG00000090266 | Inverse variance weighted | 0.781688271 | 0.376625303 | NA | NA |
| MUL1 | Colorectal cancer || id:finn-b-C3_COLORECTAL | ENSG00000090432 || id:eqtl-a-ENSG00000090432 | Inverse variance weighted | 0.088942338 | 0.765526085 | NA | NA |
| MUL1 | Colorectal cancer (all cancers excluded) || id:finn-b-C3_COLORECTAL_EXALLC | ENSG00000090432 || id:eqtl-a-ENSG00000090432 | Inverse variance weighted | 0.062661346 | 0.80233797 | NA | NA |
| CRAT | Colorectal cancer || id:ebi-a-GCST012880 | ENSG00000095321 || id:eqtl-a-ENSG00000095321 | MR Egger | 9.072208446 | 0.336241229 | 0.015864065 | 0.376264208 |
| CRAT | Colorectal cancer || id:ebi-a-GCST012880 | ENSG00000095321 || id:eqtl-a-ENSG00000095321 | Inverse variance weighted | 10.06747445 | 0.345050453 | NA | NA |
| MRPS18A | Colorectal cancer (SPA correction) || id:ebi-a-GCST90013866 | ENSG00000096080 || id:eqtl-a-ENSG00000096080 | Inverse variance weighted | 0.470757085 | 0.49263908 | NA | NA |
| MRPS18A | Colorectal cancer || id:ebi-a-GCST012880 | ENSG00000096080 || id:eqtl-a-ENSG00000096080 | Inverse variance weighted | 2.213322708 | 0.136823695 | NA | NA |
| MRPS18A | Colorectal cancer (Firth correction) || id:ebi-a-GCST90013862 | ENSG00000096080 || id:eqtl-a-ENSG00000096080 | Inverse variance weighted | 0.537808509 | 0.463342373 | NA | NA |
| ACOT7 | Colorectal cancer (all cancers excluded) || id:finn-b-C3_COLORECTAL_EXALLC | ENSG00000097021 || id:eqtl-a-ENSG00000097021 | Inverse variance weighted | 3.089414971 | 0.078803136 | NA | NA |
| ACOT7 | Colorectal cancer || id:ebi-a-GCST012880 | ENSG00000097021 || id:eqtl-a-ENSG00000097021 | Inverse variance weighted | 1.820966006 | 0.177198333 | NA | NA |
| GCAT | Colorectal cancer || id:ebi-a-GCST90018588 | ENSG00000100116 || id:eqtl-a-ENSG00000100116 | MR Egger | 1.403888235 | 0.843518942 | -0.02055569 | 0.404560599 |
| GCAT | Colorectal cancer || id:ebi-a-GCST90018588 | ENSG00000100116 || id:eqtl-a-ENSG00000100116 | Inverse variance weighted | 2.270626919 | 0.810571178 | NA | NA |
| GCAT | Colorectal cancer || id:ebi-a-GCST90018808 | ENSG00000100116 || id:eqtl-a-ENSG00000100116 | Inverse variance weighted | 12.60745382 | 0.18118767 | NA | NA |
| GCAT | Colorectal cancer || id:ebi-a-GCST90018808 | ENSG00000100116 || id:eqtl-a-ENSG00000100116 | MR Egger | 12.60710486 | 0.126101972 | -0.000138066 | 0.988491636 |
| PICK1 | Colorectal cancer || id:ieu-b-4965 | ENSG00000100151 || id:eqtl-a-ENSG00000100151 | MR Egger | 2.813266957 | 0.589544777 | -0.000284507 | 0.578598239 |
| PICK1 | Colorectal cancer || id:ieu-b-4965 | ENSG00000100151 || id:eqtl-a-ENSG00000100151 | Inverse variance weighted | 3.177737886 | 0.672605491 | NA | NA |
| CYB5R3 | Colorectal cancer || id:ebi-a-GCST90018808 | ENSG00000100243 || id:eqtl-a-ENSG00000100243 | Inverse variance weighted | 12.553287 | 0.128171948 | NA | NA |
| CYB5R3 | Colorectal cancer || id:ebi-a-GCST90018808 | ENSG00000100243 || id:eqtl-a-ENSG00000100243 | MR Egger | 11.5527651 | 0.116259225 | 0.007581536 | 0.461713899 |
| CYB5R3 | Colorectal cancer || id:ebi-a-GCST012879 | ENSG00000100243 || id:eqtl-a-ENSG00000100243 | MR Egger | 4.987548014 | 0.661482885 | -0.017424381 | 0.127982099 |
| CYB5R3 | Colorectal cancer || id:ebi-a-GCST012879 | ENSG00000100243 || id:eqtl-a-ENSG00000100243 | Inverse variance weighted | 7.966838798 | 0.436716115 | NA | NA |
| TSPO | Colorectal cancer (all cancers excluded) || id:finn-b-C3_COLORECTAL_EXALLC | ENSG00000100300 || id:eqtl-a-ENSG00000100300 | Inverse variance weighted | 0.222331984 | 0.973906096 | NA | NA |
| TSPO | Colorectal cancer (all cancers excluded) || id:finn-b-C3_COLORECTAL_EXALLC | ENSG00000100300 || id:eqtl-a-ENSG00000100300 | MR Egger | 0.221127474 | 0.895329263 | -0.000887854 | 0.975466496 |
| ALKBH1 | Colorectal cancer || id:ebi-a-GCST90018808 | ENSG00000100601 || id:eqtl-a-ENSG00000100601 | MR Egger | 0.613290875 | 0.433551421 | -0.010395814 | 0.573219043 |
| ALKBH1 | Colorectal cancer || id:ebi-a-GCST90018808 | ENSG00000100601 || id:eqtl-a-ENSG00000100601 | Inverse variance weighted | 1.241953661 | 0.537419214 | NA | NA |
| APEX1 | Colorectal cancer (all cancers excluded) || id:finn-b-C3_COLORECTAL_EXALLC | ENSG00000100823 || id:eqtl-a-ENSG00000100823 | Inverse variance weighted | 0.024445172 | 0.987851806 | NA | NA |
| APEX1 | Colorectal cancer (all cancers excluded) || id:finn-b-C3_COLORECTAL_EXALLC | ENSG00000100823 || id:eqtl-a-ENSG00000100823 | MR Egger | 0.011052326 | 0.91627262 | -0.014084817 | 0.926651894 |
| APEX1 | Colorectal cancer || id:finn-b-C3_COLORECTAL | ENSG00000100823 || id:eqtl-a-ENSG00000100823 | Inverse variance weighted | 0.011516262 | 0.994258415 | NA | NA |
| APEX1 | Colorectal cancer || id:finn-b-C3_COLORECTAL | ENSG00000100823 || id:eqtl-a-ENSG00000100823 | MR Egger | 0.000922405 | 0.975771078 | 0.0124172 | 0.934704906 |
| PCK2 | Colorectal cancer (all cancers excluded) || id:finn-b-C3_COLORECTAL_EXALLC | ENSG00000100889 || id:eqtl-a-ENSG00000100889 | Inverse variance weighted | 0.501368173 | 0.778268198 | NA | NA |
| PCK2 | Colorectal cancer (all cancers excluded) || id:finn-b-C3_COLORECTAL_EXALLC | ENSG00000100889 || id:eqtl-a-ENSG00000100889 | MR Egger | 0.256122537 | 0.612796626 | 0.018563632 | 0.707270412 |
| PCK2 | Colorectal cancer || id:finn-b-C3_COLORECTAL | ENSG00000100889 || id:eqtl-a-ENSG00000100889 | Inverse variance weighted | 0.51332783 | 0.773628175 | NA | NA |
| PCK2 | Colorectal cancer || id:finn-b-C3_COLORECTAL | ENSG00000100889 || id:eqtl-a-ENSG00000100889 | MR Egger | 0.211472262 | 0.645615899 | 0.020373415 | 0.680166636 |
| UQCC1 | Colorectal cancer || id:ebi-a-GCST012876 | ENSG00000101019 || id:eqtl-a-ENSG00000101019 | MR Egger | 0.318286096 | 0.572639464 | -0.011654622 | 0.906003841 |
| UQCC1 | Colorectal cancer || id:ebi-a-GCST012876 | ENSG00000101019 || id:eqtl-a-ENSG00000101019 | Inverse variance weighted | 0.34040706 | 0.843493123 | NA | NA |
| NDUFAF5 | Colorectal cancer || id:ebi-a-GCST90018588 | ENSG00000101247 || id:eqtl-a-ENSG00000101247 | Inverse variance weighted | 0.107076184 | 0.743497733 | NA | NA |
| VWA8 | Colorectal cancer || id:ebi-a-GCST012880 | ENSG00000102763 || id:eqtl-a-ENSG00000102763 | Inverse variance weighted | 0.583305586 | 0.445019546 | NA | NA |
| VWA8 | Colorectal cancer || id:ebi-a-GCST012876 | ENSG00000102763 || id:eqtl-a-ENSG00000102763 | Inverse variance weighted | 3.158025337 | 0.075554522 | NA | NA |
| MLYCD | Colorectal cancer || id:ebi-a-GCST012880 | ENSG00000103150 || id:eqtl-a-ENSG00000103150 | Inverse variance weighted | 2.628533244 | 0.104958633 | NA | NA |
| BCKDK | Colorectal cancer || id:ebi-a-GCST90018808 | ENSG00000103507 || id:eqtl-a-ENSG00000103507 | MR Egger | 2.975699659 | 0.225857766 | 0.007200362 | 0.782384704 |
| BCKDK | Colorectal cancer || id:ebi-a-GCST90018808 | ENSG00000103507 || id:eqtl-a-ENSG00000103507 | Inverse variance weighted | 3.123623265 | 0.372954839 | NA | NA |
| BCKDK | Colorectal cancer (Firth correction) || id:ebi-a-GCST90013862 | ENSG00000103507 || id:eqtl-a-ENSG00000103507 | Inverse variance weighted | 1.202839197 | 0.272754952 | NA | NA |
| BCKDK | Colorectal cancer (SPA correction) || id:ebi-a-GCST90013866 | ENSG00000103507 || id:eqtl-a-ENSG00000103507 | Inverse variance weighted | 1.202839197 | 0.272754952 | NA | NA |
| BCKDK | Colorectal cancer || id:ebi-a-GCST90018588 | ENSG00000103507 || id:eqtl-a-ENSG00000103507 | Inverse variance weighted | 2.66159985 | 0.44679249 | NA | NA |
| BCKDK | Colorectal cancer || id:ebi-a-GCST90018588 | ENSG00000103507 || id:eqtl-a-ENSG00000103507 | MR Egger | 1.879784722 | 0.390669884 | 0.026659032 | 0.469863261 |
| MTFMT | Colorectal cancer || id:ebi-a-GCST012879 | ENSG00000103707 || id:eqtl-a-ENSG00000103707 | MR Egger | 0.598087625 | 0.439308615 | 0.005816579 | 0.946202991 |
| MTFMT | Colorectal cancer || id:ebi-a-GCST012877 | ENSG00000103707 || id:eqtl-a-ENSG00000103707 | Inverse variance weighted | 1.995343781 | 0.368736902 | NA | NA |
| MTFMT | Colorectal cancer || id:ebi-a-GCST012879 | ENSG00000103707 || id:eqtl-a-ENSG00000103707 | Inverse variance weighted | 0.605262709 | 0.738871428 | NA | NA |
| MTFMT | Colorectal cancer || id:ebi-a-GCST012877 | ENSG00000103707 || id:eqtl-a-ENSG00000103707 | MR Egger | 1.047147738 | 0.306164904 | -0.083615525 | 0.515791879 |
| DMAC2 | Colorectal cancer || id:ieu-b-4965 | ENSG00000105341 || id:eqtl-a-ENSG00000105341 | Inverse variance weighted | 0.006675567 | 0.934881985 | NA | NA |
| DMAC2 | Colorectal cancer || id:ebi-a-GCST90018808 | ENSG00000105341 || id:eqtl-a-ENSG00000105341 | Inverse variance weighted | 0.008511694 | 0.926492389 | NA | NA |
| DMAC2 | Colorectal cancer || id:ebi-a-GCST012877 | ENSG00000105341 || id:eqtl-a-ENSG00000105341 | Inverse variance weighted | 0.491026134 | 0.483469926 | NA | NA |
| DMAC2 | Colorectal cancer || id:ieu-b-4965 | ENSG00000105341 || id:eqtl-a-ENSG00000105341 | Inverse variance weighted | 0.006675567 | 0.934881985 | NA | NA |
| BCAT2 | Colorectal cancer (SPA correction) || id:ebi-a-GCST90013866 | ENSG00000105552 || id:eqtl-a-ENSG00000105552 | MR Egger | 0.158319045 | 0.69070887 | -0.026857427 | 0.614341445 |
| BCAT2 | Colorectal cancer (SPA correction) || id:ebi-a-GCST90013866 | ENSG00000105552 || id:eqtl-a-ENSG00000105552 | Inverse variance weighted | 0.638113848 | 0.726834174 | NA | NA |
| BCAT2 | Colorectal cancer (Firth correction) || id:ebi-a-GCST90013862 | ENSG00000105552 || id:eqtl-a-ENSG00000105552 | Inverse variance weighted | 0.636437065 | 0.727443801 | NA | NA |
| BCAT2 | Colorectal cancer (Firth correction) || id:ebi-a-GCST90013862 | ENSG00000105552 || id:eqtl-a-ENSG00000105552 | MR Egger | 0.157956993 | 0.691044472 | -0.026818239 | 0.614750186 |
| GCDH | Colorectal cancer || id:ebi-a-GCST90018808 | ENSG00000105607 || id:eqtl-a-ENSG00000105607 | Inverse variance weighted | 2.86240387 | 0.413329886 | NA | NA |
| GCDH | Colorectal cancer || id:finn-b-C3_COLORECTAL | ENSG00000105607 || id:eqtl-a-ENSG00000105607 | Inverse variance weighted | 1.044192875 | 0.593275478 | NA | NA |
| GCDH | Colorectal cancer (all cancers excluded) || id:finn-b-C3_COLORECTAL_EXALLC | ENSG00000105607 || id:eqtl-a-ENSG00000105607 | Inverse variance weighted | 1.683024371 | 0.43105819 | NA | NA |
| GCDH | Colorectal cancer || id:ebi-a-GCST90018808 | ENSG00000105607 || id:eqtl-a-ENSG00000105607 | MR Egger | 1.93470096 | 0.380088758 | 0.018901084 | 0.437087416 |
| GCDH | Colorectal cancer (all cancers excluded) || id:finn-b-C3_COLORECTAL_EXALLC | ENSG00000105607 || id:eqtl-a-ENSG00000105607 | MR Egger | 1.652614933 | 0.198603398 | 0.00716069 | 0.914166722 |
| GCDH | Colorectal cancer || id:finn-b-C3_COLORECTAL | ENSG00000105607 || id:eqtl-a-ENSG00000105607 | MR Egger | 1.040699809 | 0.307658771 | 0.002393444 | 0.963158645 |
| FKBP8 | Colorectal cancer || id:ebi-a-GCST012877 | ENSG00000105701 || id:eqtl-a-ENSG00000105701 | Inverse variance weighted | 1.164244016 | 0.558711517 | NA | NA |
| FKBP8 | Colorectal cancer || id:ebi-a-GCST012877 | ENSG00000105701 || id:eqtl-a-ENSG00000105701 | MR Egger | 1.017083839 | 0.313211731 | 0.007425538 | 0.768602507 |
| HIBADH | Colorectal cancer || id:ieu-b-4965 | ENSG00000106049 || id:eqtl-a-ENSG00000106049 | Inverse variance weighted | 11.96393999 | 0.007507713 | NA | NA |
| HIBADH | Colorectal cancer || id:ieu-b-4965 | ENSG00000106049 || id:eqtl-a-ENSG00000106049 | MR Egger | 0.625526063 | 0.731423216 | -0.00112051 | 0.078014569 |
| ABHD11 | Colorectal cancer || id:ebi-a-GCST012876 | ENSG00000106077 || id:eqtl-a-ENSG00000106077 | Inverse variance weighted | 1.373776418 | 0.241164101 | NA | NA |
| ABHD11 | Colorectal cancer || id:ebi-a-GCST012879 | ENSG00000106077 || id:eqtl-a-ENSG00000106077 | Inverse variance weighted | 3.415429783 | 0.064589593 | NA | NA |
| GARS1 | Colorectal cancer || id:ebi-a-GCST012880 | ENSG00000106105 || id:eqtl-a-ENSG00000106105 | Inverse variance weighted | 0.137094274 | 0.711186957 | NA | NA |
| CHCHD2 | Colorectal cancer || id:ebi-a-GCST012877 | ENSG00000106153 || id:eqtl-a-ENSG00000106153 | MR Egger | 3.821817535 | 0.430656951 | -0.022744165 | 0.561715744 |
| CHCHD2 | Colorectal cancer || id:ebi-a-GCST012877 | ENSG00000106153 || id:eqtl-a-ENSG00000106153 | Inverse variance weighted | 4.221223488 | 0.518024655 | NA | NA |
| COA1 | Colorectal cancer || id:ebi-a-GCST90018588 | ENSG00000106603 || id:eqtl-a-ENSG00000106603 | Inverse variance weighted | 0.150813985 | 0.697758695 | NA | NA |
| PHYH | Colorectal cancer || id:finn-b-C3_COLORECTAL | ENSG00000107537 || id:eqtl-a-ENSG00000107537 | Inverse variance weighted | 3.518330542 | 0.172188535 | NA | NA |
| PHYH | Colorectal cancer || id:finn-b-C3_COLORECTAL | ENSG00000107537 || id:eqtl-a-ENSG00000107537 | MR Egger | 0.104627838 | 0.746345179 | -0.073787627 | 0.315820818 |
| PHYH | Colorectal cancer (all cancers excluded) || id:finn-b-C3_COLORECTAL_EXALLC | ENSG00000107537 || id:eqtl-a-ENSG00000107537 | MR Egger | 0.184556703 | 0.667486177 | -0.075855011 | 0.311297729 |
| PHYH | Colorectal cancer (all cancers excluded) || id:finn-b-C3_COLORECTAL_EXALLC | ENSG00000107537 || id:eqtl-a-ENSG00000107537 | Inverse variance weighted | 3.716690487 | 0.155930444 | NA | NA |
| PITRM1 | Colorectal cancer (all cancers excluded) || id:finn-b-C3_COLORECTAL_EXALLC | ENSG00000107959 || id:eqtl-a-ENSG00000107959 | MR Egger | 1.334458889 | 0.720967188 | -0.021942726 | 0.49432704 |
| PITRM1 | Colorectal cancer || id:finn-b-C3_COLORECTAL | ENSG00000107959 || id:eqtl-a-ENSG00000107959 | Inverse variance weighted | 1.735578876 | 0.784244986 | NA | NA |
| PITRM1 | Colorectal cancer || id:finn-b-C3_COLORECTAL | ENSG00000107959 || id:eqtl-a-ENSG00000107959 | MR Egger | 1.310669617 | 0.726601043 | -0.018225174 | 0.560983619 |
| PITRM1 | Colorectal cancer (all cancers excluded) || id:finn-b-C3_COLORECTAL_EXALLC | ENSG00000107959 || id:eqtl-a-ENSG00000107959 | Inverse variance weighted | 1.936581175 | 0.747422137 | NA | NA |
| PPIF | Colorectal cancer || id:ebi-a-GCST012880 | ENSG00000108179 || id:eqtl-a-ENSG00000108179 | MR Egger | 2.258151108 | 0.520585415 | -0.009536 | 0.862032959 |
| PPIF | Colorectal cancer || id:ebi-a-GCST012880 | ENSG00000108179 || id:eqtl-a-ENSG00000108179 | Inverse variance weighted | 2.29393708 | 0.681873124 | NA | NA |
| MRPL27 | Colorectal cancer || id:ebi-a-GCST012877 | ENSG00000108826 || id:eqtl-a-ENSG00000108826 | Inverse variance weighted | 0.040816211 | 0.839893097 | NA | NA |
| HTATIP2 | Colorectal cancer (all cancers excluded) || id:finn-b-C3_COLORECTAL_EXALLC | ENSG00000109854 || id:eqtl-a-ENSG00000109854 | Inverse variance weighted | 0.780667129 | 0.376937186 | NA | NA |
| HTATIP2 | Colorectal cancer || id:finn-b-C3_COLORECTAL | ENSG00000109854 || id:eqtl-a-ENSG00000109854 | Inverse variance weighted | 0.815077537 | 0.366623321 | NA | NA |
| FOXRED1 | Colorectal cancer (Firth correction) || id:ebi-a-GCST90013862 | ENSG00000110074 || id:eqtl-a-ENSG00000110074 | Inverse variance weighted | 7.100397981 | 0.13067682 | NA | NA |
| FOXRED1 | Colorectal cancer || id:ebi-a-GCST012876 | ENSG00000110074 || id:eqtl-a-ENSG00000110074 | Inverse variance weighted | 5.815220407 | 0.213379242 | NA | NA |
| FOXRED1 | Colorectal cancer (SPA correction) || id:ebi-a-GCST90013866 | ENSG00000110074 || id:eqtl-a-ENSG00000110074 | MR Egger | 1.998018129 | 0.572818151 | -0.038753619 | 0.109312061 |
| FOXRED1 | Colorectal cancer (SPA correction) || id:ebi-a-GCST90013866 | ENSG00000110074 || id:eqtl-a-ENSG00000110074 | Inverse variance weighted | 7.088331197 | 0.131293307 | NA | NA |
| FOXRED1 | Colorectal cancer || id:ieu-b-4965 | ENSG00000110074 || id:eqtl-a-ENSG00000110074 | MR Egger | 1.132475144 | 0.769240798 | -0.000359651 | 0.196515921 |
| FOXRED1 | Colorectal cancer || id:ebi-a-GCST012876 | ENSG00000110074 || id:eqtl-a-ENSG00000110074 | MR Egger | 5.793934365 | 0.122077684 | -0.002099092 | 0.923014434 |
| FOXRED1 | Colorectal cancer || id:ieu-b-4965 | ENSG00000110074 || id:eqtl-a-ENSG00000110074 | Inverse variance weighted | 3.871223517 | 0.423714292 | NA | NA |
| FOXRED1 | Colorectal cancer (Firth correction) || id:ebi-a-GCST90013862 | ENSG00000110074 || id:eqtl-a-ENSG00000110074 | MR Egger | 1.984002168 | 0.575733746 | -0.038703499 | 0.108730414 |
| FOXRED1 | Colorectal cancer || id:ieu-b-4965 | ENSG00000110074 || id:eqtl-a-ENSG00000110074 | Inverse variance weighted | 3.871223517 | 0.423714292 | NA | NA |
| PTPMT1 | Colorectal cancer || id:ebi-a-GCST012880 | ENSG00000110536 || id:eqtl-a-ENSG00000110536 | Inverse variance weighted | 0.017393402 | 0.895076006 | NA | NA |
| CHPT1 | Colorectal cancer || id:ebi-a-GCST012879 | ENSG00000111666 || id:eqtl-a-ENSG00000111666 | Inverse variance weighted | 4.534558444 | 0.475272995 | NA | NA |
| CHPT1 | Colorectal cancer || id:ebi-a-GCST012879 | ENSG00000111666 || id:eqtl-a-ENSG00000111666 | MR Egger | 3.836807904 | 0.428541765 | 0.013104813 | 0.450529815 |
| MRPL2 | Colorectal cancer || id:ebi-a-GCST012880 | ENSG00000112651 || id:eqtl-a-ENSG00000112651 | Inverse variance weighted | 0.381244275 | 0.536938072 | NA | NA |
| NNT | Colorectal cancer || id:ebi-a-GCST012878 | ENSG00000112992 || id:eqtl-a-ENSG00000112992 | Inverse variance weighted | 2.098631482 | 0.71762381 | NA | NA |
| NNT | Colorectal cancer || id:ebi-a-GCST012878 | ENSG00000112992 || id:eqtl-a-ENSG00000112992 | MR Egger | 1.576569412 | 0.66471414 | -0.109811393 | 0.5221954 |
| EHHADH | Colorectal cancer || id:ebi-a-GCST012877 | ENSG00000113790 || id:eqtl-a-ENSG00000113790 | Inverse variance weighted | 0.462923479 | 0.496260842 | NA | NA |
| EHHADH | Colorectal cancer || id:ebi-a-GCST012876 | ENSG00000113790 || id:eqtl-a-ENSG00000113790 | Inverse variance weighted | 2.438705454 | 0.11837388 | NA | NA |
| EHHADH | Colorectal cancer || id:ebi-a-GCST90018588 | ENSG00000113790 || id:eqtl-a-ENSG00000113790 | Inverse variance weighted | 0.659346249 | 0.416790837 | NA | NA |
| OGG1 | Colorectal cancer || id:ebi-a-GCST012880 | ENSG00000114026 || id:eqtl-a-ENSG00000114026 | Inverse variance weighted | 3.677241 | 0.055160055 | NA | NA |
| OGG1 | Colorectal cancer || id:ebi-a-GCST012878 | ENSG00000114026 || id:eqtl-a-ENSG00000114026 | Inverse variance weighted | 3.00998067 | 0.082753278 | NA | NA |
| PCCB | Colorectal cancer || id:ebi-a-GCST90018588 | ENSG00000114054 || id:eqtl-a-ENSG00000114054 | Inverse variance weighted | 0.569058809 | 0.752368232 | NA | NA |
| PCCB | Colorectal cancer || id:ebi-a-GCST90018588 | ENSG00000114054 || id:eqtl-a-ENSG00000114054 | MR Egger | 0.469792021 | 0.493082864 | 0.021771631 | 0.805690174 |
| HTRA2 | Colorectal cancer || id:ieu-b-4965 | ENSG00000115317 || id:eqtl-a-ENSG00000115317 | Inverse variance weighted | 0.714561274 | 0.397933726 | NA | NA |
| HTRA2 | Colorectal cancer || id:ieu-b-4965 | ENSG00000115317 || id:eqtl-a-ENSG00000115317 | Inverse variance weighted | 0.714561274 | 0.397933726 | NA | NA |
| EFHD1 | Colorectal cancer || id:ebi-a-GCST012880 | ENSG00000115468 || id:eqtl-a-ENSG00000115468 | Inverse variance weighted | 0.039263672 | 0.842927213 | NA | NA |
| EFHD1 | Colorectal cancer || id:ebi-a-GCST012879 | ENSG00000115468 || id:eqtl-a-ENSG00000115468 | Inverse variance weighted | 0.031241542 | 0.859702589 | NA | NA |
| WARS2 | Colorectal cancer || id:finn-b-C3_COLORECTAL | ENSG00000116874 || id:eqtl-a-ENSG00000116874 | MR Egger | 4.598275226 | 0.331053095 | -0.007693518 | 0.820027313 |
| WARS2 | Colorectal cancer || id:finn-b-C3_COLORECTAL | ENSG00000116874 || id:eqtl-a-ENSG00000116874 | Inverse variance weighted | 4.666102417 | 0.457971584 | NA | NA |
| MRPS15 | Colorectal cancer || id:ebi-a-GCST012876 | ENSG00000116898 || id:eqtl-a-ENSG00000116898 | Inverse variance weighted | 0.396412749 | 0.528947702 | NA | NA |
| KMO | Colorectal cancer || id:finn-b-C3_COLORECTAL | ENSG00000117009 || id:eqtl-a-ENSG00000117009 | Inverse variance weighted | 2.746227864 | 0.43242843 | NA | NA |
| KMO | Colorectal cancer || id:finn-b-C3_COLORECTAL | ENSG00000117009 || id:eqtl-a-ENSG00000117009 | MR Egger | 1.609531301 | 0.447192714 | -0.025325499 | 0.398014516 |
| KMO | Colorectal cancer (all cancers excluded) || id:finn-b-C3_COLORECTAL_EXALLC | ENSG00000117009 || id:eqtl-a-ENSG00000117009 | Inverse variance weighted | 2.240740917 | 0.523968324 | NA | NA |
| KMO | Colorectal cancer (all cancers excluded) || id:finn-b-C3_COLORECTAL_EXALLC | ENSG00000117009 || id:eqtl-a-ENSG00000117009 | MR Egger | 1.346879896 | 0.509951351 | -0.022736709 | 0.444228638 |
| NSUN4 | Colorectal cancer (Firth correction) || id:ebi-a-GCST90013862 | ENSG00000117481 || id:eqtl-a-ENSG00000117481 | MR Egger | 0.205695165 | 0.995060205 | 0.030050823 | 0.625220723 |
| NSUN4 | Colorectal cancer || id:ieu-b-4965 | ENSG00000117481 || id:eqtl-a-ENSG00000117481 | Inverse variance weighted | 5.234959588 | 0.3878836 | NA | NA |
| NSUN4 | Colorectal cancer (Firth correction) || id:ebi-a-GCST90013862 | ENSG00000117481 || id:eqtl-a-ENSG00000117481 | Inverse variance weighted | 0.484833882 | 0.992668093 | NA | NA |
| NSUN4 | Colorectal cancer || id:ieu-b-4965 | ENSG00000117481 || id:eqtl-a-ENSG00000117481 | MR Egger | 4.871208881 | 0.300769357 | 0.000434425 | 0.613758244 |
| NSUN4 | Colorectal cancer || id:ebi-a-GCST90018808 | ENSG00000117481 || id:eqtl-a-ENSG00000117481 | Inverse variance weighted | 2.519339796 | 0.773579685 | NA | NA |
| NSUN4 | Colorectal cancer || id:ebi-a-GCST90018808 | ENSG00000117481 || id:eqtl-a-ENSG00000117481 | MR Egger | 2.406595038 | 0.661435763 | 0.015968811 | 0.753914058 |
| NSUN4 | Colorectal cancer (SPA correction) || id:ebi-a-GCST90013866 | ENSG00000117481 || id:eqtl-a-ENSG00000117481 | MR Egger | 0.206661922 | 0.995015255 | 0.029968672 | 0.626036278 |
| ABCD3 | Colorectal cancer || id:ebi-a-GCST012878 | ENSG00000117528 || id:eqtl-a-ENSG00000117528 | Inverse variance weighted | 2.289709951 | 0.318270071 | NA | NA |
| ABCD3 | Colorectal cancer || id:ebi-a-GCST012878 | ENSG00000117528 || id:eqtl-a-ENSG00000117528 | MR Egger | 0.345406954 | 0.556724729 | -0.082888761 | 0.396074449 |
| PRDX6 | Colorectal cancer || id:finn-b-C3_COLORECTAL | ENSG00000117592 || id:eqtl-a-ENSG00000117592 | MR Egger | 2.338331604 | 0.938771219 | -0.024258141 | 0.233991631 |
| PRDX6 | Colorectal cancer || id:finn-b-C3_COLORECTAL | ENSG00000117592 || id:eqtl-a-ENSG00000117592 | Inverse variance weighted | 4.034571524 | 0.853990894 | NA | NA |
| MTFR1L | Colorectal cancer || id:ebi-a-GCST012878 | ENSG00000117640 || id:eqtl-a-ENSG00000117640 | Inverse variance weighted | 0.313210379 | 0.575716818 | NA | NA |
| NDUFA8 | Colorectal cancer || id:ebi-a-GCST012877 | ENSG00000119421 || id:eqtl-a-ENSG00000119421 | Inverse variance weighted | 3.073583435 | 0.079573889 | NA | NA |
| HSDL2 | Colorectal cancer || id:ebi-a-GCST012877 | ENSG00000119471 || id:eqtl-a-ENSG00000119471 | Inverse variance weighted | 9.71464598 | 0.045518779 | NA | NA |
| HSDL2 | Colorectal cancer || id:ebi-a-GCST90018808 | ENSG00000119471 || id:eqtl-a-ENSG00000119471 | Inverse variance weighted | 9.189076201 | 0.05654337 | NA | NA |
| HSDL2 | Colorectal cancer || id:ebi-a-GCST90018808 | ENSG00000119471 || id:eqtl-a-ENSG00000119471 | MR Egger | 9.106671201 | 0.027905745 | 0.004523113 | 0.879607203 |
| HSDL2 | Colorectal cancer || id:ebi-a-GCST012877 | ENSG00000119471 || id:eqtl-a-ENSG00000119471 | MR Egger | 2.08453563 | 0.555047641 | -0.060149746 | 0.070019524 |
| MTRF1 | Colorectal cancer || id:finn-b-C3_COLORECTAL | ENSG00000120662 || id:eqtl-a-ENSG00000120662 | Inverse variance weighted | 0.908176068 | 0.340598825 | NA | NA |
| MTRF1 | Colorectal cancer (all cancers excluded) || id:finn-b-C3_COLORECTAL_EXALLC | ENSG00000120662 || id:eqtl-a-ENSG00000120662 | Inverse variance weighted | 1.318224266 | 0.250910988 | NA | NA |
| MTERF2 | Colorectal cancer || id:ebi-a-GCST012880 | ENSG00000120832 || id:eqtl-a-ENSG00000120832 | Inverse variance weighted | 0.772132928 | 0.379558022 | NA | NA |
| EPHX2 | Colorectal cancer || id:ieu-b-4965 | ENSG00000120915 || id:eqtl-a-ENSG00000120915 | Inverse variance weighted | 9.394564873 | 0.152573627 | NA | NA |
| EPHX2 | Colorectal cancer || id:finn-b-C3_COLORECTAL | ENSG00000120915 || id:eqtl-a-ENSG00000120915 | Inverse variance weighted | 11.93438495 | 0.063448706 | NA | NA |
| EPHX2 | Colorectal cancer (all cancers excluded) || id:finn-b-C3_COLORECTAL_EXALLC | ENSG00000120915 || id:eqtl-a-ENSG00000120915 | Inverse variance weighted | 13.5095185 | 0.035621682 | NA | NA |
| EPHX2 | Colorectal cancer || id:ieu-b-4965 | ENSG00000120915 || id:eqtl-a-ENSG00000120915 | Inverse variance weighted | 9.394564873 | 0.152573627 | NA | NA |
| EPHX2 | Colorectal cancer || id:finn-b-C3_COLORECTAL | ENSG00000120915 || id:eqtl-a-ENSG00000120915 | MR Egger | 9.330203901 | 0.096596403 | 0.022813684 | 0.290590606 |
| EPHX2 | Colorectal cancer (all cancers excluded) || id:finn-b-C3_COLORECTAL_EXALLC | ENSG00000120915 || id:eqtl-a-ENSG00000120915 | MR Egger | 9.757011407 | 0.082420372 | 0.027669835 | 0.224173296 |
| EPHX2 | Colorectal cancer || id:ieu-b-4965 | ENSG00000120915 || id:eqtl-a-ENSG00000120915 | MR Egger | 9.259798174 | 0.099139693 | 5.51E-05 | 0.798125094 |
| CEP89 | Colorectal cancer || id:ebi-a-GCST012878 | ENSG00000121289 || id:eqtl-a-ENSG00000121289 | Inverse variance weighted | 0.237737974 | 0.625844664 | NA | NA |
| ECHDC2 | Colorectal cancer || id:ebi-a-GCST90018808 | ENSG00000121310 || id:eqtl-a-ENSG00000121310 | MR Egger | 5.078389229 | 0.406388625 | 0.006056866 | 0.673189323 |
| ECHDC2 | Colorectal cancer || id:ebi-a-GCST012876 | ENSG00000121310 || id:eqtl-a-ENSG00000121310 | MR Egger | 6.208171055 | 0.286486794 | 0.019444497 | 0.366780885 |
| ECHDC2 | Colorectal cancer || id:ieu-b-4965 | ENSG00000121310 || id:eqtl-a-ENSG00000121310 | Inverse variance weighted | 11.70908754 | 0.068782407 | NA | NA |
| ECHDC2 | Colorectal cancer || id:ieu-b-4965 | ENSG00000121310 || id:eqtl-a-ENSG00000121310 | MR Egger | 10.05378732 | 0.073725473 | 0.000352508 | 0.40584313 |
| ECHDC2 | Colorectal cancer || id:ebi-a-GCST012876 | ENSG00000121310 || id:eqtl-a-ENSG00000121310 | Inverse variance weighted | 7.429826872 | 0.282917915 | NA | NA |
| ECHDC2 | Colorectal cancer || id:ebi-a-GCST90018808 | ENSG00000121310 || id:eqtl-a-ENSG00000121310 | Inverse variance weighted | 5.281846794 | 0.508205169 | NA | NA |
| ECHDC2 | Colorectal cancer || id:ieu-b-4965 | ENSG00000121310 || id:eqtl-a-ENSG00000121310 | Inverse variance weighted | 11.70908754 | 0.068782407 | NA | NA |
| SERAC1 | Colorectal cancer || id:ebi-a-GCST012879 | ENSG00000122335 || id:eqtl-a-ENSG00000122335 | Inverse variance weighted | 0.01866445 | 0.89133283 | NA | NA |
| PRXL2A | Colorectal cancer || id:ebi-a-GCST012880 | ENSG00000122378 || id:eqtl-a-ENSG00000122378 | Inverse variance weighted | 0.636275567 | 0.425062695 | NA | NA |
| CISD1 | Colorectal cancer || id:ebi-a-GCST90018588 | ENSG00000122873 || id:eqtl-a-ENSG00000122873 | Inverse variance weighted | 1.055796879 | 0.901220596 | NA | NA |
| CISD1 | Colorectal cancer || id:ebi-a-GCST90018588 | ENSG00000122873 || id:eqtl-a-ENSG00000122873 | MR Egger | 0.792927116 | 0.851158015 | 0.011234003 | 0.64351926 |
| CISD1 | Colorectal cancer || id:ebi-a-GCST90018808 | ENSG00000122873 || id:eqtl-a-ENSG00000122873 | Inverse variance weighted | 0.310484292 | 0.997441241 | NA | NA |
| CISD1 | Colorectal cancer || id:ebi-a-GCST90018808 | ENSG00000122873 || id:eqtl-a-ENSG00000122873 | MR Egger | 0.309980747 | 0.989160808 | -0.000319206 | 0.983171912 |
| SLC25A16 | Colorectal cancer || id:ebi-a-GCST012879 | ENSG00000122912 || id:eqtl-a-ENSG00000122912 | MR Egger | 2.307923612 | 0.315384798 | -0.001228933 | 0.97243339 |
| SLC25A16 | Colorectal cancer || id:ebi-a-GCST90018808 | ENSG00000122912 || id:eqtl-a-ENSG00000122912 | MR Egger | 0.590365787 | 0.74439543 | -0.004030321 | 0.870816999 |
| SLC25A16 | Colorectal cancer || id:ebi-a-GCST012879 | ENSG00000122912 || id:eqtl-a-ENSG00000122912 | Inverse variance weighted | 2.309678779 | 0.510669246 | NA | NA |
| SLC25A16 | Colorectal cancer || id:ebi-a-GCST90018808 | ENSG00000122912 || id:eqtl-a-ENSG00000122912 | Inverse variance weighted | 0.62430873 | 0.890845588 | NA | NA |
| ATPAF1 | Colorectal cancer || id:ebi-a-GCST012877 | ENSG00000123472 || id:eqtl-a-ENSG00000123472 | Inverse variance weighted | 1.666720589 | 0.434586491 | NA | NA |
| ATPAF1 | Colorectal cancer || id:ebi-a-GCST90018808 | ENSG00000123472 || id:eqtl-a-ENSG00000123472 | MR Egger | 0.21141353 | 0.645661742 | -0.005501567 | 0.818967026 |
| ATPAF1 | Colorectal cancer || id:ebi-a-GCST012877 | ENSG00000123472 || id:eqtl-a-ENSG00000123472 | MR Egger | 1.660462653 | 0.197540869 | -0.002284558 | 0.960966594 |
| ATPAF1 | Colorectal cancer || id:ebi-a-GCST90018808 | ENSG00000123472 || id:eqtl-a-ENSG00000123472 | Inverse variance weighted | 0.296845359 | 0.86206666 | NA | NA |
| MRS2 | Colorectal cancer || id:ebi-a-GCST90018588 | ENSG00000124532 || id:eqtl-a-ENSG00000124532 | Inverse variance weighted | 1.966799658 | 0.16078797 | NA | NA |
| GOT2 | Colorectal cancer || id:ebi-a-GCST90018808 | ENSG00000125166 || id:eqtl-a-ENSG00000125166 | Inverse variance weighted | 1.548689854 | 0.213328958 | NA | NA |
| GOT2 | Colorectal cancer || id:ebi-a-GCST90018588 | ENSG00000125166 || id:eqtl-a-ENSG00000125166 | Inverse variance weighted | 0.800196118 | 0.37103474 | NA | NA |
| GOT2 | Colorectal cancer (SPA correction) || id:ebi-a-GCST90013866 | ENSG00000125166 || id:eqtl-a-ENSG00000125166 | Inverse variance weighted | 0.309802851 | 0.577801189 | NA | NA |
| GOT2 | Colorectal cancer (Firth correction) || id:ebi-a-GCST90013862 | ENSG00000125166 || id:eqtl-a-ENSG00000125166 | Inverse variance weighted | 0.309802851 | 0.577801189 | NA | NA |
| GOT2 | Colorectal cancer (all cancers excluded) || id:finn-b-C3_COLORECTAL_EXALLC | ENSG00000125166 || id:eqtl-a-ENSG00000125166 | Inverse variance weighted | 0.158068818 | 0.690940768 | NA | NA |
| SLC25A19 | Colorectal cancer || id:ebi-a-GCST012879 | ENSG00000125454 || id:eqtl-a-ENSG00000125454 | Inverse variance weighted | 1.899677218 | 0.168114453 | NA | NA |
| PANK2 | Colorectal cancer || id:ebi-a-GCST012880 | ENSG00000125779 || id:eqtl-a-ENSG00000125779 | Inverse variance weighted | 0.033607848 | 0.854543463 | NA | NA |
| PRDX5 | Colorectal cancer || id:ebi-a-GCST012879 | ENSG00000126432 || id:eqtl-a-ENSG00000126432 | Inverse variance weighted | 6.732088155 | 0.750472206 | NA | NA |
| PRDX5 | Colorectal cancer || id:ebi-a-GCST012880 | ENSG00000126432 || id:eqtl-a-ENSG00000126432 | Inverse variance weighted | 12.98703954 | 0.224397354 | NA | NA |
| PRDX5 | Colorectal cancer || id:ebi-a-GCST012880 | ENSG00000126432 || id:eqtl-a-ENSG00000126432 | MR Egger | 12.85226551 | 0.169405829 | 0.007305984 | 0.765673622 |
| PRDX5 | Colorectal cancer || id:ebi-a-GCST012879 | ENSG00000126432 || id:eqtl-a-ENSG00000126432 | MR Egger | 6.696113307 | 0.668724497 | 0.00205883 | 0.853776176 |
| TRAP1 | Colorectal cancer || id:ebi-a-GCST012878 | ENSG00000126602 || id:eqtl-a-ENSG00000126602 | MR Egger | 0.425925293 | 0.513995149 | 0.060986629 | 0.730934514 |
| TRAP1 | Colorectal cancer || id:ebi-a-GCST012878 | ENSG00000126602 || id:eqtl-a-ENSG00000126602 | Inverse variance weighted | 0.628201514 | 0.730445427 | NA | NA |
| RHOT1 | Colorectal cancer || id:ebi-a-GCST012877 | ENSG00000126858 || id:eqtl-a-ENSG00000126858 | Inverse variance weighted | 2.304291533 | 0.315958069 | NA | NA |
| RHOT1 | Colorectal cancer || id:ebi-a-GCST012877 | ENSG00000126858 || id:eqtl-a-ENSG00000126858 | MR Egger | 0.811092964 | 0.367797313 | -0.081825823 | 0.436614625 |
| PNKD | Colorectal cancer || id:ebi-a-GCST012878 | ENSG00000127838 || id:eqtl-a-ENSG00000127838 | Inverse variance weighted | 6.143537626 | 0.803063056 | NA | NA |
| PNKD | Colorectal cancer || id:ebi-a-GCST012876 | ENSG00000127838 || id:eqtl-a-ENSG00000127838 | Inverse variance weighted | 15.26656315 | 0.122641443 | NA | NA |
| PNKD | Colorectal cancer || id:ebi-a-GCST012878 | ENSG00000127838 || id:eqtl-a-ENSG00000127838 | MR Egger | 5.7110405 | 0.768453897 | 0.025055323 | 0.527226295 |
| PNKD | Colorectal cancer || id:finn-b-C3_COLORECTAL | ENSG00000127838 || id:eqtl-a-ENSG00000127838 | Inverse variance weighted | 17.01869279 | 0.073951378 | NA | NA |
| PNKD | Colorectal cancer || id:ebi-a-GCST012879 | ENSG00000127838 || id:eqtl-a-ENSG00000127838 | MR Egger | 6.725610372 | 0.665662694 | 0.0052135 | 0.667555614 |
| PNKD | Colorectal cancer (all cancers excluded) || id:finn-b-C3_COLORECTAL_EXALLC | ENSG00000127838 || id:eqtl-a-ENSG00000127838 | MR Egger | 15.90589474 | 0.068872948 | -0.005083783 | 0.839190592 |
| PNKD | Colorectal cancer || id:ebi-a-GCST012876 | ENSG00000127838 || id:eqtl-a-ENSG00000127838 | MR Egger | 13.22671962 | 0.152612926 | -0.018014676 | 0.268955659 |
| PNKD | Colorectal cancer || id:finn-b-C3_COLORECTAL | ENSG00000127838 || id:eqtl-a-ENSG00000127838 | MR Egger | 17.00580614 | 0.04862513 | -0.002054776 | 0.935990358 |
| PNKD | Colorectal cancer || id:ebi-a-GCST012879 | ENSG00000127838 || id:eqtl-a-ENSG00000127838 | Inverse variance weighted | 6.922708393 | 0.732721057 | NA | NA |
| PNKD | Colorectal cancer (all cancers excluded) || id:finn-b-C3_COLORECTAL_EXALLC | ENSG00000127838 || id:eqtl-a-ENSG00000127838 | Inverse variance weighted | 15.98300681 | 0.100119884 | NA | NA |
| ECHS1 | Colorectal cancer || id:ebi-a-GCST012878 | ENSG00000127884 || id:eqtl-a-ENSG00000127884 | Inverse variance weighted | 0.799322196 | 0.371296099 | NA | NA |
| PAICS | Colorectal cancer (Firth correction) || id:ebi-a-GCST90013862 | ENSG00000128050 || id:eqtl-a-ENSG00000128050 | Inverse variance weighted | 0.049995455 | 0.823071183 | NA | NA |
| PAICS | Colorectal cancer (SPA correction) || id:ebi-a-GCST90013866 | ENSG00000128050 || id:eqtl-a-ENSG00000128050 | Inverse variance weighted | 0.049995455 | 0.823071183 | NA | NA |
| MPST | Colorectal cancer (all cancers excluded) || id:finn-b-C3_COLORECTAL_EXALLC | ENSG00000128309 || id:eqtl-a-ENSG00000128309 | Inverse variance weighted | 0.174759689 | 0.675915906 | NA | NA |
| MTX2 | Colorectal cancer || id:ebi-a-GCST012879 | ENSG00000128654 || id:eqtl-a-ENSG00000128654 | Inverse variance weighted | 0.24240713 | 0.622472939 | NA | NA |
| IVD | Colorectal cancer || id:ieu-b-4965 | ENSG00000128928 || id:eqtl-a-ENSG00000128928 | MR Egger | 1.676910564 | 0.642079247 | -0.001101294 | 0.268836357 |
| IVD | Colorectal cancer || id:ieu-b-4965 | ENSG00000128928 || id:eqtl-a-ENSG00000128928 | Inverse variance weighted | 3.508960309 | 0.476517218 | NA | NA |
| IVD | Colorectal cancer || id:ebi-a-GCST90018808 | ENSG00000128928 || id:eqtl-a-ENSG00000128928 | MR Egger | 3.49865623 | 0.320936445 | -0.02865146 | 0.651113364 |
| IVD | Colorectal cancer || id:ebi-a-GCST90018808 | ENSG00000128928 || id:eqtl-a-ENSG00000128928 | Inverse variance weighted | 3.790834343 | 0.435052759 | NA | NA |
| DUT | Colorectal cancer || id:ebi-a-GCST012878 | ENSG00000128951 || id:eqtl-a-ENSG00000128951 | MR Egger | 6.698303154 | 0.035114133 | -0.053468567 | 0.934049581 |
| DUT | Colorectal cancer (Firth correction) || id:ebi-a-GCST90013862 | ENSG00000128951 || id:eqtl-a-ENSG00000128951 | MR Egger | 0.025597232 | 0.872887888 | 0.115179003 | 0.544216498 |
| DUT | Colorectal cancer (SPA correction) || id:ebi-a-GCST90013866 | ENSG00000128951 || id:eqtl-a-ENSG00000128951 | MR Egger | 0.023545912 | 0.878045962 | 0.113294928 | 0.548527868 |
| DUT | Colorectal cancer || id:ieu-b-4965 | ENSG00000128951 || id:eqtl-a-ENSG00000128951 | MR Egger | 3.061095287 | 0.216417115 | -0.000510598 | 0.827343155 |
| DUT | Colorectal cancer || id:ieu-b-4965 | ENSG00000128951 || id:eqtl-a-ENSG00000128951 | Inverse variance weighted | 3.155151574 | 0.3683169 | NA | NA |
| DUT | Colorectal cancer (Firth correction) || id:ebi-a-GCST90013862 | ENSG00000128951 || id:eqtl-a-ENSG00000128951 | Inverse variance weighted | 0.782350312 | 0.676261694 | NA | NA |
| DUT | Colorectal cancer (SPA correction) || id:ebi-a-GCST90013866 | ENSG00000128951 || id:eqtl-a-ENSG00000128951 | Inverse variance weighted | 0.759860634 | 0.683909065 | NA | NA |
| DUT | Colorectal cancer || id:ebi-a-GCST012878 | ENSG00000128951 || id:eqtl-a-ENSG00000128951 | Inverse variance weighted | 6.727564412 | 0.081107251 | NA | NA |
| DUT | Colorectal cancer || id:ieu-b-4965 | ENSG00000128951 || id:eqtl-a-ENSG00000128951 | Inverse variance weighted | 3.155151574 | 0.3683169 | NA | NA |
| ATP5IF1 | Colorectal cancer || id:ebi-a-GCST012878 | ENSG00000130770 || id:eqtl-a-ENSG00000130770 | Inverse variance weighted | 0.308493845 | 0.578605896 | NA | NA |
| COX4I1 | Colorectal cancer (SPA correction) || id:ebi-a-GCST90013866 | ENSG00000131143 || id:eqtl-a-ENSG00000131143 | Inverse variance weighted | 0.8733723 | 0.831848461 | NA | NA |
| COX4I1 | Colorectal cancer (SPA correction) || id:ebi-a-GCST90013866 | ENSG00000131143 || id:eqtl-a-ENSG00000131143 | MR Egger | 0.319400198 | 0.852399386 | 0.0207101 | 0.534268221 |
| COX4I1 | Colorectal cancer || id:ebi-a-GCST012879 | ENSG00000131143 || id:eqtl-a-ENSG00000131143 | MR Egger | 0.937480441 | 0.62579013 | 0.030877639 | 0.291887218 |
| COX4I1 | Colorectal cancer || id:ebi-a-GCST012879 | ENSG00000131143 || id:eqtl-a-ENSG00000131143 | Inverse variance weighted | 2.948902663 | 0.399570661 | NA | NA |
| COX4I1 | Colorectal cancer (Firth correction) || id:ebi-a-GCST90013862 | ENSG00000131143 || id:eqtl-a-ENSG00000131143 | Inverse variance weighted | 0.874604248 | 0.831551656 | NA | NA |
| COX4I1 | Colorectal cancer (Firth correction) || id:ebi-a-GCST90013862 | ENSG00000131143 || id:eqtl-a-ENSG00000131143 | MR Egger | 0.314148191 | 0.854640731 | 0.020863493 | 0.53214409 |
| MRPL35 | Colorectal cancer || id:finn-b-C3_COLORECTAL | ENSG00000132313 || id:eqtl-a-ENSG00000132313 | Inverse variance weighted | 0.924607981 | 0.336267969 | NA | NA |
| GRSF1 | Colorectal cancer || id:ebi-a-GCST012879 | ENSG00000132463 || id:eqtl-a-ENSG00000132463 | Inverse variance weighted | 1.246921935 | 0.264141186 | NA | NA |
| RIDA | Colorectal cancer || id:ebi-a-GCST012878 | ENSG00000132541 || id:eqtl-a-ENSG00000132541 | Inverse variance weighted | 1.327053715 | 0.249330042 | NA | NA |
| MUTYH | Colorectal cancer (Firth correction) || id:ebi-a-GCST90013862 | ENSG00000132781 || id:eqtl-a-ENSG00000132781 | Inverse variance weighted | 2.759907166 | 0.096653846 | NA | NA |
| MUTYH | Colorectal cancer || id:ieu-b-4965 | ENSG00000132781 || id:eqtl-a-ENSG00000132781 | Inverse variance weighted | 1.366783255 | 0.242365335 | NA | NA |
| MUTYH | Colorectal cancer || id:ebi-a-GCST90018808 | ENSG00000132781 || id:eqtl-a-ENSG00000132781 | Inverse variance weighted | 0.032195791 | 0.857598621 | NA | NA |
| MUTYH | Colorectal cancer (SPA correction) || id:ebi-a-GCST90013866 | ENSG00000132781 || id:eqtl-a-ENSG00000132781 | Inverse variance weighted | 2.763697683 | 0.096425131 | NA | NA |
| MUTYH | Colorectal cancer || id:ieu-b-4965 | ENSG00000132781 || id:eqtl-a-ENSG00000132781 | Inverse variance weighted | 1.366783255 | 0.242365335 | NA | NA |
| CASP9 | Colorectal cancer || id:ebi-a-GCST90018588 | ENSG00000132906 || id:eqtl-a-ENSG00000132906 | Inverse variance weighted | 0.000474296 | 0.982624774 | NA | NA |
| HSD17B4 | Colorectal cancer || id:ebi-a-GCST012878 | ENSG00000133835 || id:eqtl-a-ENSG00000133835 | MR Egger | 0.644931239 | 0.421930745 | -0.277337253 | 0.341489259 |
| HSD17B4 | Colorectal cancer || id:ebi-a-GCST012878 | ENSG00000133835 || id:eqtl-a-ENSG00000133835 | Inverse variance weighted | 3.473770554 | 0.17606795 | NA | NA |
| SPIRE1 | Colorectal cancer || id:ebi-a-GCST012879 | ENSG00000134278 || id:eqtl-a-ENSG00000134278 | MR Egger | 0.945792006 | 0.623194878 | 0.041747389 | 0.201923823 |
| SPIRE1 | Colorectal cancer || id:ebi-a-GCST012879 | ENSG00000134278 || id:eqtl-a-ENSG00000134278 | Inverse variance weighted | 4.454304616 | 0.216402607 | NA | NA |
| ECHDC3 | Colorectal cancer (SPA correction) || id:ebi-a-GCST90013866 | ENSG00000134463 || id:eqtl-a-ENSG00000134463 | MR Egger | 3.152021365 | 0.532714989 | 0.029983761 | 0.23925269 |
| ECHDC3 | Colorectal cancer (Firth correction) || id:ebi-a-GCST90013862 | ENSG00000134463 || id:eqtl-a-ENSG00000134463 | Inverse variance weighted | 5.05193132 | 0.409575378 | NA | NA |
| ECHDC3 | Colorectal cancer (SPA correction) || id:ebi-a-GCST90013866 | ENSG00000134463 || id:eqtl-a-ENSG00000134463 | Inverse variance weighted | 5.06079662 | 0.408505672 | NA | NA |
| ECHDC3 | Colorectal cancer (Firth correction) || id:ebi-a-GCST90013862 | ENSG00000134463 || id:eqtl-a-ENSG00000134463 | MR Egger | 3.152485649 | 0.532639333 | 0.029941824 | 0.240210573 |
| TIMM10 | Colorectal cancer || id:ebi-a-GCST012877 | ENSG00000134809 || id:eqtl-a-ENSG00000134809 | Inverse variance weighted | 4.721315043 | 0.317105461 | NA | NA |
| TIMM10 | Colorectal cancer || id:ebi-a-GCST012877 | ENSG00000134809 || id:eqtl-a-ENSG00000134809 | MR Egger | 3.746949297 | 0.290117413 | 0.107730231 | 0.442146496 |
| USP30 | Colorectal cancer || id:ebi-a-GCST90018588 | ENSG00000135093 || id:eqtl-a-ENSG00000135093 | Inverse variance weighted | 0.190145533 | 0.662795519 | NA | NA |
| PNPLA8 | Colorectal cancer || id:finn-b-C3_COLORECTAL | ENSG00000135241 || id:eqtl-a-ENSG00000135241 | Inverse variance weighted | 2.407278427 | 0.300100094 | NA | NA |
| PNPLA8 | Colorectal cancer || id:ebi-a-GCST012878 | ENSG00000135241 || id:eqtl-a-ENSG00000135241 | MR Egger | 3.404476787 | 0.065019731 | 0.14264174 | 0.702711363 |
| PNPLA8 | Colorectal cancer || id:ebi-a-GCST012878 | ENSG00000135241 || id:eqtl-a-ENSG00000135241 | Inverse variance weighted | 4.269859847 | 0.118252877 | NA | NA |
| PNPLA8 | Colorectal cancer || id:finn-b-C3_COLORECTAL | ENSG00000135241 || id:eqtl-a-ENSG00000135241 | MR Egger | 1.021938029 | 0.312059744 | 0.076800017 | 0.451763584 |
| PNPLA8 | Colorectal cancer (all cancers excluded) || id:finn-b-C3_COLORECTAL_EXALLC | ENSG00000135241 || id:eqtl-a-ENSG00000135241 | Inverse variance weighted | 1.862394354 | 0.394081642 | NA | NA |
| PNPLA8 | Colorectal cancer (all cancers excluded) || id:finn-b-C3_COLORECTAL_EXALLC | ENSG00000135241 || id:eqtl-a-ENSG00000135241 | MR Egger | 0.706465085 | 0.400619822 | 0.070807381 | 0.47695787 |
| GLS2 | Colorectal cancer || id:ebi-a-GCST90018588 | ENSG00000135423 || id:eqtl-a-ENSG00000135423 | Inverse variance weighted | 1.537267492 | 0.215024992 | NA | NA |
| CYP27A1 | Colorectal cancer || id:ebi-a-GCST012880 | ENSG00000135929 || id:eqtl-a-ENSG00000135929 | MR Egger | 19.92524983 | 0.399088776 | -0.000228497 | 0.986756659 |
| CYP27A1 | Colorectal cancer || id:ebi-a-GCST012880 | ENSG00000135929 || id:eqtl-a-ENSG00000135929 | Inverse variance weighted | 19.92554647 | 0.462595739 | NA | NA |
| CYP27A1 | Colorectal cancer || id:ebi-a-GCST90018808 | ENSG00000135929 || id:eqtl-a-ENSG00000135929 | MR Egger | 23.50234324 | 0.215939193 | -0.000114087 | 0.984302452 |
| CYP27A1 | Colorectal cancer || id:ebi-a-GCST90018808 | ENSG00000135929 || id:eqtl-a-ENSG00000135929 | Inverse variance weighted | 23.50283485 | 0.264783647 | NA | NA |
| MTHFS | Colorectal cancer || id:ieu-b-4965 | ENSG00000136371 || id:eqtl-a-ENSG00000136371 | Inverse variance weighted | 2.52796191 | 0.639635877 | NA | NA |
| MTHFS | Colorectal cancer || id:ebi-a-GCST90018808 | ENSG00000136371 || id:eqtl-a-ENSG00000136371 | MR Egger | 4.006640926 | 0.405107844 | 0.003850334 | 0.752272972 |
| MTHFS | Colorectal cancer || id:ieu-b-4965 | ENSG00000136371 || id:eqtl-a-ENSG00000136371 | MR Egger | 0.552219398 | 0.907278058 | -0.000338454 | 0.254503148 |
| MTHFS | Colorectal cancer || id:ebi-a-GCST012880 | ENSG00000136371 || id:eqtl-a-ENSG00000136371 | MR Egger | 1.983455508 | 0.575847667 | -0.006183515 | 0.834268084 |
| MTHFS | Colorectal cancer || id:ebi-a-GCST90018808 | ENSG00000136371 || id:eqtl-a-ENSG00000136371 | Inverse variance weighted | 4.121156926 | 0.532107593 | NA | NA |
| MTHFS | Colorectal cancer || id:ebi-a-GCST012880 | ENSG00000136371 || id:eqtl-a-ENSG00000136371 | Inverse variance weighted | 2.035461588 | 0.729236427 | NA | NA |
| MTHFS | Colorectal cancer || id:ieu-b-4965 | ENSG00000136371 || id:eqtl-a-ENSG00000136371 | Inverse variance weighted | 2.52796191 | 0.639635877 | NA | NA |
| BPHL | Colorectal cancer (all cancers excluded) || id:finn-b-C3_COLORECTAL_EXALLC | ENSG00000137274 || id:eqtl-a-ENSG00000137274 | Inverse variance weighted | 2.752687446 | 0.097091112 | NA | NA |
| BPHL | Colorectal cancer || id:finn-b-C3_COLORECTAL | ENSG00000137274 || id:eqtl-a-ENSG00000137274 | Inverse variance weighted | 2.241674168 | 0.134335464 | NA | NA |
| MTCH1 | Colorectal cancer || id:ebi-a-GCST012877 | ENSG00000137409 || id:eqtl-a-ENSG00000137409 | Inverse variance weighted | 0.369909762 | 0.946385825 | NA | NA |
| MTCH1 | Colorectal cancer || id:ebi-a-GCST012877 | ENSG00000137409 || id:eqtl-a-ENSG00000137409 | MR Egger | 0.126252583 | 0.938824901 | -0.01396155 | 0.670457558 |
| VARS2 | Colorectal cancer (Firth correction) || id:ebi-a-GCST90013862 | ENSG00000137411 || id:eqtl-a-ENSG00000137411 | MR Egger | 7.494184121 | 0.023586234 | -0.010403898 | 0.868054922 |
| VARS2 | Colorectal cancer (SPA correction) || id:ebi-a-GCST90013866 | ENSG00000137411 || id:eqtl-a-ENSG00000137411 | Inverse variance weighted | 7.591925621 | 0.055242943 | NA | NA |
| VARS2 | Colorectal cancer (Firth correction) || id:ebi-a-GCST90013862 | ENSG00000137411 || id:eqtl-a-ENSG00000137411 | Inverse variance weighted | 7.626965811 | 0.054384351 | NA | NA |
| VARS2 | Colorectal cancer (SPA correction) || id:ebi-a-GCST90013866 | ENSG00000137411 || id:eqtl-a-ENSG00000137411 | MR Egger | 7.472934467 | 0.023838169 | -0.009884444 | 0.87480662 |
| FDX1 | Colorectal cancer || id:ieu-b-4965 | ENSG00000137714 || id:eqtl-a-ENSG00000137714 | Inverse variance weighted | 1.935795056 | 0.164125829 | NA | NA |
| FDX1 | Colorectal cancer || id:ieu-b-4965 | ENSG00000137714 || id:eqtl-a-ENSG00000137714 | Inverse variance weighted | 1.935795056 | 0.164125829 | NA | NA |
| FDX1 | Colorectal cancer || id:ebi-a-GCST012879 | ENSG00000137714 || id:eqtl-a-ENSG00000137714 | Inverse variance weighted | 0.696780536 | 0.4038676 | NA | NA |
| KYAT3 | Colorectal cancer || id:ebi-a-GCST012876 | ENSG00000137944 || id:eqtl-a-ENSG00000137944 | Inverse variance weighted | 5.66738483 | 0.225403309 | NA | NA |
| KYAT3 | Colorectal cancer || id:ebi-a-GCST012876 | ENSG00000137944 || id:eqtl-a-ENSG00000137944 | MR Egger | 3.361938359 | 0.339114596 | -0.026560111 | 0.246957064 |
| PPA2 | Colorectal cancer || id:ebi-a-GCST012876 | ENSG00000138777 || id:eqtl-a-ENSG00000138777 | Inverse variance weighted | 3.441537766 | 0.063576493 | NA | NA |
| HADH | Colorectal cancer || id:ebi-a-GCST012878 | ENSG00000138796 || id:eqtl-a-ENSG00000138796 | MR Egger | 0.058454308 | 0.808955862 | 0.043895621 | 0.661268685 |
| HADH | Colorectal cancer || id:ebi-a-GCST012878 | ENSG00000138796 || id:eqtl-a-ENSG00000138796 | Inverse variance weighted | 0.405035736 | 0.81667189 | NA | NA |
| ETFA | Colorectal cancer || id:ieu-b-4965 | ENSG00000140374 || id:eqtl-a-ENSG00000140374 | Inverse variance weighted | 8.684654006 | 0.122322857 | NA | NA |
| ETFA | Colorectal cancer || id:ieu-b-4965 | ENSG00000140374 || id:eqtl-a-ENSG00000140374 | MR Egger | 8.363638175 | 0.079130023 | 0.00019521 | 0.715164602 |
| PIF1 | Colorectal cancer || id:ebi-a-GCST012878 | ENSG00000140451 || id:eqtl-a-ENSG00000140451 | Inverse variance weighted | 1.006823713 | 0.315664983 | NA | NA |
| PIF1 | Colorectal cancer || id:ebi-a-GCST012876 | ENSG00000140451 || id:eqtl-a-ENSG00000140451 | Inverse variance weighted | 0.001850066 | 0.965691657 | NA | NA |
| PTRH2 | Colorectal cancer || id:ieu-b-4965 | ENSG00000141378 || id:eqtl-a-ENSG00000141378 | Inverse variance weighted | 0.009911304 | 0.920697199 | NA | NA |
| PTRH2 | Colorectal cancer (SPA correction) || id:ebi-a-GCST90013866 | ENSG00000141378 || id:eqtl-a-ENSG00000141378 | Inverse variance weighted | 0.56799084 | 0.451058409 | NA | NA |
| PTRH2 | Colorectal cancer (Firth correction) || id:ebi-a-GCST90013862 | ENSG00000141378 || id:eqtl-a-ENSG00000141378 | Inverse variance weighted | 0.56799084 | 0.451058409 | NA | NA |
| PTRH2 | Colorectal cancer || id:ieu-b-4965 | ENSG00000141378 || id:eqtl-a-ENSG00000141378 | Inverse variance weighted | 0.009911304 | 0.920697199 | NA | NA |
| ALDH9A1 | Colorectal cancer || id:ieu-b-4965 | ENSG00000143149 || id:eqtl-a-ENSG00000143149 | MR Egger | 1.953799118 | 0.162178047 | -0.000110659 | 0.839851406 |
| ALDH9A1 | Colorectal cancer || id:ieu-b-4965 | ENSG00000143149 || id:eqtl-a-ENSG00000143149 | Inverse variance weighted | 2.082850791 | 0.352951228 | NA | NA |
| MPC2 | Colorectal cancer (Firth correction) || id:ebi-a-GCST90013862 | ENSG00000143158 || id:eqtl-a-ENSG00000143158 | Inverse variance weighted | 1.101415716 | 0.293955624 | NA | NA |
| MPC2 | Colorectal cancer (SPA correction) || id:ebi-a-GCST90013866 | ENSG00000143158 || id:eqtl-a-ENSG00000143158 | Inverse variance weighted | 1.101415716 | 0.293955624 | NA | NA |
| MGST3 | Colorectal cancer (SPA correction) || id:ebi-a-GCST90013866 | ENSG00000143198 || id:eqtl-a-ENSG00000143198 | Inverse variance weighted | 1.666753282 | 0.947659003 | NA | NA |
| MGST3 | Colorectal cancer (SPA correction) || id:ebi-a-GCST90013866 | ENSG00000143198 || id:eqtl-a-ENSG00000143198 | MR Egger | 1.663717749 | 0.893438626 | 0.001503127 | 0.958196005 |
| MGST3 | Colorectal cancer (Firth correction) || id:ebi-a-GCST90013862 | ENSG00000143198 || id:eqtl-a-ENSG00000143198 | Inverse variance weighted | 1.667535137 | 0.947599991 | NA | NA |
| MGST3 | Colorectal cancer (Firth correction) || id:ebi-a-GCST90013862 | ENSG00000143198 || id:eqtl-a-ENSG00000143198 | MR Egger | 1.664505963 | 0.893340711 | 0.001501599 | 0.958239765 |
| PPOX | Colorectal cancer || id:ebi-a-GCST90018808 | ENSG00000143224 || id:eqtl-a-ENSG00000143224 | Inverse variance weighted | 0.019686005 | 0.888417571 | NA | NA |
| PYCR2 | Colorectal cancer || id:ebi-a-GCST012876 | ENSG00000143811 || id:eqtl-a-ENSG00000143811 | MR Egger | 1.552077154 | 0.67030414 | -0.012367308 | 0.513430846 |
| PYCR2 | Colorectal cancer || id:ebi-a-GCST012876 | ENSG00000143811 || id:eqtl-a-ENSG00000143811 | Inverse variance weighted | 2.098323212 | 0.717680447 | NA | NA |
| SLC25A26 | Colorectal cancer || id:ebi-a-GCST012879 | ENSG00000144741 || id:eqtl-a-ENSG00000144741 | Inverse variance weighted | 0.968861752 | 0.324964219 | NA | NA |
| FARS2 | Colorectal cancer || id:ebi-a-GCST012879 | ENSG00000145982 || id:eqtl-a-ENSG00000145982 | Inverse variance weighted | 0.296661483 | 0.585982981 | NA | NA |
| PRSS35 | Colorectal cancer || id:ebi-a-GCST012880 | ENSG00000146250 || id:eqtl-a-ENSG00000146250 | Inverse variance weighted | 0.109096776 | 0.741174796 | NA | NA |
| SLC25A37 | Colorectal cancer || id:ebi-a-GCST90018808 | ENSG00000147454 || id:eqtl-a-ENSG00000147454 | Inverse variance weighted | 4.856811465 | 0.562305129 | NA | NA |
| SLC25A37 | Colorectal cancer || id:ebi-a-GCST90018808 | ENSG00000147454 || id:eqtl-a-ENSG00000147454 | MR Egger | 4.096407521 | 0.535620865 | 0.007029873 | 0.42309089 |
| PLPBP | Colorectal cancer || id:ebi-a-GCST012876 | ENSG00000147471 || id:eqtl-a-ENSG00000147471 | Inverse variance weighted | 0.036972057 | 0.847521949 | NA | NA |
| PLPBP | Colorectal cancer || id:ebi-a-GCST012880 | ENSG00000147471 || id:eqtl-a-ENSG00000147471 | Inverse variance weighted | 0.062449853 | 0.802664926 | NA | NA |
| ADHFE1 | Colorectal cancer || id:ebi-a-GCST012877 | ENSG00000147576 || id:eqtl-a-ENSG00000147576 | Inverse variance weighted | 3.64129978 | 0.302893088 | NA | NA |
| ADHFE1 | Colorectal cancer || id:ebi-a-GCST012877 | ENSG00000147576 || id:eqtl-a-ENSG00000147576 | MR Egger | 0.537695265 | 0.764259695 | -0.042414943 | 0.22017944 |
| LACTB2 | Colorectal cancer || id:ebi-a-GCST012878 | ENSG00000147592 || id:eqtl-a-ENSG00000147592 | Inverse variance weighted | 0.029163404 | 0.864402292 | NA | NA |
| AK3 | Colorectal cancer || id:ebi-a-GCST012879 | ENSG00000147853 || id:eqtl-a-ENSG00000147853 | MR Egger | 0.665600245 | 0.881263711 | 0.011209537 | 0.67471637 |
| AK3 | Colorectal cancer || id:ebi-a-GCST012879 | ENSG00000147853 || id:eqtl-a-ENSG00000147853 | Inverse variance weighted | 0.880211109 | 0.927382533 | NA | NA |
| MIGA2 | Colorectal cancer (all cancers excluded) || id:finn-b-C3_COLORECTAL_EXALLC | ENSG00000148343 || id:eqtl-a-ENSG00000148343 | Inverse variance weighted | 0.008532668 | 0.926402134 | NA | NA |
| MIGA2 | Colorectal cancer || id:finn-b-C3_COLORECTAL | ENSG00000148343 || id:eqtl-a-ENSG00000148343 | Inverse variance weighted | 0.015580248 | 0.900665369 | NA | NA |
| MSRB2 | Colorectal cancer (Firth correction) || id:ebi-a-GCST90013862 | ENSG00000148450 || id:eqtl-a-ENSG00000148450 | Inverse variance weighted | 14.21372872 | 0.114923015 | NA | NA |
| MSRB2 | Colorectal cancer || id:ebi-a-GCST012880 | ENSG00000148450 || id:eqtl-a-ENSG00000148450 | MR Egger | 10.23981508 | 0.41971153 | -0.024418381 | 0.168122024 |
| MSRB2 | Colorectal cancer || id:ebi-a-GCST90018808 | ENSG00000148450 || id:eqtl-a-ENSG00000148450 | MR Egger | 32.74647545 | 0.000300431 | -0.01176223 | 0.420151868 |
| MSRB2 | Colorectal cancer || id:ieu-b-4965 | ENSG00000148450 || id:eqtl-a-ENSG00000148450 | MR Egger | 14.73329025 | 0.064543705 | -0.000226636 | 0.291775492 |
| MSRB2 | Colorectal cancer || id:ieu-b-4965 | ENSG00000148450 || id:eqtl-a-ENSG00000148450 | Inverse variance weighted | 17.07904575 | 0.047492421 | NA | NA |
| MSRB2 | Colorectal cancer || id:ieu-b-4965 | ENSG00000148450 || id:eqtl-a-ENSG00000148450 | Inverse variance weighted | 17.07904575 | 0.047492421 | NA | NA |
| MSRB2 | Colorectal cancer (SPA correction) || id:ebi-a-GCST90013866 | ENSG00000148450 || id:eqtl-a-ENSG00000148450 | MR Egger | 10.48892162 | 0.232371585 | -0.02210535 | 0.136791112 |
| MSRB2 | Colorectal cancer || id:ebi-a-GCST012877 | ENSG00000148450 || id:eqtl-a-ENSG00000148450 | MR Egger | 16.8018032 | 0.078866235 | -0.013188963 | 0.344923998 |
| MSRB2 | Colorectal cancer (SPA correction) || id:ebi-a-GCST90013866 | ENSG00000148450 || id:eqtl-a-ENSG00000148450 | Inverse variance weighted | 14.07437616 | 0.119704012 | NA | NA |
| MSRB2 | Colorectal cancer (Firth correction) || id:ebi-a-GCST90013862 | ENSG00000148450 || id:eqtl-a-ENSG00000148450 | MR Egger | 10.53468334 | 0.229483204 | -0.022339561 | 0.133169821 |
| MSRB2 | Colorectal cancer || id:ebi-a-GCST012877 | ENSG00000148450 || id:eqtl-a-ENSG00000148450 | Inverse variance weighted | 18.45283884 | 0.071650625 | NA | NA |
| MSRB2 | Colorectal cancer || id:ebi-a-GCST012880 | ENSG00000148450 || id:eqtl-a-ENSG00000148450 | Inverse variance weighted | 12.50082105 | 0.327198082 | NA | NA |
| MSRB2 | Colorectal cancer || id:ebi-a-GCST90018808 | ENSG00000148450 || id:eqtl-a-ENSG00000148450 | Inverse variance weighted | 35.061038 | 0.000242122 | NA | NA |
| PDSS1 | Colorectal cancer || id:ebi-a-GCST90018588 | ENSG00000148459 || id:eqtl-a-ENSG00000148459 | Inverse variance weighted | 3.084082587 | 0.079061839 | NA | NA |
| GLUD1 | Colorectal cancer || id:ebi-a-GCST90018808 | ENSG00000148672 || id:eqtl-a-ENSG00000148672 | Inverse variance weighted | 0.270716242 | 0.602851715 | NA | NA |
| GLUD1 | Colorectal cancer || id:ebi-a-GCST012877 | ENSG00000148672 || id:eqtl-a-ENSG00000148672 | Inverse variance weighted | 0.321322354 | 0.57081404 | NA | NA |
| GLUD1 | Colorectal cancer || id:ebi-a-GCST012878 | ENSG00000148672 || id:eqtl-a-ENSG00000148672 | Inverse variance weighted | 0.095475747 | 0.757327993 | NA | NA |
| ME3 | Colorectal cancer || id:ebi-a-GCST90018588 | ENSG00000151376 || id:eqtl-a-ENSG00000151376 | Inverse variance weighted | 2.408857044 | 0.491988016 | NA | NA |
| ME3 | Colorectal cancer || id:ebi-a-GCST90018588 | ENSG00000151376 || id:eqtl-a-ENSG00000151376 | MR Egger | 2.252934086 | 0.324176537 | -0.00747897 | 0.745581013 |
| QDPR | Colorectal cancer || id:ebi-a-GCST90018808 | ENSG00000151552 || id:eqtl-a-ENSG00000151552 | MR Egger | 5.863463004 | 0.118445335 | 0.004883631 | 0.798995973 |
| QDPR | Colorectal cancer || id:ebi-a-GCST90018808 | ENSG00000151552 || id:eqtl-a-ENSG00000151552 | Inverse variance weighted | 6.014630418 | 0.198058326 | NA | NA |
| MMAA | Colorectal cancer (Firth correction) || id:ebi-a-GCST90013862 | ENSG00000151611 || id:eqtl-a-ENSG00000151611 | Inverse variance weighted | 0.045937231 | 0.830289939 | NA | NA |
| MMAA | Colorectal cancer (SPA correction) || id:ebi-a-GCST90013866 | ENSG00000151611 || id:eqtl-a-ENSG00000151611 | Inverse variance weighted | 0.045937231 | 0.830289939 | NA | NA |
| ACSL1 | Colorectal cancer (Firth correction) || id:ebi-a-GCST90013862 | ENSG00000151726 || id:eqtl-a-ENSG00000151726 | Inverse variance weighted | 7.935480848 | 0.047364112 | NA | NA |
| ACSL1 | Colorectal cancer (SPA correction) || id:ebi-a-GCST90013866 | ENSG00000151726 || id:eqtl-a-ENSG00000151726 | MR Egger | 5.513149834 | 0.06350892 | -0.085030163 | 0.446979166 |
| ACSL1 | Colorectal cancer (Firth correction) || id:ebi-a-GCST90013862 | ENSG00000151726 || id:eqtl-a-ENSG00000151726 | MR Egger | 5.503150061 | 0.063827252 | -0.085095326 | 0.446363525 |
| ACSL1 | Colorectal cancer (SPA correction) || id:ebi-a-GCST90013866 | ENSG00000151726 || id:eqtl-a-ENSG00000151726 | Inverse variance weighted | 7.942097835 | 0.047223649 | NA | NA |
| GUF1 | Colorectal cancer || id:ebi-a-GCST012877 | ENSG00000151806 || id:eqtl-a-ENSG00000151806 | Inverse variance weighted | 2.069872431 | 0.150234087 | NA | NA |
| PDK1 | Colorectal cancer || id:ebi-a-GCST012878 | ENSG00000152256 || id:eqtl-a-ENSG00000152256 | Inverse variance weighted | 0.718127725 | 0.868932068 | NA | NA |
| PDK1 | Colorectal cancer || id:ebi-a-GCST012878 | ENSG00000152256 || id:eqtl-a-ENSG00000152256 | MR Egger | 0.68773246 | 0.709023768 | -0.017313385 | 0.877647551 |
| TOMM70 | Colorectal cancer || id:ebi-a-GCST012880 | ENSG00000154174 || id:eqtl-a-ENSG00000154174 | Inverse variance weighted | 0.087415597 | 0.767488764 | NA | NA |
| ACSS1 | Colorectal cancer || id:ebi-a-GCST012877 | ENSG00000154930 || id:eqtl-a-ENSG00000154930 | Inverse variance weighted | 3.096513667 | 0.376982716 | NA | NA |
| ACSS1 | Colorectal cancer || id:ebi-a-GCST012877 | ENSG00000154930 || id:eqtl-a-ENSG00000154930 | MR Egger | 2.484587818 | 0.288721158 | 0.555457898 | 0.555457898 |
| AGPAT5 | Colorectal cancer || id:ieu-b-4965 | ENSG00000155189 || id:eqtl-a-ENSG00000155189 | Inverse variance weighted | 1.790108313 | 0.408585471 | NA | NA |
| AGPAT5 | Colorectal cancer || id:ieu-b-4965 | ENSG00000155189 || id:eqtl-a-ENSG00000155189 | MR Egger | 0.542082292 | 0.461571036 | 0.000560599 | 0.464809041 |
| ALAS2 | Colorectal cancer (all cancers excluded) || id:finn-b-C3_COLORECTAL_EXALLC | ENSG00000158578 || id:eqtl-a-ENSG00000158578 | MR Egger | 3.212544316 | 0.667255365 | 0.102884795 | 0.20503453 |
| ALAS2 | Colorectal cancer || id:ebi-a-GCST012876 | ENSG00000158578 || id:eqtl-a-ENSG00000158578 | MR Egger | 3.546984708 | 0.616290476 | 0.022554439 | 0.650840206 |
| ALAS2 | Colorectal cancer || id:finn-b-C3_COLORECTAL | ENSG00000158578 || id:eqtl-a-ENSG00000158578 | MR Egger | 3.311705997 | 0.652049982 | 0.098533865 | 0.217851449 |
| ALAS2 | Colorectal cancer (all cancers excluded) || id:finn-b-C3_COLORECTAL_EXALLC | ENSG00000158578 || id:eqtl-a-ENSG00000158578 | Inverse variance weighted | 5.33392378 | 0.501751993 | NA | NA |
| ALAS2 | Colorectal cancer || id:finn-b-C3_COLORECTAL | ENSG00000158578 || id:eqtl-a-ENSG00000158578 | Inverse variance weighted | 5.297319408 | 0.506283547 | NA | NA |
| ALAS2 | Colorectal cancer || id:ebi-a-GCST012876 | ENSG00000158578 || id:eqtl-a-ENSG00000158578 | Inverse variance weighted | 3.778326373 | 0.706645117 | NA | NA |
| PINK1 | Colorectal cancer || id:ebi-a-GCST012876 | ENSG00000158828 || id:eqtl-a-ENSG00000158828 | Inverse variance weighted | 0.724018794 | 0.696275825 | NA | NA |
| PINK1 | Colorectal cancer || id:ebi-a-GCST012876 | ENSG00000158828 || id:eqtl-a-ENSG00000158828 | MR Egger | 0.123145566 | 0.725647597 | -0.019009943 | 0.58020649 |
| TOMM40L | Colorectal cancer || id:ieu-b-4965 | ENSG00000158882 || id:eqtl-a-ENSG00000158882 | Inverse variance weighted | 3.786222349 | 0.285492775 | NA | NA |
| TOMM40L | Colorectal cancer || id:ieu-b-4965 | ENSG00000158882 || id:eqtl-a-ENSG00000158882 | MR Egger | 0.820907054 | 0.663349335 | -0.001342922 | 0.227209058 |
| MRPL10 | Colorectal cancer || id:ebi-a-GCST012878 | ENSG00000159111 || id:eqtl-a-ENSG00000159111 | Inverse variance weighted | 1.021115077 | 0.312254656 | NA | NA |
| CBR3 | Colorectal cancer || id:ebi-a-GCST90018588 | ENSG00000159231 || id:eqtl-a-ENSG00000159231 | Inverse variance weighted | 0.348209204 | 0.950720626 | NA | NA |
| CBR3 | Colorectal cancer || id:ebi-a-GCST90018808 | ENSG00000159231 || id:eqtl-a-ENSG00000159231 | MR Egger | 0.423135617 | 0.809314401 | -0.023566196 | 0.179606248 |
| CBR3 | Colorectal cancer || id:ebi-a-GCST90018588 | ENSG00000159231 || id:eqtl-a-ENSG00000159231 | MR Egger | 0.014560025 | 0.992746422 | -0.009758477 | 0.621882092 |
| CBR3 | Colorectal cancer || id:ebi-a-GCST90018808 | ENSG00000159231 || id:eqtl-a-ENSG00000159231 | Inverse variance weighted | 4.540202346 | 0.20873224 | NA | NA |
| ALDH4A1 | Colorectal cancer || id:ebi-a-GCST012880 | ENSG00000159423 || id:eqtl-a-ENSG00000159423 | Inverse variance weighted | 0.996955114 | 0.318048404 | NA | NA |
| AK4 | Colorectal cancer (all cancers excluded) || id:finn-b-C3_COLORECTAL_EXALLC | ENSG00000162433 || id:eqtl-a-ENSG00000162433 | Inverse variance weighted | 0.109889024 | 0.946537668 | NA | NA |
| AK4 | Colorectal cancer || id:finn-b-C3_COLORECTAL | ENSG00000162433 || id:eqtl-a-ENSG00000162433 | Inverse variance weighted | 0.14151349 | 0.931688503 | NA | NA |
| AK4 | Colorectal cancer || id:finn-b-C3_COLORECTAL | ENSG00000162433 || id:eqtl-a-ENSG00000162433 | MR Egger | 0.123270287 | 0.725514314 | -0.004812254 | 0.914530662 |
| AK4 | Colorectal cancer (all cancers excluded) || id:finn-b-C3_COLORECTAL_EXALLC | ENSG00000162433 || id:eqtl-a-ENSG00000162433 | MR Egger | 0.095665304 | 0.757094789 | -0.004281069 | 0.924431605 |
| COQ8A | Colorectal cancer || id:ebi-a-GCST90018588 | ENSG00000163050 || id:eqtl-a-ENSG00000163050 | Inverse variance weighted | 1.76474741 | 0.778925311 | NA | NA |
| COQ8A | Colorectal cancer || id:ebi-a-GCST90018808 | ENSG00000163050 || id:eqtl-a-ENSG00000163050 | Inverse variance weighted | 3.664254077 | 0.598692369 | NA | NA |
| COQ8A | Colorectal cancer || id:ebi-a-GCST90018808 | ENSG00000163050 || id:eqtl-a-ENSG00000163050 | MR Egger | 3.655105791 | 0.45468916 | -0.000920789 | 0.928401386 |
| COQ8A | Colorectal cancer || id:ebi-a-GCST90018588 | ENSG00000163050 || id:eqtl-a-ENSG00000163050 | MR Egger | 1.699132009 | 0.637126787 | -0.003483174 | 0.814391813 |
| BOLA3 | Colorectal cancer || id:finn-b-C3_COLORECTAL | ENSG00000163170 || id:eqtl-a-ENSG00000163170 | Inverse variance weighted | 0.158461514 | 0.690576931 | NA | NA |
| BOLA3 | Colorectal cancer (all cancers excluded) || id:finn-b-C3_COLORECTAL_EXALLC | ENSG00000163170 || id:eqtl-a-ENSG00000163170 | Inverse variance weighted | 0.046627737 | 0.829038753 | NA | NA |
| NAXE | Colorectal cancer (all cancers excluded) || id:finn-b-C3_COLORECTAL_EXALLC | ENSG00000163382 || id:eqtl-a-ENSG00000163382 | Inverse variance weighted | 1.455879187 | 0.227586925 | NA | NA |
| PPM1K | Colorectal cancer (all cancers excluded) || id:finn-b-C3_COLORECTAL_EXALLC | ENSG00000163644 || id:eqtl-a-ENSG00000163644 | Inverse variance weighted | 0.000139287 | 0.990583595 | NA | NA |
| PPM1K | Colorectal cancer || id:finn-b-C3_COLORECTAL | ENSG00000163644 || id:eqtl-a-ENSG00000163644 | Inverse variance weighted | 0.003292994 | 0.954238822 | NA | NA |
| PPM1K | Colorectal cancer || id:ebi-a-GCST012879 | ENSG00000163644 || id:eqtl-a-ENSG00000163644 | Inverse variance weighted | 0.565089097 | 0.452217005 | NA | NA |
| SLC25A46 | Colorectal cancer || id:ebi-a-GCST012880 | ENSG00000164209 || id:eqtl-a-ENSG00000164209 | MR Egger | 0.046206287 | 0.829801252 | 0.019511925 | 0.715417929 |
| SLC25A46 | Colorectal cancer || id:ebi-a-GCST012880 | ENSG00000164209 || id:eqtl-a-ENSG00000164209 | Inverse variance weighted | 0.2760168 | 0.871091375 | NA | NA |
| NDUFS4 | Colorectal cancer || id:ieu-b-4965 | ENSG00000164258 || id:eqtl-a-ENSG00000164258 | Inverse variance weighted | 0.504472441 | 0.477541535 | NA | NA |
| NDUFS4 | Colorectal cancer || id:ieu-b-4965 | ENSG00000164258 || id:eqtl-a-ENSG00000164258 | Inverse variance weighted | 0.504472441 | 0.477541535 | NA | NA |
| CASP3 | Colorectal cancer || id:ebi-a-GCST012876 | ENSG00000164305 || id:eqtl-a-ENSG00000164305 | Inverse variance weighted | 3.890123056 | 0.273575903 | NA | NA |
| CASP3 | Colorectal cancer || id:ebi-a-GCST012876 | ENSG00000164305 || id:eqtl-a-ENSG00000164305 | MR Egger | 3.164076836 | 0.205555662 | 0.048768166 | 0.56798337 |
| CASP3 | Colorectal cancer || id:finn-b-C3_COLORECTAL | ENSG00000164305 || id:eqtl-a-ENSG00000164305 | MR Egger | 2.45954353 | 0.292359297 | -0.059835641 | 0.48235976 |
| CASP3 | Colorectal cancer || id:finn-b-C3_COLORECTAL | ENSG00000164305 || id:eqtl-a-ENSG00000164305 | Inverse variance weighted | 3.359809157 | 0.339404697 | NA | NA |
| CASP3 | Colorectal cancer (all cancers excluded) || id:finn-b-C3_COLORECTAL_EXALLC | ENSG00000164305 || id:eqtl-a-ENSG00000164305 | Inverse variance weighted | 2.285237527 | 0.515354903 | NA | NA |
| CASP3 | Colorectal cancer (all cancers excluded) || id:finn-b-C3_COLORECTAL_EXALLC | ENSG00000164305 || id:eqtl-a-ENSG00000164305 | MR Egger | 1.936165891 | 0.379810458 | -0.037736089 | 0.614513521 |
| PRIMPOL | Colorectal cancer || id:ebi-a-GCST012877 | ENSG00000164306 || id:eqtl-a-ENSG00000164306 | Inverse variance weighted | 1.46E-05 | 0.996952448 | NA | NA |
| CCDC127 | Colorectal cancer || id:ieu-b-4965 | ENSG00000164366 || id:eqtl-a-ENSG00000164366 | MR Egger | 0.38862521 | 0.533022937 | -2.10E-05 | 0.954612826 |
| CCDC127 | Colorectal cancer || id:ieu-b-4965 | ENSG00000164366 || id:eqtl-a-ENSG00000164366 | Inverse variance weighted | 0.393725318 | 0.821303424 | NA | NA |
| ACSL6 | Colorectal cancer || id:ebi-a-GCST012880 | ENSG00000164398 || id:eqtl-a-ENSG00000164398 | Inverse variance weighted | 0.015724409 | 0.900209258 | NA | NA |
| PDSS2 | Colorectal cancer || id:ebi-a-GCST012880 | ENSG00000164494 || id:eqtl-a-ENSG00000164494 | Inverse variance weighted | 0.043345205 | 0.835076625 | NA | NA |
| OXR1 | Colorectal cancer || id:ebi-a-GCST012876 | ENSG00000164830 || id:eqtl-a-ENSG00000164830 | MR Egger | 1.152473221 | 0.283032016 | -0.045954823 | 0.372162626 |
| OXR1 | Colorectal cancer || id:ebi-a-GCST012876 | ENSG00000164830 || id:eqtl-a-ENSG00000164830 | Inverse variance weighted | 3.78420548 | 0.150754478 | NA | NA |
| TMEM65 | Colorectal cancer || id:ebi-a-GCST012879 | ENSG00000164983 || id:eqtl-a-ENSG00000164983 | Inverse variance weighted | 0.932917071 | 0.334106071 | NA | NA |
| LETM2 | Colorectal cancer || id:ebi-a-GCST012880 | ENSG00000165046 || id:eqtl-a-ENSG00000165046 | Inverse variance weighted | 1.859827531 | 0.932128126 | NA | NA |
| LETM2 | Colorectal cancer || id:ebi-a-GCST012880 | ENSG00000165046 || id:eqtl-a-ENSG00000165046 | MR Egger | 1.656050275 | 0.894389489 | -0.026266412 | 0.670594521 |
| MICU2 | Colorectal cancer || id:ebi-a-GCST012879 | ENSG00000165487 || id:eqtl-a-ENSG00000165487 | MR Egger | 0.275974077 | 0.871109983 | -0.013354395 | 0.64758633 |
| MICU2 | Colorectal cancer || id:ebi-a-GCST012879 | ENSG00000165487 || id:eqtl-a-ENSG00000165487 | Inverse variance weighted | 0.559588467 | 0.905618058 | NA | NA |
| MICU2 | Colorectal cancer || id:ebi-a-GCST012878 | ENSG00000165487 || id:eqtl-a-ENSG00000165487 | MR Egger | 0.619337725 | 0.733689868 | -0.061146492 | 0.5109871 |
| MICU2 | Colorectal cancer || id:ebi-a-GCST012878 | ENSG00000165487 || id:eqtl-a-ENSG00000165487 | Inverse variance weighted | 1.247920146 | 0.741535491 | NA | NA |
| IFI27 | Colorectal cancer || id:ebi-a-GCST012877 | ENSG00000165949 || id:eqtl-a-ENSG00000165949 | Inverse variance weighted | 2.456008002 | 0.652530183 | NA | NA |
| IFI27 | Colorectal cancer || id:ebi-a-GCST012877 | ENSG00000165949 || id:eqtl-a-ENSG00000165949 | MR Egger | 2.449112275 | 0.484557868 | 0.001777276 | 0.939049835 |
| IDH3A | Colorectal cancer || id:ebi-a-GCST90018808 | ENSG00000166411 || id:eqtl-a-ENSG00000166411 | Inverse variance weighted | 0.016484435 | 0.89783898 | NA | NA |
| IDH3A | Colorectal cancer || id:ieu-b-4965 | ENSG00000166411 || id:eqtl-a-ENSG00000166411 | Inverse variance weighted | 1.470504766 | 0.22526607 | NA | NA |
| IDH3A | Colorectal cancer || id:ieu-b-4965 | ENSG00000166411 || id:eqtl-a-ENSG00000166411 | Inverse variance weighted | 1.470504766 | 0.22526607 | NA | NA |
| LDHD | Colorectal cancer || id:ebi-a-GCST90018808 | ENSG00000166816 || id:eqtl-a-ENSG00000166816 | Inverse variance weighted | 2.483849071 | 0.47821699 | NA | NA |
| LDHD | Colorectal cancer || id:ebi-a-GCST90018808 | ENSG00000166816 || id:eqtl-a-ENSG00000166816 | MR Egger | 0.541846844 | 0.762674898 | -0.030825878 | 0.298114244 |
| ATP23 | Colorectal cancer (SPA correction) || id:ebi-a-GCST90013866 | ENSG00000166896 || id:eqtl-a-ENSG00000166896 | Inverse variance weighted | 3.379651823 | 0.066005998 | NA | NA |
| ATP23 | Colorectal cancer (Firth correction) || id:ebi-a-GCST90013862 | ENSG00000166896 || id:eqtl-a-ENSG00000166896 | Inverse variance weighted | 3.405896279 | 0.064963814 | NA | NA |
| ENDOG | Colorectal cancer || id:ebi-a-GCST012880 | ENSG00000167136 || id:eqtl-a-ENSG00000167136 | MR Egger | 0.679554555 | 0.711928868 | -0.026379059 | 0.512912461 |
| ENDOG | Colorectal cancer || id:ebi-a-GCST012880 | ENSG00000167136 || id:eqtl-a-ENSG00000167136 | Inverse variance weighted | 1.301660353 | 0.728738847 | NA | NA |
| GLOD4 | Colorectal cancer || id:ebi-a-GCST012876 | ENSG00000167699 || id:eqtl-a-ENSG00000167699 | Inverse variance weighted | 0.492058107 | 0.483010664 | NA | NA |
| ECI1 | Colorectal cancer || id:ieu-b-4965 | ENSG00000167969 || id:eqtl-a-ENSG00000167969 | Inverse variance weighted | 0.746357389 | 0.387631959 | NA | NA |
| ECI1 | Colorectal cancer || id:ieu-b-4965 | ENSG00000167969 || id:eqtl-a-ENSG00000167969 | Inverse variance weighted | 0.746357389 | 0.387631959 | NA | NA |
| ECI1 | Colorectal cancer || id:ebi-a-GCST012879 | ENSG00000167969 || id:eqtl-a-ENSG00000167969 | Inverse variance weighted | 0.77978338 | 0.377207398 | NA | NA |
| FTH1 | Colorectal cancer || id:ebi-a-GCST90018808 | ENSG00000167996 || id:eqtl-a-ENSG00000167996 | Inverse variance weighted | 1.848740517 | 0.173929951 | NA | NA |
| FTH1 | Colorectal cancer || id:ebi-a-GCST012878 | ENSG00000167996 || id:eqtl-a-ENSG00000167996 | Inverse variance weighted | 1.015942001 | 0.313483516 | NA | NA |
| DTYMK | Colorectal cancer || id:ebi-a-GCST012880 | ENSG00000168393 || id:eqtl-a-ENSG00000168393 | Inverse variance weighted | 0.025148675 | 0.873997158 | NA | NA |
| NDUFS5 | Colorectal cancer || id:finn-b-C3_COLORECTAL | ENSG00000168653 || id:eqtl-a-ENSG00000168653 | MR Egger | 2.844220729 | 0.416272037 | 0.020422785 | 0.54469166 |
| NDUFS5 | Colorectal cancer (all cancers excluded) || id:finn-b-C3_COLORECTAL_EXALLC | ENSG00000168653 || id:eqtl-a-ENSG00000168653 | Inverse variance weighted | 2.806201844 | 0.590762639 | NA | NA |
| NDUFS5 | Colorectal cancer || id:finn-b-C3_COLORECTAL | ENSG00000168653 || id:eqtl-a-ENSG00000168653 | Inverse variance weighted | 3.308066293 | 0.507655241 | NA | NA |
| NDUFS5 | Colorectal cancer (all cancers excluded) || id:finn-b-C3_COLORECTAL_EXALLC | ENSG00000168653 || id:eqtl-a-ENSG00000168653 | MR Egger | 2.173917016 | 0.537104161 | 0.024119867 | 0.484619157 |
| MFF | Colorectal cancer || id:ebi-a-GCST012877 | ENSG00000168958 || id:eqtl-a-ENSG00000168958 | Inverse variance weighted | 1.039470192 | 0.307944723 | NA | NA |
| MFF | Colorectal cancer || id:ebi-a-GCST012876 | ENSG00000168958 || id:eqtl-a-ENSG00000168958 | Inverse variance weighted | 2.275810359 | 0.131406424 | NA | NA |
| PRELID1 | Colorectal cancer || id:ebi-a-GCST90018808 | ENSG00000169230 || id:eqtl-a-ENSG00000169230 | Inverse variance weighted | 0.676880767 | 0.712881278 | NA | NA |
| PRELID1 | Colorectal cancer || id:ebi-a-GCST90018808 | ENSG00000169230 || id:eqtl-a-ENSG00000169230 | MR Egger | 0.11696514 | 0.732349638 | 0.048651712 | 0.59103784 |
| PRELID1 | Colorectal cancer (all cancers excluded) || id:finn-b-C3_COLORECTAL_EXALLC | ENSG00000169230 || id:eqtl-a-ENSG00000169230 | Inverse variance weighted | 0.225695733 | 0.634733909 | NA | NA |
| PUSL1 | Colorectal cancer || id:ebi-a-GCST90018588 | ENSG00000169972 || id:eqtl-a-ENSG00000169972 | Inverse variance weighted | 0.157722131 | 0.691262413 | NA | NA |
| PUSL1 | Colorectal cancer || id:ebi-a-GCST90018808 | ENSG00000169972 || id:eqtl-a-ENSG00000169972 | Inverse variance weighted | 1.536886279 | 0.215081872 | NA | NA |
| TRMT61B | Colorectal cancer || id:ebi-a-GCST012880 | ENSG00000171103 || id:eqtl-a-ENSG00000171103 | Inverse variance weighted | 0.081439174 | 0.775356478 | NA | NA |
| MFN1 | Colorectal cancer || id:ebi-a-GCST012877 | ENSG00000171109 || id:eqtl-a-ENSG00000171109 | MR Egger | 2.58238477 | 0.108058752 | 0.017110015 | 0.816044667 |
| MFN1 | Colorectal cancer || id:ebi-a-GCST012877 | ENSG00000171109 || id:eqtl-a-ENSG00000171109 | Inverse variance weighted | 2.81059878 | 0.245293607 | NA | NA |
| GATM | Colorectal cancer || id:ebi-a-GCST012878 | ENSG00000171766 || id:eqtl-a-ENSG00000171766 | Inverse variance weighted | 0.393421691 | 0.941598042 | NA | NA |
| GATM | Colorectal cancer || id:ebi-a-GCST012878 | ENSG00000171766 || id:eqtl-a-ENSG00000171766 | MR Egger | 0.392491899 | 0.821810086 | 0.002554244 | 0.978443563 |
| BCL2 | Colorectal cancer || id:ebi-a-GCST012879 | ENSG00000171791 || id:eqtl-a-ENSG00000171791 | Inverse variance weighted | 5.399950964 | 0.144746635 | NA | NA |
| BCL2 | Colorectal cancer || id:ebi-a-GCST012879 | ENSG00000171791 || id:eqtl-a-ENSG00000171791 | MR Egger | 0.59067292 | 0.744281124 | -0.138265009 | 0.159593488 |
| NME6 | Colorectal cancer || id:ebi-a-GCST012878 | ENSG00000172113 || id:eqtl-a-ENSG00000172113 | Inverse variance weighted | 0.000152319 | 0.990152946 | NA | NA |
| TEFM | Colorectal cancer || id:ebi-a-GCST90018588 | ENSG00000172171 || id:eqtl-a-ENSG00000172171 | Inverse variance weighted | 1.685264312 | 0.430575688 | NA | NA |
| TEFM | Colorectal cancer || id:ebi-a-GCST90018808 | ENSG00000172171 || id:eqtl-a-ENSG00000172171 | Inverse variance weighted | 4.035273591 | 0.401253136 | NA | NA |
| TEFM | Colorectal cancer || id:ebi-a-GCST90018588 | ENSG00000172171 || id:eqtl-a-ENSG00000172171 | MR Egger | 0.91093879 | 0.339865453 | 0.023130714 | 0.540595436 |
| TEFM | Colorectal cancer || id:ebi-a-GCST90018808 | ENSG00000172171 || id:eqtl-a-ENSG00000172171 | MR Egger | 3.994014563 | 0.262111175 | -0.003341081 | 0.871473917 |
| SUCLG2 | Colorectal cancer || id:ebi-a-GCST012876 | ENSG00000172340 || id:eqtl-a-ENSG00000172340 | MR Egger | 0.528851496 | 0.467090115 | -0.049547455 | 0.275846983 |
| SUCLG2 | Colorectal cancer || id:ebi-a-GCST012876 | ENSG00000172340 || id:eqtl-a-ENSG00000172340 | Inverse variance weighted | 5.201361701 | 0.074223026 | NA | NA |
| DCAKD | Colorectal cancer (all cancers excluded) || id:finn-b-C3_COLORECTAL_EXALLC | ENSG00000172992 || id:eqtl-a-ENSG00000172992 | Inverse variance weighted | 5.960864924 | 0.113529833 | NA | NA |
| DCAKD | Colorectal cancer (all cancers excluded) || id:finn-b-C3_COLORECTAL_EXALLC | ENSG00000172992 || id:eqtl-a-ENSG00000172992 | MR Egger | 4.990592803 | 0.082472003 | 0.02225974 | 0.596547755 |
| DCAKD | Colorectal cancer || id:finn-b-C3_COLORECTAL | ENSG00000172992 || id:eqtl-a-ENSG00000172992 | MR Egger | 4.604563923 | 0.100030318 | 0.019499818 | 0.62268611 |
| DCAKD | Colorectal cancer || id:finn-b-C3_COLORECTAL | ENSG00000172992 || id:eqtl-a-ENSG00000172992 | Inverse variance weighted | 5.368913424 | 0.14669269 | NA | NA |
| ATP5MD | Colorectal cancer || id:ebi-a-GCST90018808 | ENSG00000173915 || id:eqtl-a-ENSG00000173915 | Inverse variance weighted | 0.928629726 | 0.818513873 | NA | NA |
| ATP5MD | Colorectal cancer || id:ebi-a-GCST90018808 | ENSG00000173915 || id:eqtl-a-ENSG00000173915 | MR Egger | 0.68597405 | 0.709647419 | 0.006906206 | 0.671062091 |
| MSRB3 | Colorectal cancer || id:ebi-a-GCST012876 | ENSG00000174099 || id:eqtl-a-ENSG00000174099 | Inverse variance weighted | 0.35017804 | 0.839382296 | NA | NA |
| MSRB3 | Colorectal cancer || id:ebi-a-GCST012876 | ENSG00000174099 || id:eqtl-a-ENSG00000174099 | MR Egger | 0.166079831 | 0.683619549 | -0.136709609 | 0.741971573 |
| MSRB3 | Colorectal cancer || id:ebi-a-GCST90018588 | ENSG00000174099 || id:eqtl-a-ENSG00000174099 | Inverse variance weighted | 1.636582724 | 0.441184836 | NA | NA |
| MSRB3 | Colorectal cancer || id:ebi-a-GCST90018808 | ENSG00000174099 || id:eqtl-a-ENSG00000174099 | MR Egger | 1.127364702 | 0.56910954 | 0.028820451 | 0.532547682 |
| MSRB3 | Colorectal cancer || id:ebi-a-GCST90018588 | ENSG00000174099 || id:eqtl-a-ENSG00000174099 | MR Egger | 0.983133904 | 0.321426319 | -0.236954179 | 0.567213877 |
| MSRB3 | Colorectal cancer || id:ebi-a-GCST90018808 | ENSG00000174099 || id:eqtl-a-ENSG00000174099 | Inverse variance weighted | 1.686583978 | 0.639920592 | NA | NA |
| PCCA | Colorectal cancer || id:ebi-a-GCST012880 | ENSG00000175198 || id:eqtl-a-ENSG00000175198 | Inverse variance weighted | 0.57863366 | 0.446848381 | NA | NA |
| UCP2 | Colorectal cancer || id:ebi-a-GCST012879 | ENSG00000175567 || id:eqtl-a-ENSG00000175567 | MR Egger | 1.798373916 | 0.772779842 | 0.036809316 | 0.595788654 |
| UCP2 | Colorectal cancer || id:ebi-a-GCST012879 | ENSG00000175567 || id:eqtl-a-ENSG00000175567 | Inverse variance weighted | 2.129555897 | 0.830943673 | NA | NA |
| ACSF3 | Colorectal cancer (Firth correction) || id:ebi-a-GCST90013862 | ENSG00000176715 || id:eqtl-a-ENSG00000176715 | Inverse variance weighted | 0.218263584 | 0.640366177 | NA | NA |
| ACSF3 | Colorectal cancer (SPA correction) || id:ebi-a-GCST90013866 | ENSG00000176715 || id:eqtl-a-ENSG00000176715 | Inverse variance weighted | 0.218263584 | 0.640366177 | NA | NA |
| ACSF3 | Colorectal cancer || id:ebi-a-GCST012876 | ENSG00000176715 || id:eqtl-a-ENSG00000176715 | Inverse variance weighted | 0.231951045 | 0.630080916 | NA | NA |
| BOK | Colorectal cancer || id:finn-b-C3_COLORECTAL | ENSG00000176720 || id:eqtl-a-ENSG00000176720 | MR Egger | 0.612254069 | 0.433940374 | 0.083191047 | 0.300717291 |
| BOK | Colorectal cancer || id:finn-b-C3_COLORECTAL | ENSG00000176720 || id:eqtl-a-ENSG00000176720 | Inverse variance weighted | 4.442713335 | 0.108461862 | NA | NA |
| BOK | Colorectal cancer (all cancers excluded) || id:finn-b-C3_COLORECTAL_EXALLC | ENSG00000176720 || id:eqtl-a-ENSG00000176720 | MR Egger | 0.548418235 | 0.458964724 | 0.091850473 | 0.278700374 |
| BOK | Colorectal cancer (all cancers excluded) || id:finn-b-C3_COLORECTAL_EXALLC | ENSG00000176720 || id:eqtl-a-ENSG00000176720 | Inverse variance weighted | 5.112700342 | 0.077587405 | NA | NA |
| TIMM22 | Colorectal cancer || id:ebi-a-GCST012878 | ENSG00000177370 || id:eqtl-a-ENSG00000177370 | MR Egger | 0.291573804 | 0.58921376 | -0.073495784 | 0.498828575 |
| TIMM22 | Colorectal cancer || id:ebi-a-GCST012878 | ENSG00000177370 || id:eqtl-a-ENSG00000177370 | Inverse variance weighted | 1.298961252 | 0.522316984 | NA | NA |
| NDUFAF3 | Colorectal cancer || id:ebi-a-GCST90018588 | ENSG00000178057 || id:eqtl-a-ENSG00000178057 | Inverse variance weighted | 1.151513093 | 0.28323263 | NA | NA |
| COX14 | Colorectal cancer || id:ebi-a-GCST012876 | ENSG00000178449 || id:eqtl-a-ENSG00000178449 | MR Egger | 0.09325675 | 0.760076995 | 0.067826236 | 0.323715223 |
| COX14 | Colorectal cancer || id:ieu-b-4965 | ENSG00000178449 || id:eqtl-a-ENSG00000178449 | Inverse variance weighted | 0.090104841 | 0.764043914 | NA | NA |
| COX14 | Colorectal cancer (all cancers excluded) || id:finn-b-C3_COLORECTAL_EXALLC | ENSG00000178449 || id:eqtl-a-ENSG00000178449 | MR Egger | 1.16774431 | 0.279865209 | -0.017302286 | 0.809648308 |
| COX14 | Colorectal cancer || id:ebi-a-GCST012876 | ENSG00000178449 || id:eqtl-a-ENSG00000178449 | Inverse variance weighted | 3.312094296 | 0.190892059 | NA | NA |
| COX14 | Colorectal cancer (SPA correction) || id:ebi-a-GCST90013866 | ENSG00000178449 || id:eqtl-a-ENSG00000178449 | Inverse variance weighted | 0.463930315 | 0.792973752 | NA | NA |
| COX14 | Colorectal cancer (SPA correction) || id:ebi-a-GCST90013866 | ENSG00000178449 || id:eqtl-a-ENSG00000178449 | MR Egger | 0.414013727 | 0.519939294 | 0.01025249 | 0.86006444 |
| COX14 | Colorectal cancer || id:ebi-a-GCST90018808 | ENSG00000178449 || id:eqtl-a-ENSG00000178449 | Inverse variance weighted | 2.762910717 | 0.251212682 | NA | NA |
| COX14 | Colorectal cancer || id:finn-b-C3_COLORECTAL | ENSG00000178449 || id:eqtl-a-ENSG00000178449 | Inverse variance weighted | 1.252424144 | 0.534613046 | NA | NA |
| COX14 | Colorectal cancer || id:ebi-a-GCST012877 | ENSG00000178449 || id:eqtl-a-ENSG00000178449 | MR Egger | 0.011514284 | 0.91454731 | -0.080696265 | 0.290691964 |
| COX14 | Colorectal cancer (Firth correction) || id:ebi-a-GCST90013862 | ENSG00000178449 || id:eqtl-a-ENSG00000178449 | Inverse variance weighted | 0.461945712 | 0.793761012 | NA | NA |
| COX14 | Colorectal cancer (Firth correction) || id:ebi-a-GCST90013862 | ENSG00000178449 || id:eqtl-a-ENSG00000178449 | MR Egger | 0.41038497 | 0.521774174 | 0.589192983 | 0.589192983 |
| COX14 | Colorectal cancer || id:ebi-a-GCST90018808 | ENSG00000178449 || id:eqtl-a-ENSG00000178449 | MR Egger | 1.763504521 | 0.184187993 | 0.589192983 | 0.589192983 |
| COX14 | Colorectal cancer || id:finn-b-C3_COLORECTAL | ENSG00000178449 || id:eqtl-a-ENSG00000178449 | MR Egger | 1.171589695 | 0.279074842 | -0.014571617 | 0.836473158 |
| COX14 | Colorectal cancer (all cancers excluded) || id:finn-b-C3_COLORECTAL_EXALLC | ENSG00000178449 || id:eqtl-a-ENSG00000178449 | Inverse variance weighted | 1.278697507 | 0.527635933 | NA | NA |
| COX14 | Colorectal cancer || id:ebi-a-GCST012879 | ENSG00000178449 || id:eqtl-a-ENSG00000178449 | Inverse variance weighted | 0.97633554 | 0.613749896 | NA | NA |
| COX14 | Colorectal cancer || id:ebi-a-GCST012877 | ENSG00000178449 || id:eqtl-a-ENSG00000178449 | Inverse variance weighted | 4.155387285 | 0.125218678 | NA | NA |
| COX14 | Colorectal cancer || id:ebi-a-GCST012880 | ENSG00000178449 || id:eqtl-a-ENSG00000178449 | Inverse variance weighted | 1.15938589 | 0.560070312 | NA | NA |
| COX14 | Colorectal cancer || id:ieu-b-4965 | ENSG00000178449 || id:eqtl-a-ENSG00000178449 | Inverse variance weighted | 0.090104841 | 0.764043914 | NA | NA |
| COX14 | Colorectal cancer || id:ebi-a-GCST012880 | ENSG00000178449 || id:eqtl-a-ENSG00000178449 | MR Egger | 1.077734081 | 0.299205011 | 0.018517021 | 0.829004894 |
| COX14 | Colorectal cancer || id:ebi-a-GCST012879 | ENSG00000178449 || id:eqtl-a-ENSG00000178449 | MR Egger | 0.370516547 | 0.542722841 | 0.026230213 | 0.57894241 |
| NSUN3 | Colorectal cancer (all cancers excluded) || id:finn-b-C3_COLORECTAL_EXALLC | ENSG00000178694 || id:eqtl-a-ENSG00000178694 | MR Egger | 2.723371741 | 0.742541578 | -0.027190255 | 0.494089949 |
| NSUN3 | Colorectal cancer || id:finn-b-C3_COLORECTAL | ENSG00000178694 || id:eqtl-a-ENSG00000178694 | MR Egger | 2.336697715 | 0.800862231 | -0.029234128 | 0.45907468 |
| NSUN3 | Colorectal cancer || id:finn-b-C3_COLORECTAL | ENSG00000178694 || id:eqtl-a-ENSG00000178694 | Inverse variance weighted | 2.979544394 | 0.811409817 | NA | NA |
| NSUN3 | Colorectal cancer (all cancers excluded) || id:finn-b-C3_COLORECTAL_EXALLC | ENSG00000178694 || id:eqtl-a-ENSG00000178694 | Inverse variance weighted | 3.266911467 | 0.774677604 | NA | NA |
| TUFM | Colorectal cancer (Firth correction) || id:ebi-a-GCST90013862 | ENSG00000178952 || id:eqtl-a-ENSG00000178952 | MR Egger | 4.596495613 | 0.331258432 | -0.009574691 | 0.643946608 |
| TUFM | Colorectal cancer (Firth correction) || id:ebi-a-GCST90013862 | ENSG00000178952 || id:eqtl-a-ENSG00000178952 | Inverse variance weighted | 4.882678531 | 0.430365552 | NA | NA |
| TUFM | Colorectal cancer (SPA correction) || id:ebi-a-GCST90013866 | ENSG00000178952 || id:eqtl-a-ENSG00000178952 | MR Egger | 4.583994448 | 0.332703763 | -0.009641234 | 0.640974993 |
| TUFM | Colorectal cancer (SPA correction) || id:ebi-a-GCST90013866 | ENSG00000178952 || id:eqtl-a-ENSG00000178952 | Inverse variance weighted | 4.874701216 | 0.431362577 | NA | NA |
| D2HGDH | Colorectal cancer || id:ebi-a-GCST012876 | ENSG00000180902 || id:eqtl-a-ENSG00000180902 | MR Egger | 0.211971964 | 0.64522617 | -0.121171064 | 0.344136221 |
| D2HGDH | Colorectal cancer || id:ebi-a-GCST012879 | ENSG00000180902 || id:eqtl-a-ENSG00000180902 | MR Egger | 0.600108394 | 0.438536672 | 0.031283577 | 0.690978598 |
| D2HGDH | Colorectal cancer || id:ebi-a-GCST012876 | ENSG00000180902 || id:eqtl-a-ENSG00000180902 | Inverse variance weighted | 2.987881639 | 0.22448625 | NA | NA |
| D2HGDH | Colorectal cancer || id:ebi-a-GCST012879 | ENSG00000180902 || id:eqtl-a-ENSG00000180902 | Inverse variance weighted | 0.878371218 | 0.644561132 | NA | NA |
| SLC25A42 | Colorectal cancer || id:ieu-b-4965 | ENSG00000181035 || id:eqtl-a-ENSG00000181035 | Inverse variance weighted | 2.267491503 | 0.13211359 | NA | NA |
| SLC25A42 | Colorectal cancer || id:ebi-a-GCST90018808 | ENSG00000181035 || id:eqtl-a-ENSG00000181035 | Inverse variance weighted | 0.086143626 | 0.769138208 | NA | NA |
| SLC25A42 | Colorectal cancer || id:ieu-b-4965 | ENSG00000181035 || id:eqtl-a-ENSG00000181035 | Inverse variance weighted | 2.267491503 | 0.13211359 | NA | NA |
| HIGD1A | Colorectal cancer (all cancers excluded) || id:finn-b-C3_COLORECTAL_EXALLC | ENSG00000181061 || id:eqtl-a-ENSG00000181061 | Inverse variance weighted | 0.072275272 | 0.788052343 | NA | NA |
| HIGD1A | Colorectal cancer || id:finn-b-C3_COLORECTAL | ENSG00000181061 || id:eqtl-a-ENSG00000181061 | Inverse variance weighted | 0.005489445 | 0.940938143 | NA | NA |
| DHTKD1 | Colorectal cancer || id:ebi-a-GCST90018808 | ENSG00000181192 || id:eqtl-a-ENSG00000181192 | Inverse variance weighted | 2.602929471 | 0.106666387 | NA | NA |
| MRPS23 | Colorectal cancer || id:ebi-a-GCST012876 | ENSG00000181610 || id:eqtl-a-ENSG00000181610 | Inverse variance weighted | 0.189264488 | 0.663529484 | NA | NA |
| IDH2 | Colorectal cancer || id:ebi-a-GCST012877 | ENSG00000182054 || id:eqtl-a-ENSG00000182054 | MR Egger | 1.439708392 | 0.486823232 | 0.060587295 | 0.22153572 |
| IDH2 | Colorectal cancer || id:ebi-a-GCST012877 | ENSG00000182054 || id:eqtl-a-ENSG00000182054 | Inverse variance weighted | 4.515936011 | 0.210873229 | NA | NA |
| IDH2 | Colorectal cancer || id:ieu-b-4965 | ENSG00000182054 || id:eqtl-a-ENSG00000182054 | MR Egger | 1.616729113 | 0.445586202 | 0.000310727 | 0.582284058 |
| IDH2 | Colorectal cancer || id:ieu-b-4965 | ENSG00000182054 || id:eqtl-a-ENSG00000182054 | Inverse variance weighted | 2.039463887 | 0.564256361 | NA | NA |
| IDH2 | Colorectal cancer || id:ieu-b-4965 | ENSG00000182054 || id:eqtl-a-ENSG00000182054 | Inverse variance weighted | 2.039463887 | 0.564256361 | NA | NA |
| YBEY | Colorectal cancer (Firth correction) || id:ebi-a-GCST90013862 | ENSG00000182362 || id:eqtl-a-ENSG00000182362 | MR Egger | 7.446375789 | 0.383926835 | -0.027533561 | 0.65904383 |
| YBEY | Colorectal cancer || id:ieu-b-4965 | ENSG00000182362 || id:eqtl-a-ENSG00000182362 | Inverse variance weighted | 3.832706744 | 0.871892909 | NA | NA |
| YBEY | Colorectal cancer || id:ieu-b-4965 | ENSG00000182362 || id:eqtl-a-ENSG00000182362 | MR Egger | 3.724096281 | 0.810950976 | 0.751373764 | 0.751373764 |
| YBEY | Colorectal cancer (SPA correction) || id:ebi-a-GCST90013866 | ENSG00000182362 || id:eqtl-a-ENSG00000182362 | MR Egger | 7.446375789 | 0.383926835 | -0.027533561 | 0.65904383 |
| YBEY | Colorectal cancer (Firth correction) || id:ebi-a-GCST90013862 | ENSG00000182362 || id:eqtl-a-ENSG00000182362 | Inverse variance weighted | 7.672069824 | 0.466140748 | NA | NA |
| YBEY | Colorectal cancer || id:ebi-a-GCST90018808 | ENSG00000182362 || id:eqtl-a-ENSG00000182362 | Inverse variance weighted | 7.752224729 | 0.458040303 | NA | NA |
| YBEY | Colorectal cancer || id:ebi-a-GCST012880 | ENSG00000182362 || id:eqtl-a-ENSG00000182362 | Inverse variance weighted | 12.23057216 | 0.141209009 | NA | NA |
| YBEY | Colorectal cancer (SPA correction) || id:ebi-a-GCST90013866 | ENSG00000182362 || id:eqtl-a-ENSG00000182362 | Inverse variance weighted | 7.672069824 | 0.466140748 | NA | NA |
| YBEY | Colorectal cancer || id:ebi-a-GCST90018808 | ENSG00000182362 || id:eqtl-a-ENSG00000182362 | MR Egger | 6.46911687 | 0.486159852 | -0.038665978 | 0.294625203 |
| YBEY | Colorectal cancer || id:ieu-b-4965 | ENSG00000182362 || id:eqtl-a-ENSG00000182362 | Inverse variance weighted | 3.832706744 | 0.871892909 | NA | NA |
| YBEY | Colorectal cancer || id:ieu-b-4965 | ENSG00000182362 || id:eqtl-a-ENSG00000182362 | MR Egger | 3.724096281 | 0.810950976 | -0.000240776 | 0.751373764 |
| YBEY | Colorectal cancer || id:ebi-a-GCST012880 | ENSG00000182362 || id:eqtl-a-ENSG00000182362 | MR Egger | 12.16868421 | 0.095146284 | 0.021548093 | 0.855695879 |
| SERHL2 | Colorectal cancer (all cancers excluded) || id:finn-b-C3_COLORECTAL_EXALLC | ENSG00000183569 || id:eqtl-a-ENSG00000183569 | Inverse variance weighted | 0.510672341 | 0.47484795 | NA | NA |
| SFXN4 | Colorectal cancer || id:ebi-a-GCST012879 | ENSG00000183605 || id:eqtl-a-ENSG00000183605 | Inverse variance weighted | 4.262926645 | 0.371590472 | NA | NA |
| SFXN4 | Colorectal cancer || id:ebi-a-GCST012877 | ENSG00000183605 || id:eqtl-a-ENSG00000183605 | MR Egger | 0.906551462 | 0.823846509 | 0.041734568 | 0.473765576 |
| SFXN4 | Colorectal cancer || id:ebi-a-GCST012879 | ENSG00000183605 || id:eqtl-a-ENSG00000183605 | MR Egger | 2.664518208 | 0.446290768 | -0.055168939 | 0.295425756 |
| SFXN4 | Colorectal cancer || id:ebi-a-GCST012877 | ENSG00000183605 || id:eqtl-a-ENSG00000183605 | Inverse variance weighted | 1.574067498 | 0.813445274 | NA | NA |
| MRPL54 | Colorectal cancer || id:ebi-a-GCST012879 | ENSG00000183617 || id:eqtl-a-ENSG00000183617 | Inverse variance weighted | 0.001346247 | 0.970731192 | NA | NA |
| NDUFB1 | Colorectal cancer (SPA correction) || id:ebi-a-GCST90013866 | ENSG00000183648 || id:eqtl-a-ENSG00000183648 | Inverse variance weighted | 0.72709353 | 0.393826914 | NA | NA |
| NDUFB1 | Colorectal cancer (Firth correction) || id:ebi-a-GCST90013862 | ENSG00000183648 || id:eqtl-a-ENSG00000183648 | Inverse variance weighted | 0.72709353 | 0.393826914 | NA | NA |
| TOP1MT | Colorectal cancer || id:ieu-b-4965 | ENSG00000184428 || id:eqtl-a-ENSG00000184428 | Inverse variance weighted | 0.003049185 | 0.95596365 | NA | NA |
| TOP1MT | Colorectal cancer || id:ieu-b-4965 | ENSG00000184428 || id:eqtl-a-ENSG00000184428 | Inverse variance weighted | 0.003049185 | 0.95596365 | NA | NA |
| NAT8L | Colorectal cancer || id:ebi-a-GCST90018808 | ENSG00000185818 || id:eqtl-a-ENSG00000185818 | Inverse variance weighted | 0.116520893 | 0.732838931 | NA | NA |
| LYRM7 | Colorectal cancer || id:finn-b-C3_COLORECTAL | ENSG00000186687 || id:eqtl-a-ENSG00000186687 | Inverse variance weighted | 0.386603305 | 0.534090274 | NA | NA |
| LYRM7 | Colorectal cancer || id:ieu-b-4965 | ENSG00000186687 || id:eqtl-a-ENSG00000186687 | Inverse variance weighted | 0.728525172 | 0.393361656 | NA | NA |
| LYRM7 | Colorectal cancer || id:ieu-b-4965 | ENSG00000186687 || id:eqtl-a-ENSG00000186687 | Inverse variance weighted | 0.728525172 | 0.393361656 | NA | NA |
| LYRM7 | Colorectal cancer (all cancers excluded) || id:finn-b-C3_COLORECTAL_EXALLC | ENSG00000186687 || id:eqtl-a-ENSG00000186687 | Inverse variance weighted | 0.527639834 | 0.467600816 | NA | NA |
| CMC1 | Colorectal cancer || id:ebi-a-GCST90018808 | ENSG00000187118 || id:eqtl-a-ENSG00000187118 | Inverse variance weighted | 3.147408654 | 0.369451301 | NA | NA |
| CMC1 | Colorectal cancer || id:ieu-b-4965 | ENSG00000187118 || id:eqtl-a-ENSG00000187118 | MR Egger | 1.158640862 | 0.560278986 | -0.000460359 | 0.359720413 |
| CMC1 | Colorectal cancer || id:ieu-b-4965 | ENSG00000187118 || id:eqtl-a-ENSG00000187118 | Inverse variance weighted | 2.54822979 | 0.466637899 | NA | NA |
| CMC1 | Colorectal cancer || id:ieu-b-4965 | ENSG00000187118 || id:eqtl-a-ENSG00000187118 | Inverse variance weighted | 2.54822979 | 0.466637899 | NA | NA |
| C15orf61 | Colorectal cancer (Firth correction) || id:ebi-a-GCST90013862 | ENSG00000189227 || id:eqtl-a-ENSG00000189227 | Inverse variance weighted | 1.046620842 | 0.306286623 | NA | NA |
| C15orf61 | Colorectal cancer (SPA correction) || id:ebi-a-GCST90013866 | ENSG00000189227 || id:eqtl-a-ENSG00000189227 | Inverse variance weighted | 1.06847452 | 0.30129027 | NA | NA |
| FHIT | Colorectal cancer || id:ieu-b-4965 | ENSG00000189283 || id:eqtl-a-ENSG00000189283 | MR Egger | 13.99227867 | 0.233421025 | 0.000143465 | 0.332387111 |
| FHIT | Colorectal cancer || id:ieu-b-4965 | ENSG00000189283 || id:eqtl-a-ENSG00000189283 | Inverse variance weighted | 15.30005876 | 0.225435826 | NA | NA |
| TOMM7 | Colorectal cancer || id:ebi-a-GCST012876 | ENSG00000196683 || id:eqtl-a-ENSG00000196683 | Inverse variance weighted | 3.027333516 | 0.387430055 | NA | NA |
| TOMM7 | Colorectal cancer || id:ebi-a-GCST012876 | ENSG00000196683 || id:eqtl-a-ENSG00000196683 | MR Egger | 2.759352664 | 0.251659994 | 0.014521047 | 0.702476176 |
| PPTC7 | Colorectal cancer || id:ieu-b-4965 | ENSG00000196850 || id:eqtl-a-ENSG00000196850 | Inverse variance weighted | 2.31589733 | 0.509482244 | NA | NA |
| PPTC7 | Colorectal cancer || id:ieu-b-4965 | ENSG00000196850 || id:eqtl-a-ENSG00000196850 | MR Egger | 2.208104237 | 0.33152498 | 0.000121487 | 0.784257446 |
| PPTC7 | Colorectal cancer (SPA correction) || id:ebi-a-GCST90013866 | ENSG00000196850 || id:eqtl-a-ENSG00000196850 | MR Egger | 1.085697023 | 0.581090649 | -0.016988332 | 0.621772845 |
| PPTC7 | Colorectal cancer (Firth correction) || id:ebi-a-GCST90013862 | ENSG00000196850 || id:eqtl-a-ENSG00000196850 | Inverse variance weighted | 1.419571218 | 0.70095361 | NA | NA |
| PPTC7 | Colorectal cancer || id:ieu-b-4965 | ENSG00000196850 || id:eqtl-a-ENSG00000196850 | Inverse variance weighted | 2.31589733 | 0.509482244 | NA | NA |
| SLC25A29 | Colorectal cancer || id:ebi-a-GCST012878 | ENSG00000197119 || id:eqtl-a-ENSG00000197119 | Inverse variance weighted | 5.864100224 | 0.20952983 | NA | NA |
| SLC25A29 | Colorectal cancer || id:ebi-a-GCST012878 | ENSG00000197119 || id:eqtl-a-ENSG00000197119 | MR Egger | 5.678476072 | 0.128345032 | -0.03584076 | 0.774670359 |
| SND1 | Colorectal cancer || id:ebi-a-GCST90018588 | ENSG00000197157 || id:eqtl-a-ENSG00000197157 | MR Egger | 0.199211001 | 0.655358459 | 0.020075506 | 0.778910686 |
| SND1 | Colorectal cancer || id:ebi-a-GCST90018588 | ENSG00000197157 || id:eqtl-a-ENSG00000197157 | Inverse variance weighted | 0.330223307 | 0.847799039 | NA | NA |
| MRPL21 | Colorectal cancer || id:ebi-a-GCST012876 | ENSG00000197345 || id:eqtl-a-ENSG00000197345 | MR Egger | 3.687390775 | 0.595241517 | -0.022303437 | 0.511611599 |
| MRPL21 | Colorectal cancer || id:finn-b-C3_COLORECTAL | ENSG00000197345 || id:eqtl-a-ENSG00000197345 | MR Egger | 1.33757653 | 0.931018 | 0.04934013 | 0.322448489 |
| MRPL21 | Colorectal cancer || id:finn-b-C3_COLORECTAL | ENSG00000197345 || id:eqtl-a-ENSG00000197345 | Inverse variance weighted | 2.542051524 | 0.863732063 | NA | NA |
| MRPL21 | Colorectal cancer (all cancers excluded) || id:finn-b-C3_COLORECTAL_EXALLC | ENSG00000197345 || id:eqtl-a-ENSG00000197345 | MR Egger | 0.883363213 | 0.971414374 | 0.05951925 | 0.247649647 |
| MRPL21 | Colorectal cancer (all cancers excluded) || id:finn-b-C3_COLORECTAL_EXALLC | ENSG00000197345 || id:eqtl-a-ENSG00000197345 | Inverse variance weighted | 2.595287006 | 0.857654821 | NA | NA |
| MRPL21 | Colorectal cancer || id:ebi-a-GCST012876 | ENSG00000197345 || id:eqtl-a-ENSG00000197345 | Inverse variance weighted | 4.186084219 | 0.651510401 | NA | NA |
| GSTK1 | Colorectal cancer || id:ebi-a-GCST012880 | ENSG00000197448 || id:eqtl-a-ENSG00000197448 | Inverse variance weighted | 0.031477346 | 0.859179629 | NA | NA |
| TXNRD1 | Colorectal cancer || id:ebi-a-GCST012879 | ENSG00000198431 || id:eqtl-a-ENSG00000198431 | Inverse variance weighted | 1.385051755 | 0.23924252 | NA | NA |
| ECI2 | Colorectal cancer || id:finn-b-C3_COLORECTAL | ENSG00000198721 || id:eqtl-a-ENSG00000198721 | Inverse variance weighted | 0.515455947 | 0.772805427 | NA | NA |
| ECI2 | Colorectal cancer || id:ebi-a-GCST90018588 | ENSG00000198721 || id:eqtl-a-ENSG00000198721 | Inverse variance weighted | 1.862914419 | 0.393979182 | NA | NA |
| ECI2 | Colorectal cancer || id:ieu-b-4965 | ENSG00000198721 || id:eqtl-a-ENSG00000198721 | Inverse variance weighted | 0.040923023 | 0.979746405 | NA | NA |
| ECI2 | Colorectal cancer || id:ebi-a-GCST90018808 | ENSG00000198721 || id:eqtl-a-ENSG00000198721 | Inverse variance weighted | 0.767612161 | 0.681263525 | NA | NA |
| ECI2 | Colorectal cancer || id:ieu-b-4965 | ENSG00000198721 || id:eqtl-a-ENSG00000198721 | MR Egger | 0.03939273 | 0.842672647 | -1.29E-05 | 0.975108785 |
| ECI2 | Colorectal cancer (all cancers excluded) || id:finn-b-C3_COLORECTAL_EXALLC | ENSG00000198721 || id:eqtl-a-ENSG00000198721 | Inverse variance weighted | 0.682415777 | 0.710911103 | NA | NA |
| ECI2 | Colorectal cancer || id:ebi-a-GCST90018588 | ENSG00000198721 || id:eqtl-a-ENSG00000198721 | MR Egger | 0.965514428 | 0.325801421 | -0.019538562 | 0.517220688 |
| ECI2 | Colorectal cancer (all cancers excluded) || id:finn-b-C3_COLORECTAL_EXALLC | ENSG00000198721 || id:eqtl-a-ENSG00000198721 | MR Egger | 0.2459396 | 0.619948754 | -0.021386312 | 0.628319096 |
| ECI2 | Colorectal cancer || id:ebi-a-GCST90018808 | ENSG00000198721 || id:eqtl-a-ENSG00000198721 | MR Egger | 0.000759921 | 0.978007766 | -0.012951425 | 0.542125942 |
| ECI2 | Colorectal cancer || id:finn-b-C3_COLORECTAL | ENSG00000198721 || id:eqtl-a-ENSG00000198721 | MR Egger | 0.240363074 | 0.623944007 | -0.016798367 | 0.692481175 |
| ECI2 | Colorectal cancer || id:ieu-b-4965 | ENSG00000198721 || id:eqtl-a-ENSG00000198721 | Inverse variance weighted | 0.040923023 | 0.979746405 | NA | NA |
| COX20 | Colorectal cancer || id:ieu-b-4965 | ENSG00000203667 || id:eqtl-a-ENSG00000203667 | Inverse variance weighted | 0.422665426 | 0.515610036 | NA | NA |
| COX20 | Colorectal cancer || id:ieu-b-4965 | ENSG00000203667 || id:eqtl-a-ENSG00000203667 | Inverse variance weighted | 0.422665426 | 0.515610036 | NA | NA |
| COX20 | Colorectal cancer || id:ebi-a-GCST012876 | ENSG00000203667 || id:eqtl-a-ENSG00000203667 | Inverse variance weighted | 0.000264752 | 0.98701804 | NA | NA |
| NT5M | Colorectal cancer (all cancers excluded) || id:finn-b-C3_COLORECTAL_EXALLC | ENSG00000205309 || id:eqtl-a-ENSG00000205309 | MR Egger | 7.33038047 | 0.60276389 | -0.018064681 | 0.303696335 |
| NT5M | Colorectal cancer || id:finn-b-C3_COLORECTAL | ENSG00000205309 || id:eqtl-a-ENSG00000205309 | MR Egger | 8.328941688 | 0.501366692 | -0.017667802 | 0.308638362 |
| NT5M | Colorectal cancer (all cancers excluded) || id:finn-b-C3_COLORECTAL_EXALLC | ENSG00000205309 || id:eqtl-a-ENSG00000205309 | Inverse variance weighted | 8.52025222 | 0.578155385 | NA | NA |
| NT5M | Colorectal cancer || id:finn-b-C3_COLORECTAL | ENSG00000205309 || id:eqtl-a-ENSG00000205309 | Inverse variance weighted | 9.493266826 | 0.486015531 | NA | NA |
| CPT1B | Colorectal cancer || id:ebi-a-GCST012880 | ENSG00000205560 || id:eqtl-a-ENSG00000205560 | Inverse variance weighted | 1.410255961 | 0.235013907 | NA | NA |
| ARL2 | Colorectal cancer || id:ebi-a-GCST90018588 | ENSG00000213465 || id:eqtl-a-ENSG00000213465 | Inverse variance weighted | 0.026584284 | 0.870481526 | NA | NA |
| ARL2 | Colorectal cancer || id:ebi-a-GCST90018808 | ENSG00000213465 || id:eqtl-a-ENSG00000213465 | Inverse variance weighted | 0.015708066 | 0.90026086 | NA | NA |
| TSTD1 | Colorectal cancer || id:ieu-b-4965 | ENSG00000215845 || id:eqtl-a-ENSG00000215845 | Inverse variance weighted | 0.016184044 | 0.898769026 | NA | NA |
| TSTD1 | Colorectal cancer || id:ieu-b-4965 | ENSG00000215845 || id:eqtl-a-ENSG00000215845 | Inverse variance weighted | 0.016184044 | 0.898769026 | NA | NA |
| TSTD1 | Colorectal cancer (Firth correction) || id:ebi-a-GCST90013862 | ENSG00000215845 || id:eqtl-a-ENSG00000215845 | Inverse variance weighted | 0.000143592 | 0.990439203 | NA | NA |
| TSTD1 | Colorectal cancer (SPA correction) || id:ebi-a-GCST90013866 | ENSG00000215845 || id:eqtl-a-ENSG00000215845 | Inverse variance weighted | 0.000431007 | 0.983436543 | NA | NA |
| PAM16 | Colorectal cancer || id:ebi-a-GCST90018808 | ENSG00000217930 || id:eqtl-a-ENSG00000217930 | Inverse variance weighted | 0.850563329 | 0.653585667 | NA | NA |
| PAM16 | Colorectal cancer (SPA correction) || id:ebi-a-GCST90013866 | ENSG00000217930 || id:eqtl-a-ENSG00000217930 | Inverse variance weighted | 1.042250905 | 0.307298548 | NA | NA |
| PAM16 | Colorectal cancer || id:ebi-a-GCST90018588 | ENSG00000217930 || id:eqtl-a-ENSG00000217930 | Inverse variance weighted | 0.099476265 | 0.752459044 | NA | NA |
| PAM16 | Colorectal cancer || id:ebi-a-GCST90018808 | ENSG00000217930 || id:eqtl-a-ENSG00000217930 | MR Egger | 0.042585122 | 0.83650844 | 0.078466546 | 0.533870947 |
| PAM16 | Colorectal cancer || id:ebi-a-GCST012876 | ENSG00000217930 || id:eqtl-a-ENSG00000217930 | MR Egger | 4.868498069 | 0.027351295 | -0.137614618 | 0.642272288 |
| PAM16 | Colorectal cancer || id:ebi-a-GCST012876 | ENSG00000217930 || id:eqtl-a-ENSG00000217930 | Inverse variance weighted | 6.798497821 | 0.033398346 | NA | NA |
| PAM16 | Colorectal cancer (Firth correction) || id:ebi-a-GCST90013862 | ENSG00000217930 || id:eqtl-a-ENSG00000217930 | Inverse variance weighted | 1.029321282 | 0.310318132 | NA | NA |
| FAM185A | Colorectal cancer || id:ebi-a-GCST012876 | ENSG00000222011 || id:eqtl-a-ENSG00000222011 | Inverse variance weighted | 1.022958526 | 0.311818265 | NA | NA |
| PIGBOS1 | Colorectal cancer (all cancers excluded) || id:finn-b-C3_COLORECTAL_EXALLC | ENSG00000225973 || id:eqtl-a-ENSG00000225973 | Inverse variance weighted | 0.004074524 | 0.949104002 | NA | NA |
| PIGBOS1 | Colorectal cancer || id:ebi-a-GCST012876 | ENSG00000225973 || id:eqtl-a-ENSG00000225973 | Inverse variance weighted | 0.042460849 | 0.836743806 | NA | NA |
| ATP5MF | Colorectal cancer || id:ebi-a-GCST012880 | ENSG00000241468 || id:eqtl-a-ENSG00000241468 | Inverse variance weighted | 0.697699863 | 0.403557654 | NA | NA |
| CHCHD10 | Colorectal cancer || id:ebi-a-GCST012878 | ENSG00000250479 || id:eqtl-a-ENSG00000250479 | Inverse variance weighted | 0.450192936 | 0.502243346 | NA | NA |
| MPV17L2 | Colorectal cancer || id:ebi-a-GCST012878 | ENSG00000254858 || id:eqtl-a-ENSG00000254858 | Inverse variance weighted | 1.170487612 | 0.279301072 | NA | NA |
| MPV17L2 | Colorectal cancer || id:ebi-a-GCST012876 | ENSG00000254858 || id:eqtl-a-ENSG00000254858 | Inverse variance weighted | 0.174313492 | 0.676306382 | NA | NA |
| MPV17L2 | Colorectal cancer (all cancers excluded) || id:finn-b-C3_COLORECTAL_EXALLC | ENSG00000254858 || id:eqtl-a-ENSG00000254858 | Inverse variance weighted | 0.868104718 | 0.351480538 | NA | NA |

**Supplementary Table 4.** MR analysis used TwoSampleMR package on the causal effect of mitochondria-related genes mQTL on CRC outcomes.

| **gene** | **id.exposure** | **outcome** | **method** | **nsnp** | **pval** | **OR (95% CI)** |
| --- | --- | --- | --- | --- | --- | --- |
| SLC25A30 | cg00139037 | Colorectal cancer || id:ebi-a-GCST012876 | Inverse variance weighted (fixed effects) | 78 | 2.42E-41 | 1.152(1.129-1.176) |
| SLC25A30 | cg00139037 | Colorectal cancer || id:ebi-a-GCST012876 | Simple mode | 78 | 0.313533209 | 1.082(0.929-1.261) |
| SLC25A30 | cg00139037 | Colorectal cancer || id:ebi-a-GCST012876 | Weighted median | 78 | 1.08E-12 | 1.087(1.062-1.112) |
| SLC25A30 | cg00139037 | Colorectal cancer || id:ebi-a-GCST012876 | Weighted mode | 78 | 0.274254323 | 1.079(0.942-1.236) |
| SLC25A30 | cg00139037 | Colorectal cancer || id:ebi-a-GCST012876 | MR Egger | 78 | 3.04E-07 | 1.736(1.432-2.105) |
| MRPL32 | cg00365680 | Colorectal cancer || id:ebi-a-GCST012876 | Simple mode | 198 | 1.98E-23 | 0.875(0.855-0.896) |
| MRPL32 | cg00365680 | Colorectal cancer || id:ebi-a-GCST012876 | Weighted mode | 198 | 9.51E-20 | 0.904(0.887-0.922) |
| MRPL32 | cg00365680 | Colorectal cancer || id:ebi-a-GCST012876 | Weighted median | 198 | 2.95E-133 | 0.89(0.882-0.899) |
| MRPL32 | cg00365680 | Colorectal cancer || id:ebi-a-GCST012876 | Inverse variance weighted (fixed effects) | 198 | 0 | 0.883(0.877-0.889) |
| MRPL32 | cg00365680 | Colorectal cancer || id:ebi-a-GCST012876 | MR Egger | 198 | 5.58E-16 | 0.921(0.904-0.938) |
| ACSF3 | cg02193283 | Colorectal cancer || id:ebi-a-GCST012876 | Weighted median | 342 | 1.05E-102 | 1.185(1.166-1.203) |
| ACSF3 | cg02193283 | Colorectal cancer || id:ebi-a-GCST012876 | Inverse variance weighted (fixed effects) | 342 | 0 | 1.194(1.186-1.202) |
| ACSF3 | cg02193283 | Colorectal cancer || id:ebi-a-GCST012876 | MR Egger | 342 | 2.84E-42 | 1.328(1.282-1.376) |
| ACSF3 | cg02193283 | Colorectal cancer || id:ebi-a-GCST012876 | Simple mode | 342 | 4.81E-13 | 1.222(1.16-1.287) |
| ACSF3 | cg02193283 | Colorectal cancer || id:ebi-a-GCST012876 | Weighted mode | 342 | 6.38E-10 | 1.225(1.151-1.304) |
| ME3 | cg02493602 | Colorectal cancer || id:ebi-a-GCST012876 | Inverse variance weighted (fixed effects) | 227 | 0.274484878 | 1.004(0.997-1.011) |
| ME3 | cg02493602 | Colorectal cancer || id:ebi-a-GCST012876 | MR Egger | 227 | 0.066097421 | 1.018(0.999-1.038) |
| ME3 | cg02493602 | Colorectal cancer || id:ebi-a-GCST012876 | Weighted median | 227 | 0.676318649 | 1.002(0.993-1.011) |
| ME3 | cg02493602 | Colorectal cancer || id:ebi-a-GCST012876 | Simple mode | 227 | 0.98804837 | 1(0.978-1.022) |
| ME3 | cg02493602 | Colorectal cancer || id:ebi-a-GCST012876 | Weighted mode | 227 | 0.957155168 | 1.001(0.981-1.02) |
| SCP2 | cg03579872 | Colorectal cancer || id:ebi-a-GCST012876 | Weighted mode | 326 | 0.000198882 | 1.063(1.03-1.098) |
| SCP2 | cg03579872 | Colorectal cancer || id:ebi-a-GCST012876 | Weighted median | 326 | 3.85E-56 | 1.061(1.053-1.069) |
| SCP2 | cg03579872 | Colorectal cancer || id:ebi-a-GCST012876 | MR Egger | 326 | 3.24E-09 | 1.089(1.059-1.119) |
| SCP2 | cg03579872 | Colorectal cancer || id:ebi-a-GCST012876 | Inverse variance weighted (fixed effects) | 326 | 1.22E-108 | 1.061(1.056-1.067) |
| SCP2 | cg03579872 | Colorectal cancer || id:ebi-a-GCST012876 | Simple mode | 326 | 0.000743138 | 1.062(1.026-1.1) |
| ACSF3 | cg04308346 | Colorectal cancer || id:ebi-a-GCST012876 | MR Egger | 10 | 0.909908038 | 1.119(0.17-7.356) |
| ACSF3 | cg04308346 | Colorectal cancer || id:ebi-a-GCST012876 | Simple mode | 10 | 0.327064798 | 0.813(0.549-1.203) |
| ACSF3 | cg04308346 | Colorectal cancer || id:ebi-a-GCST012876 | Weighted median | 10 | 0.002059957 | 0.812(0.712-0.927) |
| ACSF3 | cg04308346 | Colorectal cancer || id:ebi-a-GCST012876 | Inverse variance weighted (fixed effects) | 10 | 6.11E-15 | 0.812(0.771-0.856) |
| ACSF3 | cg04308346 | Colorectal cancer || id:ebi-a-GCST012876 | Weighted mode | 10 | 0.369413651 | 0.816(0.534-1.245) |
| MSRB2 | cg07355157 | Colorectal cancer || id:ebi-a-GCST012876 | Inverse variance weighted (fixed effects) | 157 | 5.95E-06 | 0.984(0.977-0.991) |
| MSRB2 | cg07355157 | Colorectal cancer || id:ebi-a-GCST012876 | Simple mode | 157 | 0.00054335 | 0.969(0.953-0.986) |
| MSRB2 | cg07355157 | Colorectal cancer || id:ebi-a-GCST012876 | Weighted mode | 157 | 0.298208334 | 0.993(0.981-1.006) |
| MSRB2 | cg07355157 | Colorectal cancer || id:ebi-a-GCST012876 | Weighted median | 157 | 0.183773646 | 0.993(0.984-1.003) |
| MSRB2 | cg07355157 | Colorectal cancer || id:ebi-a-GCST012876 | MR Egger | 157 | 0.992882706 | 1(0.985-1.015) |
| CYB5R3 | cg08690876 | Colorectal cancer || id:ebi-a-GCST012876 | MR Egger | 342 | 0.048399287 | 0.986(0.972-1) |
| CYB5R3 | cg08690876 | Colorectal cancer || id:ebi-a-GCST012876 | Inverse variance weighted (fixed effects) | 342 | 7.04E-107 | 1.059(1.053-1.064) |
| CYB5R3 | cg08690876 | Colorectal cancer || id:ebi-a-GCST012876 | Weighted median | 342 | 3.20E-20 | 1.04(1.031-1.049) |
| CYB5R3 | cg08690876 | Colorectal cancer || id:ebi-a-GCST012876 | Weighted mode | 342 | 7.20E-06 | 1.033(1.019-1.047) |
| CYB5R3 | cg08690876 | Colorectal cancer || id:ebi-a-GCST012876 | Weighted median | 342 | 1.61E-20 | 1.04(1.031-1.048) |
| CYB5R3 | cg08690876 | Colorectal cancer || id:ebi-a-GCST012876 | Simple mode | 342 | 9.90E-08 | 1.057(1.036-1.078) |
| CYB5R3 | cg08690876 | Colorectal cancer || id:ebi-a-GCST012876 | Simple mode | 342 | 5.68E-09 | 1.057(1.038-1.076) |
| CYB5R3 | cg08690876 | Colorectal cancer || id:ebi-a-GCST012876 | Weighted mode | 342 | 8.07E-06 | 1.033(1.019-1.047) |
| VARS2 | cg10661769 | Colorectal cancer || id:ebi-a-GCST012876 | Inverse variance weighted (fixed effects) | 2 | 0.303503972 | 1.075(0.937-1.232) |
| LIPT2 | cg11417029 | Colorectal cancer || id:ebi-a-GCST012876 | Weighted mode | 113 | 0.213933407 | 1.048(0.974-1.129) |
| LIPT2 | cg11417029 | Colorectal cancer || id:ebi-a-GCST012876 | Inverse variance weighted (fixed effects) | 113 | 6.59E-28 | 1.088(1.072-1.105) |
| LIPT2 | cg11417029 | Colorectal cancer || id:ebi-a-GCST012876 | Simple mode | 113 | 0.007202446 | 1.122(1.033-1.218) |
| LIPT2 | cg11417029 | Colorectal cancer || id:ebi-a-GCST012876 | Weighted median | 113 | 2.02E-09 | 1.061(1.041-1.082) |
| LIPT2 | cg11417029 | Colorectal cancer || id:ebi-a-GCST012876 | MR Egger | 113 | 0.728383785 | 0.948(0.699-1.284) |
| MRPL28 | cg12437481 | Colorectal cancer || id:ebi-a-GCST012876 | Weighted median | 264 | 0.164506933 | 1.004(0.998-1.009) |
| MRPL28 | cg12437481 | Colorectal cancer || id:ebi-a-GCST012876 | Simple mode | 264 | 0.664061967 | 1.003(0.989-1.018) |
| MRPL28 | cg12437481 | Colorectal cancer || id:ebi-a-GCST012876 | MR Egger | 264 | 0.483950692 | 1.002(0.996-1.009) |
| MRPL28 | cg12437481 | Colorectal cancer || id:ebi-a-GCST012876 | Inverse variance weighted (fixed effects) | 264 | 0.034083368 | 1.004(1-1.008) |
| MRPL28 | cg12437481 | Colorectal cancer || id:ebi-a-GCST012876 | Weighted mode | 264 | 0.591987349 | 1.003(0.991-1.015) |
| ACADS | cg13914990 | Colorectal cancer || id:ebi-a-GCST012876 | Weighted mode | 624 | 6.69E-05 | 0.967(0.952-0.983) |
| ACADS | cg13914990 | Colorectal cancer || id:ebi-a-GCST012876 | MR Egger | 624 | 0.008307878 | 0.981(0.967-0.995) |
| ACADS | cg13914990 | Colorectal cancer || id:ebi-a-GCST012876 | Inverse variance weighted (fixed effects) | 624 | 7.46E-105 | 0.953(0.949-0.957) |
| ACADS | cg13914990 | Colorectal cancer || id:ebi-a-GCST012876 | Weighted median | 624 | 9.96E-22 | 0.967(0.961-0.974) |
| ACADS | cg13914990 | Colorectal cancer || id:ebi-a-GCST012876 | Simple mode | 624 | 0.001682392 | 0.967(0.948-0.988) |
| NSUN4 | cg14993813 | Colorectal cancer || id:ebi-a-GCST012876 | Weighted median | 251 | 1.02E-06 | 1.022(1.013-1.031) |
| NSUN4 | cg14993813 | Colorectal cancer || id:ebi-a-GCST012876 | MR Egger | 251 | 0.000498667 | 0.908(0.86-0.958) |
| NSUN4 | cg14993813 | Colorectal cancer || id:ebi-a-GCST012876 | Inverse variance weighted (fixed effects) | 251 | 2.31E-09 | 1.023(1.015-1.03) |
| NSUN4 | cg14993813 | Colorectal cancer || id:ebi-a-GCST012876 | Simple mode | 251 | 0.132938385 | 1.023(0.993-1.053) |
| NSUN4 | cg14993813 | Colorectal cancer || id:ebi-a-GCST012876 | Weighted mode | 251 | 0.090582251 | 1.023(0.997-1.049) |
| COX15 | cg17885402 | Colorectal cancer || id:ebi-a-GCST012876 | Weighted mode | 306 | 0.004563854 | 0.938(0.898-0.98) |
| COX15 | cg17885402 | Colorectal cancer || id:ebi-a-GCST012876 | Inverse variance weighted (fixed effects) | 306 | 3.14E-258 | 0.882(0.875-0.888) |
| COX15 | cg17885402 | Colorectal cancer || id:ebi-a-GCST012876 | Weighted median | 306 | 1.94E-18 | 0.934(0.92-0.949) |
| COX15 | cg17885402 | Colorectal cancer || id:ebi-a-GCST012876 | Simple mode | 306 | 0.008081533 | 0.938(0.895-0.983) |
| COX15 | cg17885402 | Colorectal cancer || id:ebi-a-GCST012876 | MR Egger | 306 | 3.97E-16 | 0.895(0.873-0.918) |
| PNKD | cg18259342 | Colorectal cancer || id:ebi-a-GCST012876 | MR Egger | 365 | 0.009243905 | 1.014(1.003-1.024) |
| PNKD | cg18259342 | Colorectal cancer || id:ebi-a-GCST012876 | Weighted mode | 365 | 3.55E-18 | 1.059(1.046-1.073) |
| PNKD | cg18259342 | Colorectal cancer || id:ebi-a-GCST012876 | Simple mode | 365 | 1.04E-13 | 1.063(1.047-1.079) |
| PNKD | cg18259342 | Colorectal cancer || id:ebi-a-GCST012876 | Inverse variance weighted (fixed effects) | 365 | 0 | 1.08(1.077-1.084) |
| PNKD | cg18259342 | Colorectal cancer || id:ebi-a-GCST012876 | Weighted median | 365 | 4.15E-56 | 1.062(1.054-1.07) |
| COX10 | cg20170777 | Colorectal cancer || id:ebi-a-GCST012876 | MR Egger | 47 | 0.11445077 | 1.236(0.955-1.6) |
| COX10 | cg20170777 | Colorectal cancer || id:ebi-a-GCST012876 | Weighted mode | 47 | 0.000222186 | 1.316(1.151-1.505) |
| COX10 | cg20170777 | Colorectal cancer || id:ebi-a-GCST012876 | Simple mode | 47 | 0.001134373 | 1.316(1.127-1.537) |
| COX10 | cg20170777 | Colorectal cancer || id:ebi-a-GCST012876 | Inverse variance weighted (fixed effects) | 47 | 8.73E-87 | 1.299(1.266-1.333) |
| COX10 | cg20170777 | Colorectal cancer || id:ebi-a-GCST012876 | Weighted median | 47 | 2.07E-15 | 1.294(1.214-1.379) |
| CASP9 | cg21858823 | Colorectal cancer || id:ebi-a-GCST012876 | Inverse variance weighted (fixed effects) | 2 | 0.418962528 | 0.947(0.829-1.081) |
| BAD | cg23796481 | Colorectal cancer || id:ebi-a-GCST012876 | Weighted median | 59 | 0.032909758 | 1.014(1.001-1.028) |
| BAD | cg23796481 | Colorectal cancer || id:ebi-a-GCST012876 | Inverse variance weighted (fixed effects) | 59 | 1.77E-05 | 1.023(1.012-1.033) |
| BAD | cg23796481 | Colorectal cancer || id:ebi-a-GCST012876 | Weighted mode | 59 | 0.436200238 | 1.011(0.983-1.04) |
| BAD | cg23796481 | Colorectal cancer || id:ebi-a-GCST012876 | Simple mode | 59 | 0.528544812 | 1.011(0.978-1.044) |
| BAD | cg23796481 | Colorectal cancer || id:ebi-a-GCST012876 | MR Egger | 59 | 0.101385177 | 1.055(0.991-1.123) |
| ABCD3 | cg24215727 | Colorectal cancer || id:ebi-a-GCST012876 | Weighted median | 93 | 2.40E-08 | 0.949(0.932-0.967) |
| ABCD3 | cg24215727 | Colorectal cancer || id:ebi-a-GCST012876 | Simple mode | 93 | 0.250451425 | 0.949(0.869-1.037) |
| ABCD3 | cg24215727 | Colorectal cancer || id:ebi-a-GCST012876 | Weighted mode | 93 | 0.200266652 | 0.95(0.878-1.027) |
| ABCD3 | cg24215727 | Colorectal cancer || id:ebi-a-GCST012876 | Inverse variance weighted (fixed effects) | 93 | 3.46E-13 | 0.93(0.912-0.949) |
| ABCD3 | cg24215727 | Colorectal cancer || id:ebi-a-GCST012876 | MR Egger | 93 | 0.26261672 | 1.298(0.825-2.043) |
| CISD3 | cg27492942 | Colorectal cancer || id:ebi-a-GCST012876 | Simple mode | 44 | 0.292644079 | 0.972(0.922-1.024) |
| CISD3 | cg27492942 | Colorectal cancer || id:ebi-a-GCST012876 | Weighted mode | 44 | 0.623194491 | 0.989(0.945-1.034) |
| CISD3 | cg27492942 | Colorectal cancer || id:ebi-a-GCST012876 | Inverse variance weighted (fixed effects) | 44 | 0.458817845 | 0.992(0.969-1.014) |
| CISD3 | cg27492942 | Colorectal cancer || id:ebi-a-GCST012876 | MR Egger | 44 | 0.760939566 | 1.013(0.932-1.101) |
| CISD3 | cg27492942 | Colorectal cancer || id:ebi-a-GCST012876 | Weighted median | 44 | 0.853936718 | 0.997(0.966-1.029) |
| SLC25A30 | cg00139037 | Colorectal cancer || id:ebi-a-GCST012877 | Weighted mode | 76 | 0.230045626 | 0.952(0.879-1.031) |
| SLC25A30 | cg00139037 | Colorectal cancer || id:ebi-a-GCST012877 | Weighted median | 76 | 1.55E-06 | 0.953(0.934-0.972) |
| SLC25A30 | cg00139037 | Colorectal cancer || id:ebi-a-GCST012877 | MR Egger | 76 | 0.169215193 | 1.161(0.94-1.433) |
| SLC25A30 | cg00139037 | Colorectal cancer || id:ebi-a-GCST012877 | Simple mode | 76 | 0.291378886 | 0.951(0.866-1.044) |
| SLC25A30 | cg00139037 | Colorectal cancer || id:ebi-a-GCST012877 | Inverse variance weighted (fixed effects) | 76 | 1.83E-08 | 0.938(0.917-0.959) |
| MRPL32 | cg00365680 | Colorectal cancer || id:ebi-a-GCST012877 | Weighted median | 198 | 0.188672472 | 1.006(0.997-1.015) |
| MRPL32 | cg00365680 | Colorectal cancer || id:ebi-a-GCST012877 | MR Egger | 198 | 0.083986428 | 0.982(0.963-1.002) |
| MRPL32 | cg00365680 | Colorectal cancer || id:ebi-a-GCST012877 | Inverse variance weighted (fixed effects) | 198 | 0.000853919 | 1.012(1.005-1.019) |
| MRPL32 | cg00365680 | Colorectal cancer || id:ebi-a-GCST012877 | Simple mode | 198 | 0.6753065 | 1.004(0.984-1.025) |
| MRPL32 | cg00365680 | Colorectal cancer || id:ebi-a-GCST012877 | Weighted mode | 198 | 0.614929044 | 1.005(0.986-1.025) |
| ACSF3 | cg02193283 | Colorectal cancer || id:ebi-a-GCST012877 | Weighted mode | 343 | 0.077769996 | 0.973(0.943-1.003) |
| ACSF3 | cg02193283 | Colorectal cancer || id:ebi-a-GCST012877 | Inverse variance weighted (fixed effects) | 343 | 1.32E-14 | 0.971(0.963-0.978) |
| ACSF3 | cg02193283 | Colorectal cancer || id:ebi-a-GCST012877 | Simple mode | 343 | 0.076159004 | 0.972(0.942-1.003) |
| ACSF3 | cg02193283 | Colorectal cancer || id:ebi-a-GCST012877 | Weighted median | 343 | 6.00E-09 | 0.971(0.961-0.98) |
| ACSF3 | cg02193283 | Colorectal cancer || id:ebi-a-GCST012877 | MR Egger | 343 | 0.0256721 | 0.958(0.922-0.995) |
| ME3 | cg02493602 | Colorectal cancer || id:ebi-a-GCST012877 | Weighted mode | 227 | 0.088677199 | 1.017(0.998-1.037) |
| ME3 | cg02493602 | Colorectal cancer || id:ebi-a-GCST012877 | Simple mode | 227 | 0.454154614 | 1.011(0.983-1.04) |
| ME3 | cg02493602 | Colorectal cancer || id:ebi-a-GCST012877 | Weighted median | 227 | 0.245905797 | 1.007(0.995-1.019) |
| ME3 | cg02493602 | Colorectal cancer || id:ebi-a-GCST012877 | MR Egger | 227 | 0.00376222 | 1.035(1.011-1.058) |
| SCP2 | cg03579872 | Colorectal cancer || id:ebi-a-GCST012877 | MR Egger | 65 | 0.018970076 | 1.199(1.034-1.391) |
| SCP2 | cg03579872 | Colorectal cancer || id:ebi-a-GCST012877 | Simple mode | 65 | 0.218811848 | 0.955(0.889-1.027) |
| SCP2 | cg03579872 | Colorectal cancer || id:ebi-a-GCST012877 | Weighted median | 65 | 0.000165934 | 0.956(0.934-0.979) |
| SCP2 | cg03579872 | Colorectal cancer || id:ebi-a-GCST012877 | Inverse variance weighted (fixed effects) | 65 | 0.429898655 | 0.992(0.972-1.012) |
| SCP2 | cg03579872 | Colorectal cancer || id:ebi-a-GCST012877 | Weighted mode | 65 | 0.246091675 | 0.955(0.885-1.031) |
| ACSF3 | cg04308346 | Colorectal cancer || id:ebi-a-GCST012877 | Inverse variance weighted (fixed effects) | 10 | 0.233102621 | 1.035(0.978-1.094) |
| ACSF3 | cg04308346 | Colorectal cancer || id:ebi-a-GCST012877 | Weighted median | 10 | 0.237207264 | 1.034(0.978-1.093) |
| ACSF3 | cg04308346 | Colorectal cancer || id:ebi-a-GCST012877 | MR Egger | 10 | 0.993893356 | 1.008(0.135-7.537) |
| ACSF3 | cg04308346 | Colorectal cancer || id:ebi-a-GCST012877 | Simple mode | 10 | 0.626994679 | 1.032(0.913-1.167) |
| ACSF3 | cg04308346 | Colorectal cancer || id:ebi-a-GCST012877 | Weighted mode | 10 | 0.620997724 | 1.032(0.916-1.163) |
| MSRB2 | cg07355157 | Colorectal cancer || id:ebi-a-GCST012877 | Weighted mode | 158 | 0.039405972 | 1.011(1.001-1.021) |
| MSRB2 | cg07355157 | Colorectal cancer || id:ebi-a-GCST012877 | Simple mode | 158 | 5.53E-51 | 1.192(1.174-1.21) |
| MSRB2 | cg07355157 | Colorectal cancer || id:ebi-a-GCST012877 | Weighted mode | 158 | 0.063388338 | 1.011(0.999-1.022) |
| MSRB2 | cg07355157 | Colorectal cancer || id:ebi-a-GCST012877 | MR Egger | 158 | 7.62E-13 | 0.94(0.925-0.954) |
| MSRB2 | cg07355157 | Colorectal cancer || id:ebi-a-GCST012877 | Simple mode | 158 | 2.27E-49 | 1.192(1.173-1.21) |
| MSRB2 | cg07355157 | Colorectal cancer || id:ebi-a-GCST012877 | Weighted median | 158 | 0.472355644 | 1.005(0.992-1.017) |
| MSRB2 | cg07355157 | Colorectal cancer || id:ebi-a-GCST012877 | Inverse variance weighted (fixed effects) | 158 | 5.16E-47 | 1.057(1.049-1.065) |
| MSRB2 | cg07355157 | Colorectal cancer || id:ebi-a-GCST012877 | Weighted median | 158 | 0.453729776 | 1.005(0.993-1.016) |
| CYB5R3 | cg08690876 | Colorectal cancer || id:ebi-a-GCST012877 | Simple mode | 351 | 0.004956634 | 0.972(0.953-0.991) |
| CYB5R3 | cg08690876 | Colorectal cancer || id:ebi-a-GCST012877 | Weighted mode | 351 | 0.042760419 | 0.984(0.968-0.999) |
| CYB5R3 | cg08690876 | Colorectal cancer || id:ebi-a-GCST012877 | MR Egger | 351 | 0.035482108 | 0.984(0.97-0.999) |
| CYB5R3 | cg08690876 | Colorectal cancer || id:ebi-a-GCST012877 | Inverse variance weighted (fixed effects) | 351 | 0.104800457 | 0.996(0.99-1.001) |
| CYB5R3 | cg08690876 | Colorectal cancer || id:ebi-a-GCST012877 | Weighted median | 351 | 0.000227804 | 0.984(0.976-0.993) |
| ECHDC2 | cg09128567 | Colorectal cancer || id:ebi-a-GCST012877 | Weighted mode | 253 | 0.036716304 | 1.06(1.004-1.119) |
| ECHDC2 | cg09128567 | Colorectal cancer || id:ebi-a-GCST012877 | Simple mode | 253 | 0.018982239 | 1.073(1.012-1.138) |
| ECHDC2 | cg09128567 | Colorectal cancer || id:ebi-a-GCST012877 | MR Egger | 253 | 0.004021502 | 1.1(1.031-1.173) |
| ECHDC2 | cg09128567 | Colorectal cancer || id:ebi-a-GCST012877 | Inverse variance weighted (fixed effects) | 253 | 2.01E-47 | 1.072(1.062-1.082) |
| ECHDC2 | cg09128567 | Colorectal cancer || id:ebi-a-GCST012877 | Weighted median | 253 | 3.13E-35 | 1.066(1.055-1.077) |
| LIPT2 | cg11417029 | Colorectal cancer || id:ebi-a-GCST012877 | Weighted median | 113 | 7.53E-18 | 1.102(1.078-1.127) |
| LIPT2 | cg11417029 | Colorectal cancer || id:ebi-a-GCST012877 | Inverse variance weighted (fixed effects) | 113 | 8.60E-36 | 1.109(1.091-1.127) |
| LIPT2 | cg11417029 | Colorectal cancer || id:ebi-a-GCST012877 | Weighted mode | 113 | 0.007795236 | 1.132(1.035-1.237) |
| LIPT2 | cg11417029 | Colorectal cancer || id:ebi-a-GCST012877 | MR Egger | 113 | 0.977315476 | 1.005(0.725-1.392) |
| LIPT2 | cg11417029 | Colorectal cancer || id:ebi-a-GCST012877 | Simple mode | 113 | 0.020732737 | 1.132(1.021-1.255) |
| MRPL28 | cg12437481 | Colorectal cancer || id:ebi-a-GCST012877 | Weighted median | 265 | 4.34E-22 | 1.029(1.023-1.035) |
| MRPL28 | cg12437481 | Colorectal cancer || id:ebi-a-GCST012877 | MR Egger | 265 | 1.49E-07 | 1.02(1.013-1.028) |
| MRPL28 | cg12437481 | Colorectal cancer || id:ebi-a-GCST012877 | Inverse variance weighted (fixed effects) | 265 | 4.81E-49 | 1.032(1.027-1.036) |
| MRPL28 | cg12437481 | Colorectal cancer || id:ebi-a-GCST012877 | Weighted mode | 265 | 5.53E-08 | 1.025(1.016-1.034) |
| MRPL28 | cg12437481 | Colorectal cancer || id:ebi-a-GCST012877 | Simple mode | 265 | 1.05E-06 | 1.026(1.016-1.037) |
| ACADS | cg13914990 | Colorectal cancer || id:ebi-a-GCST012877 | Simple mode | 622 | 0.015309554 | 1.023(1.004-1.042) |
| ACADS | cg13914990 | Colorectal cancer || id:ebi-a-GCST012877 | Weighted median | 622 | 0.06883819 | 1.006(1-1.013) |
| ACADS | cg13914990 | Colorectal cancer || id:ebi-a-GCST012877 | Weighted mode | 622 | 0.818474695 | 0.998(0.984-1.013) |
| ACADS | cg13914990 | Colorectal cancer || id:ebi-a-GCST012877 | Inverse variance weighted (fixed effects) | 622 | 2.08E-05 | 1.01(1.005-1.015) |
| ACADS | cg13914990 | Colorectal cancer || id:ebi-a-GCST012877 | MR Egger | 622 | 8.41E-13 | 0.944(0.929-0.959) |
| NSUN4 | cg14993813 | Colorectal cancer || id:ebi-a-GCST012877 | MR Egger | 251 | 0.001385639 | 0.908(0.857-0.963) |
| NSUN4 | cg14993813 | Colorectal cancer || id:ebi-a-GCST012877 | Weighted median | 251 | 0.110069569 | 1.008(0.998-1.017) |
| NSUN4 | cg14993813 | Colorectal cancer || id:ebi-a-GCST012877 | Weighted mode | 251 | 0.630588785 | 1.007(0.98-1.034) |
| NSUN4 | cg14993813 | Colorectal cancer || id:ebi-a-GCST012877 | Simple mode | 251 | 0.658383193 | 1.007(0.978-1.036) |
| NSUN4 | cg14993813 | Colorectal cancer || id:ebi-a-GCST012877 | Inverse variance weighted (fixed effects) | 251 | 0.000127444 | 1.016(1.008-1.024) |
| COX15 | cg17885402 | Colorectal cancer || id:ebi-a-GCST012877 | Weighted mode | 307 | 0.069437791 | 0.963(0.924-1.003) |
| COX15 | cg17885402 | Colorectal cancer || id:ebi-a-GCST012877 | Simple mode | 307 | 0.174569548 | 0.969(0.926-1.014) |
| COX15 | cg17885402 | Colorectal cancer || id:ebi-a-GCST012877 | Weighted median | 307 | 7.95E-06 | 0.966(0.952-0.981) |
| COX15 | cg17885402 | Colorectal cancer || id:ebi-a-GCST012877 | MR Egger | 307 | 0.000334536 | 0.931(0.896-0.968) |
| PNKD | cg18259342 | Colorectal cancer || id:ebi-a-GCST012877 | Simple mode | 366 | 1.12E-07 | 1.036(1.023-1.05) |
| PNKD | cg18259342 | Colorectal cancer || id:ebi-a-GCST012877 | Weighted mode | 366 | 2.00E-08 | 1.036(1.024-1.049) |
| PNKD | cg18259342 | Colorectal cancer || id:ebi-a-GCST012877 | Weighted median | 366 | 2.00E-31 | 1.03(1.025-1.035) |
| PNKD | cg18259342 | Colorectal cancer || id:ebi-a-GCST012877 | Inverse variance weighted (fixed effects) | 366 | 3.14E-47 | 1.025(1.021-1.028) |
| PNKD | cg18259342 | Colorectal cancer || id:ebi-a-GCST012877 | MR Egger | 366 | 1.65E-31 | 1.074(1.062-1.086) |
| COX10 | cg20170777 | Colorectal cancer || id:ebi-a-GCST012877 | Weighted mode | 47 | 0.429673053 | 1.029(0.959-1.103) |
| COX10 | cg20170777 | Colorectal cancer || id:ebi-a-GCST012877 | Simple mode | 47 | 0.449582904 | 1.03(0.955-1.112) |
| COX10 | cg20170777 | Colorectal cancer || id:ebi-a-GCST012877 | Inverse variance weighted (fixed effects) | 47 | 0.085785557 | 1.025(0.997-1.054) |
| COX10 | cg20170777 | Colorectal cancer || id:ebi-a-GCST012877 | Weighted median | 47 | 0.110442201 | 1.028(0.994-1.064) |
| COX10 | cg20170777 | Colorectal cancer || id:ebi-a-GCST012877 | MR Egger | 47 | 0.558469569 | 1.087(0.824-1.435) |
| CASP9 | cg21858823 | Colorectal cancer || id:ebi-a-GCST012877 | Weighted mode | 115 | 0.128206013 | 0.947(0.882-1.015) |
| CASP9 | cg21858823 | Colorectal cancer || id:ebi-a-GCST012877 | Weighted median | 115 | 5.22E-15 | 0.946(0.933-0.959) |
| CASP9 | cg21858823 | Colorectal cancer || id:ebi-a-GCST012877 | MR Egger | 115 | 0.459875661 | 0.947(0.82-1.094) |
| CASP9 | cg21858823 | Colorectal cancer || id:ebi-a-GCST012877 | Simple mode | 115 | 0.106448307 | 0.947(0.886-1.011) |
| CASP9 | cg21858823 | Colorectal cancer || id:ebi-a-GCST012877 | Inverse variance weighted (fixed effects) | 115 | 5.18E-18 | 0.941(0.929-0.954) |
| BAD | cg23796481 | Colorectal cancer || id:ebi-a-GCST012877 | MR Egger | 59 | 0.508233574 | 1.023(0.956-1.096) |
| BAD | cg23796481 | Colorectal cancer || id:ebi-a-GCST012877 | Weighted median | 59 | 8.77E-06 | 1.035(1.019-1.05) |
| BAD | cg23796481 | Colorectal cancer || id:ebi-a-GCST012877 | Weighted mode | 59 | 0.171118559 | 1.023(0.991-1.057) |
| BAD | cg23796481 | Colorectal cancer || id:ebi-a-GCST012877 | Simple mode | 59 | 0.010522471 | 1.05(1.013-1.09) |
| BAD | cg23796481 | Colorectal cancer || id:ebi-a-GCST012877 | Inverse variance weighted (fixed effects) | 59 | 3.60E-11 | 1.038(1.027-1.05) |
| ABCD3 | cg24215727 | Colorectal cancer || id:ebi-a-GCST012877 | MR Egger | 92 | 0.749557178 | 1.085(0.657-1.792) |
| ABCD3 | cg24215727 | Colorectal cancer || id:ebi-a-GCST012877 | Weighted median | 92 | 2.29E-27 | 0.858(0.834-0.882) |
| ABCD3 | cg24215727 | Colorectal cancer || id:ebi-a-GCST012877 | Inverse variance weighted (fixed effects) | 92 | 1.01E-60 | 0.838(0.821-0.856) |
| ABCD3 | cg24215727 | Colorectal cancer || id:ebi-a-GCST012877 | Weighted mode | 92 | 0.096088176 | 0.862(0.724-1.025) |
| ABCD3 | cg24215727 | Colorectal cancer || id:ebi-a-GCST012877 | Simple mode | 92 | 0.095985684 | 0.86(0.721-1.025) |
| CISD3 | cg27492942 | Colorectal cancer || id:ebi-a-GCST012877 | Weighted mode | 44 | 0.000844734 | 0.873(0.811-0.94) |
| CISD3 | cg27492942 | Colorectal cancer || id:ebi-a-GCST012877 | Inverse variance weighted (fixed effects) | 44 | 7.06E-24 | 0.883(0.862-0.905) |
| CISD3 | cg27492942 | Colorectal cancer || id:ebi-a-GCST012877 | Simple mode | 44 | 0.001750042 | 0.872(0.804-0.945) |
| CISD3 | cg27492942 | Colorectal cancer || id:ebi-a-GCST012877 | Weighted median | 44 | 1.95E-10 | 0.875(0.84-0.912) |
| CISD3 | cg27492942 | Colorectal cancer || id:ebi-a-GCST012877 | MR Egger | 44 | 0.000699841 | 0.847(0.774-0.926) |
| SLC25A30 | cg00139037 | Colorectal cancer || id:ebi-a-GCST012878 | Weighted mode | 80 | 0.32684489 | 0.909(0.751-1.099) |
| SLC25A30 | cg00139037 | Colorectal cancer || id:ebi-a-GCST012878 | Simple mode | 80 | 0.015916346 | 0.77(0.626-0.948) |
| SLC25A30 | cg00139037 | Colorectal cancer || id:ebi-a-GCST012878 | Inverse variance weighted (fixed effects) | 80 | 1.36E-06 | 0.854(0.801-0.911) |
| SLC25A30 | cg00139037 | Colorectal cancer || id:ebi-a-GCST012878 | MR Egger | 80 | 0.083496185 | 1.712(0.939-3.121) |
| SLC25A30 | cg00139037 | Colorectal cancer || id:ebi-a-GCST012878 | Weighted median | 80 | 0.000304121 | 0.902(0.853-0.954) |
| MRPL32 | cg00365680 | Colorectal cancer || id:ebi-a-GCST012878 | Weighted mode | 198 | 1.47E-07 | 1.186(1.115-1.261) |
| MRPL32 | cg00365680 | Colorectal cancer || id:ebi-a-GCST012878 | Simple mode | 198 | 1.99E-07 | 1.186(1.115-1.262) |
| MRPL32 | cg00365680 | Colorectal cancer || id:ebi-a-GCST012878 | Inverse variance weighted (fixed effects) | 198 | 4.32E-28 | 1.117(1.095-1.14) |
| MRPL32 | cg00365680 | Colorectal cancer || id:ebi-a-GCST012878 | Weighted median | 198 | 1.02E-17 | 1.126(1.096-1.156) |
| MRPL32 | cg00365680 | Colorectal cancer || id:ebi-a-GCST012878 | MR Egger | 198 | 3.77E-06 | 1.148(1.084-1.215) |
| ACSF3 | cg02193283 | Colorectal cancer || id:ebi-a-GCST012878 | Weighted median | 343 | 0.432789476 | 0.988(0.96-1.018) |
| ACSF3 | cg02193283 | Colorectal cancer || id:ebi-a-GCST012878 | Inverse variance weighted (fixed effects) | 343 | 0.015615151 | 0.971(0.949-0.995) |
| ACSF3 | cg02193283 | Colorectal cancer || id:ebi-a-GCST012878 | MR Egger | 343 | 0.773445557 | 0.983(0.876-1.103) |
| ACSF3 | cg02193283 | Colorectal cancer || id:ebi-a-GCST012878 | Weighted mode | 343 | 0.772403452 | 0.989(0.917-1.067) |
| ACSF3 | cg02193283 | Colorectal cancer || id:ebi-a-GCST012878 | Simple mode | 343 | 0.802475508 | 0.989(0.906-1.079) |
| ME3 | cg02493602 | Colorectal cancer || id:ebi-a-GCST012878 | MR Egger | 225 | 0.03409452 | 1.066(1.005-1.131) |
| ME3 | cg02493602 | Colorectal cancer || id:ebi-a-GCST012878 | Weighted median | 225 | 2.23E-20 | 1.173(1.134-1.214) |
| ME3 | cg02493602 | Colorectal cancer || id:ebi-a-GCST012878 | Inverse variance weighted (fixed effects) | 225 | 2.50E-105 | 1.261(1.235-1.288) |
| ME3 | cg02493602 | Colorectal cancer || id:ebi-a-GCST012878 | Simple mode | 225 | 2.97E-06 | 1.198(1.113-1.29) |
| ME3 | cg02493602 | Colorectal cancer || id:ebi-a-GCST012878 | Weighted mode | 225 | 6.92E-08 | 1.179(1.113-1.249) |
| SCP2 | cg03579872 | Colorectal cancer || id:ebi-a-GCST012878 | MR Egger | 65 | 0.278420802 | 1.25(0.838-1.866) |
| SCP2 | cg03579872 | Colorectal cancer || id:ebi-a-GCST012878 | Weighted mode | 65 | 0.051549803 | 1.201(1.002-1.439) |
| SCP2 | cg03579872 | Colorectal cancer || id:ebi-a-GCST012878 | Simple mode | 65 | 0.068736335 | 1.205(0.989-1.468) |
| SCP2 | cg03579872 | Colorectal cancer || id:ebi-a-GCST012878 | Inverse variance weighted (fixed effects) | 65 | 1.32E-07 | 1.166(1.101-1.235) |
| SCP2 | cg03579872 | Colorectal cancer || id:ebi-a-GCST012878 | Weighted median | 65 | 1.37E-05 | 1.153(1.081-1.229) |
| ACSF3 | cg04308346 | Colorectal cancer || id:ebi-a-GCST012878 | Simple mode | 10 | 0.6086313 | 0.917(0.665-1.264) |
| ACSF3 | cg04308346 | Colorectal cancer || id:ebi-a-GCST012878 | Weighted mode | 10 | 0.611381866 | 0.918(0.668-1.262) |
| ACSF3 | cg04308346 | Colorectal cancer || id:ebi-a-GCST012878 | Inverse variance weighted (fixed effects) | 10 | 0.309892273 | 0.92(0.784-1.081) |
| ACSF3 | cg04308346 | Colorectal cancer || id:ebi-a-GCST012878 | Weighted median | 10 | 0.267061267 | 0.919(0.793-1.066) |
| ACSF3 | cg04308346 | Colorectal cancer || id:ebi-a-GCST012878 | MR Egger | 10 | 0.982253251 | 0.934(0.003-307.77) |
| MSRB2 | cg07355157 | Colorectal cancer || id:ebi-a-GCST012878 | Weighted mode | 159 | 1.50E-05 | 1.077(1.042-1.112) |
| MSRB2 | cg07355157 | Colorectal cancer || id:ebi-a-GCST012878 | Simple mode | 159 | 2.24E-31 | 1.376(1.319-1.436) |
| MSRB2 | cg07355157 | Colorectal cancer || id:ebi-a-GCST012878 | Inverse variance weighted (fixed effects) | 159 | 2.60E-31 | 1.137(1.113-1.162) |
| MSRB2 | cg07355157 | Colorectal cancer || id:ebi-a-GCST012878 | Weighted median | 159 | 8.01E-05 | 1.071(1.035-1.109) |
| MSRB2 | cg07355157 | Colorectal cancer || id:ebi-a-GCST012878 | MR Egger | 159 | 0.036582088 | 0.953(0.911-0.997) |
| CYB5R3 | cg08690876 | Colorectal cancer || id:ebi-a-GCST012878 | MR Egger | 341 | 0.000132418 | 0.919(0.88-0.959) |
| CYB5R3 | cg08690876 | Colorectal cancer || id:ebi-a-GCST012878 | Simple mode | 341 | 0.027129754 | 1.073(1.008-1.141) |
| CYB5R3 | cg08690876 | Colorectal cancer || id:ebi-a-GCST012878 | Weighted mode | 341 | 0.024545083 | 1.061(1.008-1.117) |
| CYB5R3 | cg08690876 | Colorectal cancer || id:ebi-a-GCST012878 | Weighted median | 341 | 1.20E-06 | 1.058(1.034-1.082) |
| CYB5R3 | cg08690876 | Colorectal cancer || id:ebi-a-GCST012878 | Inverse variance weighted (fixed effects) | 341 | 7.82E-07 | 1.041(1.024-1.057) |
| ECHDC2 | cg09128567 | Colorectal cancer || id:ebi-a-GCST012878 | MR Egger | 253 | 0.430051062 | 1.08(0.892-1.307) |
| ECHDC2 | cg09128567 | Colorectal cancer || id:ebi-a-GCST012878 | Weighted median | 253 | 5.02E-26 | 1.278(1.221-1.337) |
| ECHDC2 | cg09128567 | Colorectal cancer || id:ebi-a-GCST012878 | Inverse variance weighted (fixed effects) | 253 | 6.22E-118 | 1.391(1.353-1.431) |
| ECHDC2 | cg09128567 | Colorectal cancer || id:ebi-a-GCST012878 | Simple mode | 253 | 0.001570753 | 1.343(1.121-1.609) |
| ECHDC2 | cg09128567 | Colorectal cancer || id:ebi-a-GCST012878 | Weighted mode | 253 | 0.000334586 | 1.292(1.125-1.483) |
| VARS2 | cg10661769 | Colorectal cancer || id:ebi-a-GCST012878 | Inverse variance weighted (fixed effects) | 2 | 0.328616594 | 1.24(0.805-1.909) |
| LIPT2 | cg11417029 | Colorectal cancer || id:ebi-a-GCST012878 | Simple mode | 113 | 0.061632066 | 1.406(0.987-2.003) |
| LIPT2 | cg11417029 | Colorectal cancer || id:ebi-a-GCST012878 | MR Egger | 113 | 0.227285732 | 1.819(0.692-4.779) |
| LIPT2 | cg11417029 | Colorectal cancer || id:ebi-a-GCST012878 | Weighted median | 113 | 1.03E-24 | 1.405(1.316-1.499) |
| LIPT2 | cg11417029 | Colorectal cancer || id:ebi-a-GCST012878 | Inverse variance weighted (fixed effects) | 113 | 2.19E-40 | 1.387(1.322-1.456) |
| LIPT2 | cg11417029 | Colorectal cancer || id:ebi-a-GCST012878 | Weighted mode | 113 | 0.027953003 | 1.406(1.042-1.898) |
| MRPL28 | cg12437481 | Colorectal cancer || id:ebi-a-GCST012878 | MR Egger | 261 | 0.001536143 | 1.036(1.014-1.058) |
| MRPL28 | cg12437481 | Colorectal cancer || id:ebi-a-GCST012878 | Simple mode | 261 | 0.3068251 | 1.015(0.987-1.044) |
| MRPL28 | cg12437481 | Colorectal cancer || id:ebi-a-GCST012878 | Weighted median | 261 | 0.032214353 | 1.018(1.002-1.035) |
| MRPL28 | cg12437481 | Colorectal cancer || id:ebi-a-GCST012878 | Weighted mode | 261 | 0.243714185 | 1.015(0.99-1.04) |
| MRPL28 | cg12437481 | Colorectal cancer || id:ebi-a-GCST012878 | Inverse variance weighted (fixed effects) | 261 | 0.194725279 | 1.008(0.996-1.02) |
| ACADS | cg13914990 | Colorectal cancer || id:ebi-a-GCST012878 | MR Egger | 624 | 1.56E-05 | 0.903(0.862-0.945) |
| ACADS | cg13914990 | Colorectal cancer || id:ebi-a-GCST012878 | Inverse variance weighted (fixed effects) | 624 | 2.18E-37 | 1.093(1.078-1.108) |
| ACADS | cg13914990 | Colorectal cancer || id:ebi-a-GCST012878 | Weighted median | 624 | 0.000464766 | 1.039(1.017-1.061) |
| ACADS | cg13914990 | Colorectal cancer || id:ebi-a-GCST012878 | Weighted mode | 624 | 0.071688809 | 1.034(0.997-1.072) |
| ACADS | cg13914990 | Colorectal cancer || id:ebi-a-GCST012878 | Simple mode | 624 | 3.52E-14 | 1.231(1.168-1.298) |
| NSUN4 | cg14993813 | Colorectal cancer || id:ebi-a-GCST012878 | Weighted mode | 251 | 0.067743406 | 0.918(0.838-1.006) |
| NSUN4 | cg14993813 | Colorectal cancer || id:ebi-a-GCST012878 | MR Egger | 251 | 0.616908278 | 0.956(0.803-1.139) |
| NSUN4 | cg14993813 | Colorectal cancer || id:ebi-a-GCST012878 | Weighted median | 251 | 3.91E-09 | 0.917(0.891-0.944) |
| NSUN4 | cg14993813 | Colorectal cancer || id:ebi-a-GCST012878 | Simple mode | 251 | 0.082090864 | 0.918(0.834-1.011) |
| NSUN4 | cg14993813 | Colorectal cancer || id:ebi-a-GCST012878 | Inverse variance weighted (fixed effects) | 251 | 1.66E-13 | 0.912(0.89-0.935) |
| COX15 | cg17885402 | Colorectal cancer || id:ebi-a-GCST012878 | Inverse variance weighted (fixed effects) | 306 | 1.11E-33 | 0.869(0.85-0.889) |
| COX15 | cg17885402 | Colorectal cancer || id:ebi-a-GCST012878 | Weighted median | 306 | 2.06E-11 | 0.89(0.86-0.921) |
| COX15 | cg17885402 | Colorectal cancer || id:ebi-a-GCST012878 | MR Egger | 306 | 6.05E-08 | 0.799(0.738-0.865) |
| COX15 | cg17885402 | Colorectal cancer || id:ebi-a-GCST012878 | Simple mode | 306 | 0.252203253 | 0.943(0.853-1.042) |
| COX15 | cg17885402 | Colorectal cancer || id:ebi-a-GCST012878 | Weighted mode | 306 | 0.004130631 | 0.88(0.806-0.96) |
| PNKD | cg18259342 | Colorectal cancer || id:ebi-a-GCST012878 | Weighted mode | 366 | 3.17E-09 | 1.151(1.1-1.204) |
| PNKD | cg18259342 | Colorectal cancer || id:ebi-a-GCST012878 | Simple mode | 366 | 1.92E-10 | 1.174(1.119-1.231) |
| PNKD | cg18259342 | Colorectal cancer || id:ebi-a-GCST012878 | Inverse variance weighted (fixed effects) | 366 | 2.57E-175 | 1.149(1.138-1.161) |
| PNKD | cg18259342 | Colorectal cancer || id:ebi-a-GCST012878 | MR Egger | 366 | 1.07E-21 | 1.178(1.141-1.215) |
| PNKD | cg18259342 | Colorectal cancer || id:ebi-a-GCST012878 | Weighted median | 366 | 7.96E-64 | 1.149(1.131-1.168) |
| COX10 | cg20170777 | Colorectal cancer || id:ebi-a-GCST012878 | Weighted mode | 47 | 0.000188677 | 0.553(0.415-0.736) |
| COX10 | cg20170777 | Colorectal cancer || id:ebi-a-GCST012878 | Simple mode | 47 | 0.000613391 | 0.476(0.321-0.707) |
| COX10 | cg20170777 | Colorectal cancer || id:ebi-a-GCST012878 | Weighted median | 47 | 1.25E-10 | 0.558(0.467-0.667) |
| COX10 | cg20170777 | Colorectal cancer || id:ebi-a-GCST012878 | Inverse variance weighted (fixed effects) | 47 | 8.94E-48 | 0.549(0.507-0.596) |
| COX10 | cg20170777 | Colorectal cancer || id:ebi-a-GCST012878 | MR Egger | 47 | 0.944042568 | 0.971(0.435-2.169) |
| CASP9 | cg21858823 | Colorectal cancer || id:ebi-a-GCST012878 | Inverse variance weighted (fixed effects) | 2 | 0.19657961 | 1.319(0.866-2.007) |
| BAD | cg23796481 | Colorectal cancer || id:ebi-a-GCST012878 | Simple mode | 59 | 0.117078628 | 0.935(0.861-1.016) |
| BAD | cg23796481 | Colorectal cancer || id:ebi-a-GCST012878 | Weighted mode | 59 | 0.126524 | 0.936(0.861-1.018) |
| BAD | cg23796481 | Colorectal cancer || id:ebi-a-GCST012878 | MR Egger | 59 | 0.606522582 | 1.052(0.868-1.275) |
| BAD | cg23796481 | Colorectal cancer || id:ebi-a-GCST012878 | Weighted median | 59 | 0.006478343 | 0.944(0.906-0.984) |
| BAD | cg23796481 | Colorectal cancer || id:ebi-a-GCST012878 | Inverse variance weighted (fixed effects) | 59 | 0.000268993 | 0.942(0.912-0.973) |
| ABCD3 | cg24215727 | Colorectal cancer || id:ebi-a-GCST012878 | Simple mode | 92 | 0.066029912 | 0.42(0.169-1.047) |
| ABCD3 | cg24215727 | Colorectal cancer || id:ebi-a-GCST012878 | Inverse variance weighted (fixed effects) | 92 | 1.94E-180 | 0.408(0.384-0.434) |
| ABCD3 | cg24215727 | Colorectal cancer || id:ebi-a-GCST012878 | MR Egger | 92 | 0.178698829 | 0.364(0.084-1.57) |
| ABCD3 | cg24215727 | Colorectal cancer || id:ebi-a-GCST012878 | Weighted median | 92 | 1.37E-54 | 0.42(0.376-0.468) |
| ABCD3 | cg24215727 | Colorectal cancer || id:ebi-a-GCST012878 | Weighted mode | 92 | 0.043913449 | 0.42(0.183-0.965) |
| CISD3 | cg27492942 | Colorectal cancer || id:ebi-a-GCST012878 | Inverse variance weighted (fixed effects) | 44 | 1.07E-05 | 1.165(1.089-1.248) |
| CISD3 | cg27492942 | Colorectal cancer || id:ebi-a-GCST012878 | Weighted mode | 44 | 0.121207002 | 1.115(0.974-1.275) |
| CISD3 | cg27492942 | Colorectal cancer || id:ebi-a-GCST012878 | Simple mode | 44 | 0.08782352 | 1.158(0.982-1.366) |
| CISD3 | cg27492942 | Colorectal cancer || id:ebi-a-GCST012878 | MR Egger | 44 | 0.353255654 | 0.887(0.691-1.139) |
| CISD3 | cg27492942 | Colorectal cancer || id:ebi-a-GCST012878 | Weighted median | 44 | 0.021411447 | 1.125(1.018-1.244) |
| SLC25A30 | cg00139037 | Colorectal cancer || id:ebi-a-GCST012879 | MR Egger | 82 | 1.19E-07 | 1.698(1.42-2.029) |
| SLC25A30 | cg00139037 | Colorectal cancer || id:ebi-a-GCST012879 | Weighted median | 82 | 2.31E-10 | 1.055(1.038-1.073) |
| SLC25A30 | cg00139037 | Colorectal cancer || id:ebi-a-GCST012879 | Inverse variance weighted (fixed effects) | 82 | 0.297320762 | 1.01(0.991-1.029) |
| SLC25A30 | cg00139037 | Colorectal cancer || id:ebi-a-GCST012879 | Simple mode | 82 | 0.251168496 | 0.942(0.85-1.043) |
| SLC25A30 | cg00139037 | Colorectal cancer || id:ebi-a-GCST012879 | Weighted mode | 82 | 0.234484495 | 1.058(0.965-1.16) |
| MRPL32 | cg00365680 | Colorectal cancer || id:ebi-a-GCST012879 | Simple mode | 197 | 0.282294893 | 1.01(0.992-1.028) |
| MRPL32 | cg00365680 | Colorectal cancer || id:ebi-a-GCST012879 | Inverse variance weighted (fixed effects) | 197 | 2.09E-07 | 1.015(1.01-1.021) |
| MRPL32 | cg00365680 | Colorectal cancer || id:ebi-a-GCST012879 | Weighted median | 197 | 0.003166663 | 1.011(1.004-1.019) |
| MRPL32 | cg00365680 | Colorectal cancer || id:ebi-a-GCST012879 | MR Egger | 197 | 0.408500725 | 0.993(0.976-1.01) |
| MRPL32 | cg00365680 | Colorectal cancer || id:ebi-a-GCST012879 | Weighted mode | 197 | 0.284156851 | 1.01(0.992-1.028) |
| ACSF3 | cg02193283 | Colorectal cancer || id:ebi-a-GCST012879 | Inverse variance weighted (fixed effects) | 343 | 2.49E-23 | 1.035(1.028-1.042) |
| ACSF3 | cg02193283 | Colorectal cancer || id:ebi-a-GCST012879 | Weighted median | 343 | 5.68E-14 | 1.037(1.027-1.046) |
| ACSF3 | cg02193283 | Colorectal cancer || id:ebi-a-GCST012879 | Simple mode | 343 | 0.01608231 | 1.04(1.007-1.073) |
| ACSF3 | cg02193283 | Colorectal cancer || id:ebi-a-GCST012879 | MR Egger | 343 | 1.07E-09 | 1.113(1.077-1.151) |
| ACSF3 | cg02193283 | Colorectal cancer || id:ebi-a-GCST012879 | Weighted mode | 343 | 0.006123354 | 1.038(1.011-1.065) |
| ME3 | cg02493602 | Colorectal cancer || id:ebi-a-GCST012879 | Weighted mode | 226 | 5.06E-06 | 1.046(1.026-1.065) |
| ME3 | cg02493602 | Colorectal cancer || id:ebi-a-GCST012879 | Simple mode | 226 | 0.014353859 | 1.03(1.006-1.054) |
| ME3 | cg02493602 | Colorectal cancer || id:ebi-a-GCST012879 | Inverse variance weighted (fixed effects) | 226 | 1.59E-48 | 1.048(1.041-1.055) |
| ME3 | cg02493602 | Colorectal cancer || id:ebi-a-GCST012879 | MR Egger | 226 | 3.01E-11 | 1.065(1.046-1.084) |
| ME3 | cg02493602 | Colorectal cancer || id:ebi-a-GCST012879 | Weighted median | 226 | 1.63E-21 | 1.05(1.039-1.06) |
| SCP2 | cg03579872 | Colorectal cancer || id:ebi-a-GCST012879 | Inverse variance weighted (fixed effects) | 65 | 1.87E-11 | 1.063(1.044-1.082) |
| SCP2 | cg03579872 | Colorectal cancer || id:ebi-a-GCST012879 | Simple mode | 65 | 0.088297061 | 1.067(0.991-1.149) |
| SCP2 | cg03579872 | Colorectal cancer || id:ebi-a-GCST012879 | Weighted median | 65 | 1.71E-09 | 1.065(1.043-1.086) |
| SCP2 | cg03579872 | Colorectal cancer || id:ebi-a-GCST012879 | MR Egger | 65 | 0.231364574 | 1.097(0.944-1.275) |
| SCP2 | cg03579872 | Colorectal cancer || id:ebi-a-GCST012879 | Weighted mode | 65 | 0.090069113 | 1.067(0.991-1.149) |
| ACSF3 | cg04308346 | Colorectal cancer || id:ebi-a-GCST012879 | Inverse variance weighted (fixed effects) | 10 | 7.24E-06 | 0.893(0.851-0.939) |
| ACSF3 | cg04308346 | Colorectal cancer || id:ebi-a-GCST012879 | Simple mode | 10 | 0.366516924 | 0.894(0.709-1.127) |
| ACSF3 | cg04308346 | Colorectal cancer || id:ebi-a-GCST012879 | Weighted mode | 10 | 0.365835153 | 0.894(0.709-1.126) |
| ACSF3 | cg04308346 | Colorectal cancer || id:ebi-a-GCST012879 | Weighted median | 10 | 0.003537258 | 0.894(0.829-0.964) |
| ACSF3 | cg04308346 | Colorectal cancer || id:ebi-a-GCST012879 | MR Egger | 10 | 0.901654652 | 0.891(0.151-5.265) |
| MSRB2 | cg07355157 | Colorectal cancer || id:ebi-a-GCST012879 | Weighted median | 160 | 0.009299252 | 0.987(0.977-0.997) |
| MSRB2 | cg07355157 | Colorectal cancer || id:ebi-a-GCST012879 | Inverse variance weighted (fixed effects) | 160 | 7.53E-09 | 0.981(0.974-0.987) |
| MSRB2 | cg07355157 | Colorectal cancer || id:ebi-a-GCST012879 | Simple mode | 160 | 2.09E-34 | 0.897(0.885-0.909) |
| MSRB2 | cg07355157 | Colorectal cancer || id:ebi-a-GCST012879 | Weighted mode | 160 | 0.069291808 | 0.991(0.981-1.001) |
| MSRB2 | cg07355157 | Colorectal cancer || id:ebi-a-GCST012879 | MR Egger | 160 | 0.000123754 | 1.028(1.014-1.042) |
| CYB5R3 | cg08690876 | Colorectal cancer || id:ebi-a-GCST012879 | Inverse variance weighted (fixed effects) | 352 | 1.87E-14 | 1.019(1.014-1.023) |
| CYB5R3 | cg08690876 | Colorectal cancer || id:ebi-a-GCST012879 | MR Egger | 352 | 1.94E-06 | 1.032(1.019-1.046) |
| CYB5R3 | cg08690876 | Colorectal cancer || id:ebi-a-GCST012879 | Simple mode | 352 | 0.298872298 | 1.009(0.992-1.027) |
| CYB5R3 | cg08690876 | Colorectal cancer || id:ebi-a-GCST012879 | Weighted median | 352 | 0.002129542 | 1.012(1.004-1.019) |
| CYB5R3 | cg08690876 | Colorectal cancer || id:ebi-a-GCST012879 | Weighted mode | 352 | 0.110733334 | 1.01(0.998-1.023) |
| ECHDC2 | cg09128567 | Colorectal cancer || id:ebi-a-GCST012879 | MR Egger | 256 | 7.60E-11 | 1.217(1.15-1.288) |
| ECHDC2 | cg09128567 | Colorectal cancer || id:ebi-a-GCST012879 | Weighted median | 256 | 4.78E-62 | 1.101(1.088-1.113) |
| ECHDC2 | cg09128567 | Colorectal cancer || id:ebi-a-GCST012879 | Inverse variance weighted (fixed effects) | 256 | 1.31E-133 | 1.11(1.1-1.119) |
| ECHDC2 | cg09128567 | Colorectal cancer || id:ebi-a-GCST012879 | Simple mode | 256 | 0.010759687 | 1.105(1.024-1.193) |
| ECHDC2 | cg09128567 | Colorectal cancer || id:ebi-a-GCST012879 | Weighted mode | 256 | 0.009977279 | 1.104(1.025-1.189) |
| VARS2 | cg10661769 | Colorectal cancer || id:ebi-a-GCST012879 | Inverse variance weighted (fixed effects) | 2 | 0.036317478 | 1.145(1.009-1.299) |
| LIPT2 | cg11417029 | Colorectal cancer || id:ebi-a-GCST012879 | Weighted median | 113 | 1.03E-10 | 1.048(1.033-1.063) |
| LIPT2 | cg11417029 | Colorectal cancer || id:ebi-a-GCST012879 | MR Egger | 113 | 0.412803073 | 1.127(0.847-1.5) |
| LIPT2 | cg11417029 | Colorectal cancer || id:ebi-a-GCST012879 | Inverse variance weighted (fixed effects) | 113 | 1.86E-08 | 1.042(1.027-1.057) |
| LIPT2 | cg11417029 | Colorectal cancer || id:ebi-a-GCST012879 | Simple mode | 113 | 0.076296722 | 1.056(0.995-1.12) |
| LIPT2 | cg11417029 | Colorectal cancer || id:ebi-a-GCST012879 | Weighted mode | 113 | 0.063985121 | 1.056(0.997-1.118) |
| MRPL28 | cg12437481 | Colorectal cancer || id:ebi-a-GCST012879 | MR Egger | 264 | 4.08E-11 | 0.977(0.971-0.984) |
| MRPL28 | cg12437481 | Colorectal cancer || id:ebi-a-GCST012879 | Weighted mode | 264 | 0.137046581 | 0.989(0.975-1.003) |
| MRPL28 | cg12437481 | Colorectal cancer || id:ebi-a-GCST012879 | Simple mode | 264 | 0.898828332 | 1.001(0.985-1.018) |
| MRPL28 | cg12437481 | Colorectal cancer || id:ebi-a-GCST012879 | Weighted median | 264 | 0.020031721 | 0.993(0.987-0.999) |
| MRPL28 | cg12437481 | Colorectal cancer || id:ebi-a-GCST012879 | Inverse variance weighted (fixed effects) | 264 | 0.012462263 | 1.005(1.001-1.008) |
| ACADS | cg13914990 | Colorectal cancer || id:ebi-a-GCST012879 | Weighted mode | 627 | 2.71E-10 | 1.062(1.043-1.082) |
| ACADS | cg13914990 | Colorectal cancer || id:ebi-a-GCST012879 | Simple mode | 627 | 8.56E-08 | 1.082(1.051-1.113) |
| ACADS | cg13914990 | Colorectal cancer || id:ebi-a-GCST012879 | Inverse variance weighted (fixed effects) | 627 | 3.41E-282 | 1.078(1.073-1.082) |
| ACADS | cg13914990 | Colorectal cancer || id:ebi-a-GCST012879 | Weighted median | 627 | 7.80E-51 | 1.067(1.058-1.076) |
| ACADS | cg13914990 | Colorectal cancer || id:ebi-a-GCST012879 | MR Egger | 627 | 3.12E-42 | 1.109(1.094-1.124) |
| NSUN4 | cg14993813 | Colorectal cancer || id:ebi-a-GCST012879 | Weighted mode | 242 | 0.004351271 | 0.947(0.913-0.983) |
| NSUN4 | cg14993813 | Colorectal cancer || id:ebi-a-GCST012879 | Simple mode | 242 | 8.33E-06 | 0.913(0.878-0.949) |
| NSUN4 | cg14993813 | Colorectal cancer || id:ebi-a-GCST012879 | Inverse variance weighted (fixed effects) | 242 | 1.96E-71 | 0.933(0.926-0.94) |
| NSUN4 | cg14993813 | Colorectal cancer || id:ebi-a-GCST012879 | Weighted median | 242 | 9.30E-26 | 0.944(0.934-0.954) |
| NSUN4 | cg14993813 | Colorectal cancer || id:ebi-a-GCST012879 | MR Egger | 242 | 2.64E-05 | 0.892(0.847-0.94) |
| COX15 | cg17885402 | Colorectal cancer || id:ebi-a-GCST012879 | Weighted median | 305 | 2.19E-49 | 0.895(0.882-0.909) |
| COX15 | cg17885402 | Colorectal cancer || id:ebi-a-GCST012879 | Inverse variance weighted (fixed effects) | 305 | 2.91E-300 | 0.879(0.873-0.885) |
| COX15 | cg17885402 | Colorectal cancer || id:ebi-a-GCST012879 | MR Egger | 305 | 2.45E-13 | 0.91(0.889-0.932) |
| COX15 | cg17885402 | Colorectal cancer || id:ebi-a-GCST012879 | Simple mode | 305 | 3.95E-06 | 0.895(0.854-0.937) |
| COX15 | cg17885402 | Colorectal cancer || id:ebi-a-GCST012879 | Weighted mode | 305 | 2.28E-06 | 0.895(0.855-0.936) |
| PNKD | cg18259342 | Colorectal cancer || id:ebi-a-GCST012879 | Weighted mode | 363 | 3.92E-13 | 1.073(1.053-1.093) |
| PNKD | cg18259342 | Colorectal cancer || id:ebi-a-GCST012879 | Simple mode | 363 | 4.46E-10 | 1.069(1.047-1.091) |
| PNKD | cg18259342 | Colorectal cancer || id:ebi-a-GCST012879 | Weighted median | 363 | 3.18E-88 | 1.073(1.065-1.08) |
| PNKD | cg18259342 | Colorectal cancer || id:ebi-a-GCST012879 | Inverse variance weighted (fixed effects) | 363 | 0 | 1.076(1.072-1.079) |
| PNKD | cg18259342 | Colorectal cancer || id:ebi-a-GCST012879 | MR Egger | 363 | 9.10E-39 | 1.077(1.067-1.088) |
| COX10 | cg20170777 | Colorectal cancer || id:ebi-a-GCST012879 | Inverse variance weighted (fixed effects) | 47 | 1.97E-09 | 1.079(1.052-1.106) |
| COX10 | cg20170777 | Colorectal cancer || id:ebi-a-GCST012879 | Simple mode | 47 | 0.057467404 | 1.078(1-1.163) |
| COX10 | cg20170777 | Colorectal cancer || id:ebi-a-GCST012879 | Weighted mode | 47 | 0.102667827 | 1.078(0.987-1.177) |
| COX10 | cg20170777 | Colorectal cancer || id:ebi-a-GCST012879 | Weighted median | 47 | 7.26E-06 | 1.081(1.045-1.119) |
| COX10 | cg20170777 | Colorectal cancer || id:ebi-a-GCST012879 | MR Egger | 47 | 0.221542494 | 1.168(0.914-1.494) |
| CASP9 | cg21858823 | Colorectal cancer || id:ebi-a-GCST012879 | Weighted median | 7 | 0.204470809 | 1.05(0.974-1.132) |
| CASP9 | cg21858823 | Colorectal cancer || id:ebi-a-GCST012879 | MR Egger | 7 | 0.803652643 | 1.114(0.496-2.5) |
| CASP9 | cg21858823 | Colorectal cancer || id:ebi-a-GCST012879 | Inverse variance weighted (fixed effects) | 7 | 0.043469523 | 1.064(1.002-1.129) |
| CASP9 | cg21858823 | Colorectal cancer || id:ebi-a-GCST012879 | Weighted mode | 7 | 0.574100634 | 1.048(0.898-1.222) |
| CASP9 | cg21858823 | Colorectal cancer || id:ebi-a-GCST012879 | Simple mode | 7 | 0.398807289 | 1.079(0.916-1.271) |
| BAD | cg23796481 | Colorectal cancer || id:ebi-a-GCST012879 | Inverse variance weighted (fixed effects) | 59 | 3.31E-98 | 1.109(1.099-1.12) |
| BAD | cg23796481 | Colorectal cancer || id:ebi-a-GCST012879 | Weighted mode | 59 | 0.000849638 | 1.098(1.042-1.157) |
| BAD | cg23796481 | Colorectal cancer || id:ebi-a-GCST012879 | Weighted median | 59 | 9.43E-16 | 1.099(1.074-1.124) |
| BAD | cg23796481 | Colorectal cancer || id:ebi-a-GCST012879 | MR Egger | 59 | 0.002199083 | 1.102(1.039-1.169) |
| BAD | cg23796481 | Colorectal cancer || id:ebi-a-GCST012879 | Simple mode | 59 | 0.00035074 | 1.117(1.055-1.183) |
| ABCD3 | cg24215727 | Colorectal cancer || id:ebi-a-GCST012879 | Simple mode | 92 | 0.047163254 | 1.087(1.002-1.178) |
| ABCD3 | cg24215727 | Colorectal cancer || id:ebi-a-GCST012879 | MR Egger | 92 | 0.019870505 | 1.71(1.097-2.665) |
| ABCD3 | cg24215727 | Colorectal cancer || id:ebi-a-GCST012879 | Weighted mode | 92 | 0.442979758 | 1.029(0.957-1.105) |
| ABCD3 | cg24215727 | Colorectal cancer || id:ebi-a-GCST012879 | Weighted median | 92 | 2.77E-05 | 1.038(1.02-1.056) |
| ABCD3 | cg24215727 | Colorectal cancer || id:ebi-a-GCST012879 | Inverse variance weighted (fixed effects) | 92 | 1.12E-10 | 1.063(1.044-1.083) |
| CISD3 | cg27492942 | Colorectal cancer || id:ebi-a-GCST012879 | Weighted mode | 44 | 0.015514017 | 1.061(1.013-1.111) |
| CISD3 | cg27492942 | Colorectal cancer || id:ebi-a-GCST012879 | Simple mode | 44 | 0.023200092 | 1.066(1.011-1.124) |
| CISD3 | cg27492942 | Colorectal cancer || id:ebi-a-GCST012879 | Weighted median | 44 | 0.000763897 | 1.058(1.024-1.093) |
| CISD3 | cg27492942 | Colorectal cancer || id:ebi-a-GCST012879 | Inverse variance weighted (fixed effects) | 44 | 3.30E-09 | 1.067(1.044-1.09) |
| CISD3 | cg27492942 | Colorectal cancer || id:ebi-a-GCST012879 | MR Egger | 44 | 0.696139972 | 1.016(0.939-1.099) |
| SLC25A30 | cg00139037 | Colorectal cancer || id:ebi-a-GCST012880 | MR Egger | 80 | 0.162585384 | 1.269(0.911-1.766) |
| SLC25A30 | cg00139037 | Colorectal cancer || id:ebi-a-GCST012880 | Weighted mode | 80 | 0.634048516 | 0.976(0.883-1.079) |
| SLC25A30 | cg00139037 | Colorectal cancer || id:ebi-a-GCST012880 | Weighted median | 80 | 0.044517211 | 0.973(0.948-0.999) |
| SLC25A30 | cg00139037 | Colorectal cancer || id:ebi-a-GCST012880 | Inverse variance weighted (fixed effects) | 80 | 0.067724997 | 0.968(0.934-1.002) |
| SLC25A30 | cg00139037 | Colorectal cancer || id:ebi-a-GCST012880 | Simple mode | 80 | 0.254308885 | 0.939(0.843-1.046) |
| MRPL32 | cg00365680 | Colorectal cancer || id:ebi-a-GCST012880 | MR Egger | 198 | 1.49E-10 | 0.897(0.869-0.925) |
| MRPL32 | cg00365680 | Colorectal cancer || id:ebi-a-GCST012880 | Weighted mode | 198 | 4.36E-10 | 0.895(0.865-0.925) |
| MRPL32 | cg00365680 | Colorectal cancer || id:ebi-a-GCST012880 | Weighted median | 198 | 2.00E-48 | 0.895(0.881-0.908) |
| MRPL32 | cg00365680 | Colorectal cancer || id:ebi-a-GCST012880 | Inverse variance weighted (fixed effects) | 198 | 5.88E-90 | 0.894(0.884-0.904) |
| MRPL32 | cg00365680 | Colorectal cancer || id:ebi-a-GCST012880 | Simple mode | 198 | 4.60E-08 | 0.895(0.861-0.93) |
| ACSF3 | cg02193283 | Colorectal cancer || id:ebi-a-GCST012880 | Weighted mode | 344 | 2.87E-06 | 0.878(0.832-0.926) |
| ACSF3 | cg02193283 | Colorectal cancer || id:ebi-a-GCST012880 | Simple mode | 344 | 1.49E-05 | 0.876(0.825-0.929) |
| ACSF3 | cg02193283 | Colorectal cancer || id:ebi-a-GCST012880 | Weighted median | 344 | 1.32E-37 | 0.889(0.873-0.905) |
| ACSF3 | cg02193283 | Colorectal cancer || id:ebi-a-GCST012880 | Inverse variance weighted (fixed effects) | 344 | 1.56E-78 | 0.889(0.878-0.9) |
| ACSF3 | cg02193283 | Colorectal cancer || id:ebi-a-GCST012880 | MR Egger | 344 | 4.14E-06 | 0.865(0.814-0.919) |
| ME3 | cg02493602 | Colorectal cancer || id:ebi-a-GCST012880 | MR Egger | 227 | 0.807571553 | 1.004(0.972-1.037) |
| ME3 | cg02493602 | Colorectal cancer || id:ebi-a-GCST012880 | Weighted median | 227 | 0.772921363 | 0.998(0.982-1.013) |
| ME3 | cg02493602 | Colorectal cancer || id:ebi-a-GCST012880 | Inverse variance weighted (fixed effects) | 227 | 0.038657158 | 0.988(0.977-0.999) |
| ME3 | cg02493602 | Colorectal cancer || id:ebi-a-GCST012880 | Simple mode | 227 | 0.378576561 | 1.019(0.978-1.061) |
| ME3 | cg02493602 | Colorectal cancer || id:ebi-a-GCST012880 | Weighted mode | 227 | 0.766056062 | 1.005(0.973-1.038) |
| SCP2 | cg03579872 | Colorectal cancer || id:ebi-a-GCST012880 | MR Egger | 318 | 0.574107824 | 0.987(0.941-1.034) |
| SCP2 | cg03579872 | Colorectal cancer || id:ebi-a-GCST012880 | Weighted median | 318 | 1.58E-23 | 0.936(0.924-0.948) |
| SCP2 | cg03579872 | Colorectal cancer || id:ebi-a-GCST012880 | Weighted mode | 318 | 0.010128905 | 0.952(0.917-0.988) |
| SCP2 | cg03579872 | Colorectal cancer || id:ebi-a-GCST012880 | Inverse variance weighted (fixed effects) | 318 | 4.99E-61 | 0.925(0.917-0.934) |
| SCP2 | cg03579872 | Colorectal cancer || id:ebi-a-GCST012880 | Simple mode | 318 | 9.07E-05 | 0.912(0.872-0.955) |
| ACSF3 | cg04308346 | Colorectal cancer || id:ebi-a-GCST012880 | Inverse variance weighted (fixed effects) | 10 | 0.017366206 | 1.116(1.019-1.221) |
| ACSF3 | cg04308346 | Colorectal cancer || id:ebi-a-GCST012880 | Simple mode | 10 | 0.391661643 | 1.122(0.873-1.441) |
| ACSF3 | cg04308346 | Colorectal cancer || id:ebi-a-GCST012880 | MR Egger | 10 | 0.931450743 | 0.863(0.033-22.431) |
| ACSF3 | cg04308346 | Colorectal cancer || id:ebi-a-GCST012880 | Weighted median | 10 | 0.041844509 | 1.112(1.004-1.232) |
| ACSF3 | cg04308346 | Colorectal cancer || id:ebi-a-GCST012880 | Weighted mode | 10 | 0.404112074 | 1.113(0.876-1.415) |
| MSRB2 | cg07355157 | Colorectal cancer || id:ebi-a-GCST012880 | Weighted mode | 158 | 0.286364909 | 1.012(0.99-1.033) |
| MSRB2 | cg07355157 | Colorectal cancer || id:ebi-a-GCST012880 | MR Egger | 158 | 9.77E-06 | 1.06(1.034-1.087) |
| MSRB2 | cg07355157 | Colorectal cancer || id:ebi-a-GCST012880 | Simple mode | 158 | 6.81E-17 | 0.871(0.846-0.897) |
| MSRB2 | cg07355157 | Colorectal cancer || id:ebi-a-GCST012880 | Inverse variance weighted (fixed effects) | 158 | 3.80E-08 | 0.967(0.955-0.978) |
| MSRB2 | cg07355157 | Colorectal cancer || id:ebi-a-GCST012880 | Weighted median | 158 | 0.293109989 | 1.01(0.991-1.029) |
| CYB5R3 | cg08690876 | Colorectal cancer || id:ebi-a-GCST012880 | Weighted mode | 354 | 0.001925337 | 1.043(1.016-1.07) |
| CYB5R3 | cg08690876 | Colorectal cancer || id:ebi-a-GCST012880 | Simple mode | 354 | 0.000589896 | 1.058(1.025-1.092) |
| CYB5R3 | cg08690876 | Colorectal cancer || id:ebi-a-GCST012880 | MR Egger | 354 | 6.43E-06 | 1.056(1.032-1.081) |
| CYB5R3 | cg08690876 | Colorectal cancer || id:ebi-a-GCST012880 | Inverse variance weighted (fixed effects) | 354 | 4.62E-21 | 1.042(1.033-1.051) |
| CYB5R3 | cg08690876 | Colorectal cancer || id:ebi-a-GCST012880 | Weighted median | 354 | 2.14E-10 | 1.04(1.027-1.052) |
| LIPT2 | cg11417029 | Colorectal cancer || id:ebi-a-GCST012880 | Inverse variance weighted (fixed effects) | 113 | 4.88E-86 | 1.301(1.267-1.335) |
| LIPT2 | cg11417029 | Colorectal cancer || id:ebi-a-GCST012880 | Weighted median | 113 | 2.69E-18 | 1.285(1.214-1.359) |
| LIPT2 | cg11417029 | Colorectal cancer || id:ebi-a-GCST012880 | Simple mode | 113 | 0.020464045 | 1.327(1.048-1.681) |
| LIPT2 | cg11417029 | Colorectal cancer || id:ebi-a-GCST012880 | MR Egger | 113 | 0.422336377 | 1.24(0.734-2.096) |
| LIPT2 | cg11417029 | Colorectal cancer || id:ebi-a-GCST012880 | Weighted mode | 113 | 0.000191765 | 1.327(1.149-1.533) |
| MRPL28 | cg12437481 | Colorectal cancer || id:ebi-a-GCST012880 | Simple mode | 265 | 0.164425788 | 0.987(0.969-1.005) |
| MRPL28 | cg12437481 | Colorectal cancer || id:ebi-a-GCST012880 | MR Egger | 265 | 0.000367912 | 0.979(0.967-0.99) |
| MRPL28 | cg12437481 | Colorectal cancer || id:ebi-a-GCST012880 | Weighted median | 265 | 0.075391236 | 0.991(0.981-1.001) |
| MRPL28 | cg12437481 | Colorectal cancer || id:ebi-a-GCST012880 | Weighted mode | 265 | 0.02526578 | 0.984(0.971-0.998) |
| MRPL28 | cg12437481 | Colorectal cancer || id:ebi-a-GCST012880 | Inverse variance weighted (fixed effects) | 265 | 0.003633766 | 1.01(1.003-1.016) |
| ACADS | cg13914990 | Colorectal cancer || id:ebi-a-GCST012880 | MR Egger | 624 | 0.759971039 | 0.996(0.971-1.021) |
| ACADS | cg13914990 | Colorectal cancer || id:ebi-a-GCST012880 | Weighted median | 624 | 4.85E-11 | 1.036(1.025-1.047) |
| ACADS | cg13914990 | Colorectal cancer || id:ebi-a-GCST012880 | Weighted mode | 624 | 0.001433414 | 1.037(1.014-1.061) |
| ACADS | cg13914990 | Colorectal cancer || id:ebi-a-GCST012880 | Inverse variance weighted (fixed effects) | 624 | 4.31E-20 | 1.036(1.028-1.044) |
| ACADS | cg13914990 | Colorectal cancer || id:ebi-a-GCST012880 | Simple mode | 624 | 0.006969273 | 1.041(1.011-1.072) |
| NSUN4 | cg14993813 | Colorectal cancer || id:ebi-a-GCST012880 | Weighted mode | 251 | 0.000191732 | 1.125(1.058-1.196) |
| NSUN4 | cg14993813 | Colorectal cancer || id:ebi-a-GCST012880 | Simple mode | 251 | 0.000522037 | 1.125(1.053-1.201) |
| NSUN4 | cg14993813 | Colorectal cancer || id:ebi-a-GCST012880 | Inverse variance weighted (fixed effects) | 251 | 1.61E-76 | 1.13(1.115-1.144) |
| NSUN4 | cg14993813 | Colorectal cancer || id:ebi-a-GCST012880 | Weighted median | 251 | 8.40E-39 | 1.125(1.106-1.146) |
| NSUN4 | cg14993813 | Colorectal cancer || id:ebi-a-GCST012880 | MR Egger | 251 | 0.072652398 | 1.091(0.992-1.199) |
| COX15 | cg17885402 | Colorectal cancer || id:ebi-a-GCST012880 | MR Egger | 306 | 2.78E-07 | 0.89(0.852-0.929) |
| COX15 | cg17885402 | Colorectal cancer || id:ebi-a-GCST012880 | Inverse variance weighted (fixed effects) | 306 | 4.86E-238 | 0.811(0.801-0.822) |
| COX15 | cg17885402 | Colorectal cancer || id:ebi-a-GCST012880 | Simple mode | 306 | 4.02E-08 | 0.785(0.722-0.854) |
| COX15 | cg17885402 | Colorectal cancer || id:ebi-a-GCST012880 | Weighted median | 306 | 2.68E-64 | 0.795(0.775-0.817) |
| COX15 | cg17885402 | Colorectal cancer || id:ebi-a-GCST012880 | Weighted mode | 306 | 5.63E-10 | 0.785(0.729-0.846) |
| PNKD | cg18259342 | Colorectal cancer || id:ebi-a-GCST012880 | Inverse variance weighted (fixed effects) | 362 | 1.63E-46 | 1.04(1.034-1.045) |
| PNKD | cg18259342 | Colorectal cancer || id:ebi-a-GCST012880 | Simple mode | 362 | 0.000175268 | 1.039(1.018-1.059) |
| PNKD | cg18259342 | Colorectal cancer || id:ebi-a-GCST012880 | Weighted median | 362 | 1.26E-21 | 1.037(1.03-1.045) |
| PNKD | cg18259342 | Colorectal cancer || id:ebi-a-GCST012880 | MR Egger | 362 | 1.33E-07 | 1.05(1.031-1.069) |
| PNKD | cg18259342 | Colorectal cancer || id:ebi-a-GCST012880 | Weighted mode | 362 | 8.44E-05 | 1.039(1.019-1.058) |
| COX10 | cg20170777 | Colorectal cancer || id:ebi-a-GCST012880 | Inverse variance weighted (fixed effects) | 47 | 3.89E-20 | 1.235(1.181-1.292) |
| COX10 | cg20170777 | Colorectal cancer || id:ebi-a-GCST012880 | MR Egger | 47 | 0.045193715 | 1.601(1.023-2.504) |
| COX10 | cg20170777 | Colorectal cancer || id:ebi-a-GCST012880 | Weighted median | 47 | 4.91E-07 | 1.207(1.122-1.299) |
| COX10 | cg20170777 | Colorectal cancer || id:ebi-a-GCST012880 | Simple mode | 47 | 0.041534065 | 1.19(1.011-1.399) |
| COX10 | cg20170777 | Colorectal cancer || id:ebi-a-GCST012880 | Weighted mode | 47 | 0.039239239 | 1.19(1.013-1.396) |
| CASP9 | cg21858823 | Colorectal cancer || id:ebi-a-GCST012880 | Weighted mode | 7 | 0.540257613 | 0.921(0.718-1.181) |
| CASP9 | cg21858823 | Colorectal cancer || id:ebi-a-GCST012880 | Simple mode | 7 | 0.556441564 | 0.919(0.705-1.199) |
| CASP9 | cg21858823 | Colorectal cancer || id:ebi-a-GCST012880 | Inverse variance weighted (fixed effects) | 7 | 0.129573981 | 0.918(0.821-1.025) |
| CASP9 | cg21858823 | Colorectal cancer || id:ebi-a-GCST012880 | MR Egger | 7 | 0.982911427 | 1.017(0.228-4.544) |
| CASP9 | cg21858823 | Colorectal cancer || id:ebi-a-GCST012880 | Weighted median | 7 | 0.241926399 | 0.922(0.804-1.057) |
| BAD | cg23796481 | Colorectal cancer || id:ebi-a-GCST012880 | Simple mode | 59 | 0.006650106 | 0.923(0.873-0.976) |
| BAD | cg23796481 | Colorectal cancer || id:ebi-a-GCST012880 | Inverse variance weighted (fixed effects) | 59 | 1.71E-12 | 0.939(0.922-0.955) |
| BAD | cg23796481 | Colorectal cancer || id:ebi-a-GCST012880 | Weighted mode | 59 | 0.101157758 | 0.957(0.908-1.008) |
| BAD | cg23796481 | Colorectal cancer || id:ebi-a-GCST012880 | Weighted median | 59 | 0.000144538 | 0.954(0.931-0.978) |
| BAD | cg23796481 | Colorectal cancer || id:ebi-a-GCST012880 | MR Egger | 59 | 0.025512257 | 0.882(0.792-0.982) |
| ABCD3 | cg24215727 | Colorectal cancer || id:ebi-a-GCST012880 | Weighted mode | 94 | 0.28215811 | 0.942(0.844-1.05) |
| ABCD3 | cg24215727 | Colorectal cancer || id:ebi-a-GCST012880 | Inverse variance weighted (fixed effects) | 94 | 0.289500444 | 0.982(0.95-1.016) |
| ABCD3 | cg24215727 | Colorectal cancer || id:ebi-a-GCST012880 | Simple mode | 94 | 0.277770024 | 0.94(0.841-1.051) |
| ABCD3 | cg24215727 | Colorectal cancer || id:ebi-a-GCST012880 | MR Egger | 94 | 0.008488523 | 0.346(0.16-0.75) |
| ABCD3 | cg24215727 | Colorectal cancer || id:ebi-a-GCST012880 | Weighted median | 94 | 0.22495066 | 0.983(0.957-1.011) |
| CISD3 | cg27492942 | Colorectal cancer || id:ebi-a-GCST012880 | Weighted mode | 44 | 0.840869716 | 1.01(0.92-1.108) |
| CISD3 | cg27492942 | Colorectal cancer || id:ebi-a-GCST012880 | MR Egger | 44 | 0.774040617 | 0.979(0.849-1.13) |
| CISD3 | cg27492942 | Colorectal cancer || id:ebi-a-GCST012880 | Inverse variance weighted (fixed effects) | 44 | 0.083264846 | 0.966(0.93-1.005) |
| CISD3 | cg27492942 | Colorectal cancer || id:ebi-a-GCST012880 | Simple mode | 44 | 0.203227257 | 0.936(0.846-1.035) |
| CISD3 | cg27492942 | Colorectal cancer || id:ebi-a-GCST012880 | Weighted median | 44 | 0.242915726 | 0.971(0.923-1.02) |
| SLC25A30 | cg00139037 | Colorectal cancer (Firth correction) || id:ebi-a-GCST90013862 | Simple mode | 76 | 0.133708577 | 1.296(0.927-1.812) |
| SLC25A30 | cg00139037 | Colorectal cancer (Firth correction) || id:ebi-a-GCST90013862 | Weighted mode | 76 | 0.121348374 | 1.298(0.937-1.798) |
| SLC25A30 | cg00139037 | Colorectal cancer (Firth correction) || id:ebi-a-GCST90013862 | Weighted median | 76 | 3.89E-45 | 1.291(1.246-1.338) |
| SLC25A30 | cg00139037 | Colorectal cancer (Firth correction) || id:ebi-a-GCST90013862 | Inverse variance weighted (fixed effects) | 76 | 3.49E-88 | 1.294(1.262-1.328) |
| SLC25A30 | cg00139037 | Colorectal cancer (Firth correction) || id:ebi-a-GCST90013862 | MR Egger | 76 | 0.00374135 | 1.44(1.134-1.827) |
| MRPL32 | cg00365680 | Colorectal cancer (Firth correction) || id:ebi-a-GCST90013862 | Weighted mode | 164 | 0.756965091 | 0.996(0.972-1.021) |
| MRPL32 | cg00365680 | Colorectal cancer (Firth correction) || id:ebi-a-GCST90013862 | Simple mode | 164 | 0.73377204 | 0.995(0.969-1.022) |
| MRPL32 | cg00365680 | Colorectal cancer (Firth correction) || id:ebi-a-GCST90013862 | Inverse variance weighted (fixed effects) | 164 | 1.31E-08 | 0.976(0.968-0.984) |
| MRPL32 | cg00365680 | Colorectal cancer (Firth correction) || id:ebi-a-GCST90013862 | Weighted median | 164 | 0.003916902 | 0.984(0.974-0.995) |
| MRPL32 | cg00365680 | Colorectal cancer (Firth correction) || id:ebi-a-GCST90013862 | MR Egger | 164 | 0.476808389 | 1.009(0.984-1.034) |
| ACSF3 | cg02193283 | Colorectal cancer (Firth correction) || id:ebi-a-GCST90013862 | Weighted mode | 295 | 9.26E-06 | 0.912(0.876-0.949) |
| ACSF3 | cg02193283 | Colorectal cancer (Firth correction) || id:ebi-a-GCST90013862 | Weighted median | 295 | 1.30E-36 | 0.916(0.904-0.929) |
| ACSF3 | cg02193283 | Colorectal cancer (Firth correction) || id:ebi-a-GCST90013862 | Inverse variance weighted (fixed effects) | 295 | 1.97E-71 | 0.919(0.91-0.927) |
| ACSF3 | cg02193283 | Colorectal cancer (Firth correction) || id:ebi-a-GCST90013862 | Simple mode | 295 | 7.23E-05 | 0.917(0.88-0.957) |
| ACSF3 | cg02193283 | Colorectal cancer (Firth correction) || id:ebi-a-GCST90013862 | MR Egger | 295 | 8.72E-08 | 0.88(0.841-0.921) |
| ME3 | cg02493602 | Colorectal cancer (Firth correction) || id:ebi-a-GCST90013862 | Weighted mode | 186 | 0.729696037 | 0.996(0.971-1.021) |
| ME3 | cg02493602 | Colorectal cancer (Firth correction) || id:ebi-a-GCST90013862 | MR Egger | 186 | 0.000193258 | 1.053(1.025-1.081) |
| ME3 | cg02493602 | Colorectal cancer (Firth correction) || id:ebi-a-GCST90013862 | Simple mode | 186 | 0.000142868 | 0.93(0.897-0.965) |
| ME3 | cg02493602 | Colorectal cancer (Firth correction) || id:ebi-a-GCST90013862 | Inverse variance weighted (fixed effects) | 186 | 9.09E-20 | 0.958(0.949-0.967) |
| ME3 | cg02493602 | Colorectal cancer (Firth correction) || id:ebi-a-GCST90013862 | Weighted median | 186 | 0.076987166 | 0.987(0.973-1.001) |
| SCP2 | cg03579872 | Colorectal cancer (Firth correction) || id:ebi-a-GCST90013862 | Inverse variance weighted (fixed effects) | 58 | 0.005031058 | 1.035(1.01-1.059) |
| SCP2 | cg03579872 | Colorectal cancer (Firth correction) || id:ebi-a-GCST90013862 | MR Egger | 58 | 0.350188467 | 1.086(0.915-1.29) |
| SCP2 | cg03579872 | Colorectal cancer (Firth correction) || id:ebi-a-GCST90013862 | Weighted median | 58 | 0.614341352 | 0.994(0.969-1.019) |
| SCP2 | cg03579872 | Colorectal cancer (Firth correction) || id:ebi-a-GCST90013862 | Simple mode | 58 | 0.716036748 | 0.988(0.927-1.053) |
| SCP2 | cg03579872 | Colorectal cancer (Firth correction) || id:ebi-a-GCST90013862 | Weighted mode | 58 | 0.712301374 | 0.989(0.932-1.049) |
| ACSF3 | cg04308346 | Colorectal cancer (Firth correction) || id:ebi-a-GCST90013862 | Weighted mode | 8 | 0.653562965 | 1.038(0.888-1.214) |
| ACSF3 | cg04308346 | Colorectal cancer (Firth correction) || id:ebi-a-GCST90013862 | Simple mode | 8 | 0.6622167 | 1.038(0.883-1.222) |
| ACSF3 | cg04308346 | Colorectal cancer (Firth correction) || id:ebi-a-GCST90013862 | Inverse variance weighted (fixed effects) | 8 | 0.187847446 | 1.05(0.977-1.129) |
| ACSF3 | cg04308346 | Colorectal cancer (Firth correction) || id:ebi-a-GCST90013862 | MR Egger | 8 | 0.850315897 | 1.273(0.115-14.09) |
| ACSF3 | cg04308346 | Colorectal cancer (Firth correction) || id:ebi-a-GCST90013862 | Weighted median | 8 | 0.312147059 | 1.039(0.964-1.12) |
| MSRB2 | cg07355157 | Colorectal cancer (Firth correction) || id:ebi-a-GCST90013862 | Weighted mode | 147 | 0.002767005 | 1.027(1.009-1.044) |
| MSRB2 | cg07355157 | Colorectal cancer (Firth correction) || id:ebi-a-GCST90013862 | Simple mode | 147 | 0.134695837 | 1.019(0.994-1.044) |
| MSRB2 | cg07355157 | Colorectal cancer (Firth correction) || id:ebi-a-GCST90013862 | Inverse variance weighted (fixed effects) | 147 | 5.14E-13 | 1.034(1.025-1.043) |
| MSRB2 | cg07355157 | Colorectal cancer (Firth correction) || id:ebi-a-GCST90013862 | Weighted median | 147 | 5.27E-05 | 1.027(1.014-1.041) |
| MSRB2 | cg07355157 | Colorectal cancer (Firth correction) || id:ebi-a-GCST90013862 | MR Egger | 147 | 5.22E-07 | 1.052(1.032-1.072) |
| CYB5R3 | cg08690876 | Colorectal cancer (Firth correction) || id:ebi-a-GCST90013862 | Inverse variance weighted (fixed effects) | 328 | 0.012247732 | 0.992(0.986-0.998) |
| CYB5R3 | cg08690876 | Colorectal cancer (Firth correction) || id:ebi-a-GCST90013862 | Weighted median | 328 | 0.388520774 | 0.996(0.986-1.005) |
| CYB5R3 | cg08690876 | Colorectal cancer (Firth correction) || id:ebi-a-GCST90013862 | MR Egger | 328 | 0.792808172 | 1.002(0.985-1.02) |
| CYB5R3 | cg08690876 | Colorectal cancer (Firth correction) || id:ebi-a-GCST90013862 | Weighted mode | 328 | 0.008900762 | 0.972(0.952-0.993) |
| CYB5R3 | cg08690876 | Colorectal cancer (Firth correction) || id:ebi-a-GCST90013862 | Simple mode | 328 | 0.022425656 | 1.029(1.004-1.055) |
| ECHDC2 | cg09128567 | Colorectal cancer (Firth correction) || id:ebi-a-GCST90013862 | Weighted mode | 250 | 0.005482966 | 1.218(1.061-1.398) |
| ECHDC2 | cg09128567 | Colorectal cancer (Firth correction) || id:ebi-a-GCST90013862 | Simple mode | 250 | 0.009957536 | 1.217(1.049-1.411) |
| ECHDC2 | cg09128567 | Colorectal cancer (Firth correction) || id:ebi-a-GCST90013862 | Inverse variance weighted (fixed effects) | 250 | 4.77E-290 | 1.221(1.208-1.234) |
| ECHDC2 | cg09128567 | Colorectal cancer (Firth correction) || id:ebi-a-GCST90013862 | Weighted median | 250 | 3.48E-93 | 1.218(1.195-1.241) |
| ECHDC2 | cg09128567 | Colorectal cancer (Firth correction) || id:ebi-a-GCST90013862 | MR Egger | 250 | 5.98E-14 | 1.351(1.255-1.455) |
| LIPT2 | cg11417029 | Colorectal cancer (Firth correction) || id:ebi-a-GCST90013862 | MR Egger | 97 | 0.196042413 | 1.313(0.871-1.98) |
| LIPT2 | cg11417029 | Colorectal cancer (Firth correction) || id:ebi-a-GCST90013862 | Simple mode | 97 | 0.019118064 | 1.275(1.044-1.556) |
| LIPT2 | cg11417029 | Colorectal cancer (Firth correction) || id:ebi-a-GCST90013862 | Weighted mode | 97 | 0.002416848 | 1.274(1.094-1.484) |
| LIPT2 | cg11417029 | Colorectal cancer (Firth correction) || id:ebi-a-GCST90013862 | Weighted median | 97 | 2.04E-26 | 1.26(1.207-1.315) |
| LIPT2 | cg11417029 | Colorectal cancer (Firth correction) || id:ebi-a-GCST90013862 | Inverse variance weighted (fixed effects) | 97 | 2.41E-111 | 1.26(1.234-1.285) |
| MRPL28 | cg12437481 | Colorectal cancer (Firth correction) || id:ebi-a-GCST90013862 | Simple mode | 243 | 0.00030935 | 1.022(1.01-1.033) |
| MRPL28 | cg12437481 | Colorectal cancer (Firth correction) || id:ebi-a-GCST90013862 | Weighted median | 243 | 1.94E-08 | 1.019(1.013-1.026) |
| MRPL28 | cg12437481 | Colorectal cancer (Firth correction) || id:ebi-a-GCST90013862 | Inverse variance weighted (fixed effects) | 243 | 2.40E-11 | 1.016(1.012-1.021) |
| MRPL28 | cg12437481 | Colorectal cancer (Firth correction) || id:ebi-a-GCST90013862 | MR Egger | 243 | 3.00E-06 | 1.022(1.013-1.03) |
| MRPL28 | cg12437481 | Colorectal cancer (Firth correction) || id:ebi-a-GCST90013862 | Weighted mode | 243 | 6.02E-05 | 1.02(1.01-1.03) |
| ACADS | cg13914990 | Colorectal cancer (Firth correction) || id:ebi-a-GCST90013862 | MR Egger | 577 | 8.93E-05 | 1.037(1.018-1.056) |
| ACADS | cg13914990 | Colorectal cancer (Firth correction) || id:ebi-a-GCST90013862 | Simple mode | 577 | 0.001021318 | 1.043(1.017-1.069) |
| ACADS | cg13914990 | Colorectal cancer (Firth correction) || id:ebi-a-GCST90013862 | Weighted mode | 577 | 1.04E-05 | 1.038(1.021-1.055) |
| ACADS | cg13914990 | Colorectal cancer (Firth correction) || id:ebi-a-GCST90013862 | Inverse variance weighted (fixed effects) | 577 | 3.85E-65 | 1.048(1.043-1.054) |
| ACADS | cg13914990 | Colorectal cancer (Firth correction) || id:ebi-a-GCST90013862 | Weighted median | 577 | 4.22E-17 | 1.038(1.029-1.046) |
| NSUN4 | cg14993813 | Colorectal cancer (Firth correction) || id:ebi-a-GCST90013862 | MR Egger | 230 | 5.43E-05 | 1.159(1.08-1.243) |
| NSUN4 | cg14993813 | Colorectal cancer (Firth correction) || id:ebi-a-GCST90013862 | Simple mode | 230 | 3.10E-06 | 1.207(1.117-1.304) |
| NSUN4 | cg14993813 | Colorectal cancer (Firth correction) || id:ebi-a-GCST90013862 | Inverse variance weighted (fixed effects) | 230 | 0 | 1.21(1.199-1.222) |
| NSUN4 | cg14993813 | Colorectal cancer (Firth correction) || id:ebi-a-GCST90013862 | Weighted mode | 230 | 2.61E-06 | 1.207(1.118-1.303) |
| NSUN4 | cg14993813 | Colorectal cancer (Firth correction) || id:ebi-a-GCST90013862 | Weighted median | 230 | 3.64E-72 | 1.207(1.183-1.232) |
| COX15 | cg17885402 | Colorectal cancer (Firth correction) || id:ebi-a-GCST90013862 | MR Egger | 269 | 3.89E-05 | 1.08(1.042-1.119) |
| COX15 | cg17885402 | Colorectal cancer (Firth correction) || id:ebi-a-GCST90013862 | Simple mode | 269 | 5.25E-40 | 0.758(0.732-0.785) |
| COX15 | cg17885402 | Colorectal cancer (Firth correction) || id:ebi-a-GCST90013862 | Weighted median | 269 | 7.39E-12 | 0.947(0.932-0.962) |
| COX15 | cg17885402 | Colorectal cancer (Firth correction) || id:ebi-a-GCST90013862 | Weighted mode | 269 | 0.000123327 | 0.947(0.921-0.973) |
| PNKD | cg18259342 | Colorectal cancer (Firth correction) || id:ebi-a-GCST90013862 | Weighted median | 340 | 1.85E-94 | 1.091(1.082-1.1) |
| PNKD | cg18259342 | Colorectal cancer (Firth correction) || id:ebi-a-GCST90013862 | MR Egger | 340 | 5.83E-39 | 1.105(1.091-1.12) |
| PNKD | cg18259342 | Colorectal cancer (Firth correction) || id:ebi-a-GCST90013862 | Inverse variance weighted (fixed effects) | 340 | 0 | 1.09(1.086-1.094) |
| PNKD | cg18259342 | Colorectal cancer (Firth correction) || id:ebi-a-GCST90013862 | Weighted mode | 340 | 1.71E-13 | 1.096(1.071-1.122) |
| PNKD | cg18259342 | Colorectal cancer (Firth correction) || id:ebi-a-GCST90013862 | Simple mode | 340 | 8.13E-13 | 1.096(1.07-1.123) |
| COX10 | cg20170777 | Colorectal cancer (Firth correction) || id:ebi-a-GCST90013862 | Weighted median | 40 | 5.46E-06 | 0.887(0.843-0.934) |
| COX10 | cg20170777 | Colorectal cancer (Firth correction) || id:ebi-a-GCST90013862 | MR Egger | 40 | 0.010924208 | 0.631(0.45-0.884) |
| COX10 | cg20170777 | Colorectal cancer (Firth correction) || id:ebi-a-GCST90013862 | Simple mode | 40 | 0.04996044 | 0.89(0.794-0.996) |
| COX10 | cg20170777 | Colorectal cancer (Firth correction) || id:ebi-a-GCST90013862 | Weighted mode | 40 | 0.053080711 | 0.89(0.794-0.998) |
| COX10 | cg20170777 | Colorectal cancer (Firth correction) || id:ebi-a-GCST90013862 | Inverse variance weighted (fixed effects) | 40 | 2.34E-13 | 0.88(0.85-0.91) |
| BAD | cg23796481 | Colorectal cancer (Firth correction) || id:ebi-a-GCST90013862 | Weighted mode | 54 | 0.003079743 | 1.124(1.044-1.21) |
| BAD | cg23796481 | Colorectal cancer (Firth correction) || id:ebi-a-GCST90013862 | MR Egger | 54 | 0.471735931 | 1.036(0.942-1.14) |
| BAD | cg23796481 | Colorectal cancer (Firth correction) || id:ebi-a-GCST90013862 | Weighted median | 54 | 5.06E-13 | 1.131(1.094-1.17) |
| BAD | cg23796481 | Colorectal cancer (Firth correction) || id:ebi-a-GCST90013862 | Inverse variance weighted (fixed effects) | 54 | 2.52E-102 | 1.151(1.136-1.166) |
| BAD | cg23796481 | Colorectal cancer (Firth correction) || id:ebi-a-GCST90013862 | Simple mode | 54 | 0.00011905 | 1.169(1.086-1.258) |
| ABCD3 | cg24215727 | Colorectal cancer (Firth correction) || id:ebi-a-GCST90013862 | Weighted median | 77 | 0.000753491 | 0.96(0.937-0.983) |
| ABCD3 | cg24215727 | Colorectal cancer (Firth correction) || id:ebi-a-GCST90013862 | Inverse variance weighted (fixed effects) | 77 | 0.064731184 | 0.976(0.95-1.002) |
| ABCD3 | cg24215727 | Colorectal cancer (Firth correction) || id:ebi-a-GCST90013862 | MR Egger | 77 | 0.001385652 | 0.344(0.183-0.646) |
| ABCD3 | cg24215727 | Colorectal cancer (Firth correction) || id:ebi-a-GCST90013862 | Weighted mode | 77 | 0.664098281 | 0.98(0.895-1.073) |
| ABCD3 | cg24215727 | Colorectal cancer (Firth correction) || id:ebi-a-GCST90013862 | Simple mode | 77 | 0.16508527 | 0.934(0.849-1.028) |
| CISD3 | cg27492942 | Colorectal cancer (Firth correction) || id:ebi-a-GCST90013862 | Inverse variance weighted (fixed effects) | 39 | 2.31E-12 | 0.901(0.875-0.928) |
| CISD3 | cg27492942 | Colorectal cancer (Firth correction) || id:ebi-a-GCST90013862 | Simple mode | 39 | 0.021538825 | 0.889(0.807-0.979) |
| CISD3 | cg27492942 | Colorectal cancer (Firth correction) || id:ebi-a-GCST90013862 | Weighted mode | 39 | 0.003410888 | 0.886(0.821-0.956) |
| CISD3 | cg27492942 | Colorectal cancer (Firth correction) || id:ebi-a-GCST90013862 | MR Egger | 39 | 0.009071618 | 0.861(0.774-0.958) |
| CISD3 | cg27492942 | Colorectal cancer (Firth correction) || id:ebi-a-GCST90013862 | Weighted median | 39 | 1.74E-06 | 0.89(0.849-0.934) |
| SLC25A30 | cg00139037 | Colorectal cancer (SPA correction) || id:ebi-a-GCST90013866 | Weighted mode | 77 | 0.109624151 | 1.3(0.946-1.786) |
| SLC25A30 | cg00139037 | Colorectal cancer (SPA correction) || id:ebi-a-GCST90013866 | Simple mode | 77 | 0.14689928 | 1.297(0.916-1.837) |
| SLC25A30 | cg00139037 | Colorectal cancer (SPA correction) || id:ebi-a-GCST90013866 | MR Egger | 77 | 0.003846919 | 1.435(1.132-1.819) |
| SLC25A30 | cg00139037 | Colorectal cancer (SPA correction) || id:ebi-a-GCST90013866 | Inverse variance weighted (fixed effects) | 77 | 1.08E-89 | 1.295(1.263-1.328) |
| SLC25A30 | cg00139037 | Colorectal cancer (SPA correction) || id:ebi-a-GCST90013866 | Weighted median | 77 | 1.31E-44 | 1.293(1.247-1.34) |
| MRPL32 | cg00365680 | Colorectal cancer (SPA correction) || id:ebi-a-GCST90013866 | Inverse variance weighted (fixed effects) | 164 | 1.26E-08 | 0.976(0.968-0.984) |
| MRPL32 | cg00365680 | Colorectal cancer (SPA correction) || id:ebi-a-GCST90013866 | MR Egger | 164 | 0.469111638 | 1.009(0.984-1.035) |
| MRPL32 | cg00365680 | Colorectal cancer (SPA correction) || id:ebi-a-GCST90013866 | Weighted median | 164 | 0.003929718 | 0.984(0.974-0.995) |
| MRPL32 | cg00365680 | Colorectal cancer (SPA correction) || id:ebi-a-GCST90013866 | Simple mode | 164 | 0.719821897 | 0.996(0.971-1.02) |
| MRPL32 | cg00365680 | Colorectal cancer (SPA correction) || id:ebi-a-GCST90013866 | Weighted mode | 164 | 0.781881476 | 0.996(0.97-1.023) |
| ACSF3 | cg02193283 | Colorectal cancer (SPA correction) || id:ebi-a-GCST90013866 | Weighted median | 295 | 1.13E-37 | 0.916(0.904-0.929) |
| ACSF3 | cg02193283 | Colorectal cancer (SPA correction) || id:ebi-a-GCST90013866 | Simple mode | 295 | 0.000121044 | 0.917(0.878-0.958) |
| ACSF3 | cg02193283 | Colorectal cancer (SPA correction) || id:ebi-a-GCST90013866 | Inverse variance weighted (fixed effects) | 295 | 2.05E-71 | 0.919(0.91-0.927) |
| ACSF3 | cg02193283 | Colorectal cancer (SPA correction) || id:ebi-a-GCST90013866 | Weighted mode | 295 | 8.96E-06 | 0.912(0.876-0.949) |
| ACSF3 | cg02193283 | Colorectal cancer (SPA correction) || id:ebi-a-GCST90013866 | MR Egger | 295 | 8.52E-08 | 0.88(0.841-0.921) |
| ME3 | cg02493602 | Colorectal cancer (SPA correction) || id:ebi-a-GCST90013866 | Weighted median | 186 | 0.084741297 | 0.987(0.973-1.002) |
| ME3 | cg02493602 | Colorectal cancer (SPA correction) || id:ebi-a-GCST90013866 | MR Egger | 186 | 0.000183174 | 1.053(1.025-1.081) |
| ME3 | cg02493602 | Colorectal cancer (SPA correction) || id:ebi-a-GCST90013866 | Inverse variance weighted (fixed effects) | 186 | 7.60E-20 | 0.958(0.949-0.967) |
| ME3 | cg02493602 | Colorectal cancer (SPA correction) || id:ebi-a-GCST90013866 | Simple mode | 186 | 9.12E-05 | 0.93(0.897-0.964) |
| ME3 | cg02493602 | Colorectal cancer (SPA correction) || id:ebi-a-GCST90013866 | Weighted mode | 186 | 0.752544362 | 0.996(0.972-1.021) |
| SCP2 | cg03579872 | Colorectal cancer (SPA correction) || id:ebi-a-GCST90013866 | Inverse variance weighted (fixed effects) | 308 | 8.23E-276 | 1.128(1.12-1.135) |
| SCP2 | cg03579872 | Colorectal cancer (SPA correction) || id:ebi-a-GCST90013866 | Simple mode | 308 | 0.000223472 | 1.139(1.064-1.22) |
| SCP2 | cg03579872 | Colorectal cancer (SPA correction) || id:ebi-a-GCST90013866 | Weighted mode | 308 | 0.000123711 | 1.141(1.068-1.219) |
| SCP2 | cg03579872 | Colorectal cancer (SPA correction) || id:ebi-a-GCST90013866 | MR Egger | 308 | 1.48E-36 | 1.294(1.25-1.34) |
| SCP2 | cg03579872 | Colorectal cancer (SPA correction) || id:ebi-a-GCST90013866 | Weighted median | 308 | 4.81E-69 | 1.121(1.107-1.135) |
| ACSF3 | cg04308346 | Colorectal cancer (SPA correction) || id:ebi-a-GCST90013866 | MR Egger | 8 | 0.850315897 | 1.273(0.115-14.09) |
| ACSF3 | cg04308346 | Colorectal cancer (SPA correction) || id:ebi-a-GCST90013866 | Weighted mode | 8 | 0.63774419 | 1.038(0.894-1.205) |
| ACSF3 | cg04308346 | Colorectal cancer (SPA correction) || id:ebi-a-GCST90013866 | Inverse variance weighted (fixed effects) | 8 | 0.187847446 | 1.05(0.977-1.129) |
| ACSF3 | cg04308346 | Colorectal cancer (SPA correction) || id:ebi-a-GCST90013866 | Simple mode | 8 | 0.650880648 | 1.038(0.888-1.215) |
| ACSF3 | cg04308346 | Colorectal cancer (SPA correction) || id:ebi-a-GCST90013866 | Weighted median | 8 | 0.326733908 | 1.039(0.962-1.123) |
| MSRB2 | cg07355157 | Colorectal cancer (SPA correction) || id:ebi-a-GCST90013866 | MR Egger | 147 | 5.31E-07 | 1.052(1.032-1.072) |
| MSRB2 | cg07355157 | Colorectal cancer (SPA correction) || id:ebi-a-GCST90013866 | Weighted mode | 147 | 0.00513913 | 1.027(1.008-1.045) |
| MSRB2 | cg07355157 | Colorectal cancer (SPA correction) || id:ebi-a-GCST90013866 | Weighted median | 147 | 5.90E-05 | 1.027(1.014-1.041) |
| MSRB2 | cg07355157 | Colorectal cancer (SPA correction) || id:ebi-a-GCST90013866 | Simple mode | 147 | 0.137602096 | 1.019(0.994-1.044) |
| MSRB2 | cg07355157 | Colorectal cancer (SPA correction) || id:ebi-a-GCST90013866 | Inverse variance weighted (fixed effects) | 147 | 5.38E-13 | 1.034(1.025-1.043) |
| CYB5R3 | cg08690876 | Colorectal cancer (SPA correction) || id:ebi-a-GCST90013866 | MR Egger | 319 | 0.873404708 | 0.999(0.981-1.016) |
| CYB5R3 | cg08690876 | Colorectal cancer (SPA correction) || id:ebi-a-GCST90013866 | Weighted median | 319 | 0.282387939 | 0.995(0.985-1.004) |
| CYB5R3 | cg08690876 | Colorectal cancer (SPA correction) || id:ebi-a-GCST90013866 | Inverse variance weighted (fixed effects) | 319 | 0.012311464 | 0.992(0.985-0.998) |
| CYB5R3 | cg08690876 | Colorectal cancer (SPA correction) || id:ebi-a-GCST90013866 | Weighted mode | 319 | 0.015869375 | 0.972(0.951-0.995) |
| CYB5R3 | cg08690876 | Colorectal cancer (SPA correction) || id:ebi-a-GCST90013866 | Simple mode | 319 | 0.023869551 | 1.03(1.004-1.056) |
| LIPT2 | cg11417029 | Colorectal cancer (SPA correction) || id:ebi-a-GCST90013866 | Simple mode | 97 | 0.023768004 | 1.274(1.036-1.566) |
| LIPT2 | cg11417029 | Colorectal cancer (SPA correction) || id:ebi-a-GCST90013866 | Inverse variance weighted (fixed effects) | 97 | 4.24E-112 | 1.259(1.234-1.285) |
| LIPT2 | cg11417029 | Colorectal cancer (SPA correction) || id:ebi-a-GCST90013866 | Weighted mode | 97 | 0.004196926 | 1.274(1.084-1.497) |
| LIPT2 | cg11417029 | Colorectal cancer (SPA correction) || id:ebi-a-GCST90013866 | Weighted median | 97 | 1.39E-28 | 1.26(1.209-1.312) |
| LIPT2 | cg11417029 | Colorectal cancer (SPA correction) || id:ebi-a-GCST90013866 | MR Egger | 97 | 0.198396397 | 1.31(0.87-1.972) |
| MRPL28 | cg12437481 | Colorectal cancer (SPA correction) || id:ebi-a-GCST90013866 | Weighted median | 243 | 1.00E-08 | 1.019(1.013-1.026) |
| MRPL28 | cg12437481 | Colorectal cancer (SPA correction) || id:ebi-a-GCST90013866 | Simple mode | 243 | 0.000802482 | 1.022(1.009-1.034) |
| MRPL28 | cg12437481 | Colorectal cancer (SPA correction) || id:ebi-a-GCST90013866 | MR Egger | 243 | 2.99E-06 | 1.022(1.013-1.03) |
| MRPL28 | cg12437481 | Colorectal cancer (SPA correction) || id:ebi-a-GCST90013866 | Inverse variance weighted (fixed effects) | 243 | 2.37E-11 | 1.016(1.012-1.021) |
| MRPL28 | cg12437481 | Colorectal cancer (SPA correction) || id:ebi-a-GCST90013866 | Weighted mode | 243 | 6.72E-05 | 1.02(1.01-1.03) |
| ACADS | cg13914990 | Colorectal cancer (SPA correction) || id:ebi-a-GCST90013866 | Inverse variance weighted (fixed effects) | 576 | 3.11E-64 | 1.048(1.042-1.054) |
| ACADS | cg13914990 | Colorectal cancer (SPA correction) || id:ebi-a-GCST90013866 | MR Egger | 576 | 0.000106296 | 1.037(1.018-1.056) |
| ACADS | cg13914990 | Colorectal cancer (SPA correction) || id:ebi-a-GCST90013866 | Weighted mode | 576 | 0.000139958 | 1.038(1.018-1.058) |
| ACADS | cg13914990 | Colorectal cancer (SPA correction) || id:ebi-a-GCST90013866 | Simple mode | 576 | 0.001590911 | 1.043(1.016-1.07) |
| ACADS | cg13914990 | Colorectal cancer (SPA correction) || id:ebi-a-GCST90013866 | Weighted median | 576 | 6.56E-17 | 1.038(1.029-1.047) |
| NSUN4 | cg14993813 | Colorectal cancer (SPA correction) || id:ebi-a-GCST90013866 | Simple mode | 231 | 4.11E-06 | 1.206(1.116-1.303) |
| NSUN4 | cg14993813 | Colorectal cancer (SPA correction) || id:ebi-a-GCST90013866 | Weighted mode | 231 | 3.40E-06 | 1.206(1.116-1.302) |
| NSUN4 | cg14993813 | Colorectal cancer (SPA correction) || id:ebi-a-GCST90013866 | Inverse variance weighted (fixed effects) | 231 | 0 | 1.209(1.198-1.221) |
| NSUN4 | cg14993813 | Colorectal cancer (SPA correction) || id:ebi-a-GCST90013866 | MR Egger | 231 | 6.86E-05 | 1.156(1.078-1.24) |
| NSUN4 | cg14993813 | Colorectal cancer (SPA correction) || id:ebi-a-GCST90013866 | Weighted median | 231 | 5.15E-73 | 1.206(1.182-1.231) |
| COX15 | cg17885402 | Colorectal cancer (SPA correction) || id:ebi-a-GCST90013866 | Weighted mode | 269 | 5.49E-05 | 0.947(0.923-0.972) |
| COX15 | cg17885402 | Colorectal cancer (SPA correction) || id:ebi-a-GCST90013866 | Simple mode | 269 | 3.05E-40 | 0.761(0.736-0.787) |
| COX15 | cg17885402 | Colorectal cancer (SPA correction) || id:ebi-a-GCST90013866 | Weighted median | 269 | 1.53E-11 | 0.946(0.931-0.962) |
| COX15 | cg17885402 | Colorectal cancer (SPA correction) || id:ebi-a-GCST90013866 | MR Egger | 269 | 2.54E-05 | 1.082(1.043-1.121) |
| PNKD | cg18259342 | Colorectal cancer (SPA correction) || id:ebi-a-GCST90013866 | Simple mode | 340 | 1.33E-12 | 1.096(1.07-1.123) |
| PNKD | cg18259342 | Colorectal cancer (SPA correction) || id:ebi-a-GCST90013866 | Weighted median | 340 | 1.05E-89 | 1.091(1.081-1.1) |
| PNKD | cg18259342 | Colorectal cancer (SPA correction) || id:ebi-a-GCST90013866 | Weighted mode | 340 | 6.13E-13 | 1.096(1.07-1.123) |
| PNKD | cg18259342 | Colorectal cancer (SPA correction) || id:ebi-a-GCST90013866 | Inverse variance weighted (fixed effects) | 340 | 0 | 1.09(1.086-1.094) |
| PNKD | cg18259342 | Colorectal cancer (SPA correction) || id:ebi-a-GCST90013866 | MR Egger | 340 | 6.64E-39 | 1.105(1.09-1.119) |
| COX10 | cg20170777 | Colorectal cancer (SPA correction) || id:ebi-a-GCST90013866 | Weighted mode | 40 | 0.059266769 | 0.89(0.791-1.001) |
| COX10 | cg20170777 | Colorectal cancer (SPA correction) || id:ebi-a-GCST90013866 | Inverse variance weighted (fixed effects) | 40 | 2.34E-13 | 0.88(0.85-0.91) |
| COX10 | cg20170777 | Colorectal cancer (SPA correction) || id:ebi-a-GCST90013866 | Simple mode | 40 | 0.049755592 | 0.89(0.794-0.996) |
| COX10 | cg20170777 | Colorectal cancer (SPA correction) || id:ebi-a-GCST90013866 | MR Egger | 40 | 0.010924208 | 0.631(0.45-0.884) |
| COX10 | cg20170777 | Colorectal cancer (SPA correction) || id:ebi-a-GCST90013866 | Weighted median | 40 | 1.60E-06 | 0.887(0.845-0.932) |
| BAD | cg23796481 | Colorectal cancer (SPA correction) || id:ebi-a-GCST90013866 | Weighted mode | 54 | 0.001561547 | 1.124(1.05-1.205) |
| BAD | cg23796481 | Colorectal cancer (SPA correction) || id:ebi-a-GCST90013866 | Weighted median | 54 | 6.79E-14 | 1.132(1.096-1.17) |
| BAD | cg23796481 | Colorectal cancer (SPA correction) || id:ebi-a-GCST90013866 | Simple mode | 54 | 0.000134256 | 1.17(1.086-1.261) |
| BAD | cg23796481 | Colorectal cancer (SPA correction) || id:ebi-a-GCST90013866 | Inverse variance weighted (fixed effects) | 54 | 8.00E-102 | 1.152(1.137-1.167) |
| BAD | cg23796481 | Colorectal cancer (SPA correction) || id:ebi-a-GCST90013866 | MR Egger | 54 | 0.454920906 | 1.038(0.943-1.142) |
| ABCD3 | cg24215727 | Colorectal cancer (SPA correction) || id:ebi-a-GCST90013866 | Weighted mode | 78 | 0.67264464 | 0.98(0.893-1.075) |
| ABCD3 | cg24215727 | Colorectal cancer (SPA correction) || id:ebi-a-GCST90013866 | MR Egger | 78 | 0.00199148 | 0.37(0.201-0.68) |
| ABCD3 | cg24215727 | Colorectal cancer (SPA correction) || id:ebi-a-GCST90013866 | Simple mode | 78 | 0.139802873 | 0.934(0.854-1.022) |
| ABCD3 | cg24215727 | Colorectal cancer (SPA correction) || id:ebi-a-GCST90013866 | Inverse variance weighted (fixed effects) | 78 | 0.062891444 | 0.975(0.95-1.001) |
| ABCD3 | cg24215727 | Colorectal cancer (SPA correction) || id:ebi-a-GCST90013866 | Weighted median | 78 | 0.005167714 | 0.967(0.944-0.99) |
| CISD3 | cg27492942 | Colorectal cancer (SPA correction) || id:ebi-a-GCST90013866 | Weighted median | 39 | 2.04E-06 | 0.89(0.848-0.934) |
| CISD3 | cg27492942 | Colorectal cancer (SPA correction) || id:ebi-a-GCST90013866 | Inverse variance weighted (fixed effects) | 39 | 2.10E-12 | 0.901(0.875-0.928) |
| CISD3 | cg27492942 | Colorectal cancer (SPA correction) || id:ebi-a-GCST90013866 | MR Egger | 39 | 0.008756394 | 0.861(0.774-0.957) |
| CISD3 | cg27492942 | Colorectal cancer (SPA correction) || id:ebi-a-GCST90013866 | Simple mode | 39 | 0.012571216 | 0.888(0.812-0.971) |
| CISD3 | cg27492942 | Colorectal cancer (SPA correction) || id:ebi-a-GCST90013866 | Weighted mode | 39 | 0.0061006 | 0.885(0.815-0.961) |
| SLC25A30 | cg00139037 | Colorectal cancer || id:ebi-a-GCST90018588 | MR Egger | 84 | 0.269344843 | 0.873(0.687-1.109) |
| SLC25A30 | cg00139037 | Colorectal cancer || id:ebi-a-GCST90018588 | Weighted median | 84 | 7.73E-16 | 1.107(1.08-1.135) |
| SLC25A30 | cg00139037 | Colorectal cancer || id:ebi-a-GCST90018588 | Inverse variance weighted (fixed effects) | 84 | 4.81E-20 | 1.12(1.093-1.147) |
| SLC25A30 | cg00139037 | Colorectal cancer || id:ebi-a-GCST90018588 | Simple mode | 84 | 0.046885682 | 1.161(1.004-1.343) |
| SLC25A30 | cg00139037 | Colorectal cancer || id:ebi-a-GCST90018588 | Weighted mode | 84 | 0.129626231 | 1.099(0.974-1.239) |
| MRPL32 | cg00365680 | Colorectal cancer || id:ebi-a-GCST90018588 | Weighted median | 150 | 1.13E-35 | 0.918(0.906-0.931) |
| MRPL32 | cg00365680 | Colorectal cancer || id:ebi-a-GCST90018588 | Inverse variance weighted (fixed effects) | 150 | 5.40E-68 | 0.921(0.913-0.93) |
| MRPL32 | cg00365680 | Colorectal cancer || id:ebi-a-GCST90018588 | MR Egger | 150 | 2.17E-09 | 0.913(0.887-0.939) |
| MRPL32 | cg00365680 | Colorectal cancer || id:ebi-a-GCST90018588 | Weighted mode | 150 | 1.63E-15 | 0.919(0.902-0.936) |
| MRPL32 | cg00365680 | Colorectal cancer || id:ebi-a-GCST90018588 | Simple mode | 150 | 1.12E-13 | 0.919(0.901-0.938) |
| ACSF3 | cg02193283 | Colorectal cancer || id:ebi-a-GCST90018588 | Inverse variance weighted (fixed effects) | 239 | 0.005510439 | 0.99(0.983-0.997) |
| ACSF3 | cg02193283 | Colorectal cancer || id:ebi-a-GCST90018588 | Simple mode | 239 | 0.003599209 | 0.965(0.943-0.988) |
| ACSF3 | cg02193283 | Colorectal cancer || id:ebi-a-GCST90018588 | Weighted median | 239 | 0.340133722 | 0.995(0.986-1.005) |
| ACSF3 | cg02193283 | Colorectal cancer || id:ebi-a-GCST90018588 | Weighted mode | 239 | 0.952728146 | 0.999(0.979-1.02) |
| ACSF3 | cg02193283 | Colorectal cancer || id:ebi-a-GCST90018588 | MR Egger | 239 | 0.566660224 | 1.012(0.973-1.052) |
| ME3 | cg02493602 | Colorectal cancer || id:ebi-a-GCST90018588 | MR Egger | 216 | 0.292283084 | 1.009(0.992-1.026) |
| ME3 | cg02493602 | Colorectal cancer || id:ebi-a-GCST90018588 | Weighted median | 216 | 5.16E-33 | 1.052(1.043-1.06) |
| ME3 | cg02493602 | Colorectal cancer || id:ebi-a-GCST90018588 | Simple mode | 216 | 1.33E-41 | 1.121(1.106-1.136) |
| ME3 | cg02493602 | Colorectal cancer || id:ebi-a-GCST90018588 | Weighted mode | 216 | 6.86E-20 | 1.045(1.036-1.055) |
| SCP2 | cg03579872 | Colorectal cancer || id:ebi-a-GCST90018588 | MR Egger | 341 | 0.000555923 | 0.934(0.899-0.971) |
| SCP2 | cg03579872 | Colorectal cancer || id:ebi-a-GCST90018588 | Weighted median | 341 | 0.70654307 | 0.998(0.987-1.009) |
| SCP2 | cg03579872 | Colorectal cancer || id:ebi-a-GCST90018588 | Inverse variance weighted (fixed effects) | 341 | 0.00312372 | 1.012(1.004-1.02) |
| SCP2 | cg03579872 | Colorectal cancer || id:ebi-a-GCST90018588 | Simple mode | 341 | 1.93E-40 | 1.146(1.126-1.167) |
| SCP2 | cg03579872 | Colorectal cancer || id:ebi-a-GCST90018588 | Weighted mode | 341 | 0.48304661 | 0.996(0.983-1.008) |
| ACSF3 | cg04308346 | Colorectal cancer || id:ebi-a-GCST90018588 | Weighted median | 9 | 0.370221703 | 1.024(0.972-1.08) |
| ACSF3 | cg04308346 | Colorectal cancer || id:ebi-a-GCST90018588 | Inverse variance weighted (fixed effects) | 9 | 0.517593238 | 1.017(0.966-1.072) |
| ACSF3 | cg04308346 | Colorectal cancer || id:ebi-a-GCST90018588 | Simple mode | 9 | 0.641314489 | 1.026(0.925-1.137) |
| ACSF3 | cg04308346 | Colorectal cancer || id:ebi-a-GCST90018588 | Weighted mode | 9 | 0.631817793 | 1.026(0.929-1.133) |
| ACSF3 | cg04308346 | Colorectal cancer || id:ebi-a-GCST90018588 | MR Egger | 9 | 0.751682657 | 0.748(0.133-4.216) |
| MSRB2 | cg07355157 | Colorectal cancer || id:ebi-a-GCST90018588 | MR Egger | 153 | 2.88E-13 | 1.06(1.045-1.075) |
| MSRB2 | cg07355157 | Colorectal cancer || id:ebi-a-GCST90018588 | Inverse variance weighted (fixed effects) | 153 | 1.80E-09 | 1.022(1.015-1.029) |
| MSRB2 | cg07355157 | Colorectal cancer || id:ebi-a-GCST90018588 | Weighted mode | 153 | 2.37E-06 | 1.031(1.019-1.044) |
| MSRB2 | cg07355157 | Colorectal cancer || id:ebi-a-GCST90018588 | Weighted median | 153 | 8.06E-08 | 1.029(1.018-1.04) |
| MSRB2 | cg07355157 | Colorectal cancer || id:ebi-a-GCST90018588 | Simple mode | 153 | 0.00034478 | 1.032(1.015-1.05) |
| CYB5R3 | cg08690876 | Colorectal cancer || id:ebi-a-GCST90018588 | Simple mode | 297 | 0.003017309 | 1.06(1.02-1.102) |
| CYB5R3 | cg08690876 | Colorectal cancer || id:ebi-a-GCST90018588 | Weighted mode | 297 | 0.000245594 | 1.06(1.028-1.094) |
| CYB5R3 | cg08690876 | Colorectal cancer || id:ebi-a-GCST90018588 | Weighted median | 297 | 1.18E-19 | 1.05(1.039-1.061) |
| CYB5R3 | cg08690876 | Colorectal cancer || id:ebi-a-GCST90018588 | MR Egger | 297 | 2.94E-08 | 1.063(1.041-1.085) |
| VARS2 | cg10661769 | Colorectal cancer || id:ebi-a-GCST90018588 | Inverse variance weighted (fixed effects) | 2 | 0.793899047 | 1.024(0.856-1.226) |
| LIPT2 | cg11417029 | Colorectal cancer || id:ebi-a-GCST90018588 | Simple mode | 113 | 0.028328914 | 1.216(1.023-1.445) |
| LIPT2 | cg11417029 | Colorectal cancer || id:ebi-a-GCST90018588 | Inverse variance weighted (fixed effects) | 113 | 1.56E-134 | 1.205(1.187-1.223) |
| LIPT2 | cg11417029 | Colorectal cancer || id:ebi-a-GCST90018588 | Weighted mode | 113 | 0.068233223 | 1.162(0.99-1.364) |
| LIPT2 | cg11417029 | Colorectal cancer || id:ebi-a-GCST90018588 | MR Egger | 113 | 0.06551557 | 0.759(0.567-1.015) |
| LIPT2 | cg11417029 | Colorectal cancer || id:ebi-a-GCST90018588 | Weighted median | 113 | 6.36E-55 | 1.212(1.183-1.242) |
| MRPL28 | cg12437481 | Colorectal cancer || id:ebi-a-GCST90018588 | MR Egger | 209 | 0.442705461 | 0.994(0.978-1.01) |
| MRPL28 | cg12437481 | Colorectal cancer || id:ebi-a-GCST90018588 | Weighted median | 209 | 0.125200152 | 0.993(0.984-1.002) |
| MRPL28 | cg12437481 | Colorectal cancer || id:ebi-a-GCST90018588 | Weighted mode | 209 | 0.004607409 | 0.973(0.955-0.991) |
| MRPL28 | cg12437481 | Colorectal cancer || id:ebi-a-GCST90018588 | Simple mode | 209 | 0.635931176 | 1.005(0.984-1.027) |
| ACADS | cg13914990 | Colorectal cancer || id:ebi-a-GCST90018588 | Weighted mode | 531 | 0.910383636 | 1.001(0.979-1.024) |
| ACADS | cg13914990 | Colorectal cancer || id:ebi-a-GCST90018588 | Simple mode | 531 | 0.914158949 | 1.001(0.978-1.025) |
| ACADS | cg13914990 | Colorectal cancer || id:ebi-a-GCST90018588 | Inverse variance weighted (fixed effects) | 531 | 0.007103239 | 1.006(1.002-1.01) |
| ACADS | cg13914990 | Colorectal cancer || id:ebi-a-GCST90018588 | MR Egger | 531 | 0.009599782 | 0.981(0.966-0.995) |
| ACADS | cg13914990 | Colorectal cancer || id:ebi-a-GCST90018588 | Weighted median | 531 | 0.158439656 | 1.004(0.998-1.011) |
| NSUN4 | cg14993813 | Colorectal cancer || id:ebi-a-GCST90018588 | Simple mode | 245 | 0.004239025 | 0.626(0.456-0.86) |
| NSUN4 | cg14993813 | Colorectal cancer || id:ebi-a-GCST90018588 | MR Egger | 245 | 1.58E-58 | 0.363(0.331-0.398) |
| NSUN4 | cg14993813 | Colorectal cancer || id:ebi-a-GCST90018588 | Weighted mode | 245 | 2.95E-28 | 0.626(0.582-0.673) |
| NSUN4 | cg14993813 | Colorectal cancer || id:ebi-a-GCST90018588 | Weighted mode | 245 | 1.37E-25 | 0.626(0.579-0.677) |
| NSUN4 | cg14993813 | Colorectal cancer || id:ebi-a-GCST90018588 | Inverse variance weighted (fixed effects) | 245 | 4.03E-125 | 0.751(0.734-0.769) |
| NSUN4 | cg14993813 | Colorectal cancer || id:ebi-a-GCST90018588 | Weighted median | 245 | 0.004912852 | 0.905(0.843-0.97) |
| NSUN4 | cg14993813 | Colorectal cancer || id:ebi-a-GCST90018588 | Simple mode | 245 | 0.001961185 | 0.626(0.467-0.839) |
| NSUN4 | cg14993813 | Colorectal cancer || id:ebi-a-GCST90018588 | Weighted median | 245 | 0.003715186 | 0.905(0.845-0.968) |
| COX15 | cg17885402 | Colorectal cancer || id:ebi-a-GCST90018588 | Weighted mode | 298 | 2.46E-10 | 0.92(0.897-0.943) |
| COX15 | cg17885402 | Colorectal cancer || id:ebi-a-GCST90018588 | MR Egger | 298 | 0.217556271 | 1.015(0.991-1.039) |
| COX15 | cg17885402 | Colorectal cancer || id:ebi-a-GCST90018588 | Inverse variance weighted (fixed effects) | 298 | 1.26E-60 | 0.948(0.942-0.954) |
| COX15 | cg17885402 | Colorectal cancer || id:ebi-a-GCST90018588 | Simple mode | 298 | 5.97E-08 | 0.918(0.891-0.946) |
| COX15 | cg17885402 | Colorectal cancer || id:ebi-a-GCST90018588 | Weighted median | 298 | 1.52E-17 | 0.959(0.95-0.969) |
| PNKD | cg18259342 | Colorectal cancer || id:ebi-a-GCST90018588 | Weighted mode | 368 | 5.29E-09 | 1.036(1.024-1.048) |
| PNKD | cg18259342 | Colorectal cancer || id:ebi-a-GCST90018588 | Simple mode | 368 | 6.66E-08 | 1.037(1.023-1.05) |
| PNKD | cg18259342 | Colorectal cancer || id:ebi-a-GCST90018588 | Weighted median | 368 | 4.97E-22 | 1.021(1.017-1.026) |
| PNKD | cg18259342 | Colorectal cancer || id:ebi-a-GCST90018588 | Inverse variance weighted (fixed effects) | 368 | 5.28E-63 | 1.025(1.022-1.028) |
| PNKD | cg18259342 | Colorectal cancer || id:ebi-a-GCST90018588 | MR Egger | 368 | 2.22E-18 | 1.048(1.038-1.058) |
| COX10 | cg20170777 | Colorectal cancer || id:ebi-a-GCST90018588 | Weighted mode | 47 | 0.022568066 | 0.881(0.793-0.979) |
| COX10 | cg20170777 | Colorectal cancer || id:ebi-a-GCST90018588 | Simple mode | 47 | 0.056011431 | 0.88(0.775-1) |
| COX10 | cg20170777 | Colorectal cancer || id:ebi-a-GCST90018588 | Inverse variance weighted (fixed effects) | 47 | 7.36E-09 | 0.906(0.877-0.937) |
| COX10 | cg20170777 | Colorectal cancer || id:ebi-a-GCST90018588 | MR Egger | 47 | 0.088238213 | 0.747(0.539-1.037) |
| COX10 | cg20170777 | Colorectal cancer || id:ebi-a-GCST90018588 | Weighted median | 47 | 8.22E-06 | 0.883(0.836-0.933) |
| BAD | cg23796481 | Colorectal cancer || id:ebi-a-GCST90018588 | Weighted mode | 60 | 0.01278186 | 0.955(0.923-0.989) |
| BAD | cg23796481 | Colorectal cancer || id:ebi-a-GCST90018588 | Inverse variance weighted (fixed effects) | 60 | 3.45E-16 | 0.956(0.946-0.967) |
| BAD | cg23796481 | Colorectal cancer || id:ebi-a-GCST90018588 | Simple mode | 60 | 0.017137581 | 0.955(0.921-0.991) |
| BAD | cg23796481 | Colorectal cancer || id:ebi-a-GCST90018588 | MR Egger | 60 | 0.212090145 | 0.956(0.891-1.025) |
| BAD | cg23796481 | Colorectal cancer || id:ebi-a-GCST90018588 | Weighted median | 60 | 2.46E-09 | 0.955(0.941-0.97) |
| ABCD3 | cg24215727 | Colorectal cancer || id:ebi-a-GCST90018588 | Simple mode | 92 | 0.864502959 | 1.004(0.955-1.057) |
| ABCD3 | cg24215727 | Colorectal cancer || id:ebi-a-GCST90018588 | Inverse variance weighted (fixed effects) | 92 | 0.078193256 | 1.016(0.998-1.035) |
| ABCD3 | cg24215727 | Colorectal cancer || id:ebi-a-GCST90018588 | Weighted mode | 92 | 0.877187237 | 1.004(0.95-1.063) |
| ABCD3 | cg24215727 | Colorectal cancer || id:ebi-a-GCST90018588 | MR Egger | 92 | 0.280200766 | 0.786(0.509-1.214) |
| ABCD3 | cg24215727 | Colorectal cancer || id:ebi-a-GCST90018588 | Weighted median | 92 | 0.517509734 | 1.005(0.989-1.022) |
| CISD3 | cg27492942 | Colorectal cancer || id:ebi-a-GCST90018588 | Simple mode | 35 | 0.026284927 | 0.878(0.786-0.98) |
| CISD3 | cg27492942 | Colorectal cancer || id:ebi-a-GCST90018588 | Inverse variance weighted (fixed effects) | 35 | 6.24E-22 | 0.87(0.845-0.895) |
| CISD3 | cg27492942 | Colorectal cancer || id:ebi-a-GCST90018588 | Weighted mode | 35 | 0.041269843 | 0.91(0.835-0.993) |
| CISD3 | cg27492942 | Colorectal cancer || id:ebi-a-GCST90018588 | MR Egger | 35 | 1.03E-05 | 0.763(0.689-0.845) |
| CISD3 | cg27492942 | Colorectal cancer || id:ebi-a-GCST90018588 | Weighted median | 35 | 0.00019535 | 0.892(0.84-0.947) |
| SLC25A30 | cg00139037 | Colorectal cancer || id:ebi-a-GCST90018808 | Weighted mode | 82 | 0.15684432 | 1.145(0.951-1.379) |
| SLC25A30 | cg00139037 | Colorectal cancer || id:ebi-a-GCST90018808 | Simple mode | 82 | 0.150790157 | 1.146(0.953-1.379) |
| SLC25A30 | cg00139037 | Colorectal cancer || id:ebi-a-GCST90018808 | MR Egger | 82 | 0.649998712 | 1.035(0.891-1.203) |
| SLC25A30 | cg00139037 | Colorectal cancer || id:ebi-a-GCST90018808 | Inverse variance weighted (fixed effects) | 82 | 1.12E-72 | 1.157(1.139-1.175) |
| SLC25A30 | cg00139037 | Colorectal cancer || id:ebi-a-GCST90018808 | Weighted median | 82 | 6.85E-38 | 1.148(1.124-1.172) |
| MRPL32 | cg00365680 | Colorectal cancer || id:ebi-a-GCST90018808 | Weighted mode | 200 | 0.00092653 | 0.977(0.964-0.99) |
| MRPL32 | cg00365680 | Colorectal cancer || id:ebi-a-GCST90018808 | Simple mode | 200 | 0.010523992 | 0.977(0.96-0.994) |
| MRPL32 | cg00365680 | Colorectal cancer || id:ebi-a-GCST90018808 | MR Egger | 200 | 0.401510599 | 0.993(0.978-1.009) |
| MRPL32 | cg00365680 | Colorectal cancer || id:ebi-a-GCST90018808 | Weighted median | 200 | 2.69E-09 | 0.978(0.971-0.985) |
| MRPL32 | cg00365680 | Colorectal cancer || id:ebi-a-GCST90018808 | Inverse variance weighted (fixed effects) | 200 | 4.33E-08 | 0.985(0.98-0.99) |
| ACSF3 | cg02193283 | Colorectal cancer || id:ebi-a-GCST90018808 | MR Egger | 346 | 0.001090059 | 0.956(0.931-0.982) |
| ACSF3 | cg02193283 | Colorectal cancer || id:ebi-a-GCST90018808 | Inverse variance weighted (fixed effects) | 346 | 4.75E-82 | 0.952(0.947-0.957) |
| ACSF3 | cg02193283 | Colorectal cancer || id:ebi-a-GCST90018808 | Simple mode | 346 | 2.37E-07 | 0.934(0.911-0.958) |
| ACSF3 | cg02193283 | Colorectal cancer || id:ebi-a-GCST90018808 | Weighted mode | 346 | 6.61E-09 | 0.935(0.915-0.956) |
| ACSF3 | cg02193283 | Colorectal cancer || id:ebi-a-GCST90018808 | Weighted median | 346 | 3.96E-35 | 0.952(0.944-0.959) |
| ME3 | cg02493602 | Colorectal cancer || id:ebi-a-GCST90018808 | Inverse variance weighted (fixed effects) | 222 | 1.25E-108 | 1.046(1.042-1.05) |
| ME3 | cg02493602 | Colorectal cancer || id:ebi-a-GCST90018808 | Simple mode | 222 | 3.61E-09 | 1.038(1.026-1.051) |
| ME3 | cg02493602 | Colorectal cancer || id:ebi-a-GCST90018808 | Weighted median | 222 | 5.42E-38 | 1.043(1.037-1.05) |
| ME3 | cg02493602 | Colorectal cancer || id:ebi-a-GCST90018808 | Weighted mode | 222 | 2.35E-21 | 1.045(1.036-1.054) |
| ME3 | cg02493602 | Colorectal cancer || id:ebi-a-GCST90018808 | MR Egger | 222 | 4.36E-11 | 1.038(1.027-1.049) |
| SCP2 | cg03579872 | Colorectal cancer || id:ebi-a-GCST90018808 | Inverse variance weighted (fixed effects) | 65 | 0.075742262 | 1.014(0.999-1.03) |
| SCP2 | cg03579872 | Colorectal cancer || id:ebi-a-GCST90018808 | Simple mode | 65 | 0.609456109 | 1.012(0.966-1.06) |
| SCP2 | cg03579872 | Colorectal cancer || id:ebi-a-GCST90018808 | Weighted mode | 65 | 0.566252484 | 1.013(0.97-1.058) |
| SCP2 | cg03579872 | Colorectal cancer || id:ebi-a-GCST90018808 | Weighted median | 65 | 0.186463051 | 1.012(0.994-1.031) |
| SCP2 | cg03579872 | Colorectal cancer || id:ebi-a-GCST90018808 | MR Egger | 65 | 0.011888602 | 1.132(1.031-1.243) |
| ACSF3 | cg04308346 | Colorectal cancer || id:ebi-a-GCST90018808 | MR Egger | 9 | 0.870600123 | 1.117(0.308-4.054) |
| ACSF3 | cg04308346 | Colorectal cancer || id:ebi-a-GCST90018808 | Weighted median | 9 | 0.119258383 | 1.031(0.992-1.071) |
| ACSF3 | cg04308346 | Colorectal cancer || id:ebi-a-GCST90018808 | Inverse variance weighted (fixed effects) | 9 | 0.14482475 | 1.029(0.99-1.07) |
| ACSF3 | cg04308346 | Colorectal cancer || id:ebi-a-GCST90018808 | Simple mode | 9 | 0.504625049 | 1.032(0.945-1.127) |
| ACSF3 | cg04308346 | Colorectal cancer || id:ebi-a-GCST90018808 | Weighted mode | 9 | 0.526056185 | 1.032(0.941-1.131) |
| MSRB2 | cg07355157 | Colorectal cancer || id:ebi-a-GCST90018808 | Simple mode | 163 | 5.39E-09 | 1.05(1.034-1.066) |
| MSRB2 | cg07355157 | Colorectal cancer || id:ebi-a-GCST90018808 | Weighted median | 163 | 2.88E-22 | 1.049(1.039-1.059) |
| MSRB2 | cg07355157 | Colorectal cancer || id:ebi-a-GCST90018808 | Inverse variance weighted (fixed effects) | 163 | 1.62E-97 | 1.054(1.049-1.059) |
| MSRB2 | cg07355157 | Colorectal cancer || id:ebi-a-GCST90018808 | Weighted mode | 163 | 3.57E-14 | 1.047(1.036-1.059) |
| MSRB2 | cg07355157 | Colorectal cancer || id:ebi-a-GCST90018808 | MR Egger | 163 | 4.15E-20 | 1.056(1.045-1.067) |
| CYB5R3 | cg08690876 | Colorectal cancer || id:ebi-a-GCST90018808 | Inverse variance weighted (fixed effects) | 366 | 5.92E-66 | 1.035(1.031-1.039) |
| CYB5R3 | cg08690876 | Colorectal cancer || id:ebi-a-GCST90018808 | Weighted median | 366 | 9.47E-23 | 1.032(1.026-1.039) |
| CYB5R3 | cg08690876 | Colorectal cancer || id:ebi-a-GCST90018808 | MR Egger | 366 | 3.15E-13 | 1.043(1.032-1.054) |
| CYB5R3 | cg08690876 | Colorectal cancer || id:ebi-a-GCST90018808 | Simple mode | 366 | 3.41E-06 | 1.048(1.028-1.068) |
| CYB5R3 | cg08690876 | Colorectal cancer || id:ebi-a-GCST90018808 | Weighted mode | 366 | 7.61E-07 | 1.042(1.026-1.059) |
| ECHDC2 | cg09128567 | Colorectal cancer || id:ebi-a-GCST90018808 | Inverse variance weighted (fixed effects) | 268 | 2.52E-106 | 1.085(1.077-1.093) |
| ECHDC2 | cg09128567 | Colorectal cancer || id:ebi-a-GCST90018808 | Simple mode | 268 | 1.23E-05 | 1.114(1.062-1.169) |
| ECHDC2 | cg09128567 | Colorectal cancer || id:ebi-a-GCST90018808 | Weighted mode | 268 | 0.000442594 | 1.064(1.028-1.101) |
| ECHDC2 | cg09128567 | Colorectal cancer || id:ebi-a-GCST90018808 | MR Egger | 268 | 4.16E-14 | 1.217(1.16-1.278) |
| ECHDC2 | cg09128567 | Colorectal cancer || id:ebi-a-GCST90018808 | Weighted median | 268 | 2.09E-21 | 1.061(1.048-1.075) |
| VARS2 | cg10661769 | Colorectal cancer || id:ebi-a-GCST90018808 | Inverse variance weighted (fixed effects) | 2 | 0.603917161 | 1.028(0.925-1.143) |
| LIPT2 | cg11417029 | Colorectal cancer || id:ebi-a-GCST90018808 | MR Egger | 114 | 0.346150529 | 0.903(0.73-1.116) |
| LIPT2 | cg11417029 | Colorectal cancer || id:ebi-a-GCST90018808 | Inverse variance weighted (fixed effects) | 114 | 0 | 1.295(1.282-1.309) |
| LIPT2 | cg11417029 | Colorectal cancer || id:ebi-a-GCST90018808 | Weighted median | 114 | 7.41E-62 | 1.298(1.258-1.338) |
| LIPT2 | cg11417029 | Colorectal cancer || id:ebi-a-GCST90018808 | Simple mode | 114 | 0.01864254 | 1.318(1.051-1.654) |
| LIPT2 | cg11417029 | Colorectal cancer || id:ebi-a-GCST90018808 | Weighted mode | 114 | 0.009535179 | 1.318(1.074-1.619) |
| MRPL28 | cg12437481 | Colorectal cancer || id:ebi-a-GCST90018808 | MR Egger | 272 | 1.63E-10 | 1.03(1.021-1.038) |
| MRPL28 | cg12437481 | Colorectal cancer || id:ebi-a-GCST90018808 | Weighted mode | 272 | 0.664247667 | 0.996(0.98-1.013) |
| MRPL28 | cg12437481 | Colorectal cancer || id:ebi-a-GCST90018808 | Simple mode | 272 | 0.726615161 | 0.996(0.976-1.017) |
| MRPL28 | cg12437481 | Colorectal cancer || id:ebi-a-GCST90018808 | Weighted median | 272 | 4.45E-09 | 1.018(1.012-1.024) |
| ACADS | cg13914990 | Colorectal cancer || id:ebi-a-GCST90018808 | Weighted mode | 638 | 0.000809338 | 1.018(1.007-1.028) |
| ACADS | cg13914990 | Colorectal cancer || id:ebi-a-GCST90018808 | MR Egger | 638 | 0.000221828 | 1.02(1.009-1.03) |
| ACADS | cg13914990 | Colorectal cancer || id:ebi-a-GCST90018808 | Weighted median | 638 | 3.15E-16 | 1.018(1.014-1.022) |
| ACADS | cg13914990 | Colorectal cancer || id:ebi-a-GCST90018808 | Simple mode | 638 | 0.001650545 | 1.021(1.008-1.034) |
| ACADS | cg13914990 | Colorectal cancer || id:ebi-a-GCST90018808 | Inverse variance weighted (fixed effects) | 638 | 8.84E-35 | 1.02(1.016-1.023) |
| NSUN4 | cg14993813 | Colorectal cancer || id:ebi-a-GCST90018808 | MR Egger | 256 | 1.26E-06 | 1.112(1.066-1.16) |
| NSUN4 | cg14993813 | Colorectal cancer || id:ebi-a-GCST90018808 | Weighted median | 256 | 5.96E-39 | 1.074(1.063-1.086) |
| NSUN4 | cg14993813 | Colorectal cancer || id:ebi-a-GCST90018808 | Weighted mode | 256 | 0.000140028 | 1.074(1.036-1.114) |
| NSUN4 | cg14993813 | Colorectal cancer || id:ebi-a-GCST90018808 | Inverse variance weighted (fixed effects) | 256 | 1.04E-107 | 1.082(1.074-1.089) |
| NSUN4 | cg14993813 | Colorectal cancer || id:ebi-a-GCST90018808 | Simple mode | 256 | 0.000703266 | 1.074(1.031-1.119) |
| COX15 | cg17885402 | Colorectal cancer || id:ebi-a-GCST90018808 | MR Egger | 306 | 0.087030943 | 1.017(0.998-1.038) |
| COX15 | cg17885402 | Colorectal cancer || id:ebi-a-GCST90018808 | Weighted median | 306 | 3.46E-16 | 0.96(0.95-0.969) |
| COX15 | cg17885402 | Colorectal cancer || id:ebi-a-GCST90018808 | Simple mode | 306 | 1.67E-16 | 0.871(0.844-0.898) |
| COX15 | cg17885402 | Colorectal cancer || id:ebi-a-GCST90018808 | Weighted mode | 306 | 0.035400802 | 0.98(0.962-0.999) |
| PNKD | cg18259342 | Colorectal cancer || id:ebi-a-GCST90018808 | MR Egger | 369 | 3.37E-39 | 1.057(1.049-1.065) |
| PNKD | cg18259342 | Colorectal cancer || id:ebi-a-GCST90018808 | Weighted mode | 369 | 3.09E-13 | 1.049(1.036-1.062) |
| PNKD | cg18259342 | Colorectal cancer || id:ebi-a-GCST90018808 | Weighted median | 369 | 3.98E-95 | 1.046(1.042-1.05) |
| PNKD | cg18259342 | Colorectal cancer || id:ebi-a-GCST90018808 | Inverse variance weighted (fixed effects) | 369 | 0 | 1.047(1.045-1.049) |
| PNKD | cg18259342 | Colorectal cancer || id:ebi-a-GCST90018808 | Simple mode | 369 | 1.42E-10 | 1.05(1.035-1.066) |
| COX10 | cg20170777 | Colorectal cancer || id:ebi-a-GCST90018808 | Weighted mode | 47 | 0.003253991 | 0.882(0.815-0.955) |
| COX10 | cg20170777 | Colorectal cancer || id:ebi-a-GCST90018808 | Simple mode | 47 | 0.007276622 | 0.88(0.805-0.962) |
| COX10 | cg20170777 | Colorectal cancer || id:ebi-a-GCST90018808 | Inverse variance weighted (fixed effects) | 47 | 3.00E-29 | 0.89(0.873-0.909) |
| COX10 | cg20170777 | Colorectal cancer || id:ebi-a-GCST90018808 | MR Egger | 47 | 0.031799426 | 0.798(0.653-0.974) |
| COX10 | cg20170777 | Colorectal cancer || id:ebi-a-GCST90018808 | Weighted median | 47 | 1.10E-10 | 0.885(0.852-0.918) |
| BAD | cg23796481 | Colorectal cancer || id:ebi-a-GCST90018808 | Weighted mode | 60 | 0.531609172 | 1.006(0.987-1.025) |
| BAD | cg23796481 | Colorectal cancer || id:ebi-a-GCST90018808 | MR Egger | 60 | 0.321159544 | 0.976(0.931-1.023) |
| BAD | cg23796481 | Colorectal cancer || id:ebi-a-GCST90018808 | Inverse variance weighted (fixed effects) | 60 | 0.189803635 | 1.005(0.998-1.013) |
| BAD | cg23796481 | Colorectal cancer || id:ebi-a-GCST90018808 | Simple mode | 60 | 0.548667154 | 1.006(0.986-1.026) |
| BAD | cg23796481 | Colorectal cancer || id:ebi-a-GCST90018808 | Weighted median | 60 | 0.256350121 | 1.005(0.996-1.015) |
| ABCD3 | cg24215727 | Colorectal cancer || id:ebi-a-GCST90018808 | Simple mode | 88 | 0.106320952 | 0.954(0.901-1.01) |
| ABCD3 | cg24215727 | Colorectal cancer || id:ebi-a-GCST90018808 | Weighted median | 88 | 3.44E-07 | 0.966(0.953-0.979) |
| ABCD3 | cg24215727 | Colorectal cancer || id:ebi-a-GCST90018808 | Weighted mode | 88 | 0.076282835 | 0.954(0.907-1.004) |
| ABCD3 | cg24215727 | Colorectal cancer || id:ebi-a-GCST90018808 | Inverse variance weighted (fixed effects) | 88 | 5.07E-07 | 0.965(0.952-0.979) |
| ABCD3 | cg24215727 | Colorectal cancer || id:ebi-a-GCST90018808 | MR Egger | 88 | 0.021262419 | 0.678(0.49-0.938) |
| CISD3 | cg27492942 | Colorectal cancer || id:ebi-a-GCST90018808 | Weighted mode | 44 | 0.002285962 | 0.892(0.832-0.956) |
| CISD3 | cg27492942 | Colorectal cancer || id:ebi-a-GCST90018808 | Simple mode | 44 | 0.002184977 | 0.882(0.818-0.951) |
| CISD3 | cg27492942 | Colorectal cancer || id:ebi-a-GCST90018808 | Inverse variance weighted (fixed effects) | 44 | 3.00E-31 | 0.899(0.883-0.916) |
| CISD3 | cg27492942 | Colorectal cancer || id:ebi-a-GCST90018808 | Weighted median | 44 | 5.10E-09 | 0.9(0.869-0.932) |
| CISD3 | cg27492942 | Colorectal cancer || id:ebi-a-GCST90018808 | MR Egger | 44 | 4.25E-07 | 0.823(0.772-0.877) |
| SLC25A30 | cg00139037 | Colorectal cancer || id:finn-b-C3_COLORECTAL | Simple mode | 74 | 0.286824232 | 1.075(0.942-1.226) |
| SLC25A30 | cg00139037 | Colorectal cancer || id:finn-b-C3_COLORECTAL | Weighted mode | 74 | 0.274723903 | 1.075(0.945-1.224) |
| SLC25A30 | cg00139037 | Colorectal cancer || id:finn-b-C3_COLORECTAL | MR Egger | 74 | 0.549350278 | 1.094(0.817-1.465) |
| SLC25A30 | cg00139037 | Colorectal cancer || id:finn-b-C3_COLORECTAL | Weighted median | 74 | 2.37E-09 | 1.075(1.05-1.101) |
| SLC25A30 | cg00139037 | Colorectal cancer || id:finn-b-C3_COLORECTAL | Inverse variance weighted (fixed effects) | 74 | 5.80E-07 | 1.082(1.049-1.115) |
| MRPL32 | cg00365680 | Colorectal cancer || id:finn-b-C3_COLORECTAL | Weighted mode | 199 | 4.29E-06 | 1.099(1.057-1.142) |
| MRPL32 | cg00365680 | Colorectal cancer || id:finn-b-C3_COLORECTAL | Inverse variance weighted (fixed effects) | 199 | 4.74E-47 | 1.102(1.087-1.116) |
| MRPL32 | cg00365680 | Colorectal cancer || id:finn-b-C3_COLORECTAL | Simple mode | 199 | 2.59E-05 | 1.104(1.055-1.155) |
| MRPL32 | cg00365680 | Colorectal cancer || id:finn-b-C3_COLORECTAL | Weighted median | 199 | 1.72E-28 | 1.111(1.091-1.132) |
| MRPL32 | cg00365680 | Colorectal cancer || id:finn-b-C3_COLORECTAL | MR Egger | 199 | 2.66E-24 | 1.227(1.186-1.27) |
| ACSF3 | cg02193283 | Colorectal cancer || id:finn-b-C3_COLORECTAL | Weighted median | 338 | 0.009383107 | 1.023(1.006-1.04) |
| ACSF3 | cg02193283 | Colorectal cancer || id:finn-b-C3_COLORECTAL | MR Egger | 338 | 7.62E-06 | 1.155(1.085-1.229) |
| ACSF3 | cg02193283 | Colorectal cancer || id:finn-b-C3_COLORECTAL | Simple mode | 338 | 0.927911988 | 1.002(0.963-1.043) |
| ACSF3 | cg02193283 | Colorectal cancer || id:finn-b-C3_COLORECTAL | Weighted mode | 338 | 0.859570556 | 0.997(0.965-1.03) |
| ME3 | cg02493602 | Colorectal cancer || id:finn-b-C3_COLORECTAL | MR Egger | 228 | 0.029022584 | 0.969(0.942-0.997) |
| ME3 | cg02493602 | Colorectal cancer || id:finn-b-C3_COLORECTAL | Weighted median | 228 | 0.608692659 | 0.996(0.983-1.01) |
| ME3 | cg02493602 | Colorectal cancer || id:finn-b-C3_COLORECTAL | Inverse variance weighted (fixed effects) | 228 | 0.000125245 | 0.98(0.97-0.99) |
| ME3 | cg02493602 | Colorectal cancer || id:finn-b-C3_COLORECTAL | Simple mode | 228 | 0.994095875 | 1(0.969-1.032) |
| ME3 | cg02493602 | Colorectal cancer || id:finn-b-C3_COLORECTAL | Weighted mode | 228 | 0.83577164 | 0.997(0.972-1.023) |
| SCP2 | cg03579872 | Colorectal cancer || id:finn-b-C3_COLORECTAL | Weighted mode | 65 | 0.68123649 | 0.983(0.906-1.067) |
| SCP2 | cg03579872 | Colorectal cancer || id:finn-b-C3_COLORECTAL | Inverse variance weighted (fixed effects) | 65 | 0.138615775 | 0.98(0.954-1.007) |
| SCP2 | cg03579872 | Colorectal cancer || id:finn-b-C3_COLORECTAL | Weighted median | 65 | 0.340030737 | 0.987(0.961-1.014) |
| SCP2 | cg03579872 | Colorectal cancer || id:finn-b-C3_COLORECTAL | Simple mode | 65 | 0.18933844 | 0.948(0.875-1.026) |
| SCP2 | cg03579872 | Colorectal cancer || id:finn-b-C3_COLORECTAL | MR Egger | 65 | 0.002465167 | 1.354(1.122-1.635) |
| ACSF3 | cg04308346 | Colorectal cancer || id:finn-b-C3_COLORECTAL | MR Egger | 10 | 0.851162193 | 1.328(0.076-23.311) |
| ACSF3 | cg04308346 | Colorectal cancer || id:finn-b-C3_COLORECTAL | Weighted mode | 10 | 0.257970535 | 0.887(0.73-1.078) |
| ACSF3 | cg04308346 | Colorectal cancer || id:finn-b-C3_COLORECTAL | Inverse variance weighted (fixed effects) | 10 | 0.003422668 | 0.89(0.823-0.962) |
| ACSF3 | cg04308346 | Colorectal cancer || id:finn-b-C3_COLORECTAL | Simple mode | 10 | 0.383129499 | 0.887(0.686-1.147) |
| ACSF3 | cg04308346 | Colorectal cancer || id:finn-b-C3_COLORECTAL | Weighted median | 10 | 0.157828476 | 0.888(0.754-1.047) |
| MSRB2 | cg07355157 | Colorectal cancer || id:finn-b-C3_COLORECTAL | Inverse variance weighted (fixed effects) | 156 | 0.049786233 | 0.99(0.98-1) |
| MSRB2 | cg07355157 | Colorectal cancer || id:finn-b-C3_COLORECTAL | Weighted median | 156 | 0.000226854 | 0.973(0.958-0.987) |
| MSRB2 | cg07355157 | Colorectal cancer || id:finn-b-C3_COLORECTAL | MR Egger | 156 | 0.906783942 | 0.999(0.979-1.019) |
| MSRB2 | cg07355157 | Colorectal cancer || id:finn-b-C3_COLORECTAL | Weighted mode | 156 | 0.001371957 | 0.971(0.954-0.988) |
| MSRB2 | cg07355157 | Colorectal cancer || id:finn-b-C3_COLORECTAL | Simple mode | 156 | 0.009768889 | 0.963(0.937-0.991) |
| CYB5R3 | cg08690876 | Colorectal cancer || id:finn-b-C3_COLORECTAL | Weighted mode | 348 | 5.72E-09 | 1.079(1.052-1.106) |
| CYB5R3 | cg08690876 | Colorectal cancer || id:finn-b-C3_COLORECTAL | MR Egger | 348 | 2.68E-06 | 1.051(1.03-1.073) |
| CYB5R3 | cg08690876 | Colorectal cancer || id:finn-b-C3_COLORECTAL | Inverse variance weighted (fixed effects) | 348 | 1.01E-84 | 1.078(1.07-1.087) |
| CYB5R3 | cg08690876 | Colorectal cancer || id:finn-b-C3_COLORECTAL | Simple mode | 348 | 6.95E-07 | 1.092(1.055-1.13) |
| CYB5R3 | cg08690876 | Colorectal cancer || id:finn-b-C3_COLORECTAL | Weighted median | 348 | 3.38E-27 | 1.072(1.059-1.086) |
| ECHDC2 | cg09128567 | Colorectal cancer || id:finn-b-C3_COLORECTAL | Weighted median | 247 | 0.552228266 | 0.997(0.986-1.008) |
| ECHDC2 | cg09128567 | Colorectal cancer || id:finn-b-C3_COLORECTAL | Inverse variance weighted (fixed effects) | 247 | 0.165884864 | 1.009(0.996-1.022) |
| ECHDC2 | cg09128567 | Colorectal cancer || id:finn-b-C3_COLORECTAL | Simple mode | 247 | 0.712860669 | 0.99(0.939-1.044) |
| ECHDC2 | cg09128567 | Colorectal cancer || id:finn-b-C3_COLORECTAL | Weighted mode | 247 | 0.724505382 | 0.991(0.94-1.044) |
| ECHDC2 | cg09128567 | Colorectal cancer || id:finn-b-C3_COLORECTAL | MR Egger | 247 | 0.039209998 | 1.097(1.005-1.198) |
| VARS2 | cg10661769 | Colorectal cancer || id:finn-b-C3_COLORECTAL | Inverse variance weighted (fixed effects) | 2 | 0.813195256 | 1.023(0.848-1.235) |
| LIPT2 | cg11417029 | Colorectal cancer || id:finn-b-C3_COLORECTAL | Inverse variance weighted (fixed effects) | 112 | 3.08E-227 | 1.435(1.404-1.467) |
| LIPT2 | cg11417029 | Colorectal cancer || id:finn-b-C3_COLORECTAL | Weighted median | 112 | 1.33E-64 | 1.468(1.404-1.534) |
| LIPT2 | cg11417029 | Colorectal cancer || id:finn-b-C3_COLORECTAL | MR Egger | 112 | 0.085824461 | 0.658(0.41-1.056) |
| LIPT2 | cg11417029 | Colorectal cancer || id:finn-b-C3_COLORECTAL | Simple mode | 112 | 0.02474763 | 1.469(1.055-2.046) |
| LIPT2 | cg11417029 | Colorectal cancer || id:finn-b-C3_COLORECTAL | Weighted mode | 112 | 0.019004912 | 1.469(1.07-2.017) |
| MRPL28 | cg12437481 | Colorectal cancer || id:finn-b-C3_COLORECTAL | Weighted mode | 265 | 5.96E-23 | 1.064(1.052-1.076) |
| MRPL28 | cg12437481 | Colorectal cancer || id:finn-b-C3_COLORECTAL | MR Egger | 265 | 1.51E-15 | 1.044(1.034-1.054) |
| MRPL28 | cg12437481 | Colorectal cancer || id:finn-b-C3_COLORECTAL | Simple mode | 265 | 2.32E-16 | 1.07(1.054-1.087) |
| MRPL28 | cg12437481 | Colorectal cancer || id:finn-b-C3_COLORECTAL | Weighted median | 265 | 8.42E-49 | 1.068(1.059-1.077) |
| MRPL28 | cg12437481 | Colorectal cancer || id:finn-b-C3_COLORECTAL | Inverse variance weighted (fixed effects) | 265 | 8.05E-135 | 1.073(1.067-1.079) |
| ACADS | cg13914990 | Colorectal cancer || id:finn-b-C3_COLORECTAL | Inverse variance weighted (fixed effects) | 620 | 8.56E-27 | 0.966(0.96-0.972) |
| ACADS | cg13914990 | Colorectal cancer || id:finn-b-C3_COLORECTAL | Weighted mode | 620 | 0.012323045 | 0.972(0.951-0.994) |
| ACADS | cg13914990 | Colorectal cancer || id:finn-b-C3_COLORECTAL | Weighted median | 620 | 1.46E-09 | 0.974(0.965-0.982) |
| ACADS | cg13914990 | Colorectal cancer || id:finn-b-C3_COLORECTAL | MR Egger | 620 | 1.96E-15 | 0.914(0.895-0.934) |
| ACADS | cg13914990 | Colorectal cancer || id:finn-b-C3_COLORECTAL | Simple mode | 620 | 0.040951786 | 0.971(0.943-0.999) |
| NSUN4 | cg14993813 | Colorectal cancer || id:finn-b-C3_COLORECTAL | Inverse variance weighted (fixed effects) | 253 | 0.833316156 | 1.001(0.99-1.012) |
| NSUN4 | cg14993813 | Colorectal cancer || id:finn-b-C3_COLORECTAL | Weighted median | 253 | 0.649238538 | 1.003(0.991-1.015) |
| NSUN4 | cg14993813 | Colorectal cancer || id:finn-b-C3_COLORECTAL | Weighted mode | 253 | 0.89840864 | 1.003(0.964-1.042) |
| NSUN4 | cg14993813 | Colorectal cancer || id:finn-b-C3_COLORECTAL | MR Egger | 253 | 0.013865071 | 1.108(1.022-1.202) |
| NSUN4 | cg14993813 | Colorectal cancer || id:finn-b-C3_COLORECTAL | Simple mode | 253 | 0.905144814 | 1.003(0.962-1.045) |
| COX15 | cg17885402 | Colorectal cancer || id:finn-b-C3_COLORECTAL | MR Egger | 302 | 0.199677981 | 0.971(0.93-1.015) |
| COX15 | cg17885402 | Colorectal cancer || id:finn-b-C3_COLORECTAL | Weighted median | 302 | 7.98E-12 | 0.941(0.925-0.958) |
| COX15 | cg17885402 | Colorectal cancer || id:finn-b-C3_COLORECTAL | Simple mode | 302 | 0.024082527 | 0.939(0.89-0.992) |
| COX15 | cg17885402 | Colorectal cancer || id:finn-b-C3_COLORECTAL | Weighted mode | 302 | 0.003949211 | 0.937(0.897-0.979) |
| PNKD | cg18259342 | Colorectal cancer || id:finn-b-C3_COLORECTAL | Simple mode | 375 | 4.49E-12 | 1.098(1.07-1.127) |
| PNKD | cg18259342 | Colorectal cancer || id:finn-b-C3_COLORECTAL | Weighted mode | 375 | 8.43E-13 | 1.089(1.065-1.114) |
| PNKD | cg18259342 | Colorectal cancer || id:finn-b-C3_COLORECTAL | Weighted median | 375 | 8.22E-82 | 1.092(1.083-1.102) |
| PNKD | cg18259342 | Colorectal cancer || id:finn-b-C3_COLORECTAL | Inverse variance weighted (fixed effects) | 375 | 0 | 1.095(1.089-1.1) |
| PNKD | cg18259342 | Colorectal cancer || id:finn-b-C3_COLORECTAL | MR Egger | 375 | 3.12E-16 | 1.071(1.054-1.088) |
| COX10 | cg20170777 | Colorectal cancer || id:finn-b-C3_COLORECTAL | Weighted mode | 47 | 0.916116606 | 0.995(0.904-1.095) |
| COX10 | cg20170777 | Colorectal cancer || id:finn-b-C3_COLORECTAL | Simple mode | 47 | 0.915167639 | 0.995(0.905-1.094) |
| COX10 | cg20170777 | Colorectal cancer || id:finn-b-C3_COLORECTAL | Inverse variance weighted (fixed effects) | 47 | 0.284904219 | 1.021(0.983-1.06) |
| COX10 | cg20170777 | Colorectal cancer || id:finn-b-C3_COLORECTAL | MR Egger | 47 | 0.923395446 | 0.982(0.676-1.426) |
| COX10 | cg20170777 | Colorectal cancer || id:finn-b-C3_COLORECTAL | Weighted median | 47 | 0.98674603 | 1(0.954-1.048) |
| CASP9 | cg21858823 | Colorectal cancer || id:finn-b-C3_COLORECTAL | Weighted mode | 8 | 0.44521358 | 1.144(0.826-1.585) |
| CASP9 | cg21858823 | Colorectal cancer || id:finn-b-C3_COLORECTAL | Simple mode | 8 | 0.463416101 | 1.148(0.81-1.625) |
| CASP9 | cg21858823 | Colorectal cancer || id:finn-b-C3_COLORECTAL | Inverse variance weighted (fixed effects) | 8 | 1.16E-05 | 1.232(1.122-1.353) |
| CASP9 | cg21858823 | Colorectal cancer || id:finn-b-C3_COLORECTAL | MR Egger | 8 | 0.609002764 | 1.473(0.361-6.016) |
| CASP9 | cg21858823 | Colorectal cancer || id:finn-b-C3_COLORECTAL | Weighted median | 8 | 0.249608666 | 1.141(0.912-1.427) |
| BAD | cg23796481 | Colorectal cancer || id:finn-b-C3_COLORECTAL | Inverse variance weighted (fixed effects) | 57 | 3.30E-19 | 1.072(1.056-1.088) |
| BAD | cg23796481 | Colorectal cancer || id:finn-b-C3_COLORECTAL | Simple mode | 57 | 0.005441295 | 1.089(1.028-1.153) |
| BAD | cg23796481 | Colorectal cancer || id:finn-b-C3_COLORECTAL | Weighted median | 57 | 7.13E-08 | 1.069(1.043-1.095) |
| BAD | cg23796481 | Colorectal cancer || id:finn-b-C3_COLORECTAL | MR Egger | 57 | 0.221081789 | 1.06(0.966-1.164) |
| BAD | cg23796481 | Colorectal cancer || id:finn-b-C3_COLORECTAL | Weighted mode | 57 | 0.02682737 | 1.062(1.008-1.118) |
| ABCD3 | cg24215727 | Colorectal cancer || id:finn-b-C3_COLORECTAL | Weighted mode | 80 | 0.085595739 | 0.901(0.8-1.013) |
| ABCD3 | cg24215727 | Colorectal cancer || id:finn-b-C3_COLORECTAL | Weighted median | 80 | 2.94E-11 | 0.905(0.879-0.932) |
| ABCD3 | cg24215727 | Colorectal cancer || id:finn-b-C3_COLORECTAL | Inverse variance weighted (fixed effects) | 80 | 9.10E-10 | 0.91(0.883-0.938) |
| ABCD3 | cg24215727 | Colorectal cancer || id:finn-b-C3_COLORECTAL | Simple mode | 80 | 0.124192845 | 0.899(0.787-1.028) |
| ABCD3 | cg24215727 | Colorectal cancer || id:finn-b-C3_COLORECTAL | MR Egger | 80 | 0.050398246 | 0.479(0.232-0.99) |
| CISD3 | cg27492942 | Colorectal cancer || id:finn-b-C3_COLORECTAL | Simple mode | 43 | 0.2968103 | 0.95(0.863-1.045) |
| CISD3 | cg27492942 | Colorectal cancer || id:finn-b-C3_COLORECTAL | Inverse variance weighted (fixed effects) | 43 | 0.676278053 | 1.008(0.971-1.046) |
| CISD3 | cg27492942 | Colorectal cancer || id:finn-b-C3_COLORECTAL | MR Egger | 43 | 0.05184339 | 0.869(0.758-0.997) |
| CISD3 | cg27492942 | Colorectal cancer || id:finn-b-C3_COLORECTAL | Weighted median | 43 | 0.074478395 | 0.957(0.913-1.004) |
| CISD3 | cg27492942 | Colorectal cancer || id:finn-b-C3_COLORECTAL | Weighted mode | 43 | 0.213983819 | 0.947(0.871-1.03) |
| SLC25A30 | cg00139037 | Colorectal cancer (all cancers excluded) || id:finn-b-C3_COLORECTAL_EXALLC | Inverse variance weighted (fixed effects) | 77 | 5.18E-11 | 1.108(1.075-1.143) |
| SLC25A30 | cg00139037 | Colorectal cancer (all cancers excluded) || id:finn-b-C3_COLORECTAL_EXALLC | Simple mode | 77 | 0.2174297 | 1.108(0.943-1.301) |
| SLC25A30 | cg00139037 | Colorectal cancer (all cancers excluded) || id:finn-b-C3_COLORECTAL_EXALLC | MR Egger | 77 | 0.659851864 | 1.067(0.8-1.423) |
| SLC25A30 | cg00139037 | Colorectal cancer (all cancers excluded) || id:finn-b-C3_COLORECTAL_EXALLC | Weighted median | 77 | 1.50E-13 | 1.098(1.071-1.126) |
| SLC25A30 | cg00139037 | Colorectal cancer (all cancers excluded) || id:finn-b-C3_COLORECTAL_EXALLC | Weighted mode | 77 | 0.215358111 | 1.098(0.948-1.27) |
| MRPL32 | cg00365680 | Colorectal cancer (all cancers excluded) || id:finn-b-C3_COLORECTAL_EXALLC | Inverse variance weighted (fixed effects) | 199 | 1.74E-61 | 1.119(1.105-1.134) |
| MRPL32 | cg00365680 | Colorectal cancer (all cancers excluded) || id:finn-b-C3_COLORECTAL_EXALLC | MR Egger | 199 | 4.64E-26 | 1.243(1.201-1.288) |
| MRPL32 | cg00365680 | Colorectal cancer (all cancers excluded) || id:finn-b-C3_COLORECTAL_EXALLC | Weighted median | 199 | 1.56E-37 | 1.131(1.109-1.152) |
| MRPL32 | cg00365680 | Colorectal cancer (all cancers excluded) || id:finn-b-C3_COLORECTAL_EXALLC | Simple mode | 199 | 1.22E-08 | 1.156(1.102-1.213) |
| MRPL32 | cg00365680 | Colorectal cancer (all cancers excluded) || id:finn-b-C3_COLORECTAL_EXALLC | Weighted mode | 199 | 5.14E-07 | 1.114(1.07-1.16) |
| ACSF3 | cg02193283 | Colorectal cancer (all cancers excluded) || id:finn-b-C3_COLORECTAL_EXALLC | Simple mode | 336 | 0.131681724 | 1.037(0.989-1.088) |
| ACSF3 | cg02193283 | Colorectal cancer (all cancers excluded) || id:finn-b-C3_COLORECTAL_EXALLC | Weighted mode | 336 | 0.17021872 | 1.024(0.99-1.059) |
| ACSF3 | cg02193283 | Colorectal cancer (all cancers excluded) || id:finn-b-C3_COLORECTAL_EXALLC | MR Egger | 336 | 7.23E-07 | 1.178(1.106-1.256) |
| ACSF3 | cg02193283 | Colorectal cancer (all cancers excluded) || id:finn-b-C3_COLORECTAL_EXALLC | Weighted median | 336 | 8.59E-06 | 1.043(1.024-1.062) |
| ME3 | cg02493602 | Colorectal cancer (all cancers excluded) || id:finn-b-C3_COLORECTAL_EXALLC | Weighted median | 228 | 0.48267393 | 0.995(0.981-1.009) |
| ME3 | cg02493602 | Colorectal cancer (all cancers excluded) || id:finn-b-C3_COLORECTAL_EXALLC | Inverse variance weighted (fixed effects) | 228 | 0.000293413 | 0.981(0.971-0.991) |
| ME3 | cg02493602 | Colorectal cancer (all cancers excluded) || id:finn-b-C3_COLORECTAL_EXALLC | Simple mode | 228 | 0.947374875 | 0.999(0.967-1.032) |
| ME3 | cg02493602 | Colorectal cancer (all cancers excluded) || id:finn-b-C3_COLORECTAL_EXALLC | MR Egger | 228 | 0.018325559 | 0.966(0.939-0.994) |
| ME3 | cg02493602 | Colorectal cancer (all cancers excluded) || id:finn-b-C3_COLORECTAL_EXALLC | Weighted mode | 228 | 0.787192728 | 0.997(0.972-1.021) |
| SCP2 | cg03579872 | Colorectal cancer (all cancers excluded) || id:finn-b-C3_COLORECTAL_EXALLC | Simple mode | 312 | 0.885066207 | 0.998(0.966-1.031) |
| SCP2 | cg03579872 | Colorectal cancer (all cancers excluded) || id:finn-b-C3_COLORECTAL_EXALLC | Weighted mode | 312 | 0.879096714 | 0.998(0.967-1.029) |
| SCP2 | cg03579872 | Colorectal cancer (all cancers excluded) || id:finn-b-C3_COLORECTAL_EXALLC | Inverse variance weighted (fixed effects) | 312 | 0.218550677 | 1.005(0.997-1.013) |
| SCP2 | cg03579872 | Colorectal cancer (all cancers excluded) || id:finn-b-C3_COLORECTAL_EXALLC | Weighted median | 312 | 0.67283375 | 1.002(0.993-1.01) |
| SCP2 | cg03579872 | Colorectal cancer (all cancers excluded) || id:finn-b-C3_COLORECTAL_EXALLC | MR Egger | 312 | 1.83E-07 | 1.118(1.073-1.164) |
| ACSF3 | cg04308346 | Colorectal cancer (all cancers excluded) || id:finn-b-C3_COLORECTAL_EXALLC | Weighted median | 10 | 0.066611954 | 0.861(0.733-1.01) |
| ACSF3 | cg04308346 | Colorectal cancer (all cancers excluded) || id:finn-b-C3_COLORECTAL_EXALLC | MR Egger | 10 | 0.822736358 | 1.408(0.078-25.492) |
| ACSF3 | cg04308346 | Colorectal cancer (all cancers excluded) || id:finn-b-C3_COLORECTAL_EXALLC | Inverse variance weighted (fixed effects) | 10 | 0.00017982 | 0.86(0.795-0.931) |
| ACSF3 | cg04308346 | Colorectal cancer (all cancers excluded) || id:finn-b-C3_COLORECTAL_EXALLC | Simple mode | 10 | 0.304276447 | 0.858(0.652-1.13) |
| ACSF3 | cg04308346 | Colorectal cancer (all cancers excluded) || id:finn-b-C3_COLORECTAL_EXALLC | Weighted mode | 10 | 0.272685306 | 0.858(0.664-1.109) |
| MSRB2 | cg07355157 | Colorectal cancer (all cancers excluded) || id:finn-b-C3_COLORECTAL_EXALLC | Inverse variance weighted (fixed effects) | 156 | 0.191284933 | 0.993(0.983-1.003) |
| MSRB2 | cg07355157 | Colorectal cancer (all cancers excluded) || id:finn-b-C3_COLORECTAL_EXALLC | Simple mode | 156 | 0.002468754 | 0.959(0.934-0.985) |
| MSRB2 | cg07355157 | Colorectal cancer (all cancers excluded) || id:finn-b-C3_COLORECTAL_EXALLC | Weighted median | 156 | 0.001744954 | 0.978(0.964-0.992) |
| MSRB2 | cg07355157 | Colorectal cancer (all cancers excluded) || id:finn-b-C3_COLORECTAL_EXALLC | Weighted mode | 156 | 0.020908729 | 0.978(0.96-0.996) |
| MSRB2 | cg07355157 | Colorectal cancer (all cancers excluded) || id:finn-b-C3_COLORECTAL_EXALLC | MR Egger | 156 | 0.306432996 | 1.011(0.99-1.032) |
| CYB5R3 | cg08690876 | Colorectal cancer (all cancers excluded) || id:finn-b-C3_COLORECTAL_EXALLC | Simple mode | 347 | 1.20E-06 | 1.09(1.054-1.128) |
| CYB5R3 | cg08690876 | Colorectal cancer (all cancers excluded) || id:finn-b-C3_COLORECTAL_EXALLC | Inverse variance weighted (fixed effects) | 347 | 9.73E-89 | 1.081(1.073-1.09) |
| CYB5R3 | cg08690876 | Colorectal cancer (all cancers excluded) || id:finn-b-C3_COLORECTAL_EXALLC | Weighted median | 347 | 8.69E-28 | 1.074(1.06-1.088) |
| CYB5R3 | cg08690876 | Colorectal cancer (all cancers excluded) || id:finn-b-C3_COLORECTAL_EXALLC | MR Egger | 347 | 5.01E-07 | 1.056(1.034-1.078) |
| CYB5R3 | cg08690876 | Colorectal cancer (all cancers excluded) || id:finn-b-C3_COLORECTAL_EXALLC | Weighted mode | 347 | 9.78E-08 | 1.079(1.05-1.109) |
| VARS2 | cg10661769 | Colorectal cancer (all cancers excluded) || id:finn-b-C3_COLORECTAL_EXALLC | Inverse variance weighted (fixed effects) | 2 | 0.601434893 | 1.052(0.871-1.271) |
| LIPT2 | cg11417029 | Colorectal cancer (all cancers excluded) || id:finn-b-C3_COLORECTAL_EXALLC | Weighted median | 113 | 3.50E-60 | 1.45(1.387-1.516) |
| LIPT2 | cg11417029 | Colorectal cancer (all cancers excluded) || id:finn-b-C3_COLORECTAL_EXALLC | MR Egger | 113 | 0.125776481 | 0.69(0.43-1.106) |
| LIPT2 | cg11417029 | Colorectal cancer (all cancers excluded) || id:finn-b-C3_COLORECTAL_EXALLC | Simple mode | 113 | 0.031285065 | 1.452(1.038-2.029) |
| LIPT2 | cg11417029 | Colorectal cancer (all cancers excluded) || id:finn-b-C3_COLORECTAL_EXALLC | Inverse variance weighted (fixed effects) | 113 | 1.04E-211 | 1.421(1.39-1.453) |
| LIPT2 | cg11417029 | Colorectal cancer (all cancers excluded) || id:finn-b-C3_COLORECTAL_EXALLC | Weighted mode | 113 | 0.023591856 | 1.452(1.056-1.996) |
| MRPL28 | cg12437481 | Colorectal cancer (all cancers excluded) || id:finn-b-C3_COLORECTAL_EXALLC | Weighted median | 264 | 6.34E-42 | 1.064(1.054-1.073) |
| MRPL28 | cg12437481 | Colorectal cancer (all cancers excluded) || id:finn-b-C3_COLORECTAL_EXALLC | MR Egger | 264 | 1.09E-11 | 1.038(1.027-1.048) |
| MRPL28 | cg12437481 | Colorectal cancer (all cancers excluded) || id:finn-b-C3_COLORECTAL_EXALLC | Simple mode | 264 | 4.30E-15 | 1.069(1.053-1.086) |
| MRPL28 | cg12437481 | Colorectal cancer (all cancers excluded) || id:finn-b-C3_COLORECTAL_EXALLC | Inverse variance weighted (fixed effects) | 264 | 2.59E-126 | 1.072(1.066-1.078) |
| MRPL28 | cg12437481 | Colorectal cancer (all cancers excluded) || id:finn-b-C3_COLORECTAL_EXALLC | Simple mode | 264 | 5.19E-15 | 1.069(1.052-1.086) |
| MRPL28 | cg12437481 | Colorectal cancer (all cancers excluded) || id:finn-b-C3_COLORECTAL_EXALLC | Weighted median | 264 | 2.94E-40 | 1.064(1.054-1.073) |
| MRPL28 | cg12437481 | Colorectal cancer (all cancers excluded) || id:finn-b-C3_COLORECTAL_EXALLC | Weighted mode | 264 | 9.02E-21 | 1.061(1.049-1.073) |
| MRPL28 | cg12437481 | Colorectal cancer (all cancers excluded) || id:finn-b-C3_COLORECTAL_EXALLC | Weighted mode | 264 | 1.07E-21 | 1.061(1.049-1.073) |
| ACADS | cg13914990 | Colorectal cancer (all cancers excluded) || id:finn-b-C3_COLORECTAL_EXALLC | Weighted median | 621 | 0.000255055 | 0.983(0.974-0.992) |
| ACADS | cg13914990 | Colorectal cancer (all cancers excluded) || id:finn-b-C3_COLORECTAL_EXALLC | Simple mode | 621 | 0.138731875 | 0.98(0.953-1.007) |
| ACADS | cg13914990 | Colorectal cancer (all cancers excluded) || id:finn-b-C3_COLORECTAL_EXALLC | Weighted mode | 621 | 0.116811023 | 0.981(0.958-1.005) |
| ACADS | cg13914990 | Colorectal cancer (all cancers excluded) || id:finn-b-C3_COLORECTAL_EXALLC | MR Egger | 621 | 3.21E-14 | 0.917(0.897-0.937) |
| ACADS | cg13914990 | Colorectal cancer (all cancers excluded) || id:finn-b-C3_COLORECTAL_EXALLC | Inverse variance weighted (fixed effects) | 621 | 1.61E-18 | 0.971(0.965-0.978) |
| NSUN4 | cg14993813 | Colorectal cancer (all cancers excluded) || id:finn-b-C3_COLORECTAL_EXALLC | Weighted median | 253 | 0.000498669 | 0.978(0.966-0.99) |
| NSUN4 | cg14993813 | Colorectal cancer (all cancers excluded) || id:finn-b-C3_COLORECTAL_EXALLC | Simple mode | 253 | 0.2842215 | 0.978(0.939-1.019) |
| NSUN4 | cg14993813 | Colorectal cancer (all cancers excluded) || id:finn-b-C3_COLORECTAL_EXALLC | MR Egger | 253 | 0.051513834 | 1.085(1-1.178) |
| NSUN4 | cg14993813 | Colorectal cancer (all cancers excluded) || id:finn-b-C3_COLORECTAL_EXALLC | Weighted mode | 253 | 0.254884212 | 0.978(0.941-1.016) |
| NSUN4 | cg14993813 | Colorectal cancer (all cancers excluded) || id:finn-b-C3_COLORECTAL_EXALLC | Inverse variance weighted (fixed effects) | 253 | 8.83E-06 | 0.976(0.965-0.986) |
| COX15 | cg17885402 | Colorectal cancer (all cancers excluded) || id:finn-b-C3_COLORECTAL_EXALLC | MR Egger | 302 | 0.282887912 | 0.974(0.929-1.022) |
| COX15 | cg17885402 | Colorectal cancer (all cancers excluded) || id:finn-b-C3_COLORECTAL_EXALLC | Weighted mode | 302 | 0.01362303 | 0.944(0.903-0.988) |
| COX15 | cg17885402 | Colorectal cancer (all cancers excluded) || id:finn-b-C3_COLORECTAL_EXALLC | Weighted median | 302 | 1.65E-08 | 0.948(0.93-0.966) |
| COX15 | cg17885402 | Colorectal cancer (all cancers excluded) || id:finn-b-C3_COLORECTAL_EXALLC | Simple mode | 302 | 0.066146946 | 0.949(0.898-1.003) |
| PNKD | cg18259342 | Colorectal cancer (all cancers excluded) || id:finn-b-C3_COLORECTAL_EXALLC | MR Egger | 375 | 1.38E-16 | 1.073(1.056-1.09) |
| PNKD | cg18259342 | Colorectal cancer (all cancers excluded) || id:finn-b-C3_COLORECTAL_EXALLC | Weighted mode | 375 | 1.61E-11 | 1.086(1.061-1.112) |
| PNKD | cg18259342 | Colorectal cancer (all cancers excluded) || id:finn-b-C3_COLORECTAL_EXALLC | Inverse variance weighted (fixed effects) | 375 | 0 | 1.096(1.091-1.101) |
| PNKD | cg18259342 | Colorectal cancer (all cancers excluded) || id:finn-b-C3_COLORECTAL_EXALLC | Simple mode | 375 | 6.82E-11 | 1.097(1.068-1.127) |
| PNKD | cg18259342 | Colorectal cancer (all cancers excluded) || id:finn-b-C3_COLORECTAL_EXALLC | Weighted median | 375 | 8.07E-81 | 1.093(1.083-1.103) |
| COX10 | cg20170777 | Colorectal cancer (all cancers excluded) || id:finn-b-C3_COLORECTAL_EXALLC | Weighted mode | 47 | 0.367949076 | 0.954(0.862-1.056) |
| COX10 | cg20170777 | Colorectal cancer (all cancers excluded) || id:finn-b-C3_COLORECTAL_EXALLC | Simple mode | 47 | 0.332076345 | 0.952(0.863-1.05) |
| COX10 | cg20170777 | Colorectal cancer (all cancers excluded) || id:finn-b-C3_COLORECTAL_EXALLC | Inverse variance weighted (fixed effects) | 47 | 0.282334121 | 0.979(0.943-1.017) |
| COX10 | cg20170777 | Colorectal cancer (all cancers excluded) || id:finn-b-C3_COLORECTAL_EXALLC | MR Egger | 47 | 0.794403192 | 0.951(0.652-1.387) |
| COX10 | cg20170777 | Colorectal cancer (all cancers excluded) || id:finn-b-C3_COLORECTAL_EXALLC | Weighted median | 47 | 0.08884035 | 0.96(0.917-1.006) |
| BAD | cg23796481 | Colorectal cancer (all cancers excluded) || id:finn-b-C3_COLORECTAL_EXALLC | Simple mode | 57 | 0.002045788 | 1.103(1.039-1.17) |
| BAD | cg23796481 | Colorectal cancer (all cancers excluded) || id:finn-b-C3_COLORECTAL_EXALLC | MR Egger | 57 | 0.29985027 | 1.051(0.957-1.155) |
| BAD | cg23796481 | Colorectal cancer (all cancers excluded) || id:finn-b-C3_COLORECTAL_EXALLC | Inverse variance weighted (fixed effects) | 57 | 4.64E-24 | 1.082(1.066-1.099) |
| BAD | cg23796481 | Colorectal cancer (all cancers excluded) || id:finn-b-C3_COLORECTAL_EXALLC | Weighted mode | 57 | 0.001176484 | 1.103(1.043-1.167) |
| BAD | cg23796481 | Colorectal cancer (all cancers excluded) || id:finn-b-C3_COLORECTAL_EXALLC | Weighted median | 57 | 6.71E-08 | 1.071(1.045-1.098) |
| ABCD3 | cg24215727 | Colorectal cancer (all cancers excluded) || id:finn-b-C3_COLORECTAL_EXALLC | Weighted mode | 80 | 0.834078989 | 0.988(0.887-1.102) |
| ABCD3 | cg24215727 | Colorectal cancer (all cancers excluded) || id:finn-b-C3_COLORECTAL_EXALLC | Inverse variance weighted (fixed effects) | 80 | 2.63E-16 | 0.88(0.853-0.907) |
| ABCD3 | cg24215727 | Colorectal cancer (all cancers excluded) || id:finn-b-C3_COLORECTAL_EXALLC | Weighted median | 80 | 0.045164531 | 0.966(0.933-0.999) |
| ABCD3 | cg24215727 | Colorectal cancer (all cancers excluded) || id:finn-b-C3_COLORECTAL_EXALLC | Simple mode | 80 | 0.033132162 | 0.86(0.751-0.986) |
| ABCD3 | cg24215727 | Colorectal cancer (all cancers excluded) || id:finn-b-C3_COLORECTAL_EXALLC | MR Egger | 80 | 0.030203856 | 0.437(0.209-0.911) |
| CISD3 | cg27492942 | Colorectal cancer (all cancers excluded) || id:finn-b-C3_COLORECTAL_EXALLC | MR Egger | 43 | 0.014758863 | 0.835(0.727-0.959) |
| CISD3 | cg27492942 | Colorectal cancer (all cancers excluded) || id:finn-b-C3_COLORECTAL_EXALLC | Weighted median | 43 | 0.016357463 | 0.941(0.895-0.989) |
| CISD3 | cg27492942 | Colorectal cancer (all cancers excluded) || id:finn-b-C3_COLORECTAL_EXALLC | Weighted mode | 43 | 0.10563128 | 0.93(0.854-1.013) |
| CISD3 | cg27492942 | Colorectal cancer (all cancers excluded) || id:finn-b-C3_COLORECTAL_EXALLC | Simple mode | 43 | 0.182959355 | 0.933(0.844-1.032) |
| CISD3 | cg27492942 | Colorectal cancer (all cancers excluded) || id:finn-b-C3_COLORECTAL_EXALLC | Inverse variance weighted (fixed effects) | 43 | 0.912782473 | 0.998(0.961-1.036) |
| SLC25A30 | cg00139037 | Colorectal cancer || id:ieu-b-4965 | MR Egger | 76 | 0.080083075 | 1.003(1-1.006) |
| SLC25A30 | cg00139037 | Colorectal cancer || id:ieu-b-4965 | Weighted median | 76 | 2.30E-28 | 1.002(1.002-1.002) |
| SLC25A30 | cg00139037 | Colorectal cancer || id:ieu-b-4965 | Inverse variance weighted (fixed effects) | 76 | 4.13E-37 | 1.002(1.002-1.002) |
| SLC25A30 | cg00139037 | Colorectal cancer || id:ieu-b-4965 | Simple mode | 76 | 0.110042538 | 1.002(1-1.005) |
| SLC25A30 | cg00139037 | Colorectal cancer || id:ieu-b-4965 | Weighted mode | 76 | 0.087671613 | 1.002(1-1.005) |
| MRPL32 | cg00365680 | Colorectal cancer || id:ieu-b-4965 | MR Egger | 198 | 0.97489465 | 1(1-1) |
| MRPL32 | cg00365680 | Colorectal cancer || id:ieu-b-4965 | Inverse variance weighted (fixed effects) | 198 | 8.56E-07 | 1(1-1) |
| MRPL32 | cg00365680 | Colorectal cancer || id:ieu-b-4965 | Simple mode | 198 | 0.007265083 | 1(1-1.001) |
| MRPL32 | cg00365680 | Colorectal cancer || id:ieu-b-4965 | Weighted median | 198 | 4.30E-09 | 1(1-1.001) |
| MRPL32 | cg00365680 | Colorectal cancer || id:ieu-b-4965 | Weighted mode | 198 | 0.00426979 | 1(1-1.001) |
| ACSF3 | cg02193283 | Colorectal cancer || id:ieu-b-4965 | Inverse variance weighted (fixed effects) | 334 | 2.69E-94 | 0.999(0.999-0.999) |
| ACSF3 | cg02193283 | Colorectal cancer || id:ieu-b-4965 | Simple mode | 334 | 5.44E-05 | 0.999(0.998-0.999) |
| ACSF3 | cg02193283 | Colorectal cancer || id:ieu-b-4965 | Weighted mode | 334 | 3.77E-05 | 0.999(0.999-0.999) |
| ACSF3 | cg02193283 | Colorectal cancer || id:ieu-b-4965 | Weighted median | 334 | 3.65E-35 | 0.999(0.999-0.999) |
| ACSF3 | cg02193283 | Colorectal cancer || id:ieu-b-4965 | MR Egger | 334 | 0.001578316 | 0.999(0.999-1) |
| ME3 | cg02493602 | Colorectal cancer || id:ieu-b-4965 | Simple mode | 225 | 0.334376829 | 1(0.999-1) |
| ME3 | cg02493602 | Colorectal cancer || id:ieu-b-4965 | Weighted mode | 225 | 0.205568213 | 1(0.999-1) |
| ME3 | cg02493602 | Colorectal cancer || id:ieu-b-4965 | Weighted median | 225 | 0.228349607 | 1(1-1) |
| ME3 | cg02493602 | Colorectal cancer || id:ieu-b-4965 | Inverse variance weighted (fixed effects) | 225 | 0.083004108 | 1(1-1) |
| ME3 | cg02493602 | Colorectal cancer || id:ieu-b-4965 | MR Egger | 225 | 1.50E-11 | 1.001(1.001-1.001) |
| SCP2 | cg03579872 | Colorectal cancer || id:ieu-b-4965 | Inverse variance weighted (fixed effects) | 330 | 0 | 1.002(1.002-1.002) |
| SCP2 | cg03579872 | Colorectal cancer || id:ieu-b-4965 | Weighted median | 330 | 9.04E-74 | 1.002(1.001-1.002) |
| SCP2 | cg03579872 | Colorectal cancer || id:ieu-b-4965 | Weighted mode | 330 | 0.00022235 | 1.001(1.001-1.002) |
| SCP2 | cg03579872 | Colorectal cancer || id:ieu-b-4965 | MR Egger | 330 | 6.14E-34 | 1.003(1.003-1.003) |
| SCP2 | cg03579872 | Colorectal cancer || id:ieu-b-4965 | Simple mode | 330 | 0.000692108 | 1.001(1.001-1.002) |
| ACSF3 | cg04308346 | Colorectal cancer || id:ieu-b-4965 | Inverse variance weighted (fixed effects) | 10 | 0.166053998 | 1.001(1-1.001) |
| ACSF3 | cg04308346 | Colorectal cancer || id:ieu-b-4965 | Weighted mode | 10 | 0.654553503 | 1(0.999-1.002) |
| ACSF3 | cg04308346 | Colorectal cancer || id:ieu-b-4965 | Simple mode | 10 | 0.678116441 | 1(0.999-1.002) |
| ACSF3 | cg04308346 | Colorectal cancer || id:ieu-b-4965 | Weighted median | 10 | 0.302649009 | 1(1-1.001) |
| ACSF3 | cg04308346 | Colorectal cancer || id:ieu-b-4965 | MR Egger | 10 | 0.783551976 | 1.004(0.975-1.034) |
| MSRB2 | cg07355157 | Colorectal cancer || id:ieu-b-4965 | Weighted median | 155 | 2.18E-26 | 1.001(1.001-1.001) |
| MSRB2 | cg07355157 | Colorectal cancer || id:ieu-b-4965 | Inverse variance weighted (fixed effects) | 155 | 6.27E-72 | 1.001(1.001-1.001) |
| MSRB2 | cg07355157 | Colorectal cancer || id:ieu-b-4965 | MR Egger | 155 | 5.80E-23 | 1.001(1.001-1.002) |
| MSRB2 | cg07355157 | Colorectal cancer || id:ieu-b-4965 | Weighted mode | 155 | 3.21E-14 | 1.001(1.001-1.001) |
| MSRB2 | cg07355157 | Colorectal cancer || id:ieu-b-4965 | Simple mode | 155 | 3.29E-07 | 1.001(1.001-1.001) |
| CYB5R3 | cg08690876 | Colorectal cancer || id:ieu-b-4965 | Inverse variance weighted (fixed effects) | 347 | 0.932139913 | 1(1-1) |
| CYB5R3 | cg08690876 | Colorectal cancer || id:ieu-b-4965 | Simple mode | 347 | 0.896090383 | 1(1-1) |
| CYB5R3 | cg08690876 | Colorectal cancer || id:ieu-b-4965 | Weighted median | 347 | 0.73521214 | 1(1-1) |
| CYB5R3 | cg08690876 | Colorectal cancer || id:ieu-b-4965 | Weighted mode | 347 | 0.909171765 | 1(1-1) |
| CYB5R3 | cg08690876 | Colorectal cancer || id:ieu-b-4965 | MR Egger | 347 | 0.43323229 | 1(1-1) |
| LIPT2 | cg11417029 | Colorectal cancer || id:ieu-b-4965 | MR Egger | 114 | 0.09142167 | 1.004(0.999-1.009) |
| LIPT2 | cg11417029 | Colorectal cancer || id:ieu-b-4965 | Weighted median | 114 | 2.40E-18 | 1.003(1.002-1.004) |
| LIPT2 | cg11417029 | Colorectal cancer || id:ieu-b-4965 | Inverse variance weighted (fixed effects) | 114 | 1.02E-172 | 1.003(1.003-1.004) |
| LIPT2 | cg11417029 | Colorectal cancer || id:ieu-b-4965 | Weighted mode | 114 | 5.57E-06 | 1.004(1.002-1.005) |
| LIPT2 | cg11417029 | Colorectal cancer || id:ieu-b-4965 | Simple mode | 114 | 0.00500401 | 1.004(1.001-1.006) |
| MRPL28 | cg12437481 | Colorectal cancer || id:ieu-b-4965 | Weighted median | 256 | 3.88E-44 | 1.001(1.001-1.001) |
| MRPL28 | cg12437481 | Colorectal cancer || id:ieu-b-4965 | MR Egger | 256 | 1.80E-28 | 1.001(1.001-1.001) |
| MRPL28 | cg12437481 | Colorectal cancer || id:ieu-b-4965 | Inverse variance weighted (fixed effects) | 256 | 7.22E-85 | 1.001(1.001-1.001) |
| MRPL28 | cg12437481 | Colorectal cancer || id:ieu-b-4965 | Weighted mode | 256 | 1.68E-23 | 1.001(1.001-1.001) |
| MRPL28 | cg12437481 | Colorectal cancer || id:ieu-b-4965 | Simple mode | 256 | 1.10E-11 | 1.001(1-1.001) |
| ACADS | cg13914990 | Colorectal cancer || id:ieu-b-4965 | Weighted mode | 620 | 1.02E-07 | 1.001(1.001-1.001) |
| ACADS | cg13914990 | Colorectal cancer || id:ieu-b-4965 | Simple mode | 620 | 4.06E-05 | 1.001(1-1.001) |
| ACADS | cg13914990 | Colorectal cancer || id:ieu-b-4965 | Inverse variance weighted (fixed effects) | 620 | 5.88E-145 | 1.001(1.001-1.001) |
| ACADS | cg13914990 | Colorectal cancer || id:ieu-b-4965 | Weighted median | 620 | 3.09E-54 | 1.001(1.001-1.001) |
| ACADS | cg13914990 | Colorectal cancer || id:ieu-b-4965 | MR Egger | 620 | 1.49E-06 | 1.001(1-1.001) |
| NSUN4 | cg14993813 | Colorectal cancer || id:ieu-b-4965 | Weighted median | 256 | 7.47E-62 | 1.002(1.002-1.002) |
| NSUN4 | cg14993813 | Colorectal cancer || id:ieu-b-4965 | Weighted mode | 256 | 1.10E-05 | 1.002(1.001-1.003) |
| NSUN4 | cg14993813 | Colorectal cancer || id:ieu-b-4965 | Simple mode | 256 | 2.24E-05 | 1.002(1.001-1.003) |
| NSUN4 | cg14993813 | Colorectal cancer || id:ieu-b-4965 | MR Egger | 256 | 0.0183106 | 1.001(1-1.002) |
| NSUN4 | cg14993813 | Colorectal cancer || id:ieu-b-4965 | Inverse variance weighted (fixed effects) | 256 | 2.30E-222 | 1.002(1.002-1.002) |
| COX15 | cg17885402 | Colorectal cancer || id:ieu-b-4965 | MR Egger | 303 | 0.019124601 | 1.001(1-1.001) |
| COX15 | cg17885402 | Colorectal cancer || id:ieu-b-4965 | Simple mode | 303 | 3.09E-38 | 0.996(0.996-0.997) |
| COX15 | cg17885402 | Colorectal cancer || id:ieu-b-4965 | Weighted mode | 303 | 2.41E-06 | 0.999(0.999-0.999) |
| COX15 | cg17885402 | Colorectal cancer || id:ieu-b-4965 | Weighted median | 303 | 4.89E-23 | 0.999(0.999-0.999) |
| PNKD | cg18259342 | Colorectal cancer || id:ieu-b-4965 | Weighted median | 362 | 1.41E-124 | 1.001(1.001-1.001) |
| PNKD | cg18259342 | Colorectal cancer || id:ieu-b-4965 | Simple mode | 362 | 4.18E-12 | 1.001(1.001-1.002) |
| PNKD | cg18259342 | Colorectal cancer || id:ieu-b-4965 | MR Egger | 362 | 3.80E-59 | 1.002(1.001-1.002) |
| PNKD | cg18259342 | Colorectal cancer || id:ieu-b-4965 | Inverse variance weighted (fixed effects) | 362 | 0 | 1.001(1.001-1.001) |
| PNKD | cg18259342 | Colorectal cancer || id:ieu-b-4965 | Weighted mode | 362 | 2.74E-13 | 1.001(1.001-1.002) |
| COX10 | cg20170777 | Colorectal cancer || id:ieu-b-4965 | Weighted mode | 47 | 0.015073026 | 0.998(0.996-1) |
| COX10 | cg20170777 | Colorectal cancer || id:ieu-b-4965 | Inverse variance weighted (fixed effects) | 47 | 3.72E-22 | 0.998(0.998-0.998) |
| COX10 | cg20170777 | Colorectal cancer || id:ieu-b-4965 | Simple mode | 47 | 0.008650488 | 0.998(0.996-0.999) |
| COX10 | cg20170777 | Colorectal cancer || id:ieu-b-4965 | Weighted median | 47 | 3.59E-10 | 0.998(0.997-0.999) |
| COX10 | cg20170777 | Colorectal cancer || id:ieu-b-4965 | MR Egger | 47 | 0.007855448 | 0.994(0.991-0.998) |
| CASP9 | cg21858823 | Colorectal cancer || id:ieu-b-4965 | Weighted median | 8 | 0.052804012 | 1.001(1-1.003) |
| CASP9 | cg21858823 | Colorectal cancer || id:ieu-b-4965 | Weighted mode | 8 | 0.501266782 | 1.001(0.998-1.004) |
| CASP9 | cg21858823 | Colorectal cancer || id:ieu-b-4965 | Simple mode | 8 | 0.351249993 | 1.002(0.998-1.005) |
| CASP9 | cg21858823 | Colorectal cancer || id:ieu-b-4965 | Inverse variance weighted (fixed effects) | 8 | 0.002167358 | 1.001(1.001-1.002) |
| CASP9 | cg21858823 | Colorectal cancer || id:ieu-b-4965 | MR Egger | 8 | 0.828723265 | 1.002(0.984-1.02) |
| BAD | cg23796481 | Colorectal cancer || id:ieu-b-4965 | Simple mode | 60 | 0.000503554 | 1.001(1.001-1.002) |
| BAD | cg23796481 | Colorectal cancer || id:ieu-b-4965 | Weighted mode | 60 | 0.00784431 | 1.001(1-1.001) |
| BAD | cg23796481 | Colorectal cancer || id:ieu-b-4965 | Inverse variance weighted (fixed effects) | 60 | 6.10E-46 | 1.001(1.001-1.001) |
| BAD | cg23796481 | Colorectal cancer || id:ieu-b-4965 | Weighted median | 60 | 1.55E-12 | 1.001(1.001-1.001) |
| BAD | cg23796481 | Colorectal cancer || id:ieu-b-4965 | MR Egger | 60 | 0.995424056 | 1(0.999-1.001) |
| ABCD3 | cg24215727 | Colorectal cancer || id:ieu-b-4965 | Inverse variance weighted (fixed effects) | 93 | 0.003579565 | 1(0.999-1) |
| ABCD3 | cg24215727 | Colorectal cancer || id:ieu-b-4965 | Weighted median | 93 | 2.17E-07 | 0.999(0.999-1) |
| ABCD3 | cg24215727 | Colorectal cancer || id:ieu-b-4965 | Weighted mode | 93 | 0.185628678 | 0.999(0.998-1) |
| ABCD3 | cg24215727 | Colorectal cancer || id:ieu-b-4965 | Simple mode | 93 | 0.137199556 | 0.999(0.998-1) |
| ABCD3 | cg24215727 | Colorectal cancer || id:ieu-b-4965 | MR Egger | 93 | 0.009608615 | 0.991(0.984-0.998) |
| CISD3 | cg27492942 | Colorectal cancer || id:ieu-b-4965 | Weighted mode | 42 | 0.046483208 | 0.999(0.998-1) |
| CISD3 | cg27492942 | Colorectal cancer || id:ieu-b-4965 | Simple mode | 42 | 0.075249348 | 0.999(0.998-1) |
| CISD3 | cg27492942 | Colorectal cancer || id:ieu-b-4965 | Weighted median | 42 | 0.004306504 | 0.999(0.999-1) |
| CISD3 | cg27492942 | Colorectal cancer || id:ieu-b-4965 | MR Egger | 42 | 0.425537346 | 0.999(0.998-1.001) |
| CISD3 | cg27492942 | Colorectal cancer || id:ieu-b-4965 | Inverse variance weighted (fixed effects) | 42 | 0.000156133 | 0.999(0.999-1) |

**Supplementary Table 5.** SMR and colocalization results of the association between mitochondria-related genes mQTL and CRC outcomes.

| **Gene** | **Probe ID** | **Probe Chr** | **Probe bp** | **TopSNP** | **TopSNP chr** | **TopSNP bp** | **A1** | **A2** | **Freq** | **GWAS association** | | | | **eQTL association** | | | **SMR association** | | | **HEIDI Test** | | **Date** |
| --- | --- | --- | --- | --- | --- | --- | --- | --- | --- | --- | --- | --- | --- | --- | --- | --- | --- | --- | --- | --- | --- | --- |
|  |  |  |  |  |  |  |  |  |  | **b** | **se** | ***p*** | **b** | | **se** | ***p*** | **b** | **se** | ***p*** | ***p*** | **No. of**  **SNPs** |  |
| C12orf47 | cg00049502 | 12 | 112280971 | rs9971746 | 12 | 112337362 | T | C | 0.16501 | 0.09402 | 0.025507 | 0.000227819 | -0.304097 | | 0.0427879 | 1.19E-12 | -0.309178 | 0.094488 | 0.001067383 | 0.1167257 | 17 | ebi-a-GCST012877 |
| SLC25A30 | cg00139037 | 13 | 45992673 | rs9534108 | 13 | 45919515 | G | T | 0.467197 | -0.0629717 | 0.0223476 | 0.00483504 | -0.227459 | | 0.0313472 | 3.98E-13 | 0.276849 | 0.105397 | 0.008621295 | 0.8632562 | 12 | ebi-a-GCST90013866 |
| SLC25A30 | cg00139037 | 13 | 45992673 | rs9534108 | 13 | 45919515 | G | T | 0.467197 | -0.0630187 | 0.0224069 | 0.00487674 | -0.227459 | | 0.0313472 | 3.98E-13 | 0.277055 | 0.105651 | 0.008731955 | 0.8617089 | 12 | ebi-a-GCST90013862 |
| MRPL32 | cg00365680 | 7 | 42972339 | rs76643565 | 7 | 42944461 | A | G | 0.084493 | -0.0867686 | 0.0308333 | 0.00489114 | 0.904966 | | 0.0533835 | 1.85E-64 | -0.0958805 | 0.0345375 | 0.005501044 | 0.2778448 | 20 | ebi-a-GCST012876 |
| TNXB | cg00661399 | 6 | 32049177 | rs2269426 | 6 | 32076499 | A | G | 0.412525 | -0.0614324 | 0.0176676 | 0.000506827 | 0.243375 | | 0.0340562 | 8.92E-13 | -0.252419 | 0.0807312 | 0.001768078 | 0.2185661 | 15 | ebi-a-GCST90018588 |
| C19orf40 | cg00891608 | 19 | 33463040 | rs10414757 | 19 | 33416801 | C | G | 0.236581 | 0.097 | 0.0326 | 0.00295298 | 0.517554 | | 0.0328642 | 7.06E-56 | 0.18742 | 0.064103 | 0.003458622 | 0.4925171 | 18 | finn-b-C3-COLO_EX |
| C19orf40 | cg00891608 | 19 | 33463040 | rs10414757 | 19 | 33416801 | C | G | 0.236581 | 0.1031 | 0.0323 | 0.00139601 | 0.517554 | | 0.0328642 | 7.06E-56 | 0.199206 | 0.063678 | 0.001757975 | 0.5561292 | 18 | finn-b-C3-COLO |
| RAI1 | cg01246520 | 17 | 17644344 | rs12952913 | 17 | 18020050 | T | C | 0.0318091 | 0.00180232 | 0.000606939 | 0.00299999 | 0.533858 | | 0.0700053 | 2.42E-14 | 0.00337603 | 0.00122004 | 0.005655124 | 0.7220321 | 3 | ieu-b-4965 |
| RAI1 | cg01246520 | 17 | 17644344 | rs12952913 | 17 | 18020050 | T | C | 0.0318091 | -0.132115 | 0.0453799 | 0.00359915 | 0.533858 | | 0.0700053 | 2.42E-14 | -0.247472 | 0.0909874 | 0.006531149 | 0.1211903 | 3 | ebi-a-GCST012876 |
| RAI1 | cg01246520 | 17 | 17644344 | rs12952913 | 17 | 18020050 | T | C | 0.0318091 | 0.00180232 | 0.000606939 | 0.00299999 | 0.533858 | | 0.0700053 | 2.42E-14 | 0.00337603 | 0.00122004 | 0.005655124 | 0.7220321 | 3 | ieu-b-4965 |
| ZNF192 | cg01566283 | 6 | 28109239 | rs144447022 | 6 | 29244219 | T | G | 0.0695825 | 0.10297 | 0.033995 | 0.00245471 | -0.437458 | | 0.0502534 | 3.17E-18 | -0.235383 | 0.0822803 | 0.004226516 | 0.7695726 | 12 | ebi-a-GCST012877 |
| TNXB | cg01569346 | 6 | 32064148 | rs6910310 | 6 | 32604221 | G | A | 0.0666004 | 0.00108564 | 0.000398108 | 0.0064 | -0.373024 | | 0.0453224 | 1.87E-16 | -0.00291038 | 0.0011243 | 0.009636437 | 0.1444056 | 20 | ieu-b-4965 |
| TNXB | cg01569346 | 6 | 32064148 | rs6910310 | 6 | 32604221 | G | A | 0.0666004 | 0.00108564 | 0.000398108 | 0.0064 | -0.373024 | | 0.0453224 | 1.87E-16 | -0.00291038 | 0.0011243 | 0.009636437 | 0.1444056 | 20 | ieu-b-4965 |
| ACSF3 | cg02193283 | 16 | 89164953 | rs12917851 | 16 | 89208407 | A | T | 0.136183 | 0.10643 | 0.0269784 | 7.98E-05 | 0.492912 | | 0.0461539 | 1.27E-26 | 0.215921 | 0.0583475 | 0.000215086 | 0.4850999 | 20 | ebi-a-GCST012876 |
| CCHCR1 | cg02247838 | 6 | 31110639 | rs1265087 | 6 | 31109810 | A | G | 0.314115 | 0.0648167 | 0.0237189 | 0.0062816 | -0.576284 | | 0.0336294 | 7.96E-66 | -0.112474 | 0.0416784 | 0.006963078 | 0.4127988 | 20 | ebi-a-GCST90013866 |
| CCHCR1 | cg02247838 | 6 | 31110639 | rs1265087 | 6 | 31109810 | A | G | 0.314115 | 0.0642912 | 0.023425 | 0.00626946 | -0.576284 | | 0.0336294 | 7.96E-66 | -0.111562 | 0.0411664 | 0.00672797 | 0.4172493 | 20 | ebi-a-GCST90013862 |
| CCHCR1 | cg02247838 | 6 | 31110639 | rs1265087 | 6 | 31109810 | A | G | 0.314115 | 0.000884526 | 0.000298469 | 0.00299999 | -0.576284 | | 0.0336294 | 7.96E-66 | -0.00153488 | 0.000525608 | 0.003498103 | 0.4544712 | 20 | ieu-b-4965 |
| CCHCR1 | cg02247838 | 6 | 31110639 | rs1265087 | 6 | 31109810 | A | G | 0.314115 | 0.000884526 | 0.000298469 | 0.00299999 | -0.576284 | | 0.0336294 | 7.96E-66 | -0.00153488 | 0.000525608 | 0.003498103 | 0.4544712 | 20 | ieu-b-4965 |
| COL11A2 | cg02266086 | 6 | 33161336 | rs481139 | 6 | 32576145 | A | C | 0.364811 | 0.0426 | 0.0137 | 0.00193001 | 0.210211 | | 0.0337065 | 4.47E-10 | 0.202654 | 0.0728243 | 0.005389612 | 0.3453335 | 20 | ebi-a-GCST90018808 |
| CCHCR1 | cg02451928 | 6 | 31125948 | rs2524108 | 6 | 31232451 | G | C | 0.252485 | 0.0676114 | 0.0244966 | 0.00604366 | 0.289521 | | 0.0342534 | 2.86E-17 | 0.233528 | 0.0890075 | 0.008698204 | 0.9981961 | 20 | ebi-a-GCST90013862 |
| CCHCR1 | cg02451928 | 6 | 31125948 | rs2524108 | 6 | 31232451 | G | C | 0.252485 | 0.0681732 | 0.0248961 | 0.00617561 | 0.289521 | | 0.0342534 | 2.86E-17 | 0.235469 | 0.0903907 | 0.009187118 | 0.997677 | 20 | ebi-a-GCST90013866 |
| CCHCR1 | cg02451928 | 6 | 31125948 | rs2524108 | 6 | 31232451 | G | C | 0.252485 | 0.096508 | 0.035241 | 0.00617206 | 0.289521 | | 0.0342534 | 2.86E-17 | 0.333337 | 0.127951 | 0.009182355 | 0.1734739 | 20 | ebi-a-GCST012880 |
| MOG | cg02475474 | 6 | 29635158 | rs885945 | 6 | 29696852 | T | C | 0.186879 | 0.1045 | 0.0389 | 0.00723003 | 0.454608 | | 0.0371148 | 1.71E-34 | 0.229868 | 0.087602 | 0.00869018 | 0.4046752 | 20 | finn-b-C3-COLO_EX |
| ME3 | cg02493602 | 11 | 86383696 | rs12098937 | 11 | 86384063 | G | C | 0.128231 | -0.0398 | 0.0142 | 0.00493299 | -0.91219 | | 0.0465696 | 1.97E-85 | 0.0436313 | 0.0157255 | 0.005527707 | 0.7346147 | 20 | ebi-a-GCST90018808 |
| VARS2 | cg02601318 | 6 | 30882384 | rs2596495 | 6 | 31323416 | C | G | 0.0954274 | 0.0974121 | 0.0300395 | 0.0013444 | 0.33604 | | 0.0440886 | 2.50E-14 | 0.289882 | 0.097147 | 0.002845457 | 0.9038601 | 20 | ebi-a-GCST90013862 |
| VARS2 | cg02601318 | 6 | 30882384 | rs2596495 | 6 | 31323416 | C | G | 0.0954274 | 0.0994238 | 0.0310847 | 0.00138153 | 0.33604 | | 0.0440886 | 2.50E-14 | 0.295869 | 0.100318 | 0.00318476 | 0.8803304 | 20 | ebi-a-GCST90013866 |
| ZSCAN12L1 | cg02631126 | 6 | 28058918 | rs144447022 | 6 | 29244219 | T | G | 0.0695825 | 0.10297 | 0.033995 | 0.00245471 | 0.969409 | | 0.0470975 | 3.90E-94 | 0.106219 | 0.0354454 | 0.002729185 | 0.6906841 | 17 | ebi-a-GCST012877 |
| TRIM31 | cg02995061 | 6 | 30076742 | rs385492 | 6 | 29649547 | T | C | 0.489066 | -0.0868 | 0.0285 | 0.00230001 | -0.236865 | | 0.0324134 | 2.72E-13 | 0.366453 | 0.130353 | 0.004935232 | 0.2451241 | 5 | finn-b-C3-COLO |
| TRIM31 | cg02995061 | 6 | 30076742 | rs385492 | 6 | 29649547 | T | C | 0.489066 | -0.0877 | 0.0288 | 0.00232798 | -0.236865 | | 0.0324134 | 2.72E-13 | 0.370253 | 0.131722 | 0.004940967 | 0.3344032 | 5 | finn-b-C3-COLO_EX |
| HCG27 | cg03081173 | 6 | 31166502 | rs2524108 | 6 | 31232451 | G | C | 0.252485 | 0.0681732 | 0.0248961 | 0.00617561 | -0.708158 | | 0.0330603 | 8.66E-102 | -0.0962683 | 0.0354422 | 0.006603529 | 0.5962082 | 20 | ebi-a-GCST90013866 |
| HCG27 | cg03081173 | 6 | 31166502 | rs2524108 | 6 | 31232451 | G | C | 0.252485 | 0.0676114 | 0.0244966 | 0.00604366 | -0.708158 | | 0.0330603 | 8.66E-102 | -0.095475 | 0.034878 | 0.00619267 | 0.6065357 | 20 | ebi-a-GCST90013862 |
| HCG27 | cg03081173 | 6 | 31166502 | rs2524108 | 6 | 31232451 | G | C | 0.252485 | 0.096508 | 0.035241 | 0.00617206 | -0.708158 | | 0.0330603 | 8.66E-102 | -0.13628 | 0.0501694 | 0.006599512 | 0.1080321 | 20 | ebi-a-GCST012880 |
| HCG27 | cg03081173 | 6 | 31166502 | rs2524108 | 6 | 31232451 | G | C | 0.252485 | 0.000816895 | 0.000313223 | 0.00909997 | -0.708158 | | 0.0330603 | 8.66E-102 | -0.00115355 | 0.000445573 | 0.009627995 | 0.695888 | 20 | ieu-b-4965 |
| HCG27 | cg03081173 | 6 | 31166502 | rs2524108 | 6 | 31232451 | G | C | 0.252485 | 0.000816895 | 0.000313223 | 0.00909997 | -0.708158 | | 0.0330603 | 8.66E-102 | -0.00115355 | 0.000445573 | 0.009627995 | 0.695888 | 20 | ieu-b-4965 |
| SCP2 | cg03579872 | 1 | 53393473 | rs12037492 | 1 | 53440816 | T | G | 0.369781 | 0.0706868 | 0.023068 | 0.00218193 | 0.502617 | | 0.0313738 | 9.22E-58 | 0.140638 | 0.0467278 | 0.00261491 | 0.8300593 | 20 | ebi-a-GCST90013866 |
| SCP2 | cg03579872 | 1 | 53393473 | rs12037492 | 1 | 53440816 | T | G | 0.369781 | 0.0701481 | 0.0228154 | 0.00218837 | 0.502617 | | 0.0313738 | 9.22E-58 | 0.139566 | 0.0462216 | 0.002532014 | 0.8418642 | 20 | ebi-a-GCST90013862 |
| SCP2 | cg03579872 | 1 | 53393473 | rs12037492 | 1 | 53440816 | T | G | 0.369781 | 0.000987677 | 0.000291351 | 0.000700003 | 0.502617 | | 0.0313738 | 9.22E-58 | 0.00196507 | 0.000592504 | 0.000911361 | 0.7846096 | 20 | ieu-b-4965 |
| SCP2 | cg03579872 | 1 | 53393473 | rs12037492 | 1 | 53440816 | T | G | 0.369781 | 0.000987677 | 0.000291351 | 0.000700003 | 0.502617 | | 0.0313738 | 9.22E-58 | 0.00196507 | 0.000592504 | 0.000911361 | 0.7846096 | 20 | ieu-b-4965 |
| ADAP1 | cg03810769 | 7 | 951881 | rs1881127 | 7 | 1063104 | C | T | 0.329026 | 0.064424 | 0.0228916 | 0.00503791 | 0.305226 | | 0.0338788 | 2.07E-19 | 0.21107 | 0.0785729 | 0.007224935 | 0.9647993 | 20 | ebi-a-GCST90013862 |
| ADAP1 | cg03810769 | 7 | 951881 | rs1881127 | 7 | 1063104 | C | T | 0.329026 | 0.000892865 | 0.000292158 | 0.00219999 | 0.305226 | | 0.0338788 | 2.07E-19 | 0.00292526 | 0.00101076 | 0.003802142 | 0.8024606 | 20 | ieu-b-4965 |
| ADAP1 | cg03810769 | 7 | 951881 | rs1881127 | 7 | 1063104 | C | T | 0.329026 | 0.0648893 | 0.0231333 | 0.00503141 | 0.305226 | | 0.0338788 | 2.07E-19 | 0.212594 | 0.0793792 | 0.007401644 | 0.9615963 | 20 | ebi-a-GCST90013866 |
| ADAP1 | cg03810769 | 7 | 951881 | rs1881127 | 7 | 1063104 | C | T | 0.329026 | 0.0362 | 0.0128 | 0.00482503 | 0.305226 | | 0.0338788 | 2.07E-19 | 0.118601 | 0.0439538 | 0.006969393 | 0.4442876 | 20 | ebi-a-GCST90018808 |
| ADAP1 | cg03810769 | 7 | 951881 | rs1881127 | 7 | 1063104 | C | T | 0.329026 | 0.000892865 | 0.000292158 | 0.00219999 | 0.305226 | | 0.0338788 | 2.07E-19 | 0.00292526 | 0.00101076 | 0.003802142 | 0.8024606 | 20 | ieu-b-4965 |
| ACSF3 | cg04308346 | 16 | 89203063 | rs34022105 | 16 | 89229598 | A | G | 0.136183 | 0.112254 | 0.0267811 | 2.77E-05 | -0.345108 | | 0.0453454 | 2.73E-14 | -0.325272 | 0.088593 | 0.000241099 | 0.805441 | 20 | ebi-a-GCST012876 |
| HLA-DRB1 | cg04601775 | 6 | 32557478 | rs139600543 | 6 | 32227839 | T | A | 0.236581 | 0.0011021 | 0.00032728 | 0.000759994 | 0.658758 | | 0.0363193 | 1.60E-73 | 0.001673 | 0.000505303 | 0.000930046 | 0.377624 | 20 | ieu-b-4965 |
| HLA-DRB1 | cg04601775 | 6 | 32557478 | rs139600543 | 6 | 32227839 | T | A | 0.236581 | 0.075473 | 0.0259182 | 0.00359162 | 0.658758 | | 0.0363193 | 1.60E-73 | 0.114569 | 0.0398479 | 0.004038336 | 0.4105872 | 20 | ebi-a-GCST90013866 |
| HLA-DRB1 | cg04601775 | 6 | 32557478 | rs139600543 | 6 | 32227839 | T | A | 0.236581 | 0.0011021 | 0.00032728 | 0.000759994 | 0.658758 | | 0.0363193 | 1.60E-73 | 0.001673 | 0.000505303 | 0.000930046 | 0.377624 | 20 | ieu-b-4965 |
| HLA-DRB1 | cg04601775 | 6 | 32557478 | rs139600543 | 6 | 32227839 | T | A | 0.236581 | 0.0746456 | 0.0253985 | 0.00350711 | 0.658758 | | 0.0363193 | 1.60E-73 | 0.113313 | 0.039058 | 0.003718096 | 0.4226295 | 20 | ebi-a-GCST90013862 |
| UBE2I | cg04781839 | 16 | 1374937 | rs761063 | 16 | 1376386 | G | C | 0.279324 | 0.0009024 | 0.000303671 | 0.00299999 | -0.680624 | | 0.0332231 | 2.84E-93 | -0.00132584 | 0.000450835 | 0.00327303 | 0.3669317 | 20 | ieu-b-4965 |
| UBE2I | cg04781839 | 16 | 1374937 | rs761063 | 16 | 1376386 | G | C | 0.279324 | 0.0009024 | 0.000303671 | 0.00299999 | -0.680624 | | 0.0332231 | 2.84E-93 | -0.00132584 | 0.000450835 | 0.00327303 | 0.3669317 | 20 | ieu-b-4965 |
| LOC285830 | cg05119316 | 6 | 29716135 | rs385492 | 6 | 29649547 | T | C | 0.489066 | -0.00086315 | 0.000280319 | 0.0021 | 0.349717 | | 0.0318427 | 4.63E-28 | -0.00246814 | 0.000832467 | 0.003028325 | 0.7647932 | 9 | ieu-b-4965 |
| LOC285830 | cg05119316 | 6 | 29716135 | rs385492 | 6 | 29649547 | T | C | 0.489066 | -0.0877 | 0.0288 | 0.00232798 | 0.349717 | | 0.0318427 | 4.63E-28 | -0.250774 | 0.0854592 | 0.003341595 | 0.7632298 | 8 | finn-b-C3-COLO_EX |
| LOC285830 | cg05119316 | 6 | 29716135 | rs385492 | 6 | 29649547 | T | C | 0.489066 | -0.0868 | 0.0285 | 0.00230001 | 0.349717 | | 0.0318427 | 4.63E-28 | -0.248201 | 0.08457 | 0.00333702 | 0.7187773 | 8 | finn-b-C3-COLO |
| LOC285830 | cg05119316 | 6 | 29716135 | rs385492 | 6 | 29649547 | T | C | 0.489066 | -0.00086315 | 0.000280319 | 0.0021 | 0.349717 | | 0.0318427 | 4.63E-28 | -0.00246814 | 0.000832467 | 0.003028325 | 0.7647932 | 9 | ieu-b-4965 |
| MOG | cg05279622 | 6 | 29629758 | rs2535238 | 6 | 29645038 | A | C | 0.188867 | 0.0704586 | 0.0257318 | 0.00617775 | 1.13938 | | 0.0299663 | 0 | 0.0618394 | 0.0226425 | 0.006311975 | 0.6882566 | 20 | ebi-a-GCST90013866 |
| MOG | cg05279622 | 6 | 29629758 | rs2535238 | 6 | 29645038 | A | C | 0.188867 | 0.0697568 | 0.0252599 | 0.00605494 | 1.13938 | | 0.0299663 | 0 | 0.0612235 | 0.0222283 | 0.005881646 | 0.6876988 | 20 | ebi-a-GCST90013862 |
| MTCH1 | cg05325229 | 6 | 36948816 | rs3778021 | 6 | 36948805 | G | A | 0.366799 | 0.000960919 | 0.000299508 | 0.00129999 | 0.223375 | | 0.0341913 | 6.44E-11 | 0.00430182 | 0.00149379 | 0.003979206 | 0.1848376 | 4 | ieu-b-4965 |
| MTCH1 | cg05325229 | 6 | 36948816 | rs3778021 | 6 | 36948805 | G | A | 0.366799 | 0.058861 | 0.020061 | 0.00334503 | 0.223375 | | 0.0341913 | 6.44E-11 | 0.263508 | 0.0984502 | 0.007438291 | 0.3207206 | 4 | ebi-a-GCST012877 |
| MTCH1 | cg05325229 | 6 | 36948816 | rs3778021 | 6 | 36948805 | G | A | 0.366799 | 0.000960919 | 0.000299508 | 0.00129999 | 0.223375 | | 0.0341913 | 6.44E-11 | 0.00430182 | 0.00149379 | 0.003979206 | 0.1848376 | 4 | ieu-b-4965 |
| TNXB | cg06002203 | 6 | 31976720 | rs497309 | 6 | 31892484 | C | A | 0.0735586 | 0.00109266 | 0.000414708 | 0.00840001 | -0.677961 | | 0.0444025 | 1.24E-52 | -0.00161169 | 0.00062074 | 0.009420748 | 0.5864905 | 20 | ieu-b-4965 |
| TNXB | cg06002203 | 6 | 31976720 | rs497309 | 6 | 31892484 | C | A | 0.0735586 | 0.00109266 | 0.000414708 | 0.00840001 | -0.677961 | | 0.0444025 | 1.24E-52 | -0.00161169 | 0.00062074 | 0.009420748 | 0.5864905 | 20 | ieu-b-4965 |
| HLA-DQA2 | cg06598146 | 6 | 32711632 | rs28366356 | 6 | 32565091 | T | G | 0.368787 | 0.0447 | 0.0127 | 0.000405005 | 0.218194 | | 0.0329035 | 3.33E-11 | 0.204864 | 0.0658956 | 0.001877771 | 0.2220193 | 20 | ebi-a-GCST90018808 |
| ARPC2 | cg06608945 | 2 | 219082296 | rs10209152 | 2 | 219072675 | G | A | 0.480119 | -0.0559714 | 0.0169826 | 0.000981409 | -0.493434 | | 0.0304465 | 4.53E-59 | 0.113432 | 0.0351216 | 0.001239197 | 0.3917225 | 20 | ebi-a-GCST012879 |
| ARPC2 | cg06608945 | 2 | 219082296 | rs10209152 | 2 | 219072675 | G | A | 0.480119 | -0.0550896 | 0.0180602 | 0.00228597 | -0.493434 | | 0.0304465 | 4.53E-59 | 0.111645 | 0.0372437 | 0.002720281 | 0.2776198 | 20 | ebi-a-GCST012876 |
| BAT2 | cg06833981 | 6 | 31597708 | rs1265049 | 6 | 31081080 | C | T | 0.026839 | 0.19826 | 0.057846 | 0.000609439 | -1.58748 | | 0.0932742 | 5.89E-65 | -0.12489 | 0.0371704 | 0.000779637 | 0.381213 | 20 | ebi-a-GCST012877 |
| HLA-DQB2 | cg07180897 | 6 | 32729130 | rs9274623 | 6 | 32635998 | T | G | 0.202783 | 0.11358 | 0.042824 | 0.00799264 | -1.34175 | | 0.0231749 | 0 | -0.0846506 | 0.03195 | 0.008061754 | 0.2032952 | 20 | ebi-a-GCST012880 |
| MSRB2 | cg07355157 | 10 | 23385780 | rs61849888 | 10 | 23376982 | C | A | 0.254473 | 0.000912787 | 0.000331176 | 0.00580003 | 0.879692 | | 0.0339445 | 4.45E-148 | 0.00103762 | 0.000378591 | 0.006130053 | 0.2300959 | 20 | ieu-b-4965 |
| MSRB2 | cg07355157 | 10 | 23385780 | rs61849888 | 10 | 23376982 | C | A | 0.254473 | 0.000912787 | 0.000331176 | 0.00580003 | 0.879692 | | 0.0339445 | 4.45E-148 | 0.00103762 | 0.000378591 | 0.006130053 | 0.2300959 | 20 | ieu-b-4965 |
| MSRB2 | cg07355157 | 10 | 23385780 | rs61849888 | 10 | 23376982 | C | A | 0.254473 | 0.0419 | 0.0143 | 0.00330499 | 0.879692 | | 0.0339445 | 4.45E-148 | 0.0476303 | 0.0163593 | 0.003596749 | 0.1915998 | 20 | ebi-a-GCST90018808 |
| KIF1A | cg07652786 | 2 | 241726053 | rs4676367 | 2 | 241722761 | G | A | 0.486083 | -0.0719 | 0.0267 | 0.00701698 | 0.41793 | | 0.031998 | 5.49E-39 | -0.172038 | 0.06523 | 0.008354148 | 0.5884669 | 20 | finn-b-C3-COLO |
| KIF1A | cg07652786 | 2 | 241726053 | rs4676367 | 2 | 241722761 | G | A | 0.486083 | -0.0762 | 0.0269 | 0.00469699 | 0.41793 | | 0.031998 | 5.49E-39 | -0.182327 | 0.0658612 | 0.005634029 | 0.5934175 | 20 | finn-b-C3-COLO_EX |
| PPT2 | cg08057899 | 6 | 32122460 | rs192471087 | 6 | 32119730 | C | G | 0.199801 | 0.0678 | 0.0229 | 0.00306803 | -0.403816 | | 0.0370846 | 1.30E-27 | -0.167898 | 0.0587678 | 0.004276976 | 0.1405199 | 20 | ebi-a-GCST90018808 |
| PPT2 | cg08057899 | 6 | 32122460 | rs192471087 | 6 | 32119730 | C | G | 0.199801 | 0.000898556 | 0.000329856 | 0.0064 | -0.403816 | | 0.0370846 | 1.30E-27 | -0.00222516 | 0.00084202 | 0.00822607 | 0.8683021 | 20 | ieu-b-4965 |
| PPT2 | cg08057899 | 6 | 32122460 | rs192471087 | 6 | 32119730 | C | G | 0.199801 | 0.000898556 | 0.000329856 | 0.0064 | -0.403816 | | 0.0370846 | 1.30E-27 | -0.00222516 | 0.00084202 | 0.00822607 | 0.8683021 | 20 | ieu-b-4965 |
| CYB5R3 | cg08690876 | 22 | 43040721 | rs1109585 | 22 | 43040921 | C | T | 0.49006 | -0.0535284 | 0.019133 | 0.00514683 | -0.617065 | | 0.0309365 | 1.62E-88 | 0.0867468 | 0.03131 | 0.005595665 | 0.2149444 | 20 | ebi-a-GCST90018588 |
| CYB5R3 | cg08690876 | 22 | 43040721 | rs1109585 | 22 | 43040921 | C | T | 0.49006 | -0.0355 | 0.0131 | 0.00667806 | -0.617065 | | 0.0309365 | 1.62E-88 | 0.0575304 | 0.0214246 | 0.007247467 | 0.1688887 | 20 | ebi-a-GCST90018808 |
| AXIN1 | cg08823186 | 16 | 347294 | rs214251 | 16 | 347326 | A | G | 0.213718 | -0.0625 | 0.0166 | 0.000167402 | 0.614356 | | 0.0386551 | 7.06E-57 | -0.101733 | 0.027768 | 0.000248636 | 0.1169413 | 20 | ebi-a-GCST90018808 |
| ZNF389 | cg08968635 | 6 | 28129556 | rs148696809 | 6 | 28934352 | C | T | 0.0695825 | 0.10088 | 0.033916 | 0.00293461 | 0.537883 | | 0.0505025 | 1.73E-26 | 0.18755 | 0.0654673 | 0.004172874 | 0.7607061 | 7 | ebi-a-GCST012877 |
| ECHDC2 | cg09128567 | 1 | 53387203 | rs11206056 | 1 | 53444836 | C | T | 0.369781 | 0.0717063 | 0.0228691 | 0.00178501 | 0.329292 | | 0.0316935 | 2.76E-25 | 0.217759 | 0.0725429 | 0.002683922 | 0.8327369 | 20 | ebi-a-GCST90013862 |
| ECHDC2 | cg09128567 | 1 | 53387203 | rs11206056 | 1 | 53444836 | C | T | 0.369781 | 0.0722672 | 0.0231272 | 0.00177947 | 0.329292 | | 0.0316935 | 2.76E-25 | 0.219462 | 0.0733407 | 0.00276822 | 0.8233085 | 20 | ebi-a-GCST90013866 |
| ECHDC2 | cg09128567 | 1 | 53387203 | rs11206056 | 1 | 53444836 | C | T | 0.369781 | 0.00101464 | 0.000292102 | 0.00051 | 0.329292 | | 0.0316935 | 2.76E-25 | 0.00308128 | 0.000935322 | 0.0009865 | 0.8452452 | 20 | ieu-b-4965 |
| ECHDC2 | cg09128567 | 1 | 53387203 | rs11206056 | 1 | 53444836 | C | T | 0.369781 | 0.00101464 | 0.000292102 | 0.00051 | 0.329292 | | 0.0316935 | 2.76E-25 | 0.00308128 | 0.000935322 | 0.0009865 | 0.8452452 | 20 | ieu-b-4965 |
| TAP2 | cg09408973 | 6 | 32796660 | rs241439 | 6 | 32797537 | G | T | 0.388668 | 0.000825648 | 0.000285948 | 0.00389996 | 0.312631 | | 0.0330538 | 3.13E-21 | 0.00264097 | 0.000956321 | 0.005752092 | 0.1268788 | 4 | ieu-b-4965 |
| TAP2 | cg09408973 | 6 | 32796660 | rs241439 | 6 | 32797537 | G | T | 0.388668 | 0.000825648 | 0.000285948 | 0.00389996 | 0.312631 | | 0.0330538 | 3.13E-21 | 0.00264097 | 0.000956321 | 0.005752092 | 0.1268788 | 4 | ieu-b-4965 |
| LTA | cg09621572 | 6 | 31539973 | rs1041981 | 6 | 31540784 | A | C | 0.306163 | 0.05629 | 0.020541 | 0.0061372 | -0.384563 | | 0.0328422 | 1.14E-31 | -0.146374 | 0.0548571 | 0.007624176 | 0.1082532 | 20 | ebi-a-GCST012877 |
| LTA | cg09736959 | 6 | 31540114 | rs3135041 | 6 | 31555657 | G | A | 0.27833 | 0.07056 | 0.020928 | 0.000747446 | 0.275514 | | 0.0340066 | 5.42E-16 | 0.256103 | 0.0822748 | 0.00185335 | 0.2096045 | 20 | ebi-a-GCST012877 |
| LTA | cg09736959 | 6 | 31540114 | rs3135041 | 6 | 31555657 | G | A | 0.27833 | 0.0540141 | 0.0195587 | 0.00575122 | 0.275514 | | 0.0340066 | 5.42E-16 | 0.196048 | 0.0750008 | 0.008950129 | 0.4285317 | 20 | ebi-a-GCST012876 |
| LTA | cg09736959 | 6 | 31540114 | rs3135041 | 6 | 31555657 | G | A | 0.27833 | 0.0626396 | 0.0186073 | 0.00076157 | 0.275514 | | 0.0340066 | 5.42E-16 | 0.227355 | 0.0731348 | 0.001879021 | 0.4494821 | 20 | ebi-a-GCST012879 |
| LOC285830 | cg09990446 | 6 | 29716108 | rs385492 | 6 | 29649547 | T | C | 0.489066 | -0.00086315 | 0.000280319 | 0.0021 | 0.374969 | | 0.0325383 | 9.99E-31 | -0.00230192 | 0.000773806 | 0.002931717 | 0.7594324 | 11 | ieu-b-4965 |
| LOC285830 | cg09990446 | 6 | 29716108 | rs385492 | 6 | 29649547 | T | C | 0.489066 | -0.00086315 | 0.000280319 | 0.0021 | 0.374969 | | 0.0325383 | 9.99E-31 | -0.00230192 | 0.000773806 | 0.002931717 | 0.7594324 | 11 | ieu-b-4965 |
| LOC285830 | cg09990446 | 6 | 29716108 | rs385492 | 6 | 29649547 | T | C | 0.489066 | -0.0868 | 0.0285 | 0.00230001 | 0.374969 | | 0.0325383 | 9.99E-31 | -0.231486 | 0.0786159 | 0.003234596 | 0.4736503 | 9 | finn-b-C3-COLO |
| LOC285830 | cg09990446 | 6 | 29716108 | rs385492 | 6 | 29649547 | T | C | 0.489066 | -0.0877 | 0.0288 | 0.00232798 | 0.374969 | | 0.0325383 | 9.99E-31 | -0.233886 | 0.0794426 | 0.003239087 | 0.5080302 | 9 | finn-b-C3-COLO_EX |
| HCG27 | cg10473907 | 6 | 31167664 | rs1265098 | 6 | 31106177 | C | T | 0.269384 | -0.00105857 | 0.000329932 | 0.00129999 | 0.426812 | | 0.037466 | 4.58E-30 | -0.00248018 | 0.000803088 | 0.002013042 | 0.3374032 | 20 | ieu-b-4965 |
| HCG27 | cg10473907 | 6 | 31167664 | rs1265098 | 6 | 31106177 | C | T | 0.269384 | -0.00105857 | 0.000329932 | 0.00129999 | 0.426812 | | 0.037466 | 4.58E-30 | -0.00248018 | 0.000803088 | 0.002013042 | 0.3374032 | 20 | ieu-b-4965 |
| SCD | cg10507965 | 10 | 102107251 | rs145347289 | 10 | 101518099 | T | C | 0.0347913 | 0.00178626 | 0.000617042 | 0.00379997 | 0.429102 | | 0.0705383 | 1.18E-09 | 0.00416279 | 0.0015925 | 0.008949235 | 0.1099615 | 3 | ieu-b-4965 |
| SCD | cg10507965 | 10 | 102107251 | rs145347289 | 10 | 101518099 | T | C | 0.0347913 | 0.00178626 | 0.000617042 | 0.00379997 | 0.429102 | | 0.0705383 | 1.18E-09 | 0.00416279 | 0.0015925 | 0.008949235 | 0.1099615 | 3 | ieu-b-4965 |
| VARS2 | cg10661769 | 6 | 30881484 | rs2233956 | 6 | 31081205 | C | T | 0.152087 | 0.001026 | 0.000338083 | 0.00239999 | 0.225469 | | 0.0386473 | 5.41E-09 | 0.00455051 | 0.0016902 | 0.007096391 | 0.9475321 | 20 | ieu-b-4965 |
| VARS2 | cg10661769 | 6 | 30881484 | rs2233956 | 6 | 31081205 | C | T | 0.152087 | 0.074985 | 0.025227 | 0.00295502 | 0.225469 | | 0.0386473 | 5.41E-09 | 0.332573 | 0.125572 | 0.008085729 | 0.4104916 | 20 | ebi-a-GCST012877 |
| VARS2 | cg10661769 | 6 | 30881484 | rs2233956 | 6 | 31081205 | C | T | 0.152087 | 0.001026 | 0.000338083 | 0.00239999 | 0.225469 | | 0.0386473 | 5.41E-09 | 0.00455051 | 0.0016902 | 0.007096391 | 0.9475321 | 20 | ieu-b-4965 |
| VARS2 | cg10661769 | 6 | 30881484 | rs2233956 | 6 | 31081205 | C | T | 0.152087 | 0.0873214 | 0.0260496 | 0.000885809 | 0.225469 | | 0.0386473 | 5.41E-09 | 0.387288 | 0.133249 | 0.003654967 | 0.9496107 | 20 | ebi-a-GCST90013862 |
| VARS2 | cg10661769 | 6 | 30881484 | rs2233956 | 6 | 31081205 | C | T | 0.152087 | 0.0885618 | 0.0267077 | 0.000913272 | 0.225469 | | 0.0386473 | 5.41E-09 | 0.392789 | 0.136251 | 0.00394111 | 0.9454314 | 20 | ebi-a-GCST90013866 |
| HLA-B | cg11187245 | 6 | 31323397 | rs1265094 | 6 | 31106893 | A | G | 0.468191 | -0.0718601 | 0.0222017 | 0.00120926 | 0.298379 | | 0.0328948 | 1.18E-19 | -0.240835 | 0.0790029 | 0.002300388 | 0.1150675 | 20 | ebi-a-GCST90013866 |
| HLA-B | cg11187245 | 6 | 31323397 | rs1265094 | 6 | 31106893 | A | G | 0.468191 | -0.0717754 | 0.0221901 | 0.00122073 | 0.298379 | | 0.0328948 | 1.18E-19 | -0.240551 | 0.0789558 | 0.002314019 | 0.1165283 | 20 | ebi-a-GCST90013862 |
| LIPT2 | cg11417029 | 11 | 74204987 | rs7121457 | 11 | 74205004 | G | A | 0.393638 | -0.0559 | 0.0128 | 1.27E-05 | -0.247385 | | 0.0319643 | 9.99E-15 | 0.225964 | 0.0594103 | 0.000142701 | 0.1231566 | 5 | ebi-a-GCST90018808 |
| LIPT2 | cg11417029 | 11 | 74204987 | rs7121457 | 11 | 74205004 | G | A | 0.393638 | -0.0745 | 0.027 | 0.00583996 | -0.247385 | | 0.0319643 | 9.99E-15 | 0.30115 | 0.115871 | 0.009348968 | 0.3678746 | 4 | finn-b-C3-COLO |
| CDSN | cg11468993 | 6 | 31082939 | rs3094216 | 6 | 31084048 | G | A | 0.187873 | 0.0887044 | 0.0242662 | 0.000283472 | -0.858996 | | 0.0316824 | 6.97E-162 | -0.103265 | 0.0285051 | 0.000291551 | 0.9486462 | 20 | ebi-a-GCST90013862 |
| CDSN | cg11468993 | 6 | 31082939 | rs3094216 | 6 | 31084048 | G | A | 0.187873 | 0.08046 | 0.02357 | 0.000640855 | -0.858996 | | 0.0316824 | 6.97E-162 | -0.0936675 | 0.0276556 | 0.000706816 | 0.6161609 | 20 | ebi-a-GCST012877 |
| CDSN | cg11468993 | 6 | 31082939 | rs3094216 | 6 | 31084048 | G | A | 0.187873 | 0.0896886 | 0.0247808 | 0.00029542 | -0.858996 | | 0.0316824 | 6.97E-162 | -0.104411 | 0.0291045 | 0.000333921 | 0.9414397 | 20 | ebi-a-GCST90013866 |
| C7orf25 | cg11745092 | 7 | 42951893 | rs583301 | 7 | 42947886 | C | A | 0.129225 | -0.0959969 | 0.0266883 | 0.000321951 | 0.71247 | | 0.0452148 | 6.10E-56 | -0.134738 | 0.0384224 | 0.000453596 | 0.4168772 | 20 | ebi-a-GCST012876 |
| MRPL28 | cg12437481 | 16 | 420112 | rs17136392 | 16 | 421912 | A | G | 0.0914513 | -0.00116828 | 0.0004505 | 0.00949992 | -1.77951 | | 0.0361617 | 0 | 0.000656518 | 0.000253511 | 0.009605882 | 0.8521767 | 20 | ieu-b-4965 |
| MRPL28 | cg12437481 | 16 | 420112 | rs17136392 | 16 | 421912 | A | G | 0.0914513 | -0.00116828 | 0.0004505 | 0.00949992 | -1.77951 | | 0.0361617 | 0 | 0.000656518 | 0.000253511 | 0.009605882 | 0.8521767 | 20 | ieu-b-4965 |
| ZSCAN12L1 | cg12740337 | 6 | 28058973 | rs144447022 | 6 | 29244219 | T | G | 0.0695825 | 0.10297 | 0.033995 | 0.00245471 | 1.02178 | | 0.0465584 | 9.41E-107 | 0.100775 | 0.0335858 | 0.002695092 | 0.7066451 | 17 | ebi-a-GCST012877 |
| ZNF389 | cg12963246 | 6 | 28129442 | rs144447022 | 6 | 29244219 | T | G | 0.0695825 | 0.10297 | 0.033995 | 0.00245471 | 0.811233 | | 0.0485355 | 1.03E-62 | 0.12693 | 0.0425879 | 0.002878442 | 0.8514315 | 16 | ebi-a-GCST012877 |
| HLA-DRA | cg13022993 | 6 | 32409856 | rs36096565 | 6 | 32560025 | G | A | 0.181909 | 0.0708386 | 0.0224363 | 0.00159225 | 0.343054 | | 0.046796 | 2.29E-13 | 0.206494 | 0.0712096 | 0.003733926 | 0.1586943 | 20 | ebi-a-GCST012879 |
| TNXB | cg13199127 | 6 | 32049196 | rs2269426 | 6 | 32076499 | A | G | 0.412525 | -0.0614324 | 0.0176676 | 0.000506827 | 0.283116 | | 0.0339205 | 7.03E-17 | -0.216987 | 0.0676028 | 0.0013286 | 0.1932178 | 20 | ebi-a-GCST90018588 |
| HCG27 | cg13212186 | 6 | 31166504 | rs2524108 | 6 | 31232451 | G | C | 0.252485 | 0.096508 | 0.035241 | 0.00617206 | -0.676326 | | 0.0331225 | 1.13E-92 | -0.142695 | 0.0525731 | 0.006643343 | 0.1001256 | 20 | ebi-a-GCST012880 |
| HCG27 | cg13212186 | 6 | 31166504 | rs2524108 | 6 | 31232451 | G | C | 0.252485 | 0.000816895 | 0.000313223 | 0.00909997 | -0.676326 | | 0.0331225 | 1.13E-92 | -0.00120784 | 0.000466887 | 0.009681187 | 0.6791395 | 20 | ieu-b-4965 |
| HCG27 | cg13212186 | 6 | 31166504 | rs2524108 | 6 | 31232451 | G | C | 0.252485 | 0.0681732 | 0.0248961 | 0.00617561 | -0.676326 | | 0.0331225 | 1.13E-92 | -0.100799 | 0.0371403 | 0.006647374 | 0.5787903 | 20 | ebi-a-GCST90013866 |
| HCG27 | cg13212186 | 6 | 31166504 | rs2524108 | 6 | 31232451 | G | C | 0.252485 | 0.000816895 | 0.000313223 | 0.00909997 | -0.676326 | | 0.0331225 | 1.13E-92 | -0.00120784 | 0.000466887 | 0.009681187 | 0.6791395 | 20 | ieu-b-4965 |
| HCG27 | cg13212186 | 6 | 31166504 | rs2524108 | 6 | 31232451 | G | C | 0.252485 | 0.0676114 | 0.0244966 | 0.00604366 | -0.676326 | | 0.0331225 | 1.13E-92 | -0.0999687 | 0.0365495 | 0.006235032 | 0.587196 | 20 | ebi-a-GCST90013862 |
| DDR1 | cg13396738 | 6 | 30850718 | rs3131006 | 6 | 31098134 | T | A | 0.16004 | 0.000954892 | 0.000321912 | 0.00299999 | 0.24332 | | 0.0356978 | 9.35E-12 | 0.00392443 | 0.00144285 | 0.006529954 | 0.7124933 | 20 | ieu-b-4965 |
| DDR1 | cg13396738 | 6 | 30850718 | rs3131006 | 6 | 31098134 | T | A | 0.16004 | 0.093618 | 0.0248058 | 0.00018098 | 0.24332 | | 0.0356978 | 9.35E-12 | 0.384753 | 0.116531 | 0.000960979 | 0.4027677 | 20 | ebi-a-GCST90013862 |
| DDR1 | cg13396738 | 6 | 30850718 | rs3131006 | 6 | 31098134 | T | A | 0.16004 | 0.000954892 | 0.000321912 | 0.00299999 | 0.24332 | | 0.0356978 | 9.35E-12 | 0.00392443 | 0.00144285 | 0.006529954 | 0.7124933 | 20 | ieu-b-4965 |
| DDR1 | cg13396738 | 6 | 30850718 | rs3131006 | 6 | 31098134 | T | A | 0.16004 | 0.07067 | 0.024502 | 0.00392311 | 0.24332 | | 0.0356978 | 9.35E-12 | 0.290441 | 0.109343 | 0.007901927 | 0.2191154 | 20 | ebi-a-GCST012877 |
| DDR1 | cg13396738 | 6 | 30850718 | rs3131006 | 6 | 31098134 | T | A | 0.16004 | 0.0948355 | 0.0254025 | 0.000188973 | 0.24332 | | 0.0356978 | 9.35E-12 | 0.389756 | 0.119034 | 0.0010591 | 0.3962114 | 20 | ebi-a-GCST90013866 |
| HCG27 | cg13502540 | 6 | 31166512 | rs2524108 | 6 | 31232451 | G | C | 0.252485 | 0.096508 | 0.035241 | 0.00617206 | -0.710066 | | 0.0330259 | 1.55E-102 | -0.135914 | 0.0500316 | 0.006596276 | 0.1131047 | 20 | ebi-a-GCST012880 |
| HCG27 | cg13502540 | 6 | 31166512 | rs2524108 | 6 | 31232451 | G | C | 0.252485 | 0.0676114 | 0.0244966 | 0.00604366 | -0.710066 | | 0.0330259 | 1.55E-102 | -0.0952185 | 0.0347821 | 0.006189542 | 0.7634304 | 20 | ebi-a-GCST90013862 |
| HCG27 | cg13502540 | 6 | 31166512 | rs2524108 | 6 | 31232451 | G | C | 0.252485 | 0.0681732 | 0.0248961 | 0.00617561 | -0.710066 | | 0.0330259 | 1.55E-102 | -0.0960097 | 0.0353449 | 0.006600291 | 0.7550686 | 20 | ebi-a-GCST90013866 |
| HCG27 | cg13502540 | 6 | 31166512 | rs2524108 | 6 | 31232451 | G | C | 0.252485 | 0.000816895 | 0.000313223 | 0.00909997 | -0.710066 | | 0.0330259 | 1.55E-102 | -0.00115045 | 0.000444352 | 0.009624065 | 0.6563408 | 20 | ieu-b-4965 |
| HCG27 | cg13502540 | 6 | 31166512 | rs2524108 | 6 | 31232451 | G | C | 0.252485 | 0.000816895 | 0.000313223 | 0.00909997 | -0.710066 | | 0.0330259 | 1.55E-102 | -0.00115045 | 0.000444352 | 0.009624065 | 0.6563408 | 20 | ieu-b-4965 |
| HCG22 | cg13525397 | 6 | 31025933 | rs1265049 | 6 | 31081080 | C | T | 0.026839 | 0.19826 | 0.057846 | 0.000609439 | 0.961603 | | 0.101309 | 2.27E-21 | 0.206177 | 0.0639574 | 0.00126565 | 0.2977844 | 8 | ebi-a-GCST012877 |
| HCG22 | cg13525397 | 6 | 31025933 | rs1265049 | 6 | 31081080 | C | T | 0.026839 | 0.14886 | 0.0500169 | 0.00291837 | 0.961603 | | 0.101309 | 2.27E-21 | 0.154804 | 0.0545111 | 0.004513267 | 0.9414896 | 8 | ebi-a-GCST012876 |
| ACADS | cg13914990 | 12 | 121174878 | rs1814851 | 12 | 121128633 | G | C | 0.469185 | 0.0461857 | 0.0170439 | 0.00673209 | 0.677569 | | 0.0304421 | 9.51E-110 | 0.0681638 | 0.0253402 | 0.007146362 | 0.3103598 | 20 | ebi-a-GCST012879 |
| CXCR2 | cg14150666 | 2 | 218991310 | rs4674260 | 2 | 219002376 | T | C | 0.493042 | 0.0727837 | 0.0180612 | 5.58E-05 | -0.885382 | | 0.0274768 | 8.44E-228 | -0.082206 | 0.0205582 | 6.37E-05 | 0.9955378 | 20 | ebi-a-GCST012876 |
| BTNL2 | cg14241129 | 6 | 32367729 | rs28383457 | 6 | 32609456 | T | C | 0.302187 | 0.035 | 0.013 | 0.00710608 | 0.364127 | | 0.0335224 | 1.75E-27 | 0.0961203 | 0.0367822 | 0.008969031 | 0.2611117 | 20 | ebi-a-GCST90018808 |
| CCDC88B | cg14288399 | 11 | 64110785 | rs2276014 | 11 | 64081445 | A | G | 0.161034 | -0.075957 | 0.0236121 | 0.00129599 | 0.443688 | | 0.0418748 | 3.12E-26 | -0.171195 | 0.0556164 | 0.00208304 | 0.1601478 | 20 | ebi-a-GCST012879 |
| LOC285830 | cg14298020 | 6 | 29712462 | rs385492 | 6 | 29649547 | T | C | 0.489066 | -0.0868 | 0.0285 | 0.00230001 | -0.257865 | | 0.0322047 | 1.17E-15 | 0.33661 | 0.118248 | 0.004418276 | 0.797572 | 5 | finn-b-C3-COLO |
| LOC285830 | cg14298020 | 6 | 29712462 | rs385492 | 6 | 29649547 | T | C | 0.489066 | -0.00086315 | 0.000280319 | 0.0021 | -0.257865 | | 0.0322047 | 1.17E-15 | 0.00334729 | 0.00116469 | 0.004053262 | 0.4067583 | 9 | ieu-b-4965 |
| LOC285830 | cg14298020 | 6 | 29712462 | rs385492 | 6 | 29649547 | T | C | 0.489066 | -0.00086315 | 0.000280319 | 0.0021 | -0.257865 | | 0.0322047 | 1.17E-15 | 0.00334729 | 0.00116469 | 0.004053262 | 0.4067583 | 9 | ieu-b-4965 |
| LOC285830 | cg14298020 | 6 | 29712462 | rs385492 | 6 | 29649547 | T | C | 0.489066 | -0.0877 | 0.0288 | 0.00232798 | -0.257865 | | 0.0322047 | 1.17E-15 | 0.3401 | 0.11949 | 0.004423663 | 0.7146933 | 5 | finn-b-C3-COLO_EX |
| LTA | cg14437551 | 6 | 31539986 | rs1041981 | 6 | 31540784 | A | C | 0.306163 | 0.05629 | 0.020541 | 0.0061372 | -0.377476 | | 0.0329018 | 1.81E-30 | -0.149122 | 0.0559475 | 0.007689844 | 0.1558954 | 20 | ebi-a-GCST012877 |
| PLEKHG4B | cg14640772 | 5 | 188704 | rs62330020 | 5 | 311880 | T | C | 0.0407555 | 0.2755 | 0.098 | 0.00492697 | 0.510922 | | 0.0744181 | 6.62E-12 | 0.539221 | 0.207267 | 0.009279636 | 0.1310498 | 20 | finn-b-C3-COLO_EX |
| GIPR | cg14661225 | 19 | 46171451 | rs4399645 | 19 | 46166073 | T | C | 0.393638 | 0.000874776 | 0.000286604 | 0.00230001 | 0.342576 | | 0.0319916 | 9.31E-27 | 0.00255352 | 0.000869936 | 0.003332227 | 0.5842539 | 15 | ieu-b-4965 |
| GIPR | cg14661225 | 19 | 46171451 | rs4399645 | 19 | 46166073 | T | C | 0.393638 | 0.000874776 | 0.000286604 | 0.00230001 | 0.342576 | | 0.0319916 | 9.31E-27 | 0.00255352 | 0.000869936 | 0.003332227 | 0.5842539 | 15 | ieu-b-4965 |
| NSUN4 | cg14993813 | 1 | 46806288 | rs6682266 | 1 | 46820419 | C | T | 0.272366 | -0.0650022 | 0.0241757 | 0.00717216 | -0.351591 | | 0.0346711 | 3.64E-24 | 0.18488 | 0.0711368 | 0.009351222 | 0.7311499 | 11 | ebi-a-GCST90013866 |
| NSUN4 | cg14993813 | 1 | 46806288 | rs6682266 | 1 | 46820419 | C | T | 0.272366 | -0.0653426 | 0.0244399 | 0.00728669 | -0.351591 | | 0.0346711 | 3.64E-24 | 0.185848 | 0.0718877 | 0.009730533 | 0.7366552 | 11 | ebi-a-GCST90013862 |
| HLA-DRA | cg15074838 | 6 | 32406521 | rs9272785 | 6 | 32610401 | A | G | 0.167992 | 0.0694096 | 0.0230264 | 0.00257531 | -0.223415 | | 0.04028 | 2.91E-08 | -0.310676 | 0.117303 | 0.008085046 | 0.11951 | 20 | ebi-a-GCST012879 |
| POMGNT1 | cg15157453 | 1 | 46663496 | rs34124738 | 1 | 46889844 | C | A | 0.400596 | 0.0535011 | 0.01841 | 0.00365982 | 0.188345 | | 0.0322785 | 5.38E-09 | 0.284059 | 0.109198 | 0.009286655 | 0.2533692 | 3 | ebi-a-GCST012876 |
| CUX2 | cg15920906 | 12 | 111619414 | rs7959625 | 12 | 111623138 | G | A | 0.17992 | 0.10022 | 0.0245015 | 4.31E-05 | -0.282811 | | 0.0440959 | 1.42E-10 | -0.354371 | 0.102755 | 0.000563329 | 0.3661568 | 11 | ebi-a-GCST012876 |
| CNP | cg15935113 | 17 | 40118557 | rs12952915 | 17 | 40118552 | A | G | 0.178926 | 0.11896 | 0.042261 | 0.00487966 | -0.481854 | | 0.0414258 | 2.84E-31 | -0.24688 | 0.0902366 | 0.006220711 | 0.1414609 | 8 | ebi-a-GCST012880 |
| HLA-DRB1 | cg15982117 | 6 | 32552106 | rs532965 | 6 | 32577973 | G | T | 0.156064 | 0.072252 | 0.0222876 | 0.00118779 | 0.699535 | | 0.0397467 | 2.47E-69 | 0.103286 | 0.0323966 | 0.001431764 | 0.4453043 | 20 | ebi-a-GCST012879 |
| ACSF3 | cg16692470 | 16 | 89184092 | rs7186059 | 16 | 89179874 | A | G | 0.22664 | 0.0724543 | 0.0216099 | 0.000799908 | -0.233164 | | 0.0381988 | 1.03E-09 | -0.310744 | 0.105742 | 0.003296066 | 0.6497665 | 20 | ebi-a-GCST012876 |
| C6orf10 | cg17039645 | 6 | 32294503 | rs35265698 | 6 | 32561334 | G | C | 0.159046 | 0.0699662 | 0.0222829 | 0.00169005 | -0.660702 | | 0.0394684 | 6.70E-63 | -0.105897 | 0.0343142 | 0.002028092 | 0.1695866 | 20 | ebi-a-GCST012879 |
| MLLT6 | cg17337668 | 17 | 36863216 | rs67365003 | 17 | 36847713 | A | G | 0.181909 | -0.0761011 | 0.0261654 | 0.00363212 | -0.313838 | | 0.0409137 | 1.71E-14 | 0.242485 | 0.0891641 | 0.006537316 | 0.1125464 | 7 | ebi-a-GCST90018588 |
| MLLT6 | cg17337668 | 17 | 36863216 | rs67365003 | 17 | 36847713 | A | G | 0.181909 | -0.06 | 0.0174 | 0.000563197 | -0.313838 | | 0.0409137 | 1.71E-14 | 0.191181 | 0.060787 | 0.001660289 | 0.2976926 | 11 | ebi-a-GCST90018808 |
| ZSCAN12L1 | cg17849569 | 6 | 28058911 | rs148696809 | 6 | 28934352 | C | T | 0.0695825 | 0.10088 | 0.033916 | 0.00293461 | 1.02061 | | 0.0467545 | 1.23E-105 | 0.0988429 | 0.0335382 | 0.003206928 | 0.708671 | 17 | ebi-a-GCST012877 |
| PSORS1C1 | cg17849733 | 6 | 31082200 | rs3130979 | 6 | 31082197 | C | G | 0.302187 | 0.0886738 | 0.022908 | 0.000117682 | -1.18402 | | 0.021639 | 0 | -0.0748921 | 0.019396 | 0.000112824 | 0.7867439 | 20 | ebi-a-GCST90013862 |
| PSORS1C1 | cg17849733 | 6 | 31082200 | rs3130979 | 6 | 31082197 | C | G | 0.302187 | 0.0896057 | 0.0232622 | 0.000117163 | -1.18402 | | 0.021639 | 0 | -0.0756792 | 0.0196954 | 0.000121799 | 0.7790968 | 20 | ebi-a-GCST90013866 |
| PSORS1C1 | cg17849733 | 6 | 31082200 | rs3130979 | 6 | 31082197 | C | G | 0.302187 | 0.064343 | 0.021003 | 0.00218731 | -1.18402 | | 0.021639 | 0 | -0.0543428 | 0.0177665 | 0.002222815 | 0.3808291 | 20 | ebi-a-GCST012877 |
| C6orf10 | cg17851230 | 6 | 32289373 | rs140566858 | 6 | 32226520 | G | T | 0.222664 | 0.0011021 | 0.00032728 | 0.000759994 | -0.344393 | | 0.038505 | 3.75E-19 | -0.00320012 | 0.00101543 | 0.001624406 | 0.3906611 | 20 | ieu-b-4965 |
| C6orf10 | cg17851230 | 6 | 32289373 | rs140566858 | 6 | 32226520 | G | T | 0.222664 | 0.0011021 | 0.00032728 | 0.000759994 | -0.344393 | | 0.038505 | 3.75E-19 | -0.00320012 | 0.00101543 | 0.001624406 | 0.3906611 | 20 | ieu-b-4965 |
| C6orf10 | cg17851230 | 6 | 32289373 | rs140566858 | 6 | 32226520 | G | T | 0.222664 | 0.0746438 | 0.0253985 | 0.00350792 | -0.344393 | | 0.038505 | 3.75E-19 | -0.21674 | 0.0776278 | 0.00523765 | 0.3854546 | 20 | ebi-a-GCST90013862 |
| C6orf10 | cg17851230 | 6 | 32289373 | rs140566858 | 6 | 32226520 | G | T | 0.222664 | 0.0754712 | 0.0259182 | 0.00359244 | -0.344393 | | 0.038505 | 3.75E-19 | -0.219143 | 0.0791456 | 0.005625368 | 0.3783856 | 20 | ebi-a-GCST90013866 |
| BAT4 | cg17867605 | 6 | 31634162 | rs1144708 | 6 | 31710020 | T | C | 0.361829 | 0.10037 | 0.032495 | 0.00200979 | -0.248294 | | 0.0343257 | 4.71E-13 | -0.404239 | 0.142305 | 0.004502297 | 0.1758121 | 3 | ebi-a-GCST012880 |
| COX15 | cg17885402 | 10 | 101492224 | rs2281636 | 10 | 101492403 | T | G | 0.40159 | 0.098768 | 0.03212 | 0.00210552 | -0.560988 | | 0.0290442 | 4.02E-83 | -0.176061 | 0.0579772 | 0.002391622 | 0.7864881 | 20 | ebi-a-GCST012880 |
| HLA-DOB | cg18073883 | 6 | 32780861 | rs241439 | 6 | 32797537 | G | T | 0.388668 | 0.000825648 | 0.000285948 | 0.00389996 | -0.263605 | | 0.0331729 | 1.92E-15 | -0.00313214 | 0.00115415 | 0.006651516 | 0.2610289 | 5 | ieu-b-4965 |
| HLA-DOB | cg18073883 | 6 | 32780861 | rs241439 | 6 | 32797537 | G | T | 0.388668 | 0.000825648 | 0.000285948 | 0.00389996 | -0.263605 | | 0.0331729 | 1.92E-15 | -0.00313214 | 0.00115415 | 0.006651516 | 0.2610289 | 5 | ieu-b-4965 |
| PNKD | cg18259342 | 2 | 219184859 | rs62183995 | 2 | 219189654 | A | G | 0.337972 | -0.0864842 | 0.022919 | 0.000160994 | -0.936536 | | 0.0277493 | 1.07E-249 | 0.0923448 | 0.0246246 | 0.000176761 | 0.8213614 | 20 | ebi-a-GCST90013866 |
| PNKD | cg18259342 | 2 | 219184859 | rs62183995 | 2 | 219189654 | A | G | 0.337972 | -0.0606499 | 0.0178062 | 0.000658931 | -0.936536 | | 0.0277493 | 1.07E-249 | 0.0647598 | 0.0191094 | 0.000701745 | 0.605463 | 20 | ebi-a-GCST012879 |
| PNKD | cg18259342 | 2 | 219184859 | rs62183995 | 2 | 219189654 | A | G | 0.337972 | -0.0428 | 0.0132 | 0.00119399 | -0.936536 | | 0.0277493 | 1.07E-249 | 0.0457003 | 0.0141594 | 0.001248493 | 0.6983789 | 20 | ebi-a-GCST90018808 |
| PNKD | cg18259342 | 2 | 219184859 | rs62183995 | 2 | 219189654 | A | G | 0.337972 | -0.00121994 | 0.000288931 | 2.40E-05 | -0.936536 | | 0.0277493 | 1.07E-249 | 0.00130261 | 0.000310915 | 2.79E-05 | 0.9838963 | 20 | ieu-b-4965 |
| PNKD | cg18259342 | 2 | 219184859 | rs62183995 | 2 | 219189654 | A | G | 0.337972 | -0.00121994 | 0.000288931 | 2.40E-05 | -0.936536 | | 0.0277493 | 1.07E-249 | 0.00130261 | 0.000310915 | 2.79E-05 | 0.9838963 | 20 | ieu-b-4965 |
| PNKD | cg18259342 | 2 | 219184859 | rs62183995 | 2 | 219189654 | A | G | 0.337972 | -0.0868369 | 0.0231015 | 0.000164165 | -0.936536 | | 0.0277493 | 1.07E-249 | 0.0927214 | 0.0248195 | 0.000187097 | 0.8211327 | 20 | ebi-a-GCST90013862 |
| TNXB | cg19108771 | 6 | 32076337 | rs2269426 | 6 | 32076499 | A | G | 0.412525 | -0.0614324 | 0.0176676 | 0.000506827 | 0.576523 | | 0.0326464 | 8.58E-70 | -0.106557 | 0.0312335 | 0.000645782 | 0.218673 | 20 | ebi-a-GCST90018588 |
| ARPC2 | cg19141132 | 2 | 219080920 | rs1877712 | 2 | 219168432 | G | A | 0.419483 | -0.0010466 | 0.000281824 | 2.00E-04 | 0.185238 | | 0.0320907 | 7.82E-09 | -0.00565003 | 0.00180908 | 0.001789294 | 0.1915506 | 6 | ieu-b-4965 |
| ARPC2 | cg19141132 | 2 | 219080920 | rs1877712 | 2 | 219168432 | G | A | 0.419483 | -0.0433 | 0.0124 | 0.000471704 | 0.185238 | | 0.0320907 | 7.82E-09 | -0.233753 | 0.0782366 | 0.002810181 | 0.1686245 | 6 | ebi-a-GCST90018808 |
| ARPC2 | cg19141132 | 2 | 219080920 | rs1877712 | 2 | 219168432 | G | A | 0.419483 | -0.0536898 | 0.018442 | 0.00359956 | 0.185238 | | 0.0320907 | 7.82E-09 | -0.289842 | 0.111504 | 0.009339041 | 0.5905234 | 6 | ebi-a-GCST012876 |
| ARPC2 | cg19141132 | 2 | 219080920 | rs1877712 | 2 | 219168432 | G | A | 0.419483 | -0.0640879 | 0.0170694 | 0.00017366 | 0.185238 | | 0.0320907 | 7.82E-09 | -0.345976 | 0.109926 | 0.001647602 | 0.6424625 | 6 | ebi-a-GCST012879 |
| ARPC2 | cg19141132 | 2 | 219080920 | rs1877712 | 2 | 219168432 | G | A | 0.419483 | -0.0010466 | 0.000281824 | 2.00E-04 | 0.185238 | | 0.0320907 | 7.82E-09 | -0.00565003 | 0.00180908 | 0.001789294 | 0.1915506 | 6 | ieu-b-4965 |
| ARPC2 | cg19141132 | 2 | 219080920 | rs1877712 | 2 | 219168432 | G | A | 0.419483 | -0.0745206 | 0.0223644 | 0.000861926 | 0.185238 | | 0.0320907 | 7.82E-09 | -0.402296 | 0.139405 | 0.003904096 | 0.1183225 | 6 | ebi-a-GCST90013866 |
| ARPC2 | cg19141132 | 2 | 219080920 | rs1877712 | 2 | 219168432 | G | A | 0.419483 | -0.0745639 | 0.0224172 | 0.000872409 | 0.185238 | | 0.0320907 | 7.82E-09 | -0.40253 | 0.139672 | 0.003952068 | 0.1172369 | 6 | ebi-a-GCST90013862 |
| TAP2 | cg19887824 | 6 | 32797476 | rs241439 | 6 | 32797537 | G | T | 0.388668 | 0.000825648 | 0.000285948 | 0.00389996 | -0.722375 | | 0.0310784 | 1.65E-119 | -0.00114296 | 0.000398887 | 0.004165063 | 0.4999417 | 20 | ieu-b-4965 |
| TAP2 | cg19887824 | 6 | 32797476 | rs241439 | 6 | 32797537 | G | T | 0.388668 | 0.000825648 | 0.000285948 | 0.00389996 | -0.722375 | | 0.0310784 | 1.65E-119 | -0.00114296 | 0.000398887 | 0.004165063 | 0.4999417 | 20 | ieu-b-4965 |
| HLA-DRB1 | cg20022036 | 6 | 32549496 | rs9271699 | 6 | 32593179 | G | A | 0.481113 | 0.0642417 | 0.022125 | 0.00368918 | 0.592903 | | 0.0296381 | 5.01E-89 | 0.108351 | 0.0377074 | 0.004059899 | 0.2139448 | 20 | ebi-a-GCST012876 |
| DPCR1 | cg20114524 | 6 | 30920086 | rs116311740 | 6 | 30907240 | C | G | 0.0357853 | 0.131909 | 0.0479257 | 0.00591684 | -2.98361 | | 0.0908036 | 8.80E-237 | -0.0442112 | 0.0161192 | 0.006092541 | 0.3435571 | 20 | ebi-a-GCST012876 |
| COX10 | cg20170777 | 17 | 13972099 | rs17609880 | 17 | 13996397 | G | A | 0.176938 | 0.0803937 | 0.0235002 | 0.00062395 | 0.287711 | | 0.0396919 | 4.21E-13 | 0.279425 | 0.0903195 | 0.001976498 | 0.6365806 | 9 | ebi-a-GCST012876 |
| LMF2 | cg21719361 | 22 | 50944355 | rs134970 | 22 | 50935082 | T | C | 0.385686 | -0.182333 | 0.0613138 | 0.002979 | -0.37172 | | 0.0323331 | 1.37E-30 | 0.490512 | 0.170375 | 0.003989219 | 0.1394072 | 9 | ebi-a-GCST012878 |
| CASP9 | cg21858823 | 1 | 15850916 | rs4646018 | 1 | 15840374 | T | C | 0.485089 | 0.0809 | 0.0276 | 0.003437 | 0.296591 | | 0.0328439 | 1.71E-19 | 0.272766 | 0.0978369 | 0.005303987 | 0.1274641 | 15 | finn-b-C3-COLO_EX |
| CASP9 | cg21858823 | 1 | 15850916 | rs4646018 | 1 | 15840374 | T | C | 0.485089 | 0.0839 | 0.0274 | 0.00218902 | 0.296591 | | 0.0328439 | 1.71E-19 | 0.282881 | 0.0975497 | 0.003733205 | 0.1495201 | 15 | finn-b-C3-COLO |
| UNC119B | cg21892295 | 12 | 121157589 | rs3752531 | 12 | 121157851 | G | A | 0.414513 | 0.0526005 | 0.0172715 | 0.00232284 | -0.922834 | | 0.0270914 | 2.54E-254 | -0.0569989 | 0.0187904 | 0.002418077 | 0.2082271 | 20 | ebi-a-GCST012879 |
| TRIM27 | cg21997109 | 6 | 28877723 | rs145648066 | 6 | 29146523 | C | T | 0.193837 | 0.064124 | 0.024038 | 0.00763871 | 0.513033 | | 0.0389829 | 1.48E-39 | 0.12499 | 0.0478076 | 0.008937367 | 0.6085719 | 13 | ebi-a-GCST012877 |
| TNXB | cg22056544 | 6 | 32038230 | rs6449 | 6 | 32006655 | T | C | 0.306163 | -0.0720166 | 0.0181628 | 7.34E-05 | 0.284427 | | 0.0369451 | 1.38E-14 | -0.253199 | 0.0718294 | 0.000423474 | 0.2358264 | 13 | ebi-a-GCST90018588 |
| POMGNT1 | cg22112435 | 1 | 46664449 | rs34124738 | 1 | 46889844 | C | A | 0.400596 | 0.0535011 | 0.01841 | 0.00365982 | 0.448953 | | 0.0319384 | 6.99E-45 | 0.119169 | 0.0418737 | 0.004428507 | 0.239592 | 3 | ebi-a-GCST012876 |
| HLA-B | cg22731440 | 6 | 31323506 | rs2596495 | 6 | 31323416 | C | G | 0.0954274 | 0.0974121 | 0.0300395 | 0.0013444 | -1.67811 | | 0.0233852 | 0 | -0.0580487 | 0.0179191 | 0.001197419 | 0.8473811 | 20 | ebi-a-GCST90013862 |
| HLA-B | cg22731440 | 6 | 31323506 | rs2596495 | 6 | 31323416 | C | G | 0.0954274 | 0.0994238 | 0.0310847 | 0.00138153 | -1.67811 | | 0.0233852 | 0 | -0.0592475 | 0.018542 | 0.001396818 | 0.8264407 | 20 | ebi-a-GCST90013866 |
| MOG | cg23019585 | 6 | 29635110 | rs2747421 | 6 | 29645118 | C | G | 0.188867 | 0.0699392 | 0.0252612 | 0.00592652 | 0.919596 | | 0.0335351 | 1.50E-165 | 0.0760543 | 0.0276095 | 0.005875735 | 0.8085225 | 20 | ebi-a-GCST90013862 |
| MOG | cg23019585 | 6 | 29635110 | rs2747421 | 6 | 29645118 | C | G | 0.188867 | 0.070642 | 0.0257331 | 0.0060477 | 0.919596 | | 0.0335351 | 1.50E-165 | 0.0768185 | 0.0281229 | 0.006304157 | 0.8116171 | 20 | ebi-a-GCST90013866 |
| HLA-DQB1 | cg23214071 | 6 | 32627784 | rs9274623 | 6 | 32635998 | T | G | 0.202783 | 0.11358 | 0.042824 | 0.00799264 | -1.04398 | | 0.0308717 | 1.12E-250 | -0.108795 | 0.0411459 | 0.008190092 | 0.3042797 | 20 | ebi-a-GCST012880 |
| BAD | cg23796481 | 11 | 64053134 | rs479552 | 11 | 64110683 | C | G | 0.371769 | 0.0656452 | 0.0228564 | 0.00407793 | 0.535788 | | 0.0313902 | 2.54E-65 | 0.122521 | 0.0432591 | 0.004622093 | 0.8321523 | 20 | ebi-a-GCST90013866 |
| BAD | cg23796481 | 11 | 64053134 | rs479552 | 11 | 64110683 | C | G | 0.371769 | 0.0652208 | 0.0226448 | 0.00408686 | 0.535788 | | 0.0313902 | 2.54E-65 | 0.121729 | 0.042862 | 0.004511095 | 0.8314332 | 20 | ebi-a-GCST90013862 |
| ARHGAP29 | cg24049050 | 1 | 94702435 | rs11165099 | 1 | 94702240 | A | T | 0.299205 | 0.187681 | 0.0602685 | 0.001869 | 0.254369 | | 0.0340519 | 8.02E-14 | 0.73783 | 0.256697 | 0.004048954 | 0.1433714 | 13 | ebi-a-GCST012878 |
| MPRIP | cg24170090 | 17 | 16945301 | rs78365041 | 17 | 16945586 | G | T | 0.198807 | -0.0646964 | 0.0233073 | 0.00550668 | -0.537123 | | 0.0434958 | 4.94E-35 | 0.12045 | 0.0444756 | 0.006764441 | 0.1543763 | 20 | ebi-a-GCST012879 |
| ABCD3 | cg24215727 | 1 | 94883469 | rs2786906 | 1 | 94824664 | A | G | 0.336978 | 0.176664 | 0.0589015 | 0.00273899 | -0.206299 | | 0.034305 | 1.81E-09 | -0.856349 | 0.319056 | 0.007274517 | 0.5659024 | 15 | ebi-a-GCST012878 |
| MOG | cg24444631 | 6 | 29636366 | rs2747429 | 6 | 29648377 | C | T | 0.188867 | 0.0700328 | 0.0257371 | 0.00650699 | 0.506028 | | 0.0371896 | 3.65E-42 | 0.138397 | 0.0518681 | 0.007624709 | 0.4985778 | 20 | ebi-a-GCST90013866 |
| MOG | cg24444631 | 6 | 29636366 | rs2747429 | 6 | 29648377 | C | T | 0.188867 | 0.0693408 | 0.0252683 | 0.00637911 | 0.506028 | | 0.0371896 | 3.65E-42 | 0.13703 | 0.05094 | 0.007144783 | 0.5032069 | 20 | ebi-a-GCST90013862 |
| HIST1H2AI | cg25108566 | 6 | 27775657 | rs148696809 | 6 | 28934352 | C | T | 0.0695825 | 0.10088 | 0.033916 | 0.00293461 | 0.415262 | | 0.0479097 | 4.41E-18 | 0.242931 | 0.0863489 | 0.004902614 | 0.6558594 | 9 | ebi-a-GCST012877 |
| HLA-L | cg25643819 | 6 | 30227942 | rs1639111 | 6 | 31222116 | A | G | 0.115308 | 0.0787868 | 0.0241743 | 0.00111761 | -0.250093 | | 0.0455571 | 4.03E-08 | -0.31503 | 0.112412 | 0.005071627 | 0.4509739 | 9 | ebi-a-GCST012879 |
| TAP2 | cg25744682 | 6 | 32797578 | rs241437 | 6 | 32797684 | G | A | 0.39165 | 0.000797952 | 0.000285725 | 0.00519996 | -1.08916 | | 0.0246534 | 0 | -0.000732631 | 0.000262859 | 0.005317155 | 0.705735 | 20 | ieu-b-4965 |
| TAP2 | cg25744682 | 6 | 32797578 | rs241437 | 6 | 32797684 | G | A | 0.39165 | 0.000797952 | 0.000285725 | 0.00519996 | -1.08916 | | 0.0246534 | 0 | -0.000732631 | 0.000262859 | 0.005317155 | 0.705735 | 20 | ieu-b-4965 |
| HLA-B | cg25954539 | 6 | 31323677 | rs4081560 | 6 | 31323567 | A | G | 0.114314 | 0.13355 | 0.044956 | 0.00297201 | -1.04821 | | 0.0436939 | 3.55E-127 | -0.127408 | 0.0432159 | 0.003196666 | 0.496393 | 20 | ebi-a-GCST012880 |

**Supplementary Table 6.** Sensitivity analysis used TwoSampleMR package on the association between mitochondria-related genes mQTL and CRC outcomes.

| **Gene name** | **Var.35** | **0.1** | **Outcome** | **Exposure** | **Method** | **Heterogeneity test** | | **Pleiotropy test** | |
| --- | --- | --- | --- | --- | --- | --- | --- | --- | --- |
|  |  |  |  |  |  | **Cochran's Q** | ***P*** | **Intercept** | ***P*** |
| SLC25A30 | 1561 | 6 | Colorectal cancer (SPA correction) || id:ebi-a-GCST90013866 | cg00139037 | Inverse variance weighted | 5.603571781 | 1 | NA | NA |
| SLC25A30 | 2090 | 6 | Colorectal cancer || id:ebi-a-GCST90018588 | cg00139037 | Inverse variance weighted | 18.92815105 | 1 | NA | NA |
| SLC25A30 | 3584 | 6 | Colorectal cancer || id:ebi-a-GCST012878 | cg00139037 | Inverse variance weighted | 6.674400139 | 1 | NA | NA |
| SLC25A30 | 6 | 6 | Colorectal cancer || id:ieu-b-4965 | cg00139037 | Inverse variance weighted | 4.719549312 | 1 | NA | NA |
| SLC25A30 | 1033 | 6 | Colorectal cancer (Firth correction) || id:ebi-a-GCST90013862 | cg00139037 | Inverse variance weighted | 5.614541133 | 1 | NA | NA |
| SLC25A30 | 522 | 6 | Colorectal cancer || id:ebi-a-GCST90018808 | cg00139037 | Inverse variance weighted | 3.923847462 | 1 | NA | NA |
| SLC25A30 | 5607 | 6 | Colorectal cancer (all cancers excluded) || id:finn-b-C3_COLORECTAL_EXALLC | cg00139037 | Inverse variance weighted | 2.677122372 | 1 | NA | NA |
| SLC25A30 | 5103 | 6 | Colorectal cancer || id:finn-b-C3_COLORECTAL | cg00139037 | Inverse variance weighted | 1.975764006 | 1 | NA | NA |
| SLC25A30 | 3066 | 6 | Colorectal cancer || id:ebi-a-GCST012877 | cg00139037 | Inverse variance weighted | 8.517717575 | 1 | NA | NA |
| SLC25A30 | 2553 | 6 | Colorectal cancer || id:ebi-a-GCST012876 | cg00139037 | Inverse variance weighted | 27.37098992 | 0.99999997 | NA | NA |
| SLC25A30 | 5606 | 5 | Colorectal cancer (all cancers excluded) || id:finn-b-C3_COLORECTAL_EXALLC | cg00139037 | MR Egger | 2.610523126 | 1 | 0.007510061 | 0.797061036 |
| SLC25A30 | 1032 | 5 | Colorectal cancer (Firth correction) || id:ebi-a-GCST90013862 | cg00139037 | MR Egger | 4.843818217 | 1 | -0.021166159 | 0.382835018 |
| SLC25A30 | 1560 | 5 | Colorectal cancer (SPA correction) || id:ebi-a-GCST90013866 | cg00139037 | MR Egger | 4.873469265 | 1 | -0.020533089 | 0.395571099 |
| SLC25A30 | 2552 | 5 | Colorectal cancer || id:ebi-a-GCST012876 | cg00139037 | MR Egger | 9.728666734 | 1 | -0.081314886 | 7.18E-05 |
| SLC25A30 | 3065 | 5 | Colorectal cancer || id:ebi-a-GCST012877 | cg00139037 | MR Egger | 4.538942522 | 1 | -0.042517569 | 0.04976039 |
| SLC25A30 | 3583 | 5 | Colorectal cancer || id:ebi-a-GCST012878 | cg00139037 | MR Egger | 1.47462586 | 1 | -0.138019045 | 0.025320932 |
| SLC25A30 | 2089 | 5 | Colorectal cancer || id:ebi-a-GCST90018588 | cg00139037 | MR Egger | 14.7333688 | 1 | 0.050332704 | 0.043748356 |
| SLC25A30 | 521 | 5 | Colorectal cancer || id:ebi-a-GCST90018808 | cg00139037 | MR Egger | 1.804636874 | 1 | 0.022098236 | 0.149374732 |
| SLC25A30 | 5102 | 5 | Colorectal cancer || id:finn-b-C3_COLORECTAL | cg00139037 | MR Egger | 1.970049511 | 1 | -0.002239977 | 0.939951542 |
| SLC25A30 | 5 | 5 | Colorectal cancer || id:ieu-b-4965 | cg00139037 | MR Egger | 4.541714445 | 1 | -0.000128486 | 0.674463103 |
| MRPL32 | 3072 | 12 | Colorectal cancer || id:ebi-a-GCST012877 | cg00365680 | Inverse variance weighted | 44.41189417 | 1 | NA | NA |
| MRPL32 | 2092 | 12 | Colorectal cancer || id:ebi-a-GCST90018588 | cg00365680 | Inverse variance weighted | 101.0042343 | 0.999070509 | NA | NA |
| MRPL32 | 5107 | 12 | Colorectal cancer || id:finn-b-C3_COLORECTAL | cg00365680 | Inverse variance weighted | 102.3955229 | 0.999999998 | NA | NA |
| MRPL32 | 1563 | 12 | Colorectal cancer (SPA correction) || id:ebi-a-GCST90013866 | cg00365680 | Inverse variance weighted | 53.44343393 | 1 | NA | NA |
| MRPL32 | 5611 | 12 | Colorectal cancer (all cancers excluded) || id:finn-b-C3_COLORECTAL_EXALLC | cg00365680 | Inverse variance weighted | 96.31779351 | 1 | NA | NA |
| MRPL32 | 1037 | 12 | Colorectal cancer (Firth correction) || id:ebi-a-GCST90013862 | cg00365680 | Inverse variance weighted | 53.20655317 | 1 | NA | NA |
| MRPL32 | 3588 | 12 | Colorectal cancer || id:ebi-a-GCST012878 | cg00365680 | Inverse variance weighted | 94.73335933 | 1 | NA | NA |
| MRPL32 | 4110 | 12 | Colorectal cancer || id:ebi-a-GCST012879 | cg00365680 | Inverse variance weighted | 32.40414157 | 1 | NA | NA |
| MRPL32 | 526 | 12 | Colorectal cancer || id:ebi-a-GCST90018808 | cg00365680 | Inverse variance weighted | 51.38228138 | 1 | NA | NA |
| MRPL32 | 2559 | 12 | Colorectal cancer || id:ebi-a-GCST012876 | cg00365680 | Inverse variance weighted | 91.8042533 | 1 | NA | NA |
| MRPL32 | 4617 | 12 | Colorectal cancer || id:ebi-a-GCST012880 | cg00365680 | Inverse variance weighted | 20.72216116 | 1 | NA | NA |
| MRPL32 | 8 | 12 | Colorectal cancer || id:ieu-b-4965 | cg00365680 | Inverse variance weighted | 38.43150187 | 1 | NA | NA |
| MRPL32 | 5610 | 11 | Colorectal cancer (all cancers excluded) || id:finn-b-C3_COLORECTAL_EXALLC | cg00365680 | MR Egger | 55.26924636 | 1 | -0.062446169 | 1.07E-09 |
| MRPL32 | 1036 | 11 | Colorectal cancer (Firth correction) || id:ebi-a-GCST90013862 | cg00365680 | MR Egger | 45.3279466 | 1 | -0.021501347 | 0.005616497 |
| MRPL32 | 1562 | 11 | Colorectal cancer (SPA correction) || id:ebi-a-GCST90013866 | cg00365680 | MR Egger | 45.4705817 | 1 | -0.021616039 | 0.005344612 |
| MRPL32 | 2558 | 11 | Colorectal cancer || id:ebi-a-GCST012876 | cg00365680 | MR Egger | 68.3073604 | 1 | -0.026797119 | 2.54E-06 |
| MRPL32 | 3071 | 11 | Colorectal cancer || id:ebi-a-GCST012877 | cg00365680 | MR Egger | 35.04075828 | 1 | 0.018618707 | 0.002513455 |
| MRPL32 | 3587 | 11 | Colorectal cancer || id:ebi-a-GCST012878 | cg00365680 | MR Egger | 93.74311011 | 1 | -0.017120917 | 0.320908403 |
| MRPL32 | 4109 | 11 | Colorectal cancer || id:ebi-a-GCST012879 | cg00365680 | MR Egger | 24.8954221 | 1 | 0.014354067 | 0.006710185 |
| MRPL32 | 4616 | 11 | Colorectal cancer || id:ebi-a-GCST012880 | cg00365680 | MR Egger | 20.67257205 | 1 | -0.002133606 | 0.824011695 |
| MRPL32 | 2091 | 11 | Colorectal cancer || id:ebi-a-GCST90018588 | cg00365680 | MR Egger | 100.5204121 | 0.99898733 | 0.005672016 | 0.487786847 |
| MRPL32 | 525 | 11 | Colorectal cancer || id:ebi-a-GCST90018808 | cg00365680 | MR Egger | 50.13567936 | 1 | -0.005136116 | 0.265555555 |
| MRPL32 | 5106 | 11 | Colorectal cancer || id:finn-b-C3_COLORECTAL | cg00365680 | MR Egger | 57.90389975 | 1 | -0.064152939 | 2.52E-10 |
| MRPL32 | 7 | 11 | Colorectal cancer || id:ieu-b-4965 | cg00365680 | MR Egger | 35.1480464 | 1 | 0.000152454 | 0.071511913 |
| ACSF3 | 5653 | 56 | Colorectal cancer (all cancers excluded) || id:finn-b-C3_COLORECTAL_EXALLC | cg02193283 | Inverse variance weighted | 464.6927456 | 3.33E-06 | NA | NA |
| ACSF3 | 2130 | 58 | Colorectal cancer || id:ebi-a-GCST90018588 | cg02193283 | Inverse variance weighted | 178.3898147 | 0.998509037 | NA | NA |
| ACSF3 | 3624 | 54 | Colorectal cancer || id:ebi-a-GCST012878 | cg02193283 | Inverse variance weighted | 78.87197956 | 1 | NA | NA |
| ACSF3 | 1073 | 54 | Colorectal cancer (Firth correction) || id:ebi-a-GCST90013862 | cg02193283 | Inverse variance weighted | 61.01244853 | 1 | NA | NA |
| ACSF3 | 562 | 56 | Colorectal cancer || id:ebi-a-GCST90018808 | cg02193283 | Inverse variance weighted | 193.3306531 | 1 | NA | NA |
| ACSF3 | 5149 | 56 | Colorectal cancer || id:finn-b-C3_COLORECTAL | cg02193283 | Inverse variance weighted | 464.4501226 | 4.85E-06 | NA | NA |
| ACSF3 | 3106 | 56 | Colorectal cancer || id:ebi-a-GCST012877 | cg02193283 | Inverse variance weighted | 134.647528 | 1 | NA | NA |
| ACSF3 | 4146 | 58 | Colorectal cancer || id:ebi-a-GCST012879 | cg02193283 | Inverse variance weighted | 187.0244664 | 1 | NA | NA |
| ACSF3 | 4647 | 56 | Colorectal cancer || id:ebi-a-GCST012880 | cg02193283 | Inverse variance weighted | 91.05526753 | 1 | NA | NA |
| ACSF3 | 2593 | 58 | Colorectal cancer || id:ebi-a-GCST012876 | cg02193283 | Inverse variance weighted | 336.0613273 | 0.565319339 | NA | NA |
| ACSF3 | 48 | 58 | Colorectal cancer || id:ieu-b-4965 | cg02193283 | Inverse variance weighted | 225.1698272 | 0.999998782 | NA | NA |
| ACSF3 | 1601 | 56 | Colorectal cancer (SPA correction) || id:ebi-a-GCST90013866 | cg02193283 | Inverse variance weighted | 61.1627358 | 1 | NA | NA |
| ACSF3 | 4162 | 82 | Colorectal cancer || id:ebi-a-GCST012879 | cg02691035 | Inverse variance weighted | 169.3662391 | 1 | NA | NA |
| ACSF3 | 2142 | 81 | Colorectal cancer || id:ebi-a-GCST90018588 | cg02691035 | Inverse variance weighted | 357.1518818 | 0.990221318 | NA | NA |
| ACSF3 | 4667 | 80 | Colorectal cancer || id:ebi-a-GCST012880 | cg02691035 | Inverse variance weighted | 523.0688202 | 0.002874764 | NA | NA |
| ACSF3 | 576 | 79 | Colorectal cancer || id:ebi-a-GCST90018808 | cg02691035 | Inverse variance weighted | 626.4698112 | 1.36E-08 | NA | NA |
| ACSF3 | 5169 | 80 | Colorectal cancer || id:finn-b-C3_COLORECTAL | cg02691035 | Inverse variance weighted | 254.0139976 | 1 | NA | NA |
| ACSF3 | 63 | 81 | Colorectal cancer || id:ieu-b-4965 | cg02691035 | Inverse variance weighted | 317.0513057 | 0.999993849 | NA | NA |
| ACSF3 | 2603 | 82 | Colorectal cancer || id:ebi-a-GCST012876 | cg02691035 | Inverse variance weighted | 496.3305905 | 0.025890574 | NA | NA |
| ACSF3 | 3124 | 80 | Colorectal cancer || id:ebi-a-GCST012877 | cg02691035 | Inverse variance weighted | 163.4285625 | 1 | NA | NA |
| ACSF3 | 1087 | 76 | Colorectal cancer (Firth correction) || id:ebi-a-GCST90013862 | cg02691035 | Inverse variance weighted | 301.6935394 | 0.99072092 | NA | NA |
| ACSF3 | 5672 | 79 | Colorectal cancer (all cancers excluded) || id:finn-b-C3_COLORECTAL_EXALLC | cg02691035 | Inverse variance weighted | 184.001435 | 1 | NA | NA |
| ACSF3 | 3640 | 77 | Colorectal cancer || id:ebi-a-GCST012878 | cg02691035 | Inverse variance weighted | 95.14039541 | 1 | NA | NA |
| ACSF3 | 1617 | 80 | Colorectal cancer (SPA correction) || id:ebi-a-GCST90013866 | cg02691035 | Inverse variance weighted | 301.6935394 | 0.99072092 | NA | NA |
| ACSF3 | 5690 | 109 | Colorectal cancer (all cancers excluded) || id:finn-b-C3_COLORECTAL_EXALLC | cg04308346 | Inverse variance weighted | 1.104688205 | 0.999155916 | NA | NA |
| ACSF3 | 5191 | 110 | Colorectal cancer || id:finn-b-C3_COLORECTAL | cg04308346 | Inverse variance weighted | 1.02686784 | 0.99937305 | NA | NA |
| ACSF3 | 4184 | 112 | Colorectal cancer || id:ebi-a-GCST012879 | cg04308346 | Inverse variance weighted | 0.025988562 | 1 | NA | NA |
| ACSF3 | 4691 | 110 | Colorectal cancer || id:ebi-a-GCST012880 | cg04308346 | Inverse variance weighted | 0.207111849 | 0.99999935 | NA | NA |
| ACSF3 | 2627 | 112 | Colorectal cancer || id:ebi-a-GCST012876 | cg04308346 | Inverse variance weighted | 0.136839635 | 0.999999896 | NA | NA |
| ACSF3 | 5652 | 55 | Colorectal cancer (all cancers excluded) || id:finn-b-C3_COLORECTAL_EXALLC | cg02193283 | MR Egger | 460.4487075 | 5.20E-06 | -0.019734072 | 0.080248778 |
| ACSF3 | 5671 | 78 | Colorectal cancer (all cancers excluded) || id:finn-b-C3_COLORECTAL_EXALLC | cg02691035 | MR Egger | 174.0528734 | 1 | 0.025758194 | 0.001721346 |
| ACSF3 | 5689 | 108 | Colorectal cancer (all cancers excluded) || id:finn-b-C3_COLORECTAL_EXALLC | cg04308346 | MR Egger | 0.993291325 | 0.998290409 | -0.108533933 | 0.747139641 |
| ACSF3 | 1072 | 53 | Colorectal cancer (Firth correction) || id:ebi-a-GCST90013862 | cg02193283 | MR Egger | 57.45726883 | 1 | 0.015303989 | 0.060349098 |
| ACSF3 | 1086 | 75 | Colorectal cancer (Firth correction) || id:ebi-a-GCST90013862 | cg02691035 | MR Egger | 192.3737044 | 1 | 0.084570938 | 1.58E-22 |
| ACSF3 | 1600 | 55 | Colorectal cancer (SPA correction) || id:ebi-a-GCST90013866 | cg02193283 | MR Egger | 57.58923562 | 1 | 0.01534211 | 0.059695144 |
| ACSF3 | 1616 | 79 | Colorectal cancer (SPA correction) || id:ebi-a-GCST90013866 | cg02691035 | MR Egger | 192.3737044 | 1 | 0.084570938 | 1.58E-22 |
| ACSF3 | 2592 | 57 | Colorectal cancer || id:ebi-a-GCST012876 | cg02193283 | MR Egger | 299.8208038 | 0.942972607 | -0.037942268 | 4.52E-09 |
| ACSF3 | 2602 | 81 | Colorectal cancer || id:ebi-a-GCST012876 | cg02691035 | MR Egger | 458.1323768 | 0.223728251 | -0.039542676 | 3.51E-09 |
| ACSF3 | 2626 | 111 | Colorectal cancer || id:ebi-a-GCST012876 | cg04308346 | MR Egger | 0.025770269 | 0.999999999 | -0.070443984 | 0.747496503 |
| ACSF3 | 3105 | 55 | Colorectal cancer || id:ebi-a-GCST012877 | cg02193283 | MR Egger | 134.1517817 | 1 | 0.004727988 | 0.481855647 |
| ACSF3 | 3123 | 79 | Colorectal cancer || id:ebi-a-GCST012877 | cg02691035 | MR Egger | 159.3476533 | 1 | -0.0139117 | 0.04398136 |
| ACSF3 | 3623 | 53 | Colorectal cancer || id:ebi-a-GCST012878 | cg02193283 | MR Egger | 78.82813524 | 1 | -0.00432231 | 0.834268572 |
| ACSF3 | 3639 | 76 | Colorectal cancer || id:ebi-a-GCST012878 | cg02691035 | MR Egger | 77.75940249 | 1 | 0.090056561 | 3.69E-05 |
| ACSF3 | 4145 | 57 | Colorectal cancer || id:ebi-a-GCST012879 | cg02193283 | MR Egger | 167.8864018 | 1 | -0.026112754 | 1.62E-05 |
| ACSF3 | 4161 | 81 | Colorectal cancer || id:ebi-a-GCST012879 | cg02691035 | MR Egger | 118.5092606 | 1 | 0.043985155 | 4.16E-12 |
| ACSF3 | 4183 | 111 | Colorectal cancer || id:ebi-a-GCST012879 | cg04308346 | MR Egger | 0.025977776 | 0.999999999 | 0.00065489 | 0.997460015 |
| ACSF3 | 4646 | 55 | Colorectal cancer || id:ebi-a-GCST012880 | cg02193283 | MR Egger | 90.21712529 | 1 | 0.009921926 | 0.360573683 |
| ACSF3 | 4666 | 79 | Colorectal cancer || id:ebi-a-GCST012880 | cg02691035 | MR Egger | 453.2624678 | 0.274404956 | 0.092891987 | 2.83E-15 |
| ACSF3 | 4690 | 109 | Colorectal cancer || id:ebi-a-GCST012880 | cg04308346 | MR Egger | 0.183152784 | 0.999997276 | 0.05659783 | 0.880823053 |
| ACSF3 | 2129 | 57 | Colorectal cancer || id:ebi-a-GCST90018588 | cg02193283 | MR Egger | 177.1838205 | 0.998599176 | -0.007545777 | 0.273241331 |
| ACSF3 | 2141 | 80 | Colorectal cancer || id:ebi-a-GCST90018588 | cg02691035 | MR Egger | 353.3471138 | 0.992782552 | -0.009179452 | 0.051770003 |
| ACSF3 | 561 | 55 | Colorectal cancer || id:ebi-a-GCST90018808 | cg02193283 | MR Egger | 193.2102027 | 1 | -0.00163092 | 0.728758708 |
| ACSF3 | 575 | 78 | Colorectal cancer || id:ebi-a-GCST90018808 | cg02691035 | MR Egger | 463.1090987 | 0.215303973 | 0.048453289 | 1.00E-30 |
| ACSF3 | 5148 | 55 | Colorectal cancer || id:finn-b-C3_COLORECTAL | cg02193283 | MR Egger | 459.0783552 | 8.84E-06 | -0.021715295 | 0.04820077 |
| ACSF3 | 5168 | 79 | Colorectal cancer || id:finn-b-C3_COLORECTAL | cg02691035 | MR Egger | 206.2885001 | 1 | 0.055556332 | 1.75E-11 |
| ACSF3 | 5190 | 109 | Colorectal cancer || id:finn-b-C3_COLORECTAL | cg04308346 | MR Egger | 0.951968501 | 0.998534002 | -0.088051478 | 0.791265641 |
| ACSF3 | 47 | 57 | Colorectal cancer || id:ieu-b-4965 | cg02193283 | MR Egger | 224.0696082 | 0.999998865 | -0.000100527 | 0.294980993 |
| ACSF3 | 62 | 80 | Colorectal cancer || id:ieu-b-4965 | cg02691035 | MR Egger | 221.9124886 | 1 | 0.000934011 | 1.89E-20 |
| ME3 | 5668 | 75 | Colorectal cancer (all cancers excluded) || id:finn-b-C3_COLORECTAL_EXALLC | cg02493602 | Inverse variance weighted | 77.72506016 | 1 | NA | NA |
| ME3 | 4663 | 76 | Colorectal cancer || id:ebi-a-GCST012880 | cg02493602 | Inverse variance weighted | 91.17124789 | 1 | NA | NA |
| ME3 | 5165 | 76 | Colorectal cancer || id:finn-b-C3_COLORECTAL | cg02493602 | Inverse variance weighted | 89.85020099 | 1 | NA | NA |
| ME3 | 4158 | 78 | Colorectal cancer || id:ebi-a-GCST012879 | cg02493602 | Inverse variance weighted | 147.7086588 | 0.999982567 | NA | NA |
| ME3 | 1613 | 76 | Colorectal cancer (SPA correction) || id:ebi-a-GCST90013866 | cg02493602 | Inverse variance weighted | 173.4369474 | 0.718801542 | NA | NA |
| ME3 | 1083 | 72 | Colorectal cancer (Firth correction) || id:ebi-a-GCST90013862 | cg02493602 | Inverse variance weighted | 173.0013496 | 0.726726316 | NA | NA |
| ME3 | 572 | 75 | Colorectal cancer || id:ebi-a-GCST90018808 | cg02493602 | Inverse variance weighted | 136.7544458 | 0.999998252 | NA | NA |
| ME3 | 3636 | 73 | Colorectal cancer || id:ebi-a-GCST012878 | cg02493602 | Inverse variance weighted | 143.6326356 | 0.99999308 | NA | NA |
| ME3 | 3120 | 76 | Colorectal cancer || id:ebi-a-GCST012877 | cg02493602 | Inverse variance weighted | 315.869539 | 7.25E-05 | NA | NA |
| ME3 | 2138 | 77 | Colorectal cancer || id:ebi-a-GCST90018588 | cg02493602 | Inverse variance weighted | 418.5822497 | 3.14E-15 | NA | NA |
| ME3 | 4811 | 288 | Colorectal cancer || id:ebi-a-GCST012880 | cg11602041 | Inverse variance weighted | 41.93835102 | 1 | NA | NA |
| ME3 | 5326 | 294 | Colorectal cancer || id:finn-b-C3_COLORECTAL | cg11602041 | Inverse variance weighted | 28.3957952 | 1 | NA | NA |
| ME3 | 2764 | 296 | Colorectal cancer || id:ebi-a-GCST012876 | cg11602041 | Inverse variance weighted | 22.54512052 | 1 | NA | NA |
| ME3 | 3806 | 284 | Colorectal cancer || id:ebi-a-GCST012878 | cg11602041 | Inverse variance weighted | 44.89612427 | 1 | NA | NA |
| ME3 | 4315 | 294 | Colorectal cancer || id:ebi-a-GCST012879 | cg11602041 | Inverse variance weighted | 38.57467893 | 1 | NA | NA |
| ME3 | 221 | 289 | Colorectal cancer || id:ieu-b-4965 | cg11602041 | Inverse variance weighted | 62.42320481 | 1 | NA | NA |
| ME3 | 3281 | 288 | Colorectal cancer || id:ebi-a-GCST012877 | cg11602041 | Inverse variance weighted | 100.7134229 | 0.999966374 | NA | NA |
| ME3 | 5819 | 289 | Colorectal cancer (all cancers excluded) || id:finn-b-C3_COLORECTAL_EXALLC | cg11602041 | Inverse variance weighted | 22.18422706 | 1 | NA | NA |
| ME3 | 1249 | 286 | Colorectal cancer (Firth correction) || id:ebi-a-GCST90013862 | cg11602041 | Inverse variance weighted | 114.0308464 | 0.904529032 | NA | NA |
| ME3 | 1783 | 290 | Colorectal cancer (SPA correction) || id:ebi-a-GCST90013866 | cg11602041 | Inverse variance weighted | 113.707237 | 0.90821904 | NA | NA |
| ME3 | 733 | 286 | Colorectal cancer || id:ebi-a-GCST90018808 | cg11602041 | Inverse variance weighted | 233.8651807 | 0.00058985 | NA | NA |
| ME3 | 2469 | 561 | Colorectal cancer || id:ebi-a-GCST90018588 | cg22814480 | Inverse variance weighted | 17.30391776 | 0.998420446 | NA | NA |
| ME3 | 4016 | 569 | Colorectal cancer || id:ebi-a-GCST012878 | cg22814480 | Inverse variance weighted | 8.496808981 | 0.999999941 | NA | NA |
| ME3 | 1996 | 573 | Colorectal cancer (SPA correction) || id:ebi-a-GCST90013866 | cg22814480 | Inverse variance weighted | 18.22259077 | 0.991331786 | NA | NA |
| ME3 | 1467 | 570 | Colorectal cancer (Firth correction) || id:ebi-a-GCST90013862 | cg22814480 | Inverse variance weighted | 18.22259077 | 0.991331786 | NA | NA |
| ME3 | 4525 | 577 | Colorectal cancer || id:ebi-a-GCST012879 | cg22814480 | Inverse variance weighted | 7.448489872 | 0.999999993 | NA | NA |
| ME3 | 5011 | 571 | Colorectal cancer || id:ebi-a-GCST012880 | cg22814480 | Inverse variance weighted | 11.50234065 | 0.999994694 | NA | NA |
| ME3 | 941 | 570 | Colorectal cancer || id:ebi-a-GCST90018808 | cg22814480 | Inverse variance weighted | 15.59213323 | 0.999933906 | NA | NA |
| ME3 | 3500 | 574 | Colorectal cancer || id:ebi-a-GCST012877 | cg22814480 | Inverse variance weighted | 13.65600653 | 0.999944812 | NA | NA |
| ME3 | 2976 | 583 | Colorectal cancer || id:ebi-a-GCST012876 | cg22814480 | Inverse variance weighted | 3.601775041 | 1 | NA | NA |
| ME3 | 6022 | 575 | Colorectal cancer (all cancers excluded) || id:finn-b-C3_COLORECTAL_EXALLC | cg22814480 | Inverse variance weighted | 12.80764072 | 0.99999609 | NA | NA |
| ME3 | 440 | 573 | Colorectal cancer || id:ieu-b-4965 | cg22814480 | Inverse variance weighted | 7.673039048 | 0.999999995 | NA | NA |
| ME3 | 5523 | 579 | Colorectal cancer || id:finn-b-C3_COLORECTAL | cg22814480 | Inverse variance weighted | 13.58454461 | 0.999990645 | NA | NA |
| ME3 | 5667 | 74 | Colorectal cancer (all cancers excluded) || id:finn-b-C3_COLORECTAL_EXALLC | cg02493602 | MR Egger | 76.41204566 | 1 | 0.007476979 | 0.253061451 |
| ME3 | 5818 | 288 | Colorectal cancer (all cancers excluded) || id:finn-b-C3_COLORECTAL_EXALLC | cg11602041 | MR Egger | 13.35472719 | 1 | -0.034534933 | 0.003418681 |
| ME3 | 6021 | 574 | Colorectal cancer (all cancers excluded) || id:finn-b-C3_COLORECTAL_EXALLC | cg22814480 | MR Egger | 10.84932107 | 0.9999994 | 0.027029528 | 0.169213821 |
| ME3 | 1082 | 71 | Colorectal cancer (Firth correction) || id:ebi-a-GCST90013862 | cg02493602 | MR Egger | 116.9322974 | 0.999969276 | -0.044794734 | 2.82E-12 |
| ME3 | 1248 | 285 | Colorectal cancer (Firth correction) || id:ebi-a-GCST90013862 | cg11602041 | MR Egger | 113.7926432 | 0.896388668 | 0.005180961 | 0.626304539 |
| ME3 | 1466 | 569 | Colorectal cancer (Firth correction) || id:ebi-a-GCST90013862 | cg22814480 | MR Egger | 14.50281364 | 0.998613506 | 0.030386745 | 0.062154509 |
| ME3 | 1612 | 75 | Colorectal cancer (SPA correction) || id:ebi-a-GCST90013866 | cg02493602 | MR Egger | 117.0533326 | 0.999968141 | -0.044910274 | 2.49E-12 |
| ME3 | 1782 | 289 | Colorectal cancer (SPA correction) || id:ebi-a-GCST90013866 | cg11602041 | MR Egger | 113.4779131 | 0.900202308 | 0.005084716 | 0.632806081 |
| ME3 | 1995 | 572 | Colorectal cancer (SPA correction) || id:ebi-a-GCST90013866 | cg22814480 | MR Egger | 14.50281364 | 0.998613506 | 0.030386745 | 0.062154509 |
| ME3 | 2763 | 295 | Colorectal cancer || id:ebi-a-GCST012876 | cg11602041 | MR Egger | 22.42308513 | 1 | -0.002662566 | 0.727291015 |
| ME3 | 2975 | 582 | Colorectal cancer || id:ebi-a-GCST012876 | cg22814480 | MR Egger | 3.572943612 | 1 | 0.00219894 | 0.866069598 |
| ME3 | 3119 | 75 | Colorectal cancer || id:ebi-a-GCST012877 | cg02493602 | MR Egger | 278.1048023 | 0.00912199 | -0.028723227 | 8.96E-08 |
| ME3 | 3280 | 287 | Colorectal cancer || id:ebi-a-GCST012877 | cg11602041 | MR Egger | 79.36216389 | 0.999999994 | -0.037794922 | 7.76E-06 |
| ME3 | 3499 | 573 | Colorectal cancer || id:ebi-a-GCST012877 | cg22814480 | MR Egger | 10.86062973 | 0.999995501 | -0.023378486 | 0.102752452 |
| ME3 | 3635 | 72 | Colorectal cancer || id:ebi-a-GCST012878 | cg02493602 | MR Egger | 108.0756238 | 1 | 0.081031889 | 9.61E-09 |
| ME3 | 3805 | 283 | Colorectal cancer || id:ebi-a-GCST012878 | cg11602041 | MR Egger | 38.00817952 | 1 | -0.062368739 | 0.009508028 |
| ME3 | 4015 | 568 | Colorectal cancer || id:ebi-a-GCST012878 | cg22814480 | MR Egger | 7.839159749 | 0.999999962 | 0.033242122 | 0.422441397 |
| ME3 | 4157 | 77 | Colorectal cancer || id:ebi-a-GCST012879 | cg02493602 | MR Egger | 144.0255831 | 0.999992245 | -0.007800907 | 0.056238129 |
| ME3 | 4314 | 293 | Colorectal cancer || id:ebi-a-GCST012879 | cg11602041 | MR Egger | 38.45668046 | 1 | 0.002425557 | 0.731645039 |
| ME3 | 4524 | 576 | Colorectal cancer || id:ebi-a-GCST012879 | cg22814480 | MR Egger | 7.138313843 | 0.999999991 | -0.006782151 | 0.580837842 |
| ME3 | 4662 | 75 | Colorectal cancer || id:ebi-a-GCST012880 | cg02493602 | MR Egger | 90.07512539 | 1 | -0.007782731 | 0.296242012 |
| ME3 | 4810 | 287 | Colorectal cancer || id:ebi-a-GCST012880 | cg11602041 | MR Egger | 39.39932237 | 1 | 0.021095732 | 0.11301233 |
| ME3 | 5010 | 570 | Colorectal cancer || id:ebi-a-GCST012880 | cg22814480 | MR Egger | 9.657458424 | 0.999999151 | -0.030842272 | 0.182389635 |
| ME3 | 2137 | 76 | Colorectal cancer || id:ebi-a-GCST90018588 | cg02493602 | MR Egger | 365.4933185 | 5.02E-10 | 0.024291581 | 7.40E-08 |
| ME3 | 2468 | 560 | Colorectal cancer || id:ebi-a-GCST90018588 | cg22814480 | MR Egger | 15.79885297 | 0.999110606 | -0.014931449 | 0.227643313 |
| ME3 | 571 | 74 | Colorectal cancer || id:ebi-a-GCST90018808 | cg02493602 | MR Egger | 134.5442575 | 0.9999989 | 0.003927942 | 0.138533653 |
| ME3 | 732 | 285 | Colorectal cancer || id:ebi-a-GCST90018808 | cg11602041 | MR Egger | 188.5069087 | 0.121830288 | -0.03541616 | 2.08E-09 |
| ME3 | 940 | 569 | Colorectal cancer || id:ebi-a-GCST90018808 | cg22814480 | MR Egger | 15.36133526 | 0.999909431 | -0.004127725 | 0.633486275 |
| ME3 | 5164 | 75 | Colorectal cancer || id:finn-b-C3_COLORECTAL | cg02493602 | MR Egger | 89.09284249 | 1 | 0.005616968 | 0.38507984 |
| ME3 | 5325 | 293 | Colorectal cancer || id:finn-b-C3_COLORECTAL | cg11602041 | MR Egger | 20.64450198 | 1 | -0.031981183 | 0.006010673 |
| ME3 | 5522 | 578 | Colorectal cancer || id:finn-b-C3_COLORECTAL | cg22814480 | MR Egger | 11.70539698 | 0.999998095 | 0.026163547 | 0.177889094 |
| ME3 | 220 | 288 | Colorectal cancer || id:ieu-b-4965 | cg11602041 | MR Egger | 59.82268597 | 1 | -0.000192294 | 0.108799441 |
| ME3 | 439 | 572 | Colorectal cancer || id:ieu-b-4965 | cg22814480 | MR Egger | 6.546445369 | 0.999999999 | -0.000222054 | 0.295032009 |
| SCP2 | 1631 | 96 | Colorectal cancer (SPA correction) || id:ebi-a-GCST90013866 | cg03579872 | Inverse variance weighted | 137.3708738 | 1 | NA | NA |
| SCP2 | 2615 | 98 | Colorectal cancer || id:ebi-a-GCST012876 | cg03579872 | Inverse variance weighted | 24.45925034 | 1 | NA | NA |
| SCP2 | 4679 | 96 | Colorectal cancer || id:ebi-a-GCST012880 | cg03579872 | Inverse variance weighted | 59.48277946 | 1 | NA | NA |
| SCP2 | 4170 | 98 | Colorectal cancer || id:ebi-a-GCST012879 | cg03579872 | Inverse variance weighted | 8.963350149 | 1 | NA | NA |
| SCP2 | 73 | 97 | Colorectal cancer || id:ieu-b-4965 | cg03579872 | Inverse variance weighted | 83.65146 | 1 | NA | NA |
| SCP2 | 3652 | 91 | Colorectal cancer || id:ebi-a-GCST012878 | cg03579872 | Inverse variance weighted | 11.12751838 | 1 | NA | NA |
| SCP2 | 2148 | 93 | Colorectal cancer || id:ebi-a-GCST90018588 | cg03579872 | Inverse variance weighted | 256.0342972 | 0.999771815 | NA | NA |
| SCP2 | 1101 | 92 | Colorectal cancer (Firth correction) || id:ebi-a-GCST90013862 | cg03579872 | Inverse variance weighted | 33.20324429 | 0.995087542 | NA | NA |
| SCP2 | 1100 | 91 | Colorectal cancer (Firth correction) || id:ebi-a-GCST90013862 | cg03579872 | MR Egger | 32.88921495 | 0.994157196 | -0.012483754 | 0.577453212 |
| SCP2 | 1630 | 95 | Colorectal cancer (SPA correction) || id:ebi-a-GCST90013866 | cg03579872 | MR Egger | 75.50479386 | 1 | -0.053538472 | 6.37E-14 |
| SCP2 | 2614 | 97 | Colorectal cancer || id:ebi-a-GCST012876 | cg03579872 | MR Egger | 21.00331079 | 1 | -0.009956885 | 0.06393138 |
| SCP2 | 3651 | 90 | Colorectal cancer || id:ebi-a-GCST012878 | cg03579872 | MR Egger | 11.00910659 | 1 | -0.017937618 | 0.731909401 |
| SCP2 | 4169 | 97 | Colorectal cancer || id:ebi-a-GCST012879 | cg03579872 | MR Egger | 8.789205155 | 1 | -0.008054167 | 0.677872691 |
| SCP2 | 4678 | 95 | Colorectal cancer || id:ebi-a-GCST012880 | cg03579872 | MR Egger | 52.06741345 | 1 | -0.025015717 | 0.006826233 |
| SCP2 | 2147 | 92 | Colorectal cancer || id:ebi-a-GCST90018588 | cg03579872 | MR Egger | 238.6865117 | 0.999991038 | 0.033389293 | 3.95E-05 |
| SCP2 | 72 | 96 | Colorectal cancer || id:ieu-b-4965 | cg03579872 | MR Egger | 50.74247084 | 1 | -0.000470287 | 2.20E-08 |
| MSRB2 | 3210 | 190 | Colorectal cancer || id:ebi-a-GCST012877 | cg07355157 | Inverse variance weighted | 352.3196116 | 4.94E-17 | NA | NA |
| MSRB2 | 2202 | 185 | Colorectal cancer || id:ebi-a-GCST90018588 | cg07355157 | Inverse variance weighted | 75.13150156 | 0.999999976 | NA | NA |
| MSRB2 | 5257 | 191 | Colorectal cancer || id:finn-b-C3_COLORECTAL | cg07355157 | Inverse variance weighted | 76.69731257 | 0.999999982 | NA | NA |
| MSRB2 | 1703 | 191 | Colorectal cancer (SPA correction) || id:ebi-a-GCST90013866 | cg07355157 | Inverse variance weighted | 56.70755944 | 1 | NA | NA |
| MSRB2 | 143 | 192 | Colorectal cancer || id:ieu-b-4965 | cg07355157 | Inverse variance weighted | 61.83206089 | 1 | NA | NA |
| MSRB2 | 660 | 188 | Colorectal cancer || id:ebi-a-GCST90018808 | cg07355157 | Inverse variance weighted | 48.54870432 | 1 | NA | NA |
| MSRB2 | 4741 | 190 | Colorectal cancer || id:ebi-a-GCST012880 | cg07355157 | Inverse variance weighted | 128.7587637 | 0.951799431 | NA | NA |
| MSRB2 | 2687 | 194 | Colorectal cancer || id:ebi-a-GCST012876 | cg07355157 | Inverse variance weighted | 63.52160978 | 1 | NA | NA |
| MSRB2 | 4242 | 194 | Colorectal cancer || id:ebi-a-GCST012879 | cg07355157 | Inverse variance weighted | 186.5270215 | 0.066874527 | NA | NA |
| MSRB2 | 3728 | 186 | Colorectal cancer || id:ebi-a-GCST012878 | cg07355157 | Inverse variance weighted | 145.9135065 | 0.745473829 | NA | NA |
| MSRB2 | 1167 | 187 | Colorectal cancer (Firth correction) || id:ebi-a-GCST90013862 | cg07355157 | Inverse variance weighted | 56.71101413 | 1 | NA | NA |
| MSRB2 | 1166 | 186 | Colorectal cancer (Firth correction) || id:ebi-a-GCST90013862 | cg07355157 | MR Egger | 52.56472668 | 1 | -0.008442793 | 0.043546161 |
| MSRB2 | 1702 | 190 | Colorectal cancer (SPA correction) || id:ebi-a-GCST90013866 | cg07355157 | MR Egger | 52.5663715 | 1 | -0.008437898 | 0.043673714 |
| MSRB2 | 2686 | 193 | Colorectal cancer || id:ebi-a-GCST012876 | cg07355157 | MR Egger | 57.30267753 | 1 | -0.008243436 | 0.013688798 |
| MSRB2 | 3209 | 189 | Colorectal cancer || id:ebi-a-GCST012877 | cg07355157 | MR Egger | 69.54969993 | 1 | 0.059266302 | 7.38E-37 |
| MSRB2 | 3727 | 185 | Colorectal cancer || id:ebi-a-GCST012878 | cg07355157 | MR Egger | 69.35519927 | 1 | 0.08936189 | 3.16E-15 |
| MSRB2 | 4241 | 193 | Colorectal cancer || id:ebi-a-GCST012879 | cg07355157 | MR Egger | 128.0179063 | 0.961536119 | -0.023530215 | 1.86E-12 |
| MSRB2 | 4740 | 189 | Colorectal cancer || id:ebi-a-GCST012880 | cg07355157 | MR Egger | 61.15804916 | 1 | -0.046287235 | 7.30E-14 |
| MSRB2 | 2201 | 184 | Colorectal cancer || id:ebi-a-GCST90018588 | cg07355157 | MR Egger | 41.49984782 | 1 | -0.019646218 | 3.77E-08 |
| MSRB2 | 659 | 187 | Colorectal cancer || id:ebi-a-GCST90018808 | cg07355157 | MR Egger | 48.35862178 | 1 | -0.001035154 | 0.663431949 |
| MSRB2 | 5256 | 190 | Colorectal cancer || id:finn-b-C3_COLORECTAL | cg07355157 | MR Egger | 75.78357768 | 0.999999984 | -0.004554035 | 0.340623517 |
| MSRB2 | 142 | 191 | Colorectal cancer || id:ieu-b-4965 | cg07355157 | MR Egger | 49.51973871 | 1 | -0.000178668 | 0.000591162 |
| CYB5R3 | 1641 | 108 | Colorectal cancer (SPA correction) || id:ebi-a-GCST90013866 | cg04242898 | Inverse variance weighted | 13.95133764 | 1 | NA | NA |
| CYB5R3 | 2156 | 105 | Colorectal cancer || id:ebi-a-GCST90018588 | cg04242898 | Inverse variance weighted | 14.62256217 | 1 | NA | NA |
| CYB5R3 | 596 | 105 | Colorectal cancer || id:ebi-a-GCST90018808 | cg04242898 | Inverse variance weighted | 8.624157705 | 1 | NA | NA |
| CYB5R3 | 4689 | 108 | Colorectal cancer || id:ebi-a-GCST012880 | cg04242898 | Inverse variance weighted | 25.04119244 | 1 | NA | NA |
| CYB5R3 | 3146 | 108 | Colorectal cancer || id:ebi-a-GCST012877 | cg04242898 | Inverse variance weighted | 27.98372691 | 1 | NA | NA |
| CYB5R3 | 5189 | 108 | Colorectal cancer || id:finn-b-C3_COLORECTAL | cg04242898 | Inverse variance weighted | 3.313581226 | 1 | NA | NA |
| CYB5R3 | 3664 | 103 | Colorectal cancer || id:ebi-a-GCST012878 | cg04242898 | Inverse variance weighted | 118.7860185 | 0.4109247 | NA | NA |
| CYB5R3 | 85 | 109 | Colorectal cancer || id:ieu-b-4965 | cg04242898 | Inverse variance weighted | 21.54378437 | 1 | NA | NA |
| CYB5R3 | 5688 | 107 | Colorectal cancer (all cancers excluded) || id:finn-b-C3_COLORECTAL_EXALLC | cg04242898 | Inverse variance weighted | 4.676123283 | 1 | NA | NA |
| CYB5R3 | 2625 | 110 | Colorectal cancer || id:ebi-a-GCST012876 | cg04242898 | Inverse variance weighted | 35.23536798 | 1 | NA | NA |
| CYB5R3 | 4182 | 110 | Colorectal cancer || id:ebi-a-GCST012879 | cg04242898 | Inverse variance weighted | 20.52128765 | 1 | NA | NA |
| CYB5R3 | 4262 | 222 | Colorectal cancer || id:ebi-a-GCST012879 | cg08690876 | Inverse variance weighted | 325.0313166 | 0.836474817 | NA | NA |
| CYB5R3 | 680 | 214 | Colorectal cancer || id:ebi-a-GCST90018808 | cg08690876 | Inverse variance weighted | 248.5294607 | 0.999999396 | NA | NA |
| CYB5R3 | 5762 | 217 | Colorectal cancer (all cancers excluded) || id:finn-b-C3_COLORECTAL_EXALLC | cg08690876 | Inverse variance weighted | 169.0411528 | 1 | NA | NA |
| CYB5R3 | 4759 | 216 | Colorectal cancer || id:ebi-a-GCST012880 | cg08690876 | Inverse variance weighted | 104.3793148 | 1 | NA | NA |
[truncated: 62,615 more chars]
